# A new *mib* allele with a chromosomal deletion covering *foxc1a* exhibits anterior somite specification defect

**DOI:** 10.1038/srep10673

**Published:** 2015-06-03

**Authors:** Chia-Hao Hsu, Ji-Sheng Lin, Keng Po Lai, Jing-Woei Li, Ting-Fung Chan, May-Su You, William Ka Fai Tse, Yun-Jin Jiang

**Affiliations:** 1 Institute of Molecular and Genomic Medicine, National Health Research Institutes, Taiwan; 2 Institute of Bioinformatics and Structural Biology, National Tsing Hua University, Taiwan; 3 School of Biological Sciences, The University of Hong Kong, Hong Kong; 4 School of Life Sciences, Hong Kong Bioinformatics Centre, The Chinese University of Hong Kong, Hong Kong; 5 Department of Biology, Hong Kong Baptist University, Hong Kong; 6 Biotechnology Center, National Chung Hsing University, Taiwan; 7 Institute of Molecular and Cellular Biology, National Taiwan University, Taiwan

## Abstract

*mib*^*nn2002*^, found from an allele screen, showed early segmentation defect and severe cell death phenotypes, which are different from previously known *mib* mutants. Despite distinct morphological phenotypes, the typical *mib* molecular phenotypes: *her4* down-regulation, neurogenic phenotype and cold sensitive *dlc* expression pattern, still remained. The linkage analysis also indicated that *mib*^*nn2002*^ is a new *mib* allele. Failure of specification in anterior 7-10 somites is likely due to lack of *foxc1a* expression in *mib*^*nn2002*^ homozygotes. Somites and somite markers gradually appeared after 7-10 somite stage, suggesting that *foxc1a* is only essential for the formation of anterior 7-10 somites. Apoptosis began around 16-somite stage with *p53* up-regulation. To find the possible links of *mib*, *foxc1a* and apoptosis, transcriptome analysis was employed. About 140 genes, including *wnt3a*, *foxc1a* and *mib*, were not detected in the homozygotes. Overexpression of *foxc1a* mRNA in *mib*^*nn2002*^ homozygotes partially rescued the anterior somite specification. In the process of characterizing *mib*^*nn2002*^ mutation, we integrated the scaffolds containing *mib* locus into chromosome 2 (or linkage group 2, LG2) based on synteny comparison and transcriptome results. Genomic PCR analysis further supported the conclusion and showed that *mib*^*nn2002*^ has a chromosomal deletion with the size of about 9.6 Mbp.

Mind bomb (Mib), an E3 ubiquitin ligase, regulates Notch signaling pathway by adding ubiquitin onto Notch ligands, such as DeltaC (DlC) and Jagged, on the cell membrane^1^,^2^,^3^,^4^. So far, Mib has been known as an important regulator in the Notch-related developmental processes, such as inner ear, neuron, somite, lymphocyte, hematopoiesis, retina, intestine, kidney, pancreas, etc^3^,^5^,^6^,^7^,^8^,^9^,^10^,^11^,^12^. The impacts of Mib on these developmental processes are mainly conducted through regulating Notch signaling pathway. Recently, Wnt pathway^13^, cell death pathway^14^, hypoxia signaling and deubiquitylases^15^ have also been demonstrated or proposed to be regulatory targets of Mib. Mib contains two mib/herc2 domains, one ZZ zinc finger domain, two Mib repeats, eight ankyrin repeats and three ring finger (RF) domains^1^. The most C-terminal RF domain is responsible for Mib E3 ligase activity to ubiquitylate its substrates and further lead ubiquitylated substrates to protein translocation via vesicle trafficking and/or proteasome-dependent degradation. Without ubiquitylation, Notch intracellular domain cannot be released from plasma membrane to induce downstream transcriptional activation, such as *her4* that we used to show the Notch activation in this and previous studies^1^.

Ribs and vertebrae are originally derived from the paraxial mesoderm, where a group of cells locate in presomitic mesoderm (PSM) around the region of tail bud, propagate and be specified progressively into somites – the repeated epithelial segments. Somites give rise to dermatome, myotome and sclerotome, and further become dermis, skeletal muscle, ribs and vertebrae^16^. The segmentation defect has been found to correlate with the loss of cyclic expression of *dlc* and/or *her1* in the PSM of different Notch-related zebrafish mutants, such as *deltaC* (*bea*), *deltaD* (*aei*), *notch1a* (*des*) and *mib*^1^,^17^,^18^,^19^,^20^,^21^,^22^. In these mutants, *dlc* is desynchronizedly expressed with a salt-and-pepper-like, instead of cyclic, expression pattern, and the posterior somite boundaries are indistinct as those cells are unable to be synchronized by Notch signaling^20^,^23^,^24^,^25^. On the other hand, there are genes required for somitogenesis in a Notch-independent manner. *fss* mutants with deficiency in *tbx24* gene form no somites^26^. *foxc1a*, whose morphants exhibited a similar phenotype to *fss* mutants, acts in parallel to the *fss*/*tbx24* pathway as an essential factor in zebrafish somitogenesis^27^.

To further dissect and explore the Mib functions, we carried out an allele screen to identify new *mib* alleles. Three new *mib* alleles, *mib*^*nn2000*^, *mib*^*nn2001*^ and *mib*^*nn2002*^, were identified. *mib*^*nn2000*^ and *mib*^*nn2001*^, whose mutations lead to truncation of RF domains, showed segmentation phenotypes similar to previous *mib* alleles. Interestingly, *mib*^*nn2002*^ had an earlier segmentation defect with severe cell death. *her4* and *huc* expression also suggested the loss of *mib* activity. We then focused on *mib*^*nn2002*^ mutant for its potential in identifying *mib*’s novel function. The cell death was mainly distributed around the region of notochord and neural tube with an onset from 16-somite stage (ss) and an up-regulation of *p53*. While homozygous embryos lost anterior somite segmentation and failed to be specified into somite cell fate as shown by the expression of *myoD*, *dlc and dystrophin*, relatively normal-looking posterior somite segments formed with proper somite markers expressed gradually after 14 hours post fertilization (hpf). *foxc1a* expression was not detected at all stages. The expression of *her1* and *dlc* were down-regulated but still cyclically expressed in the PSM during all developmental stages checked. By linkage analysis, we found a close linkage near the *mib* locus. Unexpectedly, we uncovered there is a large deletion covering the loci of *foxc1a* and *mib* on LG2.

## Results

### Three new *mib* alleles, showing similar Notch molecular phenotypes to other *mib* alleles, were found from allele screen

In order to further understand *mib* gene functions in the vertebrate development, we carried out an allele screen by non-complementation between F1 progeny of ENU-mutagenized founder fish and heterozygous carriers of *mib*^*ta52b*^, a well-known and widely-used *mib* allele^1^,^17^. 3849 potential F1 carriers for new *mib* alleles were successfully screened by single pair matings to fish that were *mib*^*ta52b*^ heterozygous carriers. The resulting egg lays were checked around 48 hpf for non-complementation of the *mib* mutation. Three new alleles: *mib*^*nn2000*^, *mib*^*nn2001*^ and *mib*^*nn2002*^ were identified.

**Figure 1:**
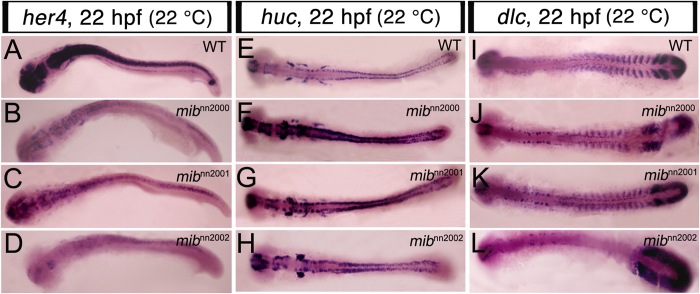
The new alleles show similar Notch molecular phenotypes to those of previous *mib* mutants.All embryos were raised at 22 °C and flat-mounted WISH performed at 22 hpf. **A**–**D** are flat-mounted *her4* WISH. (**A**) WT embryos expressed *her4* in brain and neural tube; (**B**) *mib*^*nn2000*^, (**C**) *mib*^*nn2001*^ and (**D**) *mib*^*nn2002*^ mutants all showed a down-regulation of *her4* expression. **E**–**H** are flat-mounted *huc* WISH. (**E**) WT embryos expressed *huc* in cells of brain and neural tube; (F) *mib*^*nn2000*^, (**G**) *mib*^*nn2001*^ and (**H**) *mib*^*nn2002*^ mutants all showed an up-regulation of *huc* expression. **I**–**L** are flat-mounted *dlc* WISH. (**I**) WT embryos expressed *dlc* in PSM, formed somites and cell clusters in the hindbrain; (**J**) *mib*^*nn2000*^, (**K**) *mib*^*nn2001*^ and (**L**) *mib*^*nn2002*^ mutants all showed a salt-and-pepper *dlc* pattern in the nascent somites and anterior PSM. Note that the *dlc* expression in formed somites was lost in (**L**) *mib*^*nn2002*^ mutants.

The expression of a Notch downstream gene, *her4*, and a neuronal marker, *huc*, were used to indicate the compromise of Notch signaling pathway in *mib* mutants in previous studies^1^,^7^. Besides, the disruption of cyclically expressing *dlc* was also demonstrated in *mib* and other Notch-related mutants^19^,^20^,^21^,^22^. Therefore, the expression patterns of *her4*, *huc* and *dlc* were examined in these new alleles. At 22 °C, the typical down-regulation of *her4* and up-regulation of *huc* were observed in *mib*^*nn2000*^, *mib*^*nn2001*^ and *mib*^*nn2002*^ mutants (Fig. 1A–H). Under the same condition, the *dlc* expression exhibited a salt-and-pepper (desynchronized) pattern in the anterior PSM and nascent somite region (Fig.1I–L and supplementary Fig. 1). All the analysis suggested that *mib*^*nn2000*^, *mib*^*nn2001*^ and *mib*^*nn2002*^ were new *mib* alleles that lost the E3 ubiquitin ligase activity^7^.

### *mib* ^*nn2002*^ mutants showed distinct morphological phenotypes

**Figure 2:**
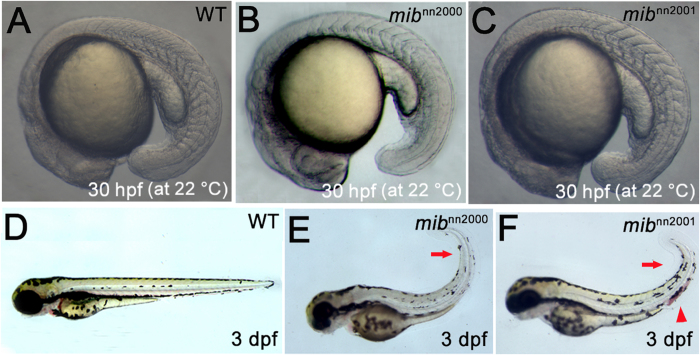
Morphological phenotypes of *mib*^*nn2000*^ and *mib*^*nn2001*^ mutants. **A**–**C** are the lateral views of 30 hpf embryos raised at 22 °C. While (**A**) WT embryos showed about 20 somites at 30 hpf, (**B**) *mib*^*nn2000*^ homozygotes showed about 11 recognizable somites and (**C**) *mib*^*nn2001*^ homozygotes showed about 14 somites. **D**–**F** are lateral views of 3 dpf embryos at 28.5 °C. Compared to (**D**) WT embryos, (**E**) *mib*^*nn2000*^ and (**F**) *mib*^*nn2001*^ mutants showed a curly tail (arrows), hemorrhage (arrowhead) and a reduction of tail pigmentation (more prominent in **E**).

**Figure 3:**
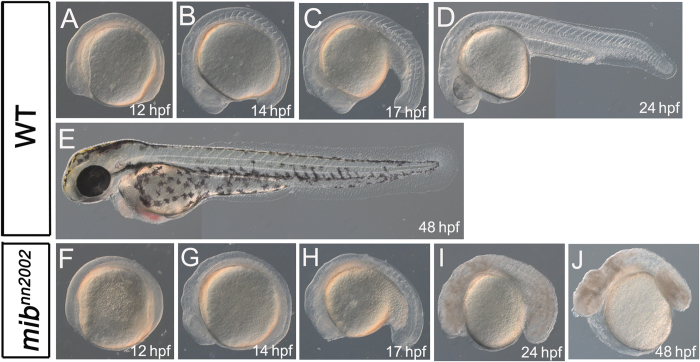
*mib*^*nn2002*^ mutants show phenotypes distinct from those of typical *mib* mutants. **A–E** are lateral views of WT embryos. **F**–**J** are lateral views of *mib*^*nn2002*^ homozygotes. **A** and **F** are at 12 hpf; **B** and **G** are at 14 hpf; **C** and **H** are at 17 hpf; **D** and **I** are at 24 hpf; **E** and **J** are at 48 hpf. No visible somites can be observed from the lateral view of *mib*^*nn2002*^ homozygotes at (**F**) 12 hpf and (**G**) 14 hpf. Visible somite-like structures can be discerned from the lateral view of *mib*^*nn2002*^ homozygotes at (**H**) 17 hpf and (**I**) 24 hpf. Cell death can be spotted in *mib*^*nn2002*^ homozygotes at (**I**) 24 hpf and (**J**) 48 hpf. Edema was obvious in *mib*^*nn2002*^ homozygotes at (**J**) 48 hpf.

Both *mib*^*nn2000*^ and *mib*^*nn2001*^ mutants showed typical *mib* segmentation defects at 22 °C: showing 11-14 and 14-17 visible somites, respectively, compared to about 20 somites (equivalent to 19 hpf at 28.5 °C) in wild-type at 30 hpf (Fig. 2A–C). Besides, *mib*^*nn2000*^ and *mib*^*nn2001*^ exhibited morphological phenotypes similar to those of *mib*^*ta52b*^ and *mib*^*m132*^ 1,17, including brain and pigmentation defects, curly tail and hemorrhage at 3 dpf (Fig. 2D–F). Compared with the later boundary formation defect in *mib*^*nn2000*^, *mib*^*nn2001*^and previously-analyzed *mib* mutants, *mib*^*nn2002*^ showed somite segmentation defect at an earlier stage (Fig. 3). The V-shaped somite boundaries were not observed in *mib*^*nn2002*^ before 14 hpf (Fig. 3G). The somite-like structures could be observed in *mib*^*nn2002*^ mutants after 17 hpf, though the somites were not V-shaped and boundaries did not form sharply (Fig. 3H [Fig f4] and Fig. 5F). Necrotic tissues appeared in *mib*^*nn2002*^ mutants after 24 hpf (Fig. 3I) and the phenotype became worse after 48 hpf with a great amount of cell death, edema and laterally curly tail with no observable pigmentation (Fig. 3J). Because of the distinct morphological phenotypes and similar Notch-related molecular phenotypes to other *mib* alleles, we then focused on analyzing *mib*^*nn2002*^ mutants as it might reveal a novel function of *mib*.

Noticeably, average 22% of embryos from incrosses of *mib*^*nn2002*^ carriers exhibited early segmentation phenotype (supplementary Table 1). The pre-selected embryos with early segmentation phenotype showed down-regulated *her4*, slightly up-regulated *huc* and severe cell death phenotypes (data not shown). Furthermore, about a quarter of transheterozygotes (*mib*^*nn2002*^/*mib*^*ta52b*^) exhibited typical *mib* phenotypes (supplementary Table 2), such as late segmentation defect (supplementary Fig. 2), curly tail and less pigmentation (supplementary Fig. 3). The impact of maternal *mib* on the transheterozygotes also can be observed (supplementary Fig. 3, embryos laid from female *mib*^*ta52b*^ carriers show a more severe *mib* phenotype, as observed previously^7^). These results demonstrated that *mib*^*nn2002*^ mutation follows the patterns of autosomal recessive inheritance with high penetrance.

The cell death in *mib*^*nn2002*^ mutants began after 17 hpf by acridine orange staining and mainly distributed around the regions of neural tube and notochord (supplementary Fig. 4). It was demonstrated to be apoptosis by using TUNEL assay and the apoptotic cell number was significantly higher in *mib*^*nn2002*^ mutants than that in the control embryos (supplementary Fig. 5). The expression of *p53* was further found to be highly up-regulated in *mib*^*nn2002*^ mutants (supplementary Fig. 6), which suggests that cell death might be related to genome instability.

### Early somite specification defects were identified in *mib* ^*nn2002*^ mutants

To discern if the segmentation defect is caused by a failure/delay in boundary formation or early somite specification, we checked the *dlc* expression first, because the disruption of cyclic expression pattern of Notch components was found highly correlated to the defect of somite boundary formation in Notch-related mutants^20^,^28^,^29^,^30^,^31^. The expression of *dlc* in *mib*^*nn2002*^ mutants under 28.5 °C incubation were examined from 12 hpf (~5 ss) to 19 hpf (20 ss). Unexpectedly, there was no obvious *dlc* desynchronization detected in the PSM, suggesting that the segmentation defect might be caused by a Notch-independent mechanism (supplementary Fig. 7). This result may be contradictory to the Notch role in somite segmentation at first glance. However, it is consistent with a previously-analyzed *mib* null allele, *mib*^*tfi91*^, which shows regular boundary formation and normal *dlc* cycling at 28.5 °C but becomes cold-sensitive and exhibits somite boundary defect and desynchronized *dlc* expression pattern at 22 °C (compared to Fig. 1L; supplementary Fig. 1; reference 7).

**Figure 4 f4:**
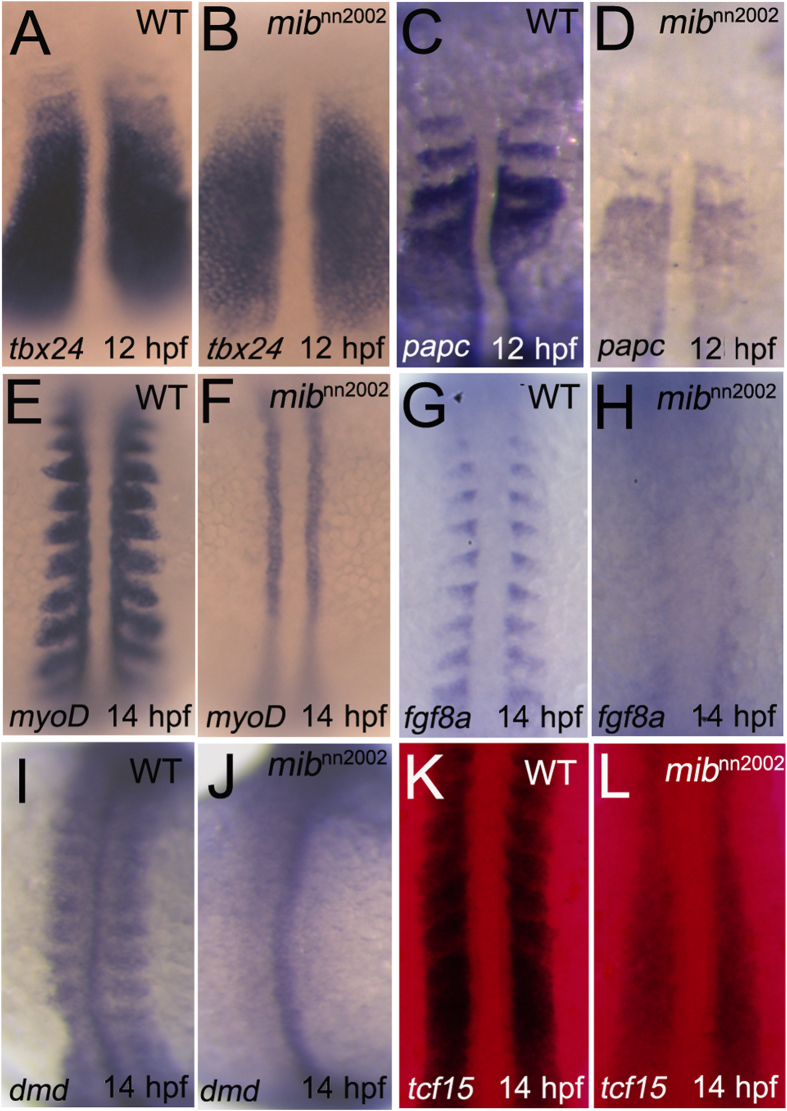
Somite/boundary markers are lost in *mib*^*nn2002*^ mutants. (**A**) WT embryos and (**B**) *mib*^*nn2002*^ mutants were stained with *tbx24* at 12 hpf. (**C**) WT embryos and (**D**) *mib*^*nn2002*^ mutants were stained with *papc* at 12 hpf. (**E**) WT embryos and (**F**) *mib*^*nn2002*^ mutants were stained with *myoD* at 14 hpf. (**G**) WT embryos and (**H**) *mib*^*nn2002*^ mutants were stained with *fgf8a* at 14 hpf. (**I**) WT embryos and (**J**) *mib*^*nn2002*^ mutants were stained with *dystrophin (dmd)* at 14 hpf. (**K**) WT embryos and (**L**) *mib*^*nn2002*^ mutants were stained with *tcf15* at 14 hpf, where DsRed filter was used to improve the signal to noise ratio of NBT/BCIP staining under transmitted light. All embryos were mounted in dorsal view with head to the top. *tbx24* and *papc* were expressed in PSM, though the anterior segmental pattern was not observed in the region of nascent somites in *mib*^*nn2002*^ mutants. The expression of *myoD*, *fgf8a*, and *dystrophin* were not detected in the region where somites normally formed. *tcf15* was weakly expressed in *mib*^*nn2002*^ mutants and no segmental pattern can be discerned.

We further used *tbx24* and *papc*, two boundary markers in the region of nascent somites^26^,^32^. The expression of *tbx24* was steadily expressed in the PSM, only lost in the anterior nascent somites in *mib*^*nn2002*^ mutants at 12 hpf (Fig. 4A–B). Similar phenomenon was also observed by using *papc*: two anterior nascent somite boundaries were lost in *mib*^*nn2002*^ mutants (Fig. 4C– D). Next, posterior somite marker, *myoD*^18^, anterior somite marker, *fgf8a*^33^, somite boundary marker, *dystrophin*^34^ and *tcf15* (*paraxis*^27^) were used. The expression of *myoD*, *fgf8a*, *dystrophin* and *tcf15* were lost or diminished in the region where somites normally form in *mib*^*nn2002*^ mutants at 14 hpf (Fig. 4E–L). Among these markers, only *myoD* was detected in the adaxial cells. These results indicate that the early somite segmentation defect in *mib*^*nn2002*^ is caused by a failure in cell fate specification. This also suggests that the early segmentation defect in *mib*^*nn2002*^ is mediated through a Notch-independent pathway.

### *foxc1a* is not essential in later somite segmentation and specification

**Figure 5:**
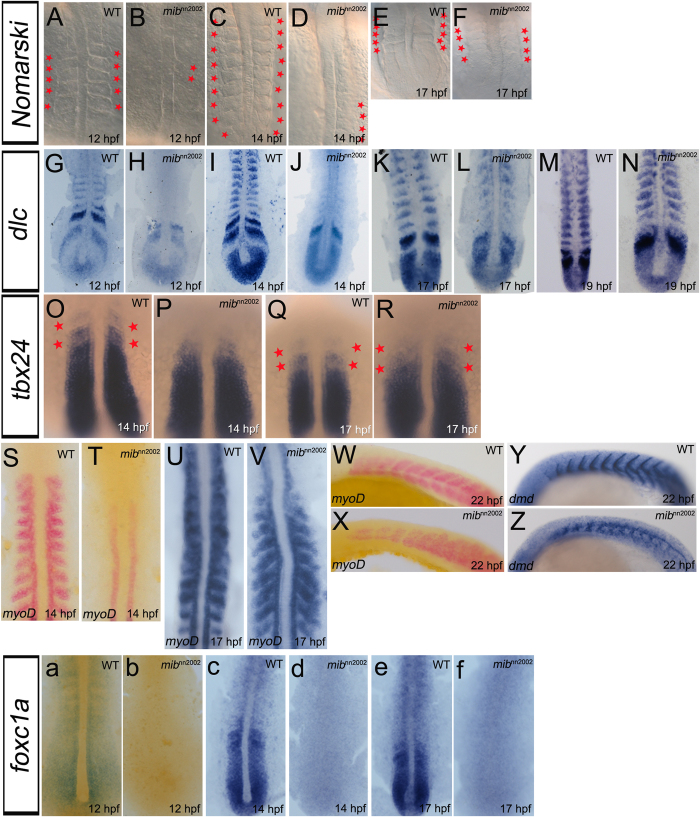
Somite segmentation and specification recover without *foxc1a* expression. **A–F** are dorsal views of WT embryos and *mib*^*nn2002*^ mutants. Small round-shaped structures can be observed in (**B**) *mib*^*nn2002*^ homozygotes at 12 hpf. The newly-generated somite-like structures increased when *mib*^*nn2002*^ homozygotes grew to (**D**) 14 hpf and (**F**) 17 hpf. **G–N** are flat mounts of WT embryos and *mib*^*nn2002*^ mutants stained with *dlc*. (**H**) The expression of *dlc* in the region where somites normally formed was not observed in *mib*^*nn2002*^ homozygotes at 12 hpf. (**J**, **L** and **N**) *dlc* progressively appeared after 14 hpf in the newly-generated somites. **O–R** are dorsal views of WT embryos and *mib*^*nn2002*^ mutants stained with *tbx24*. The segmental *tbx24* (marked with red asterisks) appeared progressively in *mib*^*nn2002*^ homozygotes. **S–V** are flat mounts of WT embryos and *mib*^*nn2002*^ mutants stained with *myoD*. **W** and **X** are lateral views of WT embryos and *mib*^*nn2002*^ mutants stained with *myoD*. The expression of *myoD* in formed somites started to emerge after 14 hpf as what was observed in *dlc*. As embryos developed, (**V**) the expression of *myoD* progressively appeared in the region where somites normally formed, except the anterior segments. (**X**) The cells that expressed *myoD* still split into dorsal and ventral myotomes even where somite boundaries were not formed. **Y** and **Z** are lateral views of WT embryos and *mib*^*nn2002*^ mutants stained with *dystrophin* (*dmd*) at 22 hpf. The expression of *dystrophin* can be observed in formed somites. Note that the somite gap delineated by *dystrophin* in formed somite region is similar between (**Y**) WT embryos and (**Z**) *mib*^*nn2002*^ mutants. **a**–**f** are dorsal views of WT embryos and *mib*^*nn2002*^ mutants stained with *foxc1a*. The expression of *foxc1a* in *mib*^*nn2002*^ homozygotes are not detected in all the stages examined (**b** 12 hpf; **d** 14 hpf and **f** 17 hpf).

Although visible somites were hardly observed in *mib*^*nn2002*^ at early stages, the somite-like structures did appear at later stages. To explore the phenotype, we analyzed the somite-like structures of *mib*^*nn2002*^ mutants in more details. Some small round-shaped structures in the region where somites were supposed to form at early stage were found in *mib*^*nn2002*^ mutants (Fig. 5A–B). The number of the small round-shaped structures increased with time in *mib*^*nn2002*^ mutants (Fig. 5A–D). The somites formed in *mib*^*nn2002*^ mutants at a relative late stage have a shape much similar to those observed in wild type (WT) (Fig. 5E–F). In order to understand whether the somites are really formed at later stages, we further examined the expression of *dlc, tbx24*, *myoD* and *dystrophin* at different stages. The expression of *dlc*, highly correlated to the formation of somite-like structures, was shown in the region of formed somites progressively in *mib*^*nn2002*^ mutants (Fig. 5G–N). The cyclically expressed *dlc* in the PSM was not altered as described previously (supplementary Fig. 7). Its expression level in the PSM was gradually recovered at later stages, in contrast to the severe down-regulation at early stages (Fig. 5G–N). Similarly, the anterior segmented *tbx24* expression pattern was progressively restored (Fig. 5O–R). *myoD* was also expressed in the formed somites with sharp and striped pattern while the normal-looking somites started appearing after 14 hpf (Fig. 5S–X). The expression of *dystrophin* also suggested that somites develop into myotome with roughly normal somite boundaries at 22 hpf (Fig. 5Y–Z). All the results suggest that somites are normally specified in *mib*^*nn2002*^ mutants at a relative late somitogenesis stage.

*foxc1a* has been shown to be an essential factor for somitogenesis and somite segmentation in a morpholino study^27^. Therefore its expression was examined. Surprisingly, the expression of *foxc1a* was not detected in *mib*^*nn2002*^mutants at all stages examined (Fig. 5a–f). These data indicated that *foxc1a* is only essential for anterior somitic cell fate determination, but not required for the later somite specification.

### *mib* ^*nn2000*^ and *mib* ^*nn2001*^ mutants both encode a Mib protein without RF3 domain

**Figure 6:**
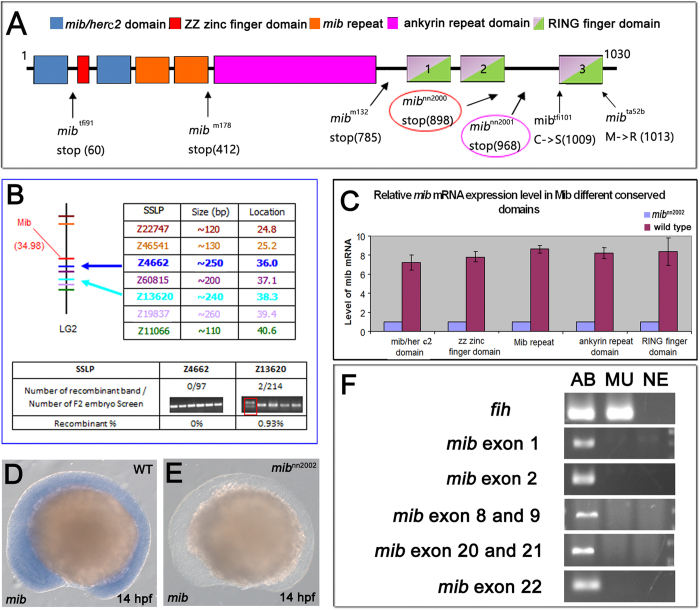
*mib* gene is deleted in *mib*^*nn2002*^ mutants. (**A**) Diagram of the mutations of different *mib* alleles. The translation of *mib* in *mib*^*nn2000*^ and *mib*^*nn2001*^ mutants stops at amino acids 5’ to ring finger domain 3 (RF3). (**B**) The linkage analysis result of *mib*^*nn2002*^ mutants. The upper part illustrates the locations of *mib* gene and the SSLP markers. The lower part demonstrates a close linkage of Z4662 and Z13620 to *mib*, with a recombination frequency of 0/97 and 2/214, respectively. (**C**) Real-time PCR results of *mib* expression in *mib*^*nn2002*^ homozygotes and WT embryos. The blue bars represent the expression level of *mib* in *mib*^*nn2002*^ homozygotes. The purple bars represent the expression level of *mib* in WT embryos. There is nearly no *mib* transcript detected in the *mib*^*nn2002*^ homozygotes. (**D**) WT embryos and (**E**) *mib*^*nn2002*^ mutants were stained with *mib* at 14 hpf, respectively. There is no *mib* mRNA detected in (**E**) *mib*^*nn2002*^ homozygous. (**F**) The results of *mib* genomic PCR were shown with *fih* (*hif1an*) as a positive control. AB represents AB WT samples; MU represents *mib*^*nn2002*^ homozygous samples; NE represents negative controls, where distilled water was used instead of genomic extracts as templates. The results showed that no *mib* genomic fragments are amplified.

To identify the mutations in these three new alleles, the *mib* mRNA was reversely transcribed, amplified and examined by sequencing. *mib*^*nn2000*^ bore a T to A transversion mutation at the position 2694 from translational start site, resulting in a nonsense mutation C-terminal to RF2 (Fig. 6A and supplementary Fig. 8A). *mib*^*nn2001*^ resulted in a different splicing isoform (*mib*ΔRF3) that has a 143 bp insert after position 2851 from translational start site, leading to a truncated protein without the RF3 domain (Fig. 6A and supplementary Fig. 8B). The characterization and mutation identification of *mib*^*nn2000*^ and *mib*^*nn2001*^ are comparable to the results from *mib*^*m132*^ and *mib*^*ta52b*^, which have RF123 domain deletion and a non-functional RF3, respectively^1^,^7^.

### *mib* gene is deleted in *mib* ^*nn2002*^ mutants

Difficulty was encountered while we were trying to amplify the coding sequence of *mib* from *mib*^*nn2002*^ mutants. Linkage analysis was then employed to assure the position of *mib*^*nn2002*^ mutation and it was demonstrated that *mib*^*nn2002*^ mutation is 3.32 cM away from the *mib* locus in chromosome 2 (LG2) with SSLP marker Z13620 (Fig. 6B). Therefore, *mib*^*nn2002*^ is indeed a new *mib* allele.

The phenocopy and rescue experiments were then used to further discern two hypotheses of the cause of *mib*^*nn2002*^ mutants. First, *mib*^*nn2002*^ mutation might encode a mutated Mib protein that leads to cell death and early segmentation defect in homozygous embryos. *mib* morpholino has been previously used in phenocopying *mib*^*tfi91*^ by knocking down *mib*^*ta52b*^ transcript^7^. By using the same approach, morpholino can down-regulate the expression of the hypothetical mutated *mib*^*nn2002*^ transcript and phenocopy the *mib* null allele, *mib*^*tfi91*^. Second, *mib*^*nn2002*^ might bear a mutation in the *mib* regulatory region that affects its expression level. If so, injecting *mib* mRNA into *mib*^*nn2002*^ should be able to rescue the *mib*^*nn2002*^ phenotypes to some extent. However, both hypotheses were proved to be incorrect. Although mRNA injection partially restored the expression level of *her4* and *huc* and morpholino injection led to an increase in the number of embryos that exhibit *mib*^*ta52b*^-like phenotypes (supplementary Fig. 9), the early segmentation defects and cell death phenotypes in *mib*^*nn2002*^ homozygotes were neither rescued nor alleviated and the ratio of *mib*^*nn2002*^ homozygotes remained unchanged in both rescue and phenocopy experiments (supplementary Fig. 9  and data not shown).

By checking the expression of *mib* with *in situ* hybridization and real-time PCR, we found that the expression of *mib* was hardly detected and might be totally lost in *mib*^*nn2002*^ mutants (Fig. 6C–E) and, thereby, started to wonder whether *mib* was deleted in *mib*^*nn2002*^ mutants. To explore the possibility, genomic PCR was employed to check the existence of *mib* gene. However, the genomic sequence of *mib* was separated on two scaffolds, 3504 and 3540, and was not placed in chromosome 2 of Zebrafish Ensembl (Zv9, http://www.ensembl.org/Danio_rerio/Info/Index). Moreover, the 5’ and 3’ genomic sequences of *mib* were separated by other genes. To further clarify the assembly/continuity of the scaffolds, we first used existing *mib* mutants, *mib*^*tfi91*^ and *mib*^*nn2000*^, to verify the continuity of the genomic sequence. The results showed that *mib*^*tfi91*^ and *mib*^*nn2000*^ mutations can be identified based on the genomic sequence of scaffolds 3504 and 3540, respectively (supplementary Fig. 10), suggesting that scaffolds 3504 and 3540 are continuous. We assembled the genomic *mib* sequence from scaffolds 3504 and 3540 and delimited the intron-exon boundaries with the help of cDNA sequence (supplementary File 11). According to the assembled *mib* genomic sequence, the primers were designed and used to examine the existence of *mib* in *mib*^*nn2002*^ with *fih*, a gene not located on LG2, as a positive control. Consistent with our speculation, *mib* could not be amplified from the genomic DNA of *mib*^*nn2002*^ mutants (Fig. 6F).

### *mib* ^*nn2002*^ mutants have an arm deletion in chromosome 2, including the loci of *mib* and *foxc1a*

**Figure 7:**
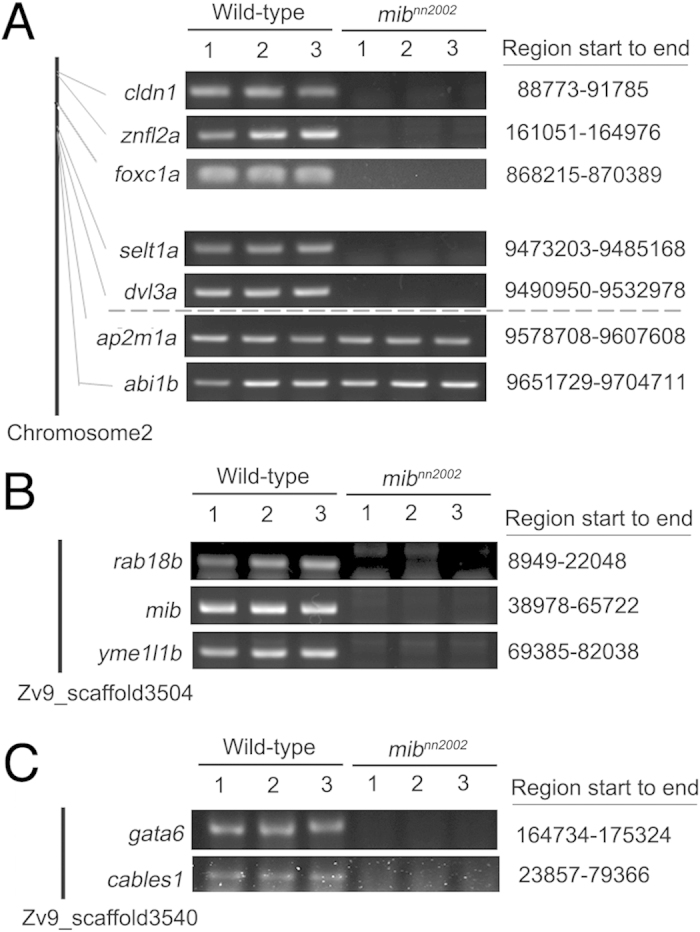
An arm of LG2, including *mib* gene, is truncated in *mib*^*nn2002*^ mutants. (**A**) The genomic PCR results of WT embryos and *mib*^*nn2002*^ mutants on LG2. Genes located near the chromosome terminal, *cldn1*, *znfl2a* and *foxc1a*, and near the suspected breaking point, *selt1a*~*abi1b*, were examined. The genes located between 88773 ∼ 9532978 bp on LG2 were not amplified in *mib*^*nn2002*^ mutants. (**B**) The genomic amplification of 5’-*mib*, *rab18b* and *yme1l1b* of scaffold 3504. (**C**) The genomic amplification of *gata6* and *cables1* of scaffold 3540. All these genes were not amplified in *mib*^*nn2002*^ mutants (see supplementary Fig. 12 and supplementary Table 4 for a more complete set of data).

In order to dissect the link between *mib*, *foxc1a* and the cell death phenotype in *mib*^*nn2002*^ mutants, we performed an RNA-seq analysis on *mib*^*nn2002*^ mutants and AB WT embryos using the Mi-Seq platform. The detailed data will be reported elsewhere. Here, we only present the genes in the chromosome 2. From the RNA-seq, an unexpected result was found. Around 140 transcripts that are located between 19233 and 9578708 in LG2 were not detected (mean expression value <0.5 as set-point) only in the mutants. The full list of LG2 can be found in the supplementary Table 3. Selected transcripts were undergone genomic PCR for confirmation, which included both ends of the truncated region: *cldn1* (88773-91785) and *dvl3a* (9490950-9532978) (Fig. 7, supplementary Fig. 12). The result (summarized in supplementary Table 4) indicated that probably there is an arm deletion in the chromosome 2 of *mib*^*nn2002*^ mutants and the breaking point is between *dvl31* and *ap2m1a* (9532978-9578708) (OR 1133-1144 from zfin *mib* map ch2, http://zfin.org/cgi-bin/view_mapplet.cgi). To conclude, *mib*^*nn2002*^ mutants have a regional deletion in the genome, where part of the chromosome 2, including *mib* and *foxc1a,* is deleted.

### The missing genes may play roles in somitogenesis and apoptosis

We further underwent DAVID analysis (see Materials and Methods) on the missing genes in *mib*^*nn2002*^ mutants. 91 genes had the DAVID ID, and 63 of them matched the zebrafish ID. The relatively low matching might be due to the present unknown genes in the list, such as those named as zebrafish gene collection (zgc) and CABZ sequences from clones or whole genome shotgun contigs. Nevertheless, the analysis identified ten enriched annotation clusters in *mib*^*nn2002*^ mutants. For example, somitogenesis and segmentation were identified in the analysis, which matched our molecular and phenotypic data in the mutants. In addition, signalings that are critical in early embryogenesis, such as Hedgehog and Wnt signaling pathways were deregulated (supplementary Table 5). All these results further support the phenotypic analysis of *mib*^*nn2002*^ mutants.

**Figure 8:**
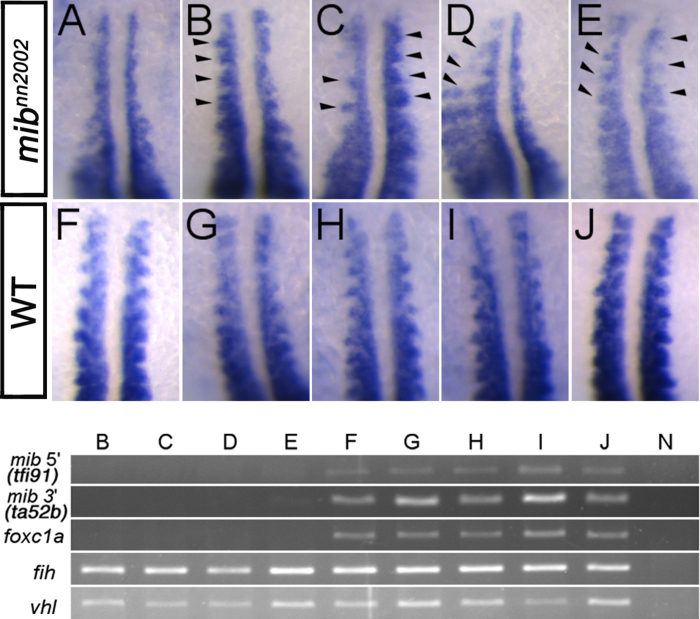
Anterior *myoD* expression in somites is partially rescued by *foxc1a* overexpression at 16 hpf.Representative WISH results of (**A**) non-injected control *mib*^*nn2002*^ mutants, (**B–E**) 20 pg *foxc1a* mRNA injected *mib*^*nn2002*^ mutants and (**F–J**) 20 pg *foxc1a* mRNA injected WT (siblings) are shown. The rescued somites are marked by arrowheads. *myoD* expression in the anterior somites of **E** was rescued symmetrically, while those of **B**–**D** were rescued asymmetrically in terms of *myoD* expression pattern, which may be caused by the unequal segregation of injected *foxc1a* mRNA. Lower panel shows the genotyping results of the embryos shown in **B**–**J**. The genomic fragments of *mib* 5’ (amplified by tfi91 primer set), *mib* 3’ (amplified by ta52b primer set) and *foxc1a* were not detected in **B**–**E**, while they were amplified in **F**–**J**. *fih* and *vhl* were amplified in all samples except for the negative control N. None of the genomic fragments was detected in the negative control.

To confirm the causal consequence of *foxc1a* deficiency in anterior somite segmentation phenotype found in *mib*^*nn2002*^ mutants, *foxc1a* mRNA was injected into *mib*^*nn2002*^ mutants and found to be able to partially rescue the anterior *myoD* expression in *mib*^*nn2002*^ mutants at 16 hpf (Fig. 8). Two major rescued features of *myoD* expression in the anterior somites were segmental (Fig. 8B–E) and laterally-extended (prominently seen in Fig. 8D) *myoD* pattern, compared to Fig. 5V and Fig. 8A. In the first experiment, 111 embryos were collected for 16 hpf *myoD* expression pattern analysis. 22 (20% of total embryos) partially rescued or non-rescued like *mib*^*nn2002*^ homozygotes and 23 WT-like siblings were selected for imaging and genotyping. The 22 homozgyote-like embryos and the 23 WT-like embryos were all genotyped as *mib*^*nn2002*^ homozygotes and WT siblings, respectively. Among these 22 homozygotes, 6 *foxc1a*-overexpressed homozygotes showed a partially rescued *myoD* expression pattern as shown in Fig. 8B–E (27% of selected homozygotes). To better understand the rescue spectrum, we analyzed all the 38 injected embryos from the second set (supplementary Fig. 13 and data not shown). There were 9 embryos genotyped as homozygotes (supplementary Fig. 13B), 28 as WT and one as unknown (none of the primer sets worked, though the *myoD* expression pattern suggested that it is a homozygous mutant). Therefore, 24% (9/37) embryos are *mib*^*nn2002*^ homozygous. 4 (44%) homozygotes were partially rescued (supplementary Fig. 13Ac, d, h, i) and 3 (33%) homozygotes were almost fully rescued (supplementary Fig. 13Aa, e, f). These results strengthen the notion that *foxc1a* is only required for anterior somite segmentation.

*selt1a* and *scp2a* (related to oxidative damage) and *smc6* (related to chromosome structure maintenance) were identified from the deleted genes in *mib*^*nn2002*^ mutants. This finding suggests that DNA damage and chromosome instability may be the cause of *p53* up-regulation (supplementary Fig. 6) and further lead to cell death in *mib*^*nn2002*^ mutants. To explore this possibility, γ-H2AX staining was employed, because phosphorylated H2AX (γ-H2AX) can be spotted shortly after the induction of DNA double-stranded breaks (DSBs) and is essential for recruiting several DNA repair proteins to the DNA damage sites^35^. Indeed, many γ-H2AX foci can be detected in *mib*^*nn2002*^ mutants in contrast to control embryos (supplementary Fig. 14). Moreover, we injected *p53* morpholino into *mib*^*nn2002*^ mutants and found that dying cells are decreased, suggesting that cell death in *mib*^*nn2002*^ mutants is *p53*-dependent (supplementary Fig. 15).

## Discussion

Previously, *mib* was found to be an essential factor in Notch signaling pathway. The loss of *mib* function will lead to the failure of adding ubiquitin onto Notch ligands and then the down-regulation or loss of Notch downstream gene expression. To further explore the functions of *mib*, we carried out an allele screen and found three new *mib* alleles, *mib*^*nn2000*^, *mib*^*nn2001*^ and *mib*^*nn2002*^. *mib*^*nn2000*^ and *mib*^*nn2001*^ mutants exhibited typical *mib* phenotypes, including disruption in somite boundary formation, curly tail and down-regulation of Notch target genes. Interestingly, *mib*^*nn2002*^ mutants showed defects in early somite boundary formation and cell death. Although *mib*^*nn2002*^ mutants showed similar Notch molecular phenotypes as other *mib* mutants, their morphological phenotypes were demonstrated to be unrelated to the loss of *mib* in *mib*^*nn2002*^ mutants, because it was not rescued by *mib* or *mib*2 overexpression. Instead, the loss of *foxc1a* is more likely to be the cause of the anterior segmentation defect. In a previous morpholino study, the down-regulation of *foxc1a* specifically leads to the loss of *myoD* expression in the region where somites were supposed to form and does not affect the expression of *myoD* in the adaxial cells^27^. The failure of somite segmentation was not caused by the disruption of cyclic *dlc* expression in the PSM as the *dlc* is normally and cyclically expressed in the PSM of *mib*^*nn2002*^ mutants. Furthermore, *mib*^*nn2002*^ mutation contained an arm deletion and lost around 140 genes, including *mib* and *foxc1a*, on LG2. Therefore, it is possible that the failure of somite specification is caused by the loss of *foxc1a*. In other words, anterior somite segmentation defect found in *mib*^*nn2002*^ mutants is caused by a Notch-independent pathway.

*mib*^*nn2002*^ is probably a spontaneous deletion allele that lacks *mib* and other genes, because ENU mutagenesis mainly induces random mutations and produces almost all possible nucleotide substitution changes (transversion and transition), including nonsense mutations (e.g. *mib*^*nn2000*^), mutations that affect transcript splicing (e.g. *mib*^*nn2001*^) and missense mutations^36^. Interestingly, *syu*^*t4*^ has been identified as a spontaneous deletion allele deleting *shh* gene. Similar to *mib*^*nn2002*^ mutants, *syu*^*t4*^ mutants also showed a brain necrosis phenotype, which may be due to other genes affected by the deletion that has not been delimited and characterized^37^.

In the transcriptome study, three genes related to somite development were found: *mib*, *foxc1a* and *wnt3a*. The participation of *mib* in the anterior somite formation is excluded, because the null allele (*mib*^*tfi91*^) did not exhibits early boundary formation defect, at least at 28.5 °C^7^. *wnt3a* is unlikely responsible for the somite defect, either, for two reasons. First, mouse *Wnt3a* regulates cyclic Notch signaling through one bHLH transcription factor, Mesogenin1 (Msgn1)^38^; however, cyclic *dlc* expression is still observed in *mib*^*nn2002*^ mutants (supplementary Fig. 7). Second, *wnt* signaling does not appear involved in modulating segmentation clock but wavefront velocity in zebrafish^39^.

It was believed that *foxc1a* is crucial for the process of somite segmentation, because morpholino was able to inhibit somite formation before 7 ss but Foxc1a level was restored after 7 ss due to a negative feedback regulation of Foxc1a on transcription of its own gene^27^. Similar study was also carried out in compound *Foxc1; Foxc2* homozygous mice (zebrafish did not have *foxc2*). It was found that mouse anterior somites (9.5 dpc with about 20 somites; with greater than 60 somites in total after 15 dpc) are not formed at 9.5 dpc; however, the formation of the posterior somite cannot be analyzed because the mice died far too early^40^. In our study, the expression of *foxc1a* was lost as it is deleted in the genome of *mib*^*nn2002*^ mutants. Nevertheless, the somite segmentation and boundary formation were relatively well processed without *foxc1a* at a relative late stage. Importantly, the anterior *myoD* expression pattern could be partially rescued in *mib*^*nn2002*^ mutants by overexpressing *foxc1a*. These results, therefore, demonstrate that *foxc1a* is only required for the formation of anterior somites but not for that of posterior somites.

As mentioned early, the genomic sequence of *mib* was placed on two scaffolds. Although the two scaffolds were proved to be the sequence encoded *mib* genomic DNA, they failed to be assembled into one as there are two genes, *rab18b* and *Loc100331480 (PTCHD3 (3 of 3)* located between the 5’ and the 3’ parts of *mib* genomic sequences (supplementary Fig. 10). Through comparing synteny of genomic DNA from different species, including Cavefish, Tilapia, Tetraodon, Stickleback, Platyfish, *Fugu*, Medaka, Cod, *Xenopus*, Mouse and Human, none of the *mib* genomic DNA is separated by *rab18b* in Ensembl database (data not shown). Besides, there is no overlap between the shotgun sequences, cu694527 and cu681854, which constitute scaffold 3504. Therefore, we propose that the shotgun sequence cu694527 should be removed and then the scaffold 3504 and scaffold 3540 can be assembled into one scaffold (supplementary Fig. 16).

On the other hand, the position of *mib* gene was suggested to be located on LG2 through different mappings, such as T51, LN54 and MGH genetic maps, based on the data in ZFIN genetic maps (http://zfin.org/action/mapping/all-panels?record=JUMPTOREFCROSS). However, the genomic sequence of *mib* was not placed in any chromosome in zebrafish. To unravel the ambiguity, we analyzed and compared the synteny of genes near the *mib* locus and found that the synteny in zebrafish is much more similar to that in cavefish (supplementary Fig. 17), as in the case of *hox* gene clusters demonstrated previously^41^. Therefore, we used the information of these syntenies and the existed Ensembl sequences to rebuild a hypothetical genomic structure of LG2 near the locus of *mib* (supplementary Fig. 18). In the hypothetical genomic structure of LG2, nearby *mib* locus are the genes, such as *mkxb*, *rab18b*, *ptchd3*, *yme1l1b*, *gata6* and *cables1*. The transcripts and genomic sequences of those nearby genes were also not detected in *mib*^*nn2002*^ mutants (Fig. 7; supplementary Fig. 12; supplementary Table 4).

## Materials and Methods

### Zebrafish

Zebrafish was fed and maintained with the help of Taiwan Zebrafish Core Facility at NHRI (http://www.zebrafish-nthu-nhri.org/chinese/index.php). The maintenance followed the standard operation protocol of the core facility. Embryos were raised at 28.5 °C unless otherwise specified. The allele screen was performed according to a published protocol^37^. All experimental procedures on zebrafish were approved by the Institutional Animal Care and Use Committee, National Health Research Institutes, Taiwan (NHRI-IACUC-100028) and carried out in accordance with the approved guidelines.

Laid embryos that were used in the whole mount *in situ* hybridization experiment (WISH) and live imaging were collected within a 15 min interval after mating started. The collected embryos were selected from pooled quality eggs right after the collection and sorted into 50 ∼ 55 embryos per 10 cm dish with E3 egg water to avoid developmental delay. The embryos was staged as described^42^ and phenotypically checked by comparing with WT or WT-like control embryos (the siblings) that were collected in the same time windows. The homozygotes were identified and collected into different vials according to their developmental stages. Embryos were then fixed in 4% paraformaldehyde and kept in fridge for 1 ∼ 3 days. Afterwards, embryos were dechorionated, dehydrated by methanol and stored in freezer.

### Phenocopy, rescue and morpholino experiments

For *mib* phenocopy and rescue experiments, 10 ng/μl of *mib* exon1/intron morpholino^1^ and 1 ng/μl *mib1* mRNA or *mib2* mRNA were injected into embryos around 1 ∼ 2 cell stage, respectively. Embryos were placed in 28.5 °C incubator before the desired stage.

For *foxc1a* rescue or overexpression experiments, 20 pg zebrafish *foxc1a* mRNA was injected into embryos of one-cell stage. Embryos were raised at 28.5 °C before the desired stage.

For *p53* knockdown experiments, 15.2 ng *p53* morpholino^43^ was injected into the embryos of one-cell stage. Embryos were raised at 28.5 °C before the desired stage.

### Whole mount *in situ* hybridization (WISH)

RNA probes were produced with digoxigenin or fluorescein labeling mix (Roche). *huc*, *her4*, *myoD*, *dlc*, *tbx24*, *her1* and *mib* plasmids were used as templates^26^,^44^,^45^,^46^,^47^. The templates of *dystrophin*, *foxc1a*, *fgf8a*, *papc*, *tcf15* and *p53* were generated by RT-PCR with primers (listed in supplementary Table 6). The protocol of WISH followed the instruction of previous literatures^48^,^49^,^50^. Embryos were mounted in 95% glycerol (Sigma, cat:15523)/PBST (UniRegion Bio-Tech, cat:UR-PBS001 and Tween20) pH 5.5 or flat-mounted in 95% glycerol/PBST pH 5.5 or 2:1 benzyl benzoate (Sigma, cat:B6630)/benzyl alcohol (Alfa Aesar, cat:041218). Images were captured by Zeiss Axiovision Imager A1 or Zeiss Discovery V8.

### Tunel and acridine orange staining

Embryos were placed in 2 μg/ml acridine orange (Sigma, cat:A6014)/Egg water for 30 min, then washed 4 times with a 30 min interval. Embryos anaesthetized by tricaine (Sigma, cat:E10521) were mounted in 4% methylcellulose (Sigma, cat:M0387) with E3 egg water on top and captured by Zeiss Axiovision Imager A1 or Zeiss Discovery V8.

Embryos for Tunel assay (Millipore, cat:S7160) were dehydrated in 70% ethanol overnight after fixation. 10 min ethanol/acetic acid treatment at −20 degree was used to increase membrane permeability. After the pretreatment of equilibration buffer at room temperature for 10 min, embryos were placed in TdT labeling solution for 2 hours and then washed by phosphate buffered saline. Embryos were then mounted in 2% low-temperature agarose and captured by Nikon A1 confocal microscope.

The pixels of green fluorescence were counted and assayed by Image J. The statistic calculation of the pixels was carried out by Excel (Microsoft).

### γ-H2AX staining

Laid embryos were collected as mentioned above. The stage of embryos was staged as described^42^ and doubly checked by WT or WT-like control embryos (the siblings) that were collected in the same time windows. Embryos were dechorionated and sorted into different dishes of *mib*^*nn2002*^ homozygotes and WT siblings at 14 hpf. WT and *mib*^*nn2002*^ homozygotes that did not exposed to UV were moved to room temperate for 15 min. At the same time, the WT-UV control was placed in laminar flow hood with UV on for 15 min. Afterwards, all dishes were moved to 28.5 °C incubator for 1 hour before collected. Collected embryos were dehydrated by −20 °C methanol/acetone (1:1) and stored at −20 °C. Embryos were then sorted into 10 embryos/vial and washed by 1% Triton/PBS several times on shaker. After overnight blocking, embryos were washed by 2% BSA/1% Triton/PBS at 4 °C on shaker, and 1:1000 rabbit anti-human γ-H2AX (Cell signaling, #9718) in 2% BSA/1% Triton/PBS was used for overnight reaction. After intensive wash, 1:1000 goat anti-rabbit Alexa-488 antibody (Molecular probe) in 2% BSA/1% Triton/PBS was used for overnight reaction. After intensive wash, embryos were fixed by 4% paraformaldehyde. Embryos were further deyolked and flat-mounted in 75% glycerol to avoid strong background fluorescence from yolk. Images were taken by Zeiss Axiovision Imager A1.

### Genomic PCR and real-time RT-PCR

Genomic DNA was extracted by 50 mM NaOH and 1 M Tris-HCl pH 8.0. One PCR mix (GeneDireX, cat:MB203-0100) and 5x PCR Master Mix II (GeneMark, cat:RP02D) were used with primers (supplementary Table 6). For WISH samples, individual samples were washed repeatedly and sequentially by 1x PBST and double-distilled H_2_O to avoid glycerol contamination, cross contamination between samples and salt interference before extraction was carried out. PCR protocol followed the instruction of 5x PCR Master Mix II (GeneMark, cat:RP02D).

For the RT-PCR, embryos were collected and the total RNA was extracted using TRIzol (Invitrogen). Reverse transcription followed the instruction of SuperScript III FirstStrand (Invitrogen, cat:18080-051). After reverse transcription, PCRs were conducted using LightCycler 480 real-time PCR detection system using SYBR Green I Master (Roche). The data were normalized using the expression levels of β-actin mRNA. The occurrence of primer dimers and secondary products was inspected using melting curve analysis. Our data indicated that the amplification was specific. There was only one PCR product amplified for individual set of primers. Primer sequences were listed in supplementary Table 6.

### Genomic DNA extraction of F2 embryos and linkage analysis

F1 heterozygous parents (nn2002/WIK) were crossed to produce F2 embryos for genomic DNA extraction. *mib*^*nn2002*^ embryos were identified at 10 ss, lysed by 50 μl lysis buffer (10 mM Tris-HCl (pH 8.3), 1.0 mM EDTA, 12.5 mM KCl, 0.3% Tween 20, 0.3% NP-40), heated at 98 °C for 10 min, and incubated at 55 °C overnight with 1 mg/ml proteinase K. Proteinase K was then inactivated by incubation at 98 °C for 10 min. Afterwards, typical PCR assays were performed using selected simple-sequence length polymorphisms (SSLPs) markers. Since the mutant has the typical *mib* mutant phenotype, we selected seven SSLPs markers at chromosome 2, where the *mib* is located. PCR products were then undergone gel electrophoresis, and recombinant band was counted in each PCR reaction.

### RNA preparation and Illumina RNA-seq

Fish heterozygous for the *mib*^*nn2002*^ alleles were crossed to obtain *mib*^*nn2002*^ homozygotes. Total RNA from 15 pooled homozygous mutants and AB wild type at 16 ss was extracted using the mirVanaTM miRNA isolation kit (Applied Biosystems). RNA quality was assessed using the Agilent 2100 Bioanalyzer system and samples with a RNA Integrity Number (RIN) greater than 8 were used for RNA library construction.

Library construction was conducted according to the manufacturer’s protocol (Illumina, TruSeq Stranded mRNA Sample Prep). Briefly, six RNA (cDNA) libraries were constructed (three AB wild-type and three *mib*^*nn2002*^ homozygotes), and each prepared from 300 ng of total RNA. Illumina adaptors (Integrated DNA technology) were ligated to their 5’ and 3’ ends to construct strand-specific cDNA libraries. Sequencing was performed on the Illumina MiSeq sequencer to generate 2.0 Gb of sequencing data per sample. Sequences were extracted from image files using the Illumina pipeline set at default parameters. Low quality, homopolymers and adaptor sequences were removed, and the resulting filtered reads were then aligned with the zebrafish reference genome Zv9 (GCA_000002035.2) and undergone transcriptome analysis by CLC Genomics Workbench 6. Only selected data on chromosome 2 are shown in this study to prove the arm deletion. Data deposit and further bioinformatics analysis will be reported and released in the future (unpublished data).

### Bioinformatics analysis

The arm deletion was found after the rearrangement of the chromosome two. Individual reads of wild-type AB fish and *mib*^*nn2002*^ mutant samples (n = 3 for each) were listed (supplementary Table 3). The Database for Annotation, Visualization and Integrated Discovery (DAVID) was used for functional annotation clustering analysis on the missing genes in *mib*^*nn2002*^ mutants with the highest classification stringency as default^51^,^52^. 

#### Note

Similar to what we have demonstrated here, we noticed that *foxc1a* plays an important role in early somitogenesis shown by examining two TALEN-induced *foxc1a* null alleles^52^.

## Additional Information

**How to cite this article**: Hsu, C.-H. *et al.* A new *mib* allele with a chromosomal deletion covering *foxc1a* exhibits anterior somite specification defect. *Sci. Rep.* **5**, 10673; doi: 10.1038/srep10673 (2015).

## Supplementary Material

Supporting Information
