# Supplementary material for: A new mib allele with a chromosomal deletion covering foxc1a exhibits anterior somite specification defect
Source: Sci Rep. 2015 Jun 3;5:10673. doi: 10.1038/srep10673 (PMC4454137; doi:10.1038/srep10673)
Supplement: Supporting Information [file srep10673-s1.pdf]

Supplementary information:

**A new *mib* allele with a chromosomal deletion covering *foxc1a* exhibits anterior somite specification defect**

Chia-Hao Hsu[1,2], Ji-Sheng Lin[1], Keng Po Lai[3], Jing-Woei Li[4], Ting-Fung Chan[4], May-Su You[1], William Ka Fai Tse[5] and Yun-Jin Jiang*[,1,6,7]

[1]Institute of Molecular and Genomic Medicine, National Health Research Institutes, Taiwan
[2]Institute of Bioinformatics and Structural Biology, National Tsing Hua University, Taiwan
[3]School of Biological Sciences, The University of Hong Kong, Hong Kong
[4]School of Life Sciences, Hong Kong Bioinformatics Centre, The Chinese University of Hong Kong, Hong Kong
[5]Department of Biology, Hong Kong Baptist University, Hong Kong
[6]Biotechnology Center, National Chung Hsing University, Taiwan
[7]Institute of Molecular and Cellular Biology, National Taiwan University, Taiwan

Table S1. Percentage of embryos showing nn2002 homozygous phenotype from each clutch.

| Clutch no. | no. of embryos showing early segmentation phenotype | no. of embryos showing no segmentation phenotype | ratio of homozygotes |
|---|---|---|---|
| 1 | 21 | 78 | 21% |
| 2 | 16 | 64 | 20% |
| 3 | 14 | 58 | 19% |
| 4 | 19 | 65 | 23% |
| 5 | 44 | 151 | 23% |
| 6 | 11 | 23 | 32% |
| Total | 125 | 439 | 22% |

Table S2. Percentage of embryos showing transheterozygous (ta52b/nn2002) phenotypes from each clutch.

| Clutch no. | no. of transheterozygotes from $mib^{ta52b}$ ♀ x $mib^{nn2002}$ ♂ | no. of total embryos | ratio of transheterozygotes | no. of transheterozygotes from $mib^{nn2002}$ ♀ x $mib^{ta52b}$ ♂ | no. of total embryos | ratio of transheterozygotes |
|---|---|---|---|---|---|---|
| 1 | 45 | 158 | 28% | | | |
| 2 | 24 | 118 | 20% | | | |
| 3 | 5 | 21 | 24% | | | |
| 4 | 17 | 57 | 30% | | | |
| 5 | | | | 2 | 6 | 33% |
| 6 | 11 | 59 | 19% | | | |
| 7 | 29 | 102 | 28% | | | |
| 8 | 41 | 145 | 28% | | | |
| 9 | 12 | 35 | 34% | | | |
| 10 | | | | 51 | 198 | 26% |
| 11 | 23 | 82 | 28% | | | |
| 12 | 24 | 105 | 23% | | | |
| 13 | 27 | 115 | 23% | | | |
| 14 | 35 | 175 | 20% | | | |
| 15 | 36 | 179 | 20% | | | |
| 16 | 62 | 274 | 23% | | | |
| 17 | 31 | 143 | 22% | | | |
| 18 | 14 | 43 | 33% | | | |
| 19 | | | | 63 | 205 | 31% |
| 20 | | | | 11 | 37 | 30% |
| 21 | | | | 42 | 177 | 24% |
| 22 | | | | 66 | 268 | 25% |
| 23 | | | | 54 | 219 | 25% |
| Total | 436 | 1811 | 24% | 289 | 1110 | 26% |

Table S3. List of genes in LG2 and their relative expression in wild type embryos (AB) and *mib* mutants (2002).

| Feature ID | AB1 Expression values | AB 2 Expression values | AB3 Expression values | 2002-1 Expression values | 2002-2 Expression values | 2002-3 Expression values | Chromosome | Chromosome region start | Chromosome region end |
|---|---|---|---|---|---|---|---|---|---|
| ZNF554 (5 of 5) | 0 | 0.4499938 | 0 | 0 | 0 | 0 | 2 | 19233 | 21298 |
| CABZ01092286.1 | 3.6315475 | 10.712303 | 6.9913215 | 0 | 0 | 0 | 2 | 23916 | 32332 |
| CABZ01090951.1 | 2.5127515 | 2.0062799 | 1.0365979 | 0 | 0 | 0 | 2 | 50561 | 53808 |
| CABZ01084950.1 | 6.6380348 | 13.60351 | 12.459818 | 0 | 0 | 0 | 2 | 60855 | 62944 |
| CABZ01084952.1 | 0 | 0 | 0 | 0 | 0 | 0 | 2 | 73298 | 80311 |
| zgc:158345 | 18.337346 | 15.903438 | 23.73781 | 0 | 0 | 0 | 2 | 81941 | 87436 |
| cldn1 | 4.4232592 | 8.9273643 | 13.837681 | 0 | 0 | 0 | 2 | 88773 | 91785 |
| CPN2 | 9.2958934 | 14.239643 | 11.632473 | 0 | 0 | 0 | 2 | 93928 | 97276 |
| ANGPTL1 (2 of 2) | 0 | 0 | 0 | 0 | 0 | 0 | 2 | 108647 | 114343 |
| CABZ01085888.1 | 11.678024 | 8.702582 | 16.861577 | 0 | 0 | 0 | 2 | 115939 | 151915 |
| fggy | 7.3862678 | 6.6346734 | 7.6177345 | 0 | 0 | 1.4793259 | 2 | 154495 | 158192 |
| znfl2a | 69.331429 | 74.93296 | 57.75331 | 0 | 0 | 0 | 2 | 161051 | 164976 |
| igfbp1b | 0 | 0.3346374 | 0 | 0 | 0 | 0 | 2 | 167258 | 170770 |
| adcy1b | 0 | 0 | 0 | 0 | 0 | 0 | 2 | 179198 | 199691 |
| MOV10L1 | 0.3377878 | 0.786634 | 0.975445 | 0 | 0 | 0 | 2 | 200138 | 211977 |
| zgc:171570 | 3.0876964 | 2.4927312 | 3.8638077 | 0 | 0 | 0 | 2 | 221432 | 246482 |
| zgc:113293 | 0 | 0 | 0 | 0 | 0 | 0 | 2 | 250271 | 255471 |
| DHX30 | 1.9104279 | 1.7795878 | 2.2067313 | 0 | 0 | 0 | 2 | 256480 | 277540 |
| CABZ01064861.1 | 0 | 0.3278235 | 0 | 0 | 0 | 0 | 2 | 282825 | 287502 |
| PHLPP1 | 0.8673863 | 0.5386542 | 1.6698603 | 0 | 0 | 0 | 2 | 290248 | 301002 |
| wu:fi19a06 | 0 | 0.4998397 | 0 | 0 | 0 | 0 | 2 | 304364 | 314202 |
| CU928013.2 | 2.7183719 | 2.6728755 | 3.7069276 | 0 | 0 | 0 | 2 | 319490 | 340465 |
| zgc:113452 | 5.7539346 | 5.1781727 | 4.5060053 | 0 | 0 | 0 | 2 | 334714 | 346967 |
| CU693494.2 | 0 | 0 | 0 | 0 | 0 | 0 | 2 | 372564 | 373196 |
| SHARPIN (2 of 2) | 0 | 0.4013795 | 0.6221501 | 0 | 0 | 0 | 2 | 377851 | 385099 |
| CU693494.3 | 0.1015127 | 0.9456036 | 1.0259996 | 0 | 0 | 0 | 2 | 387136 | 392836 |
| wrnip1 | 1.6606899 | 1.1602152 | 1.4986402 | 0 | 0 | 0 | 2 | 394830 | 410278 |
| MYLK4 (1 of 2) | 1.2608858 | 0.4698124 | 0.7282233 | 0 | 0 | 0 | 2 | 427011 | 447851 |
| CABZ01064012.1 | 0 | 0 | 0 | 0 | 0 | 0 | 2 | 689562 | 790847 |
| foxc1a | 16.336109 | 14.216155 | 18.000801 | 0 | 0 | 0 | 2 | 868219 | 870389 |
| CU856140.1 | 0.8288701 | 0 | 0 | 0 | 0 | 0 | 2 | 878904 | 888438 |
| foxf2a | 1.8110757 | 2.53056 | 1.7433082 | 0 | 0 | 0 | 2 | 895049 | 897768 |
| foxq1a | 2.0006596 | 3.2613695 | 2.8886972 | 0 | 0 | 0 | 2 | 902565 | 903497 |
| irf4a | 0.2857648 | 0 | 0.4126078 | 0 | 0 | 0 | 2 | 1038215 | 1063571 |
| dusp22b | 0.3251943 | 0.9087679 | 0.4695391 | 0 | 0 | 0 | 2 | 1065725 | 1082670 |
| ripk1l | 4.2831927 | 3.1918786 | 3.0921919 | 0 | 0 | 0 | 2 | 1088190 | 1104351 |
| si:ch211-241e1.5 | 1.9021217 | 1.476542 | 1.6783684 | 0 | 0 | 0 | 2 | 1119723 | 1128678 |
| si:ch211-241e1.3 | 0 | 0 | 0 | 0 | 0 | 0 | 2 | 1128112 | 1169253 |
| si:ch211-241e1.4 | 0 | 0 | 0 | 0 | 0 | 0 | 2 | 1131839 | 1134018 |
| si:ch211-241e1.2 | 0.1655388 | 0 | 0 | 0 | 0 | 0 | 2 | 1185863 | 1268105 |
| polr2d | 149.96869 | 121.26047 | 129.70156 | 0 | 0 | 0 | 2 | 1308283 | 1313492 |
| CABZ01084564.1 | 0 | 0 | 0 | 0 | 0 | 0 | 2 | 1358788 | 1372630 |
| OSBPL1A (2 of 2) | 0.3535256 | 0 | 0.5104459 | 0 | 0 | 0 | 2 | 1386360 | 1401154 |
| ABCF3 | 24.004828 | 21.913585 | 24.261936 | 0 | 0 | 0 | 2 | 1402471 | 1409711 |
| prkacbb (2 1411669 | 6.349032 | 6.093422 | 11.528438 | 0 | 0 | 0 | 2 | 1411670 | 1439425 |
| CU467642.1 | 5.8150012 | 7.3126088 | 9.2357318 | 0 | 0 | 0 | 2 | 1522259 | 1622366 |
| LPHN2 (1 of 4) | 8.5346722 | 6.6678721 | 9.9378851 | 0 | 0 | 0 | 2 | 1626935 | 1688776 |
| FP102463.1 | 3.5219158 | 2.4605319 | 2.1188321 | 0 | 0 | 0 | 2 | 1691997 | 1695667 |
| exosc4 | 29.205547 | 40.808005 | 26.584857 | 0 | 0 | 0 | 2 | 1709545 | 1713492 |
| FP245444.1 | 6.8854209 | 12.413916 | 11.545164 | 0 | 0 | 0 | 2 | 1727339 | 1743445 |
| BCL10 | 1.0477774 | 0.9760179 | 1.1346426 | 0 | 0 | 0 | 2 | 1747679 | 1756376 |
| MCOLN3 (1 of 2) | 1.4349749 | 2.4060552 | 3.7294573 | 0 | 0 | 0 | 2 | 1760876 | 1787181 |
| ssx2ip | 3.8780098 | 1.3761582 | 1.5998147 | 0 | 0 | 0 | 2 | 1792049 | 1810559 |
| zgc:112335 | 0.5858805 | 2.7287757 | 3.8067153 | 0 | 0 | 0 | 2 | 1812389 | 1822105 |
| SLC22A23 (3 of 3) | 2.4206828 | 3.5702531 | 4.6602094 | 0 | 0 | 0 | 2 | 1823446 | 1897681 |
| 5S_rRNA (2 182444 | 0 | 0 | 0 | 0 | 0 | 0 | 2 | 1824450 | 1824564 |
| FP243387.1 | 0 | 0 | 0 | 0 | 0 | 0 | 2 | 1856129 | 1856224 |
| dspa | 19.497449 | 23.782491 | 26.538543 | 0 | 0 | 0 | 2 | 1912379 | 1964899 |
| hig1 | 10.291989 | 15.432922 | 17.759893 | 0 | 0 | 0 | 2 | 1970655 | 1975857 |
| si:ch211-163f10.4 | 0 | 0 | 0 | 0 | 0 | 0 | 2 | 1973479 | 1974747 |
| CCDC13 | 0.6076222 | 0.5660078 | 0.8773289 | 0 | 0 | 0 | 2 | 1983827 | 2009351 |

| | | | | | | | | | |
|---|---|---|---|---|---|---|---|---|---|
| tmie | 0 | 0 | 0 | 0 | 0 | 0 | 2 | 2025945 | 2081419 |
| pth1ra | 0 | 0.6168789 | 0 | 0 | 0 | 0 | 2 | 2092256 | 2240623 |
| mir460 | 0 | 0 | 0 | 0 | 0 | 0 | 2 | 2103466 | 2103542 |
| ss18 | 48.369846 | 54.92678 | 48.555349 | 0 | 0 | 0 | 2 | 2368246 | 2384584 |
| FP236168.1 | 2.1371128 | 4.5502803 | 6.8326618 | 0 | 0 | 0 | 2 | 2392229 | 2424395 |
| FP102801.1 (2 2428 | 0.1011606 | 0 | 0 | 0 | 0 | 0 | 2 | 2428046 | 2446159 |
| rpe65a | 0.8027302 | 0 | 0.7726934 | 0 | 0 | 0 | 2 | 2448547 | 2463377 |
| FP102801.1 (2 2472 | 3.7877748 | 8.7032883 | 6.1982719 | 0 | 0 | 0 | 2 | 2472964 | 2488188 |
| si:ch211-188c16.1 | 0 | 0.1610574 | 0.2496438 | 0 | 0.1566048 | 0 | 2 | 2490000 | 2505953 |
| c8a | 9.1500755 | 9.7410413 | 11.638739 | 0 | 0 | 0 | 2 | 2506994 | 2523112 |
| c8b | 9.3984576 | 10.467674 | 11.505147 | 0 | 0 | 0 | 2 | 2519755 | 2533944 |
| dab1b | 0 | 0.504871 | 0.2608551 | 0 | 0 | 0 | 2 | 2542265 | 2589205 |
| si:ch211-188c16.4 | 0 | 0 | 0 | 0 | 0 | 0 | 2 | 2543103 | 2547948 |
| prkacbb (2 2614676. | 1.007496 | 2.6448504 | 2.6449013 | 0 | 0 | 0 | 2 | 2614677 | 2642068 |
| zgc:66286 | 197.97436 | 178.77025 | 182.30211 | 0 | 0 | 0 | 2 | 2654111 | 2659244 |
| CRHR2 | 0 | 0 | 0 | 0 | 0 | 0 | 2 | 2681130 | 2785846 |
| aqp1a.1 | 1.9840725 | 1.4785509 | 2.291798 | 0 | 0 | 0 | 2 | 2888643 | 2900295 |
| aqp1a.2 | 0 | 0 | 0 | 0 | 0 | 0 | 2 | 2912958 | 2917596 |
| thoc1 | 61.949203 | 64.445908 | 62.67801 | 0 | 0 | 0 | 2 | 2923616 | 2941632 |
| usp14 | 56.795291 | 63.368194 | 61.503865 | 0 | 0 | 0.297108 | 2 | 2941647 | 2964335 |
| rock1 | 13.554978 | 13.430877 | 14.585248 | 0 | 0 | 0 | 2 | 2970496 | 3052905 |
| eif2b5 | 25.673513 | 26.494293 | 27.983669 | 0 | 0 | 0 | 2 | 3055167 | 3068257 |
| wacb | 4.9512345 | 2.3060688 | 2.6808565 | 0 | 0 | 0 | 2 | 3070270 | 3086434 |
| bambib | 3.3529651 | 3.6745052 | 5.6955927 | 0 | 0 | 0 | 2 | 3091038 | 3098818 |
| adipoqb | 0 | 0 | 0 | 0 | 0 | 0 | 2 | 3178319 | 3185111 |
| tmtopsa | 0 | 0 | 0 | 0 | 0 | 0 | 2 | 3255205 | 3340664 |
| CABZ01063645.2 | 0 | 0 | 0 | 0 | 0 | 0 | 2 | 3258302 | 3258383 |
| CABZ01063645.1 | 0 | 0 | 0 | 0 | 0 | 0 | 2 | 3260712 | 3260802 |
| CABZ01088149.2 | 0 | 1.8341518 | 0 | 0 | 0 | 0 | 2 | 3385910 | 3386762 |
| CABZ01088149.1 | 2.7482559 | 2.0480281 | 4.761757 | 0 | 0 | 0 | 2 | 3388619 | 3392143 |
| SERBP1 (2 of 3) | 73.090362 | 72.972722 | 81.179351 | 0 | 0 | 0 | 2 | 3397407 | 3409194 |
| IPP (1 of 2) | 1.2855478 | 2.3950081 | 0.9280835 | 0 | 0 | 0 | 2 | 3413431 | 3421555 |
| guk1a | 13.009744 | 11.064937 | 13.884129 | 0 | 0 | 0 | 2 | 3425900 | 3444870 |
| C2H1orf35 | 7.518766 | 6.2425404 | 6.1360784 | 0 | 0 | 0.4583061 | 2 | 3444090 | 3461887 |
| arf1 | 64.72169 | 55.83353 | 67.983156 | 0 | 0 | 0 | 2 | 3463796 | 3476115 |
| LRRC52 (2 of 2) | 0 | 0 | 0 | 0 | 0 | 0 | 2 | 3497699 | 3505613 |
| wu:fi26e04 | 0 | 0 | 0 | 0 | 0 | 0 | 2 | 3508171 | 3530580 |
| pik3r3a | 9.1626098 | 10.321501 | 10.460645 | 0 | 0 | 0 | 2 | 3546016 | 3567452 |
| ipp | 0 | 0.9501508 | 1.472762 | 0 | 0 | 0 | 2 | 3580312 | 3589765 |
| wnt3a | 1.2750105 | 0.3958961 | 2.4546033 | 0 | 0 | 0 | 2 | 3599278 | 3652698 |
| wnt9a | 0.4040293 | 0.7527168 | 1.7501003 | 0 | 0 | 0 | 2 | 3695124 | 3764786 |
| 5S_rRNA (2 375137 | 0 | 0 | 0 | 0 | 0 | 0 | 2 | 3751376 | 3751490 |
| snap47 | 0.437488 | 1.1546559 | 0.9475152 | 0 | 0 | 0 | 2 | 3879432 | 3898088 |
| jmjd4 | 2.6275555 | 1.4685607 | 1.8969274 | 0 | 0 | 0 | 2 | 3897559 | 3908363 |
| iba57 | 7.6047294 | 5.4739241 | 4.4919241 | 0 | 0 | 0 | 2 | 3910978 | 3914185 |
| CU633991.1 | 0 | 0 | 0 | 0 | 0 | 0 | 2 | 3922257 | 3923551 |
| cyp8b1 | 0 | 0 | 0 | 0 | 0 | 0 | 2 | 3930186 | 3932157 |
| CU694226.2 | 0 | 0 | 0 | 0 | 0 | 0 | 2 | 3946885 | 3948851 |
| CU694226.1 | 0 | 0 | 0 | 0 | 0 | 0 | 2 | 3962396 | 3964308 |
| cx47.1 | 0 | 0 | 0 | 0 | 0 | 0 | 2 | 3967127 | 3992746 |
| CU694226.3 | 0 | 0 | 0 | 0 | 0 | 0 | 2 | 3990061 | 3990173 |
| SERP1 | 108.21668 | 102.62974 | 105.34575 | 0.561183 | 0 | 0 | 2 | 3996683 | 4003534 |
| FUZ | 1.0089813 | 0.9398789 | 1.4568402 | 0 | 0 | 0 | 2 | 4011338 | 4016439 |
| gpt2l | 13.245633 | 14.309711 | 13.353553 | 0 | 0 | 0 | 2 | 4027400 | 4067295 |
| c1ql3b (2 4089627..4 | 0 | 0.3039818 | 0 | 0 | 0 | 0 | 2 | 4089628 | 4092270 |
| pter | 1.2944628 | 3.3159734 | 0.4672598 | 0 | 0 | 0 | 2 | 4097884 | 4110658 |
| mmp16b | 0.1511674 | 0 | 0.2182665 | 0 | 0 | 0 | 2 | 4124602 | 4172324 |
| egfra | 0.3568859 | 0.6648875 | 1.0305955 | 0 | 0 | 0 | 2 | 4190333 | 4307017 |
| npc1 | 13.88407 | 19.095981 | 20.85408 | 0 | 0 | 0 | 2 | 4310671 | 4353828 |
| MPP7 (2 of 3) | 0 | 0 | 0 | 0 | 0 | 0 | 2 | 4358228 | 4380961 |
| phb2 | 43.882587 | 47.856217 | 37.089281 | 0 | 0 | 0 | 2 | 4408283 | 4429954 |
| PEX5L (2 of 2) | 0.2709953 | 0.2524355 | 0.3912826 | 0 | 0 | 0 | 2 | 4440346 | 4511292 |
| CABZ01087197.1 | 3.7332308 | 5.7959196 | 7.1870785 | 0 | 0 | 0 | 2 | 4530979 | 4541827 |
| MFN1 (4 of 4) | 0 | 0 | 0 | 0 | 0 | 0 | 2 | 4552774 | 4574576 |
| tnk2b | 0.110294 | 0.2054805 | 0.3185009 | 0 | 0 | 0 | 2 | 4622092 | 4686262 |

| | | | | | | | | | |
|---|---|---|---|---|---|---|---|---|---|
| tfr1a | 3.3503353 | 4.4583997 | 4.3767465 | 0 | 0 | 0 | 2 | 4738019 | 4763716 |
| dlg1l | 2.9761087 | 1.9658006 | 3.7502149 | 0 | 0 | 0 | 2 | 4788963 | 4963219 |
| CABZ01055591.1 | 0 | 0 | 0 | 0 | 0 | 0 | 2 | 4989926 | 4999272 |
| FAM132B | 1.8985104 | 0.7073946 | 1.644724 | 0 | 0 | 0 | 2 | 5005145 | 5032086 |
| si:ch1073-184j22.2 | 0.3697733 | 0.4592646 | 0.1779685 | 0 | 0 | 0 | 2 | 5038156 | 5046905 |
| sft2d3 | 15.062694 | 12.951776 | 10.874304 | 0 | 0 | 0 | 2 | 5048645 | 5050255 |
| wdr33 | 29.743353 | 26.312797 | 27.571588 | 0 | 0 | 0 | 2 | 5052822 | 5086135 |
| DUSP28 | 1.7879458 | 0 | 0 | 0 | 0 | 0 | 2 | 5089269 | 5092304 |
| proc | 0.7346566 | 1.9161573 | 1.9093506 | 0 | 0 | 0 | 2 | 5098931 | 5118311 |
| PROC (2 of 2) | 1.2944628 | 0 | 0 | 0 | 0 | 0 | 2 | 5110275 | 5118311 |
| saga | 0 | 0 | 0 | 0 | 0 | 0 | 2 | 5122515 | 5147484 |
| cldn15la | 0 | 0.4659099 | 0 | 0 | 0 | 0 | 2 | 5150932 | 5159828 |
| crfb16 | 0.6436605 | 0 | 0 | 0 | 0 | 0 | 2 | 5164061 | 5179317 |
| parla | 1.3847295 | 0.4837099 | 0.7497648 | 0 | 0 | 0 | 2 | 5186291 | 5203409 |
| mb21d2a | 2.9391039 | 2.2962298 | 1.9165056 | 0 | 0 | 0 | 2 | 5205467 | 5224162 |
| fgf12a | 0 | 0.1312878 | 0 | 0 | 0 | 0 | 2 | 5229885 | 5324048 |
| cwc25 | 25.537072 | 18.177703 | 17.04473 | 0 | 0 | 0 | 2 | 5351814 | 5366176 |
| sst2 | 1.7315542 | 5.6453762 | 2.5001433 | 0 | 0 | 0 | 2 | 5368743 | 5371233 |
| NYAP2 (2 of 2) | 0 | 0 | 0.1398482 | 0 | 0 | 0 | 2 | 5377260 | 5418420 |
| slc35d1b | 9.0697613 | 3.2704248 | 6.33657 | 0 | 0 | 0 | 2 | 5436277 | 5452716 |
| mier1b | 6.319529 | 4.7324622 | 8.2300255 | 0 | 0 | 0 | 2 | 5453872 | 5478506 |
| dis3l2 | 4.6721773 | 4.6523435 | 5.350298 | 0 | 0 | 0 | 2 | 5483498 | 5527479 |
| ACAP2 (2 of 2) | 0.5247724 | 0.3666241 | 1.3259827 | 0 | 0 | 0 | 2 | 5530016 | 5566691 |
| si:ch211-168b3.2 | 2.9566242 | 1.8360886 | 0 | 0 | 0 | 0 | 2 | 5569645 | 5572719 |
| tmem125b | 0 | 0 | 0 | 0 | 0 | 0 | 2 | 5578811 | 5584297 |
| slc1a7a | 0.4652581 | 0.4333938 | 0.3358866 | 0 | 0 | 0 | 2 | 5590924 | 5642026 |
| scp2a | 10.3311 | 9.4419743 | 5.6289775 | 0 | 0 | 0 | 2 | 5647589 | 5682147 |
| C2H1orf123 | 55.379514 | 67.897226 | 54.091232 | 0 | 0 | 0 | 2 | 5684429 | 5694226 |
| uck2b | 74.531433 | 65.132521 | 66.935093 | 0 | 0.2319848 | 0 | 2 | 5694306 | 5707806 |
| aldh9a1b | 15.621889 | 12.126656 | 15.904881 | 0 | 0 | 0 | 2 | 5709761 | 5734452 |
| zte38 | 0 | 0 | 0 | 0 | 0 | 0 | 2 | 5734448 | 5746425 |
| prdx1 | 31.429103 | 25.457919 | 21.210037 | 0 | 0 | 0 | 2 | 5748881 | 5758078 |
| CU467854.1 | 1.7899052 | 2.1436963 | 1.476797 | 0 | 0 | 0 | 2 | 5769983 | 5791691 |
| CU467854.2 | 0 | 0 | 0 | 0 | 0 | 0 | 2 | 5794512 | 5823951 |
| si:ch73-344o19.1 | 0 | 0.2016206 | 0.6250358 | 0 | 0 | 0 | 2 | 5823773 | 5839652 |
| rec8a | 0 | 0 | 0 | 0 | 0 | 0 | 2 | 5854588 | 5874907 |
| gng5 | 151.18493 | 160.65882 | 133.96967 | 0 | 0 | 0 | 2 | 5885534 | 5891553 |
| zp3b | 1.0062617 | 1.6403546 | 1.0896851 | 0 | 1.3671469 | 0 | 2 | 5895636 | 5901186 |
| arl14 | 0 | 1.2673294 | 0.6547994 | 0 | 0 | 0 | 2 | 5903803 | 5904831 |
| ppm1la | 8.2685067 | 8.0231445 | 8.9540015 | 0 | 0 | 0 | 2 | 5908158 | 5934900 |
| otol1a | 0 | 0 | 0 | 0 | 0 | 0 | 2 | 5946029 | 5958557 |
| SMC6 | 3.4996305 | 3.8711907 | 5.5267404 | 0 | 0 | 0 | 2 | 5964013 | 6016219 |
| 5S_rRNA (2 601009 | 0 | 0 | 0 | 0 | 0 | 0 | 2 | 6010092 | 6010206 |
| rgs13 | 0 | 0 | 0 | 0 | 0 | 0 | 2 | 6179990 | 6186663 |
| rgs1 | 0 | 0 | 0 | 0 | 0 | 0 | 2 | 6216200 | 6223208 |
| rgs18 | 0 | 0 | 0 | 0 | 0 | 0 | 2 | 6240288 | 6253261 |
| FAM5C (2 of 3) | 0.2022774 | 0 | 0 | 0 | 0 | 0 | 2 | 6439225 | 6509894 |
| 5S_rRNA (2 647081 | 0 | 0 | 0 | 0 | 0 | 0 | 2 | 6470817 | 6470931 |
| si:zfos-986b4.1 | 0 | 0 | 0 | 0 | 0 | 0 | 2 | 6580575 | 6624140 |
| rgs5b | 0 | 0 | 0 | 0 | 0 | 0 | 2 | 6590676 | 6593943 |
| nos1apb | 0 | 0.2009681 | 0 | 0 | 0 | 0 | 2 | 6630924 | 6657578 |
| si:ch211-170d12.2 | 0 | 0 | 0 | 0 | 0 | 0 | 2 | 6663337 | 6663958 |
| ddr2b | 0 | 0 | 0 | 0 | 0 | 0 | 2 | 6668120 | 6701310 |
| XPR1 (1 of 2) | 8.7595091 | 13.636581 | 10.048789 | 0 | 0 | 0 | 2 | 6704193 | 6762154 |
| cdc14aa | 1.0928662 | 1.3573582 | 0.7889796 | 0 | 0 | 0 | 2 | 6780044 | 6810356 |
| si:dkey-60b12.6 | 0 | 0 | 0 | 0 | 0 | 0 | 2 | 6785126 | 6785749 |
| VCAM1 (2 of 2) | 0 | 0 | 0 | 0 | 0 | 0 | 2 | 6811661 | 6818735 |
| extl2 | 2.4971443 | 2.6168868 | 2.7041683 | 0 | 0 | 0 | 2 | 6828061 | 6836515 |
| zgc:110366 | 5.8186265 | 5.6911305 | 2.1003386 | 0 | 0 | 0 | 2 | 6837105 | 6853273 |
| si:dkey-60b12.9 | 3.7297205 | 2.6568038 | 2.8510096 | 0 | 0 | 0 | 2 | 6854412 | 6856538 |
| RC3H1 (1 of 2) | 3.4898161 | 3.5555711 | 6.7709536 | 0 | 0 | 0 | 2 | 6859433 | 6894418 |
| si:ch211-13f8.1 | 0 | 0 | 0.1654281 | 0 | 0 | 0 | 2 | 6897790 | 6914529 |
| COLGALT2 (2 of 2 | 1.0387398 | 0.2418998 | 0.7499039 | 0 | 0 | 0 | 2 | 6918043 | 6939984 |
| zgc:153115 | 12.403483 | 14.898581 | 17.437756 | 0 | 0 | 0 | 2 | 6943373 | 6951681 |
| si:dkeyp-106c3.3 | 0 | 0 | 0 | 0 | 0 | 0 | 2 | 6984470 | 6991133 |

| | | | | | | | | | |
|---|---|---|---|---|---|---|---|---|---|
| si:dkeyp-106c3.2 | 0 | 0.8616332 | 2.0033358 | 1.5901112 | 0.8378122 | 0 | 2 | 6992921 | 6994227 |
| si:dkeyp-106c3.1 | 0 | 0.197738 | 0 | 0 | 0 | 0 | 2 | 7000339 | 7117405 |
| 5S_rRNA (2 709961 | 0 | 0 | 0 | 0 | 0 | 0 | 2 | 7099614 | 7099732 |
| CABZ01088691.2 | 0 | 0 | 0 | 0 | 0 | 0 | 2 | 7151503 | 7153771 |
| CABZ01088691.1 | 0 | 0 | 0 | 0 | 0 | 0 | 2 | 7155344 | 7158267 |
| ripk2 | 1.2510827 | 0.3329713 | 1.2902884 | 0 | 0 | 0 | 2 | 7264635 | 7298101 |
| CCDC39 | 0.3281673 | 1.3756138 | 1.421495 | 0 | 0 | 0 | 2 | 7362320 | 7480705 |
| dcun1d1 | 10.912521 | 8.8276314 | 9.9513395 | 0 | 0 | 0 | 2 | 7482658 | 7495864 |
| eif4a2 | 17.875086 | 17.536554 | 21.141696 | 0 | 0 | 0 | 2 | 7500004 | 7511589 |
| SNORD2 (2 750489 | 0 | 0 | 0 | 0 | 0 | 0 | 2 | 7504900 | 7504970 |
| SNORD2 (2 750527 | 0 | 0 | 0 | 0 | 0 | 0 | 2 | 7505280 | 7505350 |
| SNORA81 (2 75055 | 0 | 0 | 0 | 0 | 0 | 0 | 2 | 7505553 | 7505731 |
| SNORA81 (2 75077 | 0 | 0 | 0 | 0 | 0 | 0 | 2 | 7507735 | 7507924 |
| SNORD2 (2 750821 | 0 | 0 | 0 | 0 | 0 | 0 | 2 | 7508211 | 7508282 |
| b3gnt5b | 5.6720357 | 4.082761 | 1.8612945 | 0 | 0 | 0.3614541 | 2 | 7533735 | 7541614 |
| tbl1xr1b | 1.8659934 | 3.476393 | 4.4904273 | 0 | 0 | 0 | 2 | 7549012 | 7585043 |
| kcnmb2 | 0 | 0 | 0 | 0 | 0 | 0 | 2 | 7602709 | 7613741 |
| KCNMB3 | 0 | 0 | 0 | 0 | 0 | 0 | 2 | 7616255 | 7629451 |
| si:ch211-38m6.6 | 0 | 0 | 0 | 0 | 0 | 0 | 2 | 7631821 | 7658416 |
| ephb3a | 9.948859 | 7.511543 | 8.0140926 | 0 | 0 | 0 | 2 | 7708305 | 7801920 |
| pcolce2a | 0.5622336 | 1.3093192 | 1.2176903 | 0 | 0 | 0 | 2 | 7815722 | 7831598 |
| pls1 | 0 | 0.2670931 | 1.6560089 | 0 | 0 | 0 | 2 | 7845116 | 7895670 |
| chst2a | 14.607608 | 12.136126 | 11.400823 | 0 | 0 | 0 | 2 | 7896790 | 7899153 |
| 5S_rRNA (2 797362 | 0 | 0 | 0 | 0 | 0 | 0 | 2 | 7973626 | 7973741 |
| C2H3orf58 (2 of 2) | 2.5794936 | 4.1503444 | 4.7402214 | 0 | 0 | 0 | 2 | 8045450 | 8096540 |
| si:ch211-222m2.2 | 1.4447488 | 1.0093513 | 0.5215082 | 1.6557505 | 1.635744 | 0 | 2 | 8268735 | 8272479 |
| si:ch211-222m2.1 | 0 | 0 | 0.2656895 | 0.2108862 | 0.5000113 | 0 | 2 | 8274929 | 8279057 |
| si:ch211-71m22.1 | 2.0164055 | 0.8049888 | 1.2477567 | 0 | 0 | 0 | 2 | 8371294 | 8396016 |
| si:ch211-71m22.5 | 0 | 0 | 0 | 0 | 0 | 0 | 2 | 8402160 | 8416146 |
| si:ch211-71m22.2 | 0 | 0 | 0 | 0 | 0 | 0 | 2 | 8418581 | 8419473 |
| si:ch211-71m22.3 | 0 | 0 | 0 | 0 | 0 | 0 | 2 | 8428763 | 8432781 |
| fubp1 | 99.016087 | 86.636676 | 94.622399 | 0 | 0 | 0.2674701 | 2 | 8433634 | 8447445 |
| SCARNA24 | 0 | 0 | 0 | 0 | 0 | 0 | 2 | 8441729 | 8441863 |
| fam73a | 13.59714 | 14.185817 | 14.135425 | 0 | 0 | 0 | 2 | 8447449 | 8473100 |
| usp33 | 4.4192968 | 6.7868779 | 7.0707277 | 0 | 0 | 0 | 2 | 8501496 | 8561837 |
| zzz3 | 11.126238 | 11.974209 | 12.477567 | 0 | 0 | 0 | 2 | 8563505 | 8625237 |
| ak5 | 0 | 0 | 0.2633015 | 0 | 0 | 0 | 2 | 8630181 | 8844296 |
| CR450828.1 | 0 | 0 | 0 | 0 | 0 | 0 | 2 | 8680562 | 8680640 |
| pigk | 2.204168 | 3.5197892 | 4.5464819 | 0 | 0 | 0 | 2 | 8846226 | 8922838 |
| st6galnac5a | 0 | 0.4390848 | 0.6805946 | 0 | 0 | 0 | 2 | 8945288 | 9044160 |
| st6galnac3 | 0.5467532 | 1.5279226 | 4.3419301 | 0 | 0 | 0 | 2 | 9114487 | 9273952 |
| 5S_rRNA (2 919089 | 0 | 0 | 0 | 0 | 0 | 0 | 2 | 9190899 | 9191016 |
| cope | 93.83458 | 89.754625 | 85.473859 | 0.7217374 | 0.5704133 | 0 | 2 | 9297672 | 9309055 |
| wu:fi34b01 | 0 | 0 | 0.5223168 | 0 | 0 | 0.5071565 | 2 | 9313963 | 9315252 |
| zgc:153615 | 1.0278719 | 0.7181068 | 1.1130869 | 0 | 0 | 0 | 2 | 9315715 | 9334070 |
| CABZ01069745.1 | 0 | 0 | 0 | 0 | 0 | 0 | 2 | 9338010 | 9342344 |
| txndc12 | 19.632686 | 25.321979 | 23.986002 | 0 | 0 | 0 | 2 | 9361512 | 9368564 |
| gipc2 | 9.5763685 | 9.1616033 | 7.1003792 | 0 | 0 | 0 | 2 | 9371736 | 9415099 |
| dnajb4 | 1.2014775 | 0.4476766 | 0.346956 | 0 | 0 | 0 | 2 | 9415509 | 9424697 |
| zgc:136678 | 0 | 0 | 0 | 0 | 0 | 0 | 2 | 9432998 | 9448262 |
| selt1a | 33.694727 | 30.712074 | 25.633262 | 0 | 0 | 0 | 2 | 9473203 | 9485168 |
| dvl3a | 22.177302 | 22.825406 | 23.064216 | 0 | 0 | 0 | 2 | 9490950 | 9532978 |
| si:ch1073-170o4.1 | 0 | 0 | 0.9306473 | 0 | 1.1676139 | 0 | 2 | 9535335 | 9536958 |
| SNORA13 (2 95357 | 0 | 0 | 0 | 0 | 0 | 0 | 2 | 9535755 | 9535890 |
| SNORA13 (2 95361 | 0 | 0 | 0 | 0 | 0 | 0 | 2 | 9536199 | 9536328 |
| pcyt1aa | 1.4151116 | 2.0160619 | 2.4038124 | 0 | 0 | 0.2334042 | 2 | 9544319 | 9572728 |
| ap2m1a | 107.01384 | 96.070221 | 90.231648 | 120.53646 | 119.68119 | 120.82532 | 2 | 9578708 | 9607608 |
| anxa13l | 0.6481303 | 0.8049888 | 0.9358175 | 1.2379801 | 0.9784172 | 0 | 2 | 9610654 | 9631137 |
| abi1b | 1.1249754 | 1.7028843 | 1.5228008 | 1.1281157 | 1.0826422 | 0.8871609 | 2 | 9651729 | 9704711 |
| 5S_rRNA (2 966442 | 0 | 0 | 0 | 0 | 0 | 0 | 2 | 9664426 | 9664542 |
| acbd5b | 0 | 0 | 0 | 0 | 0 | 0 | 2 | 9707575 | 9730294 |
| tsen15 | 2.0835762 | 1.6904418 | 1.6497777 | 1.6175941 | 1.6437071 | 3.2037858 | 2 | 9735018 | 9746702 |
| C2H1orf21 | 25.925214 | 26.936165 | 22.315648 | 17.426951 | 21.449916 | 17.474143 | 2 | 9746978 | 9760625 |
| sec22ba | 19.695576 | 17.857434 | 16.304395 | 18.05765 | 19.504475 | 20.617325 | 2 | 9759839 | 9772558 |
| imp3 | 52.191549 | 34.794583 | 56.887854 | 41.635226 | 44.028775 | 52.367246 | 2 | 9771984 | 9778860 |

| | | | | | | | | | |
|---|---|---|---|---|---|---|---|---|---|
| SLC35A3 (2 of 2) | 3.2263937 | 3.8250884 | 4.2350007 | 4.3698908 | 7.9700109 | 2.4672479 | 2 | 9779386 | 9829157 |
| FP102157.1 | 0 | 0.5066363 | 0.7853014 | 0 | 0 | 0 | 2 | 9845472 | 9850467 |
| cmpk | 56.090983 | 59.79062 | 68.46427 | 60.638014 | 59.44703 | 79.853584 | 2 | 9852644 | 9865826 |
| bcl6ab | 6.8069443 | 4.579434 | 7.3712693 | 7.1509904 | 5.4804051 | 5.3017172 | 2 | 9876231 | 9887088 |
| insl5b | 0 | 0 | 0 | 0 | 0 | 0 | 2 | 9916659 | 9917958 |
| AHSG | 0 | 0 | 0 | 0 | 0 | 0 | 2 | 9923242 | 9930638 |
| pfn2l | 159.68389 | 126.88198 | 154.66349 | 108.17394 | 111.5458 | 107.99577 | 2 | 9935926 | 9940736 |
| nsun4 | 6.6951772 | 5.301146 | 6.283538 | 5.371093 | 5.7610105 | 1.8772795 | 2 | 9949381 | 9958330 |
| dmbx1a | 5.2970594 | 2.8650646 | 5.9212474 | 4.1123967 | 4.3335536 | 5.9889409 | 2 | 9974227 | 9981384 |
| 5S_rRNA (2 999509 | 0 | 0 | 0 | 0 | 0 | 0 | 2 | 9995091 | 9995208 |
| gadd45aa | 2.6070047 | 0.6938451 | 2.1509613 | 45.669906 | 48.575721 | 49.602578 | 2 | 10071950 | 10075877 |
| gng12a | 13.391331 | 7.399946 | 7.2098003 | 7.80361 | 7.8121095 | 7.3187421 | 2 | 10078443 | 10130118 |
| wls | 70.772148 | 87.213482 | 85.687177 | 68.012632 | 66.773498 | 58.395106 | 2 | 10133495 | 10178043 |
| si:ch211-132a5.4 | 0 | 0.6201055 | 1.9223641 | 0.7629207 | 0 | 0 | 2 | 10177784 | 10207577 |
| scinlb | 5.2893124 | 4.6091865 | 4.6807984 | 3.5197562 | 3.2454117 | 1.4352437 | 2 | 10187884 | 10242089 |
| CCDC18 (2 of 2) | 1.7613489 | 1.3511802 | 2.6927609 | 2.4935514 | 2.1584266 | 2.1788363 | 2 | 10259668 | 10355322 |
| CCDC18 (1 of 2) | 0.4771512 | 1.3334171 | 0 | 0 | 0 | 0 | 2 | 10259795 | 10272567 |
| mtf2 | 10.152675 | 7.7378278 | 13.593046 | 8.2505889 | 9.6974771 | 10.351771 | 2 | 10398979 | 10423736 |
| fam69a | 0.4502932 | 0.1398179 | 0 | 0.6880764 | 0.4078574 | 0 | 2 | 10433352 | 10478531 |
| rpl5a | 2201.2228 | 2122.4278 | 1832.0046 | 1378.9692 | 1511.3015 | 1392.8402 | 2 | 10478581 | 10494926 |
| EVI5 (2 of 2) | 0 | 0 | 0 | 0 | 0 | 0 | 2 | 10501363 | 10526503 |
| gfi1aa | 8.606321 | 9.5015075 | 6.9035718 | 8.4020289 | 4.6194125 | 4.4687971 | 2 | 10558049 | 10576954 |
| rpap2 | 6.2022043 | 8.3195018 | 7.5223609 | 5.6864158 | 4.4941653 | 3.8259175 | 2 | 10582838 | 10612358 |
| glmna | 9.9697412 | 9.7757264 | 8.839056 | 9.6217224 | 8.3964921 | 11.525075 | 2 | 10612459 | 10649203 |
| cdc7 | 13.444488 | 8.2818089 | 12.523952 | 8.9465923 | 6.2851486 | 3.0401111 | 2 | 10655336 | 10669070 |
| tceanc2 | 11.608305 | 5.6229071 | 12.738292 | 7.9822001 | 4.2057338 | 8.4627012 | 2 | 10669711 | 10671898 |
| cdcp2 | 0.5529074 | 0.2575201 | 0.7983277 | 0 | 0.5008012 | 0.3875781 | 2 | 10672547 | 10698051 |
| mrpl37 | 44.310425 | 37.234742 | 41.161057 | 39.773193 | 39.853935 | 45.613778 | 2 | 10705328 | 10723075 |
| ssbp3a | 5.8955297 | 5.280539 | 7.5301934 | 10.134834 | 12.733687 | 13.033774 | 2 | 10726557 | 10818715 |
| 5S_rRNA (2 107979 | 0 | 0 | 0 | 0 | 0 | 0 | 2 | 10797962 | 10798095 |
| acot11a | 1.1503382 | 0.5357773 | 0.8304708 | 0.659171 | 0.6077925 | 0.4031832 | 2 | 10820443 | 10857700 |
| fam151a | 0 | 0 | 0 | 0.4753844 | 0.3757122 | 0 | 2 | 10860360 | 10874219 |
| cryz | 5.2828738 | 8.4751654 | 5.5089635 | 9.7543489 | 8.7725258 | 11.109598 | 2 | 10895509 | 10910704 |
| tyw3 | 10.035567 | 10.224657 | 11.320373 | 3.5941358 | 1.9883963 | 8.3537675 | 2 | 10910620 | 10917526 |
| lhx8a | 0 | 0 | 0 | 0 | 0 | 0 | 2 | 10997100 | 11001473 |
| slc44a5a | 0 | 0 | 0 | 0 | 0 | 0 | 2 | 11008119 | 11259816 |
| 5S_rRNA (2 110351 | 0 | 0 | 0 | 0 | 0 | 0 | 2 | 11035191 | 11035312 |
| si:dkey-189n19.4 | 0 | 0 | 2.5047904 | 0 | 6.2851486 | 4.8641777 | 2 | 11122029 | 11122621 |
| si:dkey-189n19.3 | 0 | 0 | 0 | 0 | 0 | 0 | 2 | 11128345 | 11129288 |
| sdr16c5a | 32.148017 | 40.033455 | 40.065748 | 50.416942 | 51.187043 | 55.982135 | 2 | 11274377 | 11295652 |
| penka | 0 | 0 | 0 | 0 | 0 | 0 | 2 | 11299996 | 11304124 |
| impad1 | 21.856658 | 26.705651 | 35.656697 | 38.061111 | 46.792625 | 42.580787 | 2 | 11424558 | 11440036 |
| MFF (1 of 2) | 1.647983 | 1.2792642 | 0.793159 | 0.6295555 | 0.7463383 | 0.3850688 | 2 | 11442481 | 11454228 |
| ftr01 | 0.2711527 | 0 | 0 | 0 | 0 | 0 | 2 | 11461586 | 11468724 |
| greb1l (2 11548228. | 5.1671014 | 11.732225 | 13.056112 | 9.9929411 | 14.040456 | 18.110226 | 2 | 11548229 | 11568413 |
| si:dkey-203o21.2 | 0 | 0 | 0 | 0 | 0 | 0 | 2 | 11552131 | 11558509 |
| greb1l (2 11555477. | 20.305225 | 20.669535 | 23.499861 | 27.409104 | 21.946742 | 19.075955 | 2 | 11555478 | 11624584 |
| abhd3 | 0 | 0 | 0 | 0 | 0 | 0 | 2 | 11646751 | 11703651 |
| trove2 | 3.1027517 | 3.4683029 | 3.1359842 | 3.3781053 | 2.66983 | 1.3049838 | 2 | 11714916 | 11737238 |
| cdc73 | 19.802173 | 22.329338 | 24.077315 | 37.624642 | 25.252014 | 28.8578 | 2 | 11737859 | 11789602 |
| CT573433.2 | 0 | 0 | 0 | 0 | 0 | 0 | 2 | 11768536 | 11769702 |
| KCNT2 (2 of 2) | 0 | 0 | 0.2501071 | 0 | 0 | 0 | 2 | 11810785 | 11853432 |
| KCNT2 (1 of 2) | 0 | 0 | 0 | 0 | 0 | 0 | 2 | 11862531 | 11868932 |
| GPR158 (1 of 2) | 0 | 0.1125859 | 0.1745114 | 0.554061 | 0.437893 | 0.6777849 | 2 | 11899842 | 12034000 |
| 5S_rRNA (2 120157 | 0 | 0 | 0 | 0 | 0 | 0 | 2 | 12015728 | 12015844 |
| prtfdc1 | 54.300677 | 40.200357 | 40.057555 | 32.610208 | 35.867344 | 28.921837 | 2 | 12062931 | 12087538 |
| arhgap21b | 16.261982 | 17.814333 | 20.474759 | 17.295138 | 17.145078 | 17.691802 | 2 | 12099800 | 12198630 |
| dpp6b | 0.7194047 | 0.1116891 | 0.1731214 | 0.137412 | 0.2172026 | 0 | 2 | 12198703 | 12424750 |
| kmt2cb | 3.8892322 | 4.0002512 | 5.9665272 | 8.2335206 | 6.3849114 | 4.3923429 | 2 | 12639649 | 12829533 |
| prkag2 | 0 | 0 | 0 | 0 | 0 | 0 | 2 | 12848791 | 12941196 |
| 5S_rRNA (2 129155 | 0 | 0 | 0 | 0 | 0 | 0 | 2 | 12915555 | 12915671 |
| BCL2 (2 of 2) | 0 | 0 | 0 | 0 | 0 | 0 | 2 | 12979720 | 13007658 |
| kdsr | 7.7288238 | 6.2265922 | 6.63534 | 6.4636526 | 8.5140693 | 5.5641925 | 2 | 13016280 | 13046785 |
| vps4b | 30.922845 | 23.895075 | 27.905402 | 32.015955 | 34.055992 | 32.760859 | 2 | 13047975 | 13060553 |
| si:dkey-185p13.1 | 0 | 0 | 0 | 0 | 1.3569061 | 0 | 2 | 13090040 | 13125921 |

| | | | | | | | | | |
|---|---|---|---|---|---|---|---|---|---|
| NMUR1 (2 of 2) | 0 | 0 | 0 | 0 | 0 | 0 | 2 | 13168221 | 13174684 |
| wdr65 | 1.4336524 | 0.8012792 | 0.8280044 | 0 | 0.3895634 | 0 | 2 | 13254294 | 13461565 |
| 5S_rRNA (2 13354( | 0 | 0 | 0 | 0 | 0 | 0 | 2 | 13354085 | 13354199 |
| LEPROT | 4.320869 | 4.0249442 | 14.557161 | 8.2532007 | 5.2182252 | 16.153874 | 2 | 13523455 | 13524358 |
| ebna1bp2 | 165.0095 | 172.00706 | 177.74405 | 183.60554 | 141.55107 | 160.34496 | 2 | 13526922 | 13550157 |
| ZBTB41 | 2.9387805 | 2.1671964 | 1.944811 | 2.6663187 | 2.1072812 | 2.5750403 | 2 | 13575574 | 13634443 |
| zgc:66475 | 14.634567 | 17.150293 | 12.269292 | 12.714187 | 13.041604 | 5.2947448 | 2 | 13740482 | 13773947 |
| pde4bb | 0 | 0 | 0 | 0 | 0.1942446 | 0 | 2 | 13915648 | 14057782 |
| si:dkey-228i19.34 | 0 | 0 | 0 | 0 | 0 | 0 | 2 | 13960500 | 13962401 |
| si:dkey-228i19.32 | 2.5044035 | 1.5552557 | 4.8213854 | 1.9134433 | 1.5122585 | 4.6814447 | 2 | 13984724 | 13986152 |
| tctex1d1 | 3.4312783 | 4.7944188 | 2.477164 | 1.9662037 | 0.5179856 | 0.8017548 | 2 | 14241524 | 14305449 |
| SGIP1 | 0.7268752 | 0.2256978 | 0.6996766 | 0.8330333 | 0.6583742 | 0.3396843 | 2 | 14312207 | 14432336 |
| BX510915.1 | 0 | 0 | 0 | 0 | 0 | 0 | 2 | 14318806 | 14321829 |
| BX510915.3 | 0 | 0 | 0 | 0 | 0 | 0 | 2 | 14478533 | 14480583 |
| U2 (2 14480938..14 | 0 | 0 | 0 | 0 | 0 | 0 | 2 | 14480939 | 14481129 |
| U2 (2 14485750..14 | 0 | 0 | 0 | 0 | 0 | 0 | 2 | 14485751 | 14485936 |
| U2 (2 14491579..14 | 0 | 0 | 0 | 0 | 0 | 0 | 2 | 14491580 | 14491756 |
| U2 (2 14496377..14 | 0 | 0 | 0 | 0 | 0 | 0 | 2 | 14496378 | 14496568 |
| U2 (2 14501190..14 | 0 | 0 | 0 | 0 | 0 | 0 | 2 | 14501191 | 14501376 |
| U2 (2 14529046..14 | 0 | 0 | 0 | 0 | 0 | 0 | 2 | 14529047 | 14529234 |
| U2 (2 14533976..14 | 0 | 0 | 0 | 0 | 0 | 0 | 2 | 14533977 | 14534121 |
| U2 (2 14545542..14 | 0 | 0 | 0 | 0 | 0 | 0 | 2 | 14545543 | 14545729 |
| U2 (2 14588059..14 | 0 | 0 | 0 | 0 | 0 | 0 | 2 | 14588060 | 14588209 |
| ZCCHC3 | 0 | 0 | 0 | 0 | 0 | 0 | 2 | 14588404 | 14588922 |
| si:dkey-48k17.29 | 0 | 0 | 0 | 0 | 0 | 0 | 2 | 14599687 | 14600658 |
| si:ch73-366i20.25 | 0 | 1.6219924 | 0 | 0 | 0 | 0 | 2 | 14609366 | 14609830 |
| si:ch73-366i20.1 (2 | 0 | 0 | 0 | 0 | 0 | 0 | 2 | 14611738 | 14618878 |
| si:dkey-10f21.16 | 0 | 0 | 0 | 0 | 0 | 0 | 2 | 14790590 | 14791602 |
| hccsal | 0 | 0 | 0 | 0 | 0 | 0 | 2 | 14802966 | 14807865 |
| si:dkey-10f21.3 | 0 | 0 | 0.4871935 | 0 | 0 | 0 | 2 | 14813462 | 14815633 |
| hccsa | 8.1732534 | 7.9760361 | 11.801135 | 6.6906681 | 9.8706712 | 12.004255 | 2 | 14819195 | 14853746 |
| si:dkey-10f21.4 | 0 | 0.1980383 | 0 | 0 | 0.1925632 | 0.2980555 | 2 | 14855673 | 14862429 |
| cnn3b | 47.234732 | 44.785461 | 41.712173 | 33.108276 | 33.997456 | 28.97186 | 2 | 14868993 | 14895878 |
| f3b | 2.4143215 | 2.5702526 | 0.995992 | 0.3952752 | 0 | 1.4506251 | 2 | 14921325 | 14927551 |
| si:dkey-61d19.4 | 0 | 0 | 0 | 0 | 0 | 0 | 2 | 14922686 | 14928299 |
| abcd3b | 4.2973565 | 7.0963017 | 9.5892896 | 6.2681487 | 7.0770405 | 6.5724427 | 2 | 14930111 | 14946095 |
| arhgap29b | 3.8809961 | 4.0168856 | 6.1225209 | 5.1891062 | 4.3615137 | 4.8364594 | 2 | 14949001 | 15018503 |
| 5S_rRNA (2 14993{ | 0 | 0 | 0 | 0 | 0 | 0 | 2 | 14993850 | 14993966 |
| abca4b | 0 | 0 | 0.1824749 | 0 | 0 | 0 | 2 | 15030259 | 15115218 |
| tecrl2 | 0 | 0 | 0 | 0 | 0 | 0 | 2 | 15120671 | 15145420 |
| olfm3b | 0 | 0 | 0 | 0 | 0 | 0 | 2 | 15164217 | 15169961 |
| col11a1b | 3.0754591 | 2.6309655 | 4.4405705 | 2.0140696 | 2.046583 | 1.2319094 | 2 | 15210524 | 15383469 |
| amy2a | 2.3130302 | 0.8079814 | 0.4174651 | 0 | 0 | 0.4053481 | 2 | 15433338 | 15447555 |
| vav3b (2 15610726.. | 0 | 0 | 0 | 0 | 0 | 0 | 2 | 15610727 | 15720770 |
| si:dkey-94a14.1 | 0 | 0.770734 | 0 | 0 | 0 | 0 | 2 | 15654136 | 15664619 |
| 5S_rRNA (2 15689( | 0 | 0 | 0 | 0 | 0 | 0 | 2 | 15689008 | 15689123 |
| 5S_rRNA (2 15706. | 0 | 0 | 0 | 0 | 0 | 0 | 2 | 15706358 | 15706472 |
| vav3b (2 15813971.. | 0 | 0.1254166 | 0.388799 | 0 | 0 | 0 | 2 | 15813972 | 15979786 |
| selj | 9.3437556 | 7.211742 | 3.4691633 | 2.4476311 | 1.6926394 | 2.2456473 | 2 | 15981489 | 15992851 |
| sh3glb1 | 4.6585066 | 7.7810969 | 8.3498761 | 8.468551 | 8.729974 | 7.6571031 | 2 | 15994629 | 16031206 |
| arhgef4 | 1.6997326 | 0.9715842 | 1.8406477 | 0.6198099 | 0.279918 | 0.1083165 | 2 | 16019126 | 16179625 |
| si:dkey-231j24.3 | 0 | 0 | 0 | 0 | 0 | 3.3266029 | 2 | 16186657 | 16197001 |
| atr | 3.0915196 | 2.8797897 | 3.5869499 | 1.5184405 | 2.2001366 | 1.4705319 | 2 | 16204868 | 16270087 |
| GEN1 | 10.591181 | 11.36064 | 14.82891 | 11.402359 | 9.8837636 | 8.0991569 | 2 | 16270210 | 16279055 |
| agfg1b | 4.8827757 | 7.6347595 | 5.0357893 | 5.9955987 | 5.5282767 | 6.8454758 | 2 | 16280904 | 16299874 |
| fbxo36b | 1.9283217 | 0 | 2.0881878 | 0.552487 | 0 | 0 | 2 | 16302921 | 16305991 |
| CR377211.1 | 0 | 0 | 0.7665399 | 0 | 0 | 0 | 2 | 16308194 | 16309072 |
| si:dkeyp-13a3.6 | 0 | 0.574992 | 0.4456274 | 0 | 0 | 0 | 2 | 16324266 | 16327378 |
| si:dkeyp-13a3.7 | 0 | 0 | 0 | 0 | 0 | 0 | 2 | 16329407 | 16333005 |
| si:dkeyp-13a3.8 | 0 | 0 | 0 | 0 | 0 | 0 | 2 | 16333444 | 16339963 |
| si:dkeyp-13a3.9 | 0 | 0 | 0 | 0 | 0 | 0 | 2 | 16347781 | 16349263 |
| grk7a | 0 | 0.1374309 | 0 | 0.1690823 | 0 | 0 | 2 | 16357036 | 16364710 |
| atp1b3a | 45.750377 | 43.856825 | 55.008054 | 82.747385 | 68.562456 | 75.102557 | 2 | 16368289 | 16386273 |
| si:dkeyp-13a3.10 | 5.0084174 | 5.2739352 | 5.9738608 | 8.7346053 | 5.1281299 | 3.3581665 | 2 | 16406132 | 16416179 |
| xrn1 | 5.6145303 | 8.3143687 | 10.497092 | 8.496863 | 8.7364826 | 6.6603895 | 2 | 16417594 | 16467843 |

| | | | | | | | | | |
|---|---|---|---|---|---|---|---|---|---|
| gk5 | 9.0152512 | 7.8284768 | 7.5012485 | 8.5807344 | 8.4424528 | 8.3546313 | 2 | 16472899 | 16515517 |
| ppp1r7 | 27.209541 | 26.870605 | 27.176042 | 26.259724 | 27.610153 | 22.945441 | 2 | 16517108 | 16528856 |
| zgc:158450 | 71.311328 | 82.514769 | 93.3018 | 105.47705 | 92.030664 | 90.291064 | 2 | 16534447 | 16574921 |
| SNORD66 (2 16543 | 0 | 0 | 0 | 0 | 0 | 0 | 2 | 16543809 | 16543884 |
| SNORD66 (2 16548 | 0 | 0 | 0 | 0 | 0 | 0 | 2 | 16548754 | 16548814 |
| tfa | 983.20151 | 1083.5453 | 893.58213 | 2024.1956 | 2017.8582 | 1751.7505 | 2 | 16581396 | 16592930 |
| ubxn7 | 16.144172 | 27.841549 | 18.900101 | 19.502093 | 25.688598 | 24.162842 | 2 | 16636583 | 16663153 |
| clcn2 | 0 | 0 | 0.1600828 | 0 | 0 | 0 | 2 | 16726157 | 16825889 |
| 5S_rRNA (2 167686 | 0 | 0 | 0 | 0 | 0 | 0 | 2 | 16768660 | 16768779 |
| fam131a | 0 | 0 | 0 | 0 | 0 | 0 | 2 | 16837577 | 16863572 |
| thpo | 0 | 0 | 0 | 0 | 0 | 0 | 2 | 16876364 | 16893854 |
| pif1 | 5.6786796 | 6.6899931 | 6.5111999 | 2.105541 | 3.0255994 | 2.8098722 | 2 | 16918145 | 16936147 |
| si:ch211-234e14.2 | 0 | 0 | 2.6527111 | 0 | 0 | 0 | 2 | 17003072 | 17007080 |
| artnb | 0 | 0 | 0 | 0 | 0 | 0 | 2 | 17021615 | 17022236 |
| st3gal3b | 1.5121641 | 2.8172001 | 2.9111627 | 2.4262164 | 1.3696573 | 1.5546664 | 2 | 17085994 | 17214190 |
| kdm4ab | 4.9298655 | 6.0483057 | 6.4236482 | 6.201065 | 5.3365462 | 4.7200446 | 2 | 17271981 | 17313945 |
| si:dkey-183g16.1 | 0 | 0 | 0 | 0 | 0 | 0 | 2 | 17328080 | 17330598 |
| si:dkey-183g16.2 | 0 | 0 | 0.2502929 | 0 | 0 | 0 | 2 | 17341146 | 17347082 |
| ptprfb | 17.303643 | 22.092576 | 25.508398 | 29.260823 | 24.879426 | 23.326043 | 2 | 17349918 | 17599781 |
| si:dkey-21k10.2 | 0.7253687 | 2.2523004 | 2.0946796 | 0.8313068 | 1.0950162 | 0.6779605 | 2 | 17382949 | 17385372 |
| 5S_rRNA (2 174294 | 0 | 0 | 0 | 0 | 0 | 0 | 2 | 17429460 | 17429578 |
| si:dkey-21k10.3 | 0 | 0 | 0 | 0 | 0 | 0 | 2 | 17478994 | 17486322 |
| dpydb | 1.3871993 | 3.1012649 | 2.4035266 | 5.8822455 | 5.5284645 | 5.2509695 | 2 | 17988789 | 18244676 |
| ptbp2b | 18.181319 | 20.410206 | 23.110299 | 19.590015 | 13.79363 | 14.596582 | 2 | 18274456 | 18292829 |
| 5S_rRNA (2 183758 | 0 | 0 | 0 | 0 | 0 | 0 | 2 | 18375814 | 18375928 |
| rwdd3 | 3.2227476 | 2.1014211 | 2.3266181 | 1.8467107 | 1.4595174 | 1.8072704 | 2 | 18420634 | 18429023 |
| si:dkey-1p9.2 (2 184 | 0 | 0 | 0 | 0 | 0 | 0 | 2 | 18434321 | 18436482 |
| BX088713.1 | 0 | 0 | 0 | 0 | 0 | 0 | 2 | 18434884 | 18434981 |
| si:dkey-236a14.10 (2 | 0 | 0 | 0 | 0 | 0 | 0 | 2 | 18439678 | 18441841 |
| si:dkey-1p9.4 | 0 | 0.5645376 | 0 | 0 | 0 | 0 | 2 | 18448331 | 18468279 |
| si:dkey-1p9.5 | 0 | 0 | 0 | 0 | 0 | 0 | 2 | 18453186 | 18455022 |
| si:dkey-236a14.4 | 0 | 0 | 0 | 0 | 0 | 0 | 2 | 18489887 | 18491896 |
| BX664750.2 | 0 | 0 | 0 | 0 | 0 | 0 | 2 | 18490296 | 18490393 |
| si:dkey-236a14.5 | 0 | 0 | 0 | 0 | 0 | 0 | 2 | 18493335 | 18495479 |
| si:dkey-236a14.6 | 3.7407122 | 0.2903767 | 2.7005556 | 1.4290111 | 0.5646977 | 0.4370287 | 2 | 18506556 | 18510928 |
| si:dkey-236a14.7 | 0 | 0.4724934 | 0 | 0 | 0 | 0 | 2 | 18510804 | 18530295 |
| si:dkey-236a14.10 (2 | 0 | 0 | 0 | 0 | 0 | 0 | 2 | 18545728 | 18547893 |
| BX664750.3 | 0 | 0 | 0 | 0 | 0 | 0 | 2 | 18546292 | 18546389 |
| si:dkey-1p9.2 (2 185 | 0 | 0 | 0 | 0 | 0 | 0 | 2 | 18549285 | 18551455 |
| BX664750.1 | 0 | 0 | 0 | 0 | 0 | 0 | 2 | 18550793 | 18550890 |
| si:dkey-236a14.12 | 0 | 0 | 0 | 0 | 0 | 0 | 2 | 18556679 | 18563664 |
| si:dkey-236a14.13 | 2.4641787 | 1.1477068 | 2.2237248 | 1.0590246 | 0.8369827 | 2.1591812 | 2 | 18561839 | 18564712 |
| mir137-1 | 0 | 0 | 0 | 0 | 0 | 0 | 2 | 18658781 | 18658865 |
| lppr5a | 0 | 0.3008263 | 0 | 0 | 0 | 0 | 2 | 18777292 | 18837198 |
| lppr4a | 0 | 0.3846849 | 0 | 0.3549607 | 0.2805373 | 0.4342247 | 2 | 18845341 | 18884569 |
| palmda | 0.3761821 | 1.2264642 | 2.1726356 | 2.1556123 | 1.3629222 | 0.7910906 | 2 | 18919836 | 18933712 |
| frrs1a (2 18957773.. | 0 | 0 | 0 | 0 | 0 | 0 | 2 | 18957774 | 18983437 |
| si:ch211-153l6.3 | 0 | 0.3219955 | 0.4991027 | 0 | 0.3130935 | 0.4846162 | 2 | 18966911 | 18976220 |
| si:ch211-153l6.4 | 0 | 2.420345 | 3.0012856 | 4.1688773 | 3.2948037 | 2.18563 | 2 | 18971244 | 18972306 |
| si:ch211-153l6.1 | 0 | 0 | 6.5735474 | 0 | 2.0618353 | 3.1913751 | 2 | 18985447 | 18986613 |
| zfyve9a | 5.2580715 | 5.1805348 | 5.839988 | 7.996037 | 6.5027114 | 4.961672 | 2 | 19131620 | 19171555 |
| cc2d1b | 1.8937593 | 2.6460911 | 2.2786223 | 1.6277533 | 1.7152908 | 1.3274912 | 2 | 19180207 | 19205166 |
| cdc20 | 53.380286 | 51.697603 | 60.558395 | 43.211856 | 39.332106 | 40.091378 | 2 | 19218016 | 19234798 |
| elovl1a | 8.4529733 | 7.4108721 | 6.1025074 | 7.9779474 | 9.0074852 | 8.3652454 | 2 | 19237376 | 19288419 |
| si:dkey-225f23.4 | 0 | 0.5269018 | 0 | 0 | 0 | 0 | 2 | 19270815 | 19299800 |
| KNCN | 0 | 0 | 0 | 1.4338 | 0 | 0 | 2 | 19323999 | 19332537 |
| glula | 193.86722 | 185.18443 | 163.49283 | 124.85393 | 122.95683 | 145.21783 | 2 | 19460034 | 19463977 |
| PBX1A | 0 | 0 | 0 | 0 | 0 | 0 | 2 | 19615653 | 19618709 |
| pbx1a | 1.7445004 | 0 | 0.629709 | 0.49982 | 0.7900491 | 0 | 2 | 19717559 | 19749261 |
| 5S_rRNA (2 198817 | 0 | 0 | 0 | 0 | 0 | 0 | 2 | 19881796 | 19881912 |
| 5S_rRNA (2 199502 | 0 | 0 | 0 | 0 | 0 | 0 | 2 | 19950258 | 19950374 |
| frrs1a (2 20213892.. | 0 | 0 | 0 | 0 | 0 | 0 | 2 | 20213893 | 20239553 |
| agla | 3.7372711 | 4.1399426 | 6.2711927 | 3.0097386 | 3.7510229 | 3.3986073 | 2 | 20242651 | 20295817 |
| 5S_rRNA (2 202556 | 0 | 0 | 0 | 0 | 0 | 0 | 2 | 20255652 | 20255766 |
| si:ch211-267e7.2 | 0 | 0 | 0 | 0 | 0 | 0 | 2 | 20308866 | 20325163 |

| | | | | | | | | | |
|---|---|---|---|---|---|---|---|---|---|
| si:ch211-267e7.3 | 22.338279 | 22.016619 | 16.647032 | 23.453567 | 23.496517 | 17.174092 | 2 | 20325257 | 20368685 |
| olfml2bb | 1.7492231 | 0.1810471 | 1.9643983 | 1.113718 | 1.2322923 | 2.4523478 | 2 | 20374342 | 20384758 |
| si:ch211-267e7.11 | 0 | 0 | 0 | 0 | 0 | 0 | 2 | 20401639 | 20402945 |
| dusp12 | 5.5283034 | 3.9379941 | 5.1649301 | 1.4907523 | 2.3563832 | 1.8236429 | 2 | 20430282 | 20436382 |
| si:ch211-267e7.22 | 0 | 0.9460152 | 0 | 0.5819449 | 0.4599306 | 0 | 2 | 20468899 | 20473941 |
| rxrga (2 20495130..2 | 7.374911 | 4.5009185 | 5.8749961 | 0.8743445 | 2.9944366 | 2.4957075 | 2 | 20495131 | 20546256 |
| rxrga (2 20495130..2 | 0 | 0 | 0 | 0 | 0 | 0 | 2 | 20495131 | 20495229 |
| 5S_rRNA (2 205271 | 0 | 0 | 0 | 0 | 0 | 0 | 2 | 20527154 | 20527271 |
| prg4a | 1.4006619 | 0 | 1.6853142 | 0.5350749 | 0.4228877 | 0.6545592 | 2 | 20566497 | 20584574 |
| tpra | 14.810575 | 17.695175 | 16.270876 | 25.091438 | 26.319297 | 19.522573 | 2 | 20584192 | 20662758 |
| C2H1orf27 | 3.6479255 | 7.0575689 | 8.1032906 | 4.1806953 | 8.387442 | 9.4417111 | 2 | 20662898 | 20675820 |
| pdca | 0 | 0 | 0 | 0 | 0 | 0 | 2 | 20678954 | 20686429 |
| ptgs2a | 12.978995 | 10.258265 | 12.493341 | 18.029748 | 17.277537 | 13.509213 | 2 | 20712497 | 20719864 |
| cpla2 | 5.4031708 | 5.033122 | 4.1607942 | 4.128193 | 3.099517 | 4.7975323 | 2 | 20721899 | 20753362 |
| arpc5a | 36.157857 | 28.36337 | 33.16932 | 36.45352 | 38.41392 | 42.878584 | 2 | 20763673 | 20771677 |
| lyrm4 | 26.414369 | 22.261955 | 20.885631 | 7.207647 | 15.380402 | 18.515997 | 2 | 20775956 | 20777907 |
| nrn1b | 0 | 0.5734749 | 0 | 0 | 0 | 0 | 2 | 20788259 | 20790126 |
| si:ch211-279l9.7 | 3.8205578 | 4.575726 | 8.6686254 | 4.3785402 | 2.9661491 | 3.060734 | 2 | 20796355 | 20829158 |
| rreb1b | 10.350152 | 11.24818 | 13.449869 | 14.155039 | 11.812185 | 9.9639044 | 2 | 20840352 | 20858154 |
| riok1 | 19.334507 | 20.678537 | 21.712881 | 9.3009984 | 10.810134 | 13.051173 | 2 | 20859660 | 20872570 |
| nebl | 0 | 0 | 0 | 0 | 0 | 0 | 2 | 20873367 | 20878695 |
| pip4k2ab | 0 | 0 | 0 | 0 | 0.2732232 | 0.4229036 | 2 | 20886317 | 20951844 |
| bmi1b | 11.347206 | 10.570066 | 8.1919588 | 7.663328 | 4.955388 | 4.5452498 | 2 | 20960930 | 20966517 |
| dnajc1 | 36.260961 | 40.686588 | 39.564629 | 39.903952 | 51.318308 | 50.258874 | 2 | 20970998 | 20978561 |
| si:dkey-29d8.3 | 2.9083243 | 0.4925711 | 1.1452498 | 0 | 0.4789532 | 0.7413393 | 2 | 20981903 | 21039018 |
| si:dkey-29d8.2 | 0 | 0 | 0 | 0 | 0 | 0 | 2 | 21002878 | 21004102 |
| vipr1a | 0.705448 | 0 | 0 | 0 | 0.3194832 | 0 | 2 | 21026266 | 21094983 |
| zbtb47a | 0 | 0.2137138 | 0 | 0 | 0 | 0 | 2 | 21105860 | 21116657 |
| klhl40a | 8.5951518 | 12.49794 | 12.712993 | 2.402549 | 3.0381042 | 1.7634283 | 2 | 21123259 | 21131131 |
| hhatla | 2.4918409 | 3.5872803 | 3.2708185 | 2.076922 | 1.6414611 | 1.2703532 | 2 | 21133813 | 21148101 |
| ctdsplb | 31.830226 | 39.180699 | 33.648396 | 33.221897 | 34.236261 | 40.640471 | 2 | 21152242 | 21177506 |
| dre-mir-26a-2 | 0 | 0 | 0 | 0 | 0 | 0 | 2 | 21171567 | 21171694 |
| plcd1b | 11.772163 | 9.3641558 | 8.2122762 | 6.5183442 | 6.2299219 | 5.1923167 | 2 | 21184002 | 21235078 |
| si:ch211-12e1.5 | 0.5379295 | 0.7516322 | 0 | 0 | 0 | 0 | 2 | 21228248 | 21231672 |
| afg3l2 | 10.167514 | 9.7977604 | 12.149457 | 15.067827 | 13.02008 | 15.729091 | 2 | 21249272 | 21265553 |
| inhbab | 0 | 0 | 0 | 0 | 0 | 0 | 2 | 21283052 | 21300265 |
| si:ch211-12e1.3 | 0 | 1.0325272 | 0 | 0 | 0 | 0 | 2 | 21337905 | 21339602 |
| mplkip | 19.060509 | 20.510208 | 18.505462 | 21.467621 | 14.585307 | 20.271974 | 2 | 21402070 | 21404307 |
| ralab | 7.4626509 | 5.7683106 | 8.25328 | 5.0951369 | 5.8964702 | 4.8972786 | 2 | 21404875 | 21415152 |
| C2H7orf25 | 1.0492498 | 0.4886947 | 1.8937285 | 1.2024899 | 0.9503682 | 0.3677526 | 2 | 21416700 | 21422016 |
| FBXL7 | 0 | 0.9166891 | 0.5683582 | 0 | 0.1782692 | 0.2759308 | 2 | 21430651 | 21531256 |
| 5S_rRNA (2 214518 | 0 | 0 | 0 | 0 | 0 | 0 | 2 | 21451846 | 21451960 |
| chd7 | 33.377344 | 43.082707 | 47.950452 | 50.313896 | 48.955852 | 44.324814 | 2 | 21562193 | 21617220 |
| rab2a | 97.407452 | 76.117653 | 81.781842 | 102.74421 | 108.9776 | 108.23782 | 2 | 21618206 | 21644458 |
| ca8 | 10.732609 | 10.597415 | 9.6078413 | 6.8880439 | 7.9713551 | 5.7177581 | 2 | 21651559 | 21675050 |
| CR847864.1 | 0 | 0 | 0 | 0 | 0 | 0 | 2 | 21685669 | 21685775 |
| tox | 17.8247 | 17.202273 | 18.317034 | 17.299345 | 17.599389 | 16.884856 | 2 | 21779436 | 21901069 |
| cyp7a1a | 0 | 0 | 0 | 0 | 0 | 0 | 2 | 21946657 | 21949389 |
| fam110b | 1.9607305 | 1.4943643 | 2.0589415 | 1.0214045 | 1.9374007 | 2.2490783 | 2 | 22001581 | 22023508 |
| sept2 | 92.078821 | 87.465468 | 81.34445 | 97.889889 | 101.32524 | 103.04648 | 2 | 22049350 | 22073010 |
| stk25b | 29.590317 | 23.948836 | 20.661917 | 16.400019 | 22.407992 | 19.38213 | 2 | 22075319 | 22094365 |
| bokb | 1.2369883 | 0.8642027 | 0.8930267 | 0.7088236 | 0.2801035 | 1.3006598 | 2 | 22097859 | 22108079 |
| thap4 | 5.3168274 | 7.2385499 | 4.1336725 | 2.8123089 | 4.0748805 | 5.160462 | 2 | 22110767 | 22117094 |
| FBXO15 (1 of 2) | 1.2216069 | 1.8207077 | 3.1749202 | 0 | 0.4425929 | 1.0275894 | 2 | 22117431 | 22134663 |
| 5S_rRNA (2 221303 | 0 | 0 | 0 | 0 | 0 | 0 | 2 | 22130366 | 22130480 |
| agxtb | 0 | 0 | 0 | 0 | 0 | 0 | 2 | 22134387 | 22146295 |
| kif1ab | 0.0830641 | 0.7737522 | 0.7196034 | 0.4759767 | 0.5266525 | 0.2329056 | 2 | 22152026 | 22216234 |
| 5S_rRNA (2 221593 | 0 | 0 | 0 | 0 | 0 | 0 | 2 | 22159306 | 22159420 |
| espnl | 0 | 0.3668304 | 0 | 0.9026285 | 0 | 0 | 2 | 22223136 | 22246097 |
| rab6bb | 4.4714219 | 1.1359598 | 4.695391 | 3.2610208 | 6.9955127 | 5.1289957 | 2 | 22253138 | 22259454 |
| aire | 0 | 0 | 0.2914311 | 0.6939542 | 0 | 0 | 2 | 22266590 | 22304197 |
| amotl2b | 11.724971 | 8.8831933 | 10.834792 | 15.229022 | 10.195206 | 11.397005 | 2 | 22309476 | 22318822 |
| myo7bb | 0 | 0.0678044 | 0 | 0.5005217 | 0.1318597 | 0.5102417 | 2 | 22338706 | 22385207 |
| 5S_rRNA (2 223549 | 0 | 0 | 0 | 0 | 0 | 0 | 2 | 22354929 | 22355045 |
| sap130b | 7.3434246 | 11.228734 | 10.202856 | 12.862055 | 13.05176 | 10.295217 | 2 | 22391020 | 22423741 |

| | | | | | | | | | |
|---|---|---|---|---|---|---|---|---|---|
| MLLT1 (2 of 2) | 7.2162966 | 8.5673463 | 7.9677853 | 9.8918289 | 6.7925533 | 6.3479141 | 2 | 22427784 | 22451277 |
| zgc:136639os | 0 | 0 | 0 | 0 | 0 | 0 | 2 | 22443926 | 22449165 |
| ZNF414 | 7.3577927 | 4.7449918 | 7.7634831 | 7.7837342 | 6.9207147 | 5.1576802 | 2 | 22451602 | 22461949 |
| mknk2a | 1.0478754 | 1.4099355 | 1.0086656 | 0.26687 | 0.738207 | 0.326463 | 2 | 22464436 | 22482596 |
| csnk1g2a | 21.145705 | 19.697492 | 24.258063 | 20.582284 | 19.808849 | 25.178385 | 2 | 22496685 | 22536392 |
| mfsd12a | 2.1144998 | 1.2121128 | 2.1136624 | 0.932045 | 1.031277 | 0 | 2 | 22538891 | 22557288 |
| si:dkey-218h11.6 | 0 | 0 | 0 | 0 | 0 | 0 | 2 | 22588033 | 22589778 |
| prrx1a | 14.430072 | 20.048778 | 27.544817 | 5.886245 | 5.7597392 | 6.5148879 | 2 | 22622894 | 22630352 |
| kifap3b | 5.2415695 | 3.733744 | 7.3455647 | 1.0600741 | 2.9323426 | 2.5935853 | 2 | 22680583 | 22750209 |
| fam129ab | 0 | 1.2127771 | 0.5370973 | 0.2131556 | 0 | 0 | 2 | 22753914 | 22806760 |
| rnf2 | 60.183532 | 60.589784 | 59.825477 | 64.728674 | 52.624869 | 63.281359 | 2 | 22809088 | 22819674 |
| ivns1abpb | 58.669598 | 59.390897 | 80.579149 | 24.599318 | 23.90605 | 24.073951 | 2 | 22837433 | 22849650 |
| si:ch73-129a22.11 (2 | 0 | 0 | 0.3043309 | 0.9662284 | 0.7636427 | 0.5909954 | 2 | 22861058 | 22870500 |
| FP016152.1 | 0 | 0 | 1.6158 | 1.9237676 | 1.5204181 | 3.1378029 | 2 | 22890713 | 22896737 |
| si:ch73-129a22.11 (2 | 1.6548009 | 0 | 0 | 0.6321601 | 0.4996173 | 0 | 2 | 22929932 | 22931788 |
| CLDN18 | 0 | 0 | 0 | 0 | 0 | 1.6127327 | 2 | 22977987 | 22981393 |
| hfm1 | 0.1207696 | 0.1124984 | 0.5231278 | 0.2768154 | 0.1093883 | 0 | 2 | 22984098 | 23009022 |
| znf644a | 6.1360062 | 4.828838 | 6.8738353 | 4.8497611 | 5.2702773 | 3.1146837 | 2 | 23014596 | 23039201 |
| lrrc8da | 1.8031447 | 2.5194782 | 3.3845641 | 1.8598403 | 1.4698942 | 2.527944 | 2 | 23046115 | 23050291 |
| gtf2b | 34.22525 | 41.029788 | 40.822652 | 50.138194 | 50.678019 | 50.068768 | 2 | 23053276 | 23065450 |
| pkn2 | 4.4607676 | 4.6914243 | 6.4407792 | 6.4315414 | 4.9527281 | 5.6486257 | 2 | 23068424 | 23128679 |
| 5S_rRNA (2 23111C | 0 | 0 | 0 | 0 | 0 | 0 | 2 | 23111019 | 23111135 |
| zgc:56628 | 0.3277064 | 0 | 0.4731662 | 0 | 0 | 0 | 2 | 23132627 | 23136270 |
| hs2st1a | 9.4178375 | 11.185364 | 15.297925 | 13.49161 | 15.567793 | 11.222949 | 2 | 23149705 | 23181558 |
| sep15 | 159.41591 | 139.94806 | 129.7357 | 119.03063 | 133.45368 | 135.45174 | 2 | 23182115 | 23192145 |
| si:dkey-43b14.6 | 0 | 0.3104957 | 0 | 0.7640106 | 0 | 0 | 2 | 23194397 | 23196918 |
| si:dkey-43b14.7 | 0.9150075 | 0.2841137 | 0 | 0 | 0 | 0 | 2 | 23195649 | 23238279 |
| si:dkey-43b14.8 | 0.7291466 | 2.037628 | 1.0527947 | 0 | 1.3208633 | 0 | 2 | 23230502 | 23231814 |
| si:dkey-43b14.9 | 0 | 0 | 0 | 0 | 0.3001962 | 0.4646533 | 2 | 23235660 | 23238142 |
| si:dkey-43b14.3 | 0 | 0 | 0 | 0 | 0 | 0 | 2 | 23258737 | 23258827 |
| crsp7 | 4.2848016 | 3.991347 | 5.9054928 | 5.8034193 | 7.585592 | 7.0993445 | 2 | 23298624 | 23304670 |
| cnn2 | 71.558665 | 63.8213 | 67.598694 | 44.49458 | 36.716982 | 37.087371 | 2 | 23309714 | 23315897 |
| angptl4 | 6.475501 | 13.815251 | 15.985137 | 14.842462 | 12.676503 | 8.1998627 | 2 | 23321879 | 23327384 |
| rps28 | 1140.0902 | 1050.5332 | 907.13715 | 970.78805 | 1036.8984 | 1011.1604 | 2 | 23354448 | 23356528 |
| 5S_rRNA (2 23358C | 0 | 0 | 0 | 0 | 0 | 0 | 2 | 23358056 | 23358171 |
| si:rp71-39b20.4 | 0 | 0 | 0 | 0 | 0 | 0.8076937 | 2 | 23359248 | 23366668 |
| si:rp71-39b20.6 | 0 | 0 | 0 | 0 | 0 | 0 | 2 | 23366305 | 23379871 |
| insl3 | 0 | 0 | 0 | 0 | 0 | 0 | 2 | 23375494 | 23376826 |
| kcnn1a | 0.5236577 | 0.3484241 | 0.6480814 | 0.6001366 | 0.4065498 | 0.2097569 | 2 | 23401945 | 23453652 |
| OCEL1 | 0 | 0 | 0 | 0 | 0.253555 | 0 | 2 | 23456213 | 23461254 |
| zgc:154006 | 0.679593 | 0.422033 | 0.3270819 | 0 | 0 | 0 | 2 | 23461785 | 23477693 |
| nr2f6a | 24.639804 | 26.451114 | 25.380962 | 31.509902 | 28.577595 | 24.64428 | 2 | 23477764 | 23489841 |
| si:dkey-208k22.3 | 0 | 0 | 0 | 0 | 0 | 0 | 2 | 23495059 | 23498314 |
| si:dkey-208k22.2 | 1.3725113 | 1.9177675 | 0.4954328 | 1.572963 | 0.6215827 | 0 | 2 | 23508686 | 23511649 |
| si:dkey-208k22.1 | 0 | 0 | 1.2641437 | 0 | 0 | 0 | 2 | 23513106 | 23515557 |
| ano8a | 0.1779763 | 0.0828936 | 0.1284875 | 0.815877 | 1.0478244 | 0.2495164 | 2 | 23515198 | 23544177 |
| mrpl34 | 71.21825 | 62.165413 | 61.841858 | 53.081205 | 74.430715 | 53.0647 | 2 | 23543963 | 23546681 |
| C2H6orf52 | 28.696626 | 27.974585 | 38.543503 | 39.771162 | 35.210427 | 35.085729 | 2 | 23546710 | 23553511 |
| fitm1 | 13.342043 | 8.2855215 | 8.2560892 | 0 | 2.8773059 | 0.8907174 | 2 | 23555464 | 23559292 |
| vmhcl | 0 | 0.0642373 | 0.0995698 | 0.1580634 | 0 | 0.0966798 | 2 | 23574503 | 23589822 |
| vmhc | 2.259259 | 4.4267666 | 3.8245097 | 0.0892834 | 0.0705636 | 0.1092207 | 2 | 23594235 | 23607626 |
| mir736 | 0 | 0 | 0 | 0 | 0 | 0 | 2 | 23602850 | 23602929 |
| MAP4 (1 of 3) | 0.2083586 | 0.2587849 | 0.2005622 | 0 | 0 | 0.1947409 | 2 | 23611810 | 23687140 |
| kcnh2 | 0 | 0.3466459 | 0 | 0.1421604 | 0.1123541 | 0 | 2 | 23695890 | 23783096 |
| si:dkeyp-99c4.5 | 0 | 0 | 0 | 0 | 0 | 0 | 2 | 23758671 | 23788133 |
| si:dkeyp-99c4.4 | 2.5710956 | 2.3950081 | 9.2808349 | 13.259688 | 17.46596 | 28.836668 | 2 | 23795298 | 23796544 |
| slc12a7a | 2.4033675 | 5.3481661 | 5.0124475 | 14.99603 | 11.247179 | 8.9851591 | 2 | 23802571 | 23832951 |
| si:dkeyp-99c4.2 | 0 | 0 | 0 | 0 | 0 | 0 | 2 | 23849203 | 23850896 |
| col15a1b | 19.686526 | 18.940914 | 21.618886 | 16.418132 | 19.756703 | 15.67876 | 2 | 23850967 | 23927192 |
| tgfbr1a | 2.4661683 | 4.594534 | 2.7695363 | 4.7105761 | 7.942243 | 11.909095 | 2 | 23932610 | 24008101 |
| VOPP1 (2 of 2) | 0 | 0 | 0 | 0 | 0 | 0 | 2 | 24026726 | 24040524 |
| gorasp1 | 9.5125592 | 4.8485097 | 9.9772545 | 17.586936 | 14.305965 | 10.694175 | 2 | 24043868 | 24055506 |
| csrnp1a | 2.6836324 | 5.2774348 | 4.7358944 | 6.8345994 | 4.3212907 | 2.9262769 | 2 | 24060404 | 24073222 |
| si:dkey-24c2.7 | 2.9818137 | 0 | 0 | 0 | 0 | 0 | 2 | 24080107 | 24091068 |
| xirp1 | 6.7123071 | 7.0825901 | 11.235528 | 7.0799351 | 5.8645253 | 6.2458493 | 2 | 24106798 | 24114653 |

| | | | | | | | | | |
|---|---|---|---|---|---|---|---|---|---|
| si:dkey-24c2.6 | 0 | 0 | 0 | 1.0351111 | 0 | 0 | 2 | 24123890 | 24132952 |
| oxsr1a | 5.0149452 | 8.3594994 | 10.67086 | 15.72963 | 11.475373 | 11.841301 | 2 | 24142810 | 24162533 |
| 5S_rRNA (2 241841 | 0 | 0 | 0 | 0 | 0 | 0 | 2 | 24184157 | 24184273 |
| cdyl | 3.7417834 | 4.7303467 | 3.087233 | 5.207174 | 7.7466436 | 7.4940653 | 2 | 24187298 | 24239708 |
| rpp40 | 9.3783518 | 6.6273509 | 9.3387195 | 4.4474628 | 9.9591076 | 4.9872148 | 2 | 24243923 | 24251106 |
| si:dkey-58b18.8 | 0 | 0 | 0 | 0 | 1.0755121 | 0.8323561 | 2 | 24255064 | 24259492 |
| si:dkey-58b18.9 | 0 | 0 | 0 | 0 | 0 | 0 | 2 | 24261085 | 24261683 |
| si:dkey-58b18.10 (2 | 0 | 0 | 0 | 0 | 0 | 0 | 2 | 24263325 | 24264250 |
| si:dkey-58b18.5 | 0 | 0 | 0 | 0 | 0.5418926 | 0.8387588 | 2 | 24286474 | 24290464 |
| si:dkey-58b18.6 | 0.7302877 | 0 | 0 | 0 | 0 | 0 | 2 | 24292042 | 24292916 |
| si:dkey-58b18.10 (2 | 0 | 0 | 0 | 0 | 0 | 0 | 2 | 24294313 | 24295239 |
| si:dkey-58b18.2 | 0 | 0 | 0 | 0 | 0.5418926 | 0.8387588 | 2 | 24317519 | 24321510 |
| si:dkey-58b18.3 | 0 | 0 | 0 | 0 | 0.6165955 | 1.4315796 | 2 | 24317628 | 24325424 |
| si:dkey-58b18.10 (2 | 0 | 0 | 0 | 0 | 0 | 0 | 2 | 24325352 | 24326278 |
| CRTC1 (1 of 2) | 0 | 0.9790405 | 1.8969274 | 0.9033909 | 0.2379934 | 0 | 2 | 24347533 | 24372807 |
| MAST3 (2 of 2) | 2.2822196 | 5.3707363 | 4.3358341 | 6.4699995 | 5.4398487 | 5.5571822 | 2 | 24410684 | 24452847 |
| pik3r2 | 4.9064073 | 4.7396539 | 8.9208773 | 11.454208 | 9.7110196 | 8.1524224 | 2 | 24470404 | 24517964 |
| ifi30 | 90.653248 | 84.800955 | 88.36572 | 198.14176 | 226.23573 | 205.92217 | 2 | 24532156 | 24537113 |
| mpv17l2 | 23.885034 | 20.858636 | 19.829991 | 31.137219 | 31.910299 | 36.834553 | 2 | 24540024 | 24548501 |
| pde4ca | 0.3104816 | 0.1446088 | 0 | 0.3558266 | 0.5624434 | 0 | 2 | 24550615 | 24632413 |
| U3 (2 24598592..24 | 0 | 0 | 0 | 0 | 0 | 0 | 2 | 24598593 | 24598798 |
| rab3aa | 0.9689656 | 1.0831245 | 2.5183129 | 1.1104805 | 1.93083 | 1.3584549 | 2 | 24637599 | 24648248 |
| si:dkey-149i17.7 | 0 | 0 | 0 | 0 | 0 | 0 | 2 | 24655576 | 24656341 |
| si:dkey-149i17.9 | 0 | 0 | 0 | 0 | 0 | 0 | 2 | 24666576 | 24668529 |
| si:dkey-149i17.8 | 0 | 0 | 0 | 0 | 0 | 0 | 2 | 24666576 | 24671484 |
| agtr1a | 0 | 0 | 0 | 0 | 0 | 0 | 2 | 24701266 | 24702342 |
| gyg1a | 3.5568129 | 4.6385027 | 5.9059215 | 4.076276 | 5.3156692 | 3.7398927 | 2 | 24706355 | 24714408 |
| hltf | 6.0416766 | 6.7290088 | 8.9130495 | 6.3219564 | 5.1154175 | 6.8129975 | 2 | 24713629 | 24731591 |
| nck1a | 1.0789684 | 1.256341 | 1.5578927 | 1.8548234 | 0.9772861 | 1.8908436 | 2 | 24733456 | 24750945 |
| slc35g2a | 0.193552 | 0.1802961 | 0 | 0 | 0 | 0 | 2 | 24759987 | 24766030 |
| stag1a | 2.6248566 | 3.2955522 | 6.9207928 | 1.5695008 | 1.6539056 | 0.6399921 | 2 | 24788976 | 24875072 |
| pccb | 16.907748 | 22.469688 | 21.808617 | 28.161356 | 27.565842 | 26.864595 | 2 | 24878158 | 24889628 |
| msl2a | 4.2516373 | 5.4626952 | 5.5037713 | 7.0568366 | 5.3116713 | 7.1938789 | 2 | 24894944 | 24898998 |
| nceh1a | 2.9913708 | 2.0898749 | 2.1595789 | 1.1427509 | 2.0320973 | 2.7958628 | 2 | 24901206 | 24909611 |
| 5S_rRNA (2 249254 | 0 | 0 | 0 | 0 | 0 | 0 | 2 | 24925431 | 24925547 |
| si:dkey-223d7.6 | 0 | 0 | 0 | 0 | 0 | 0 | 2 | 24927931 | 24928898 |
| si:dkeyp-7b3.1 | 8.9914036 | 6.7004851 | 6.4912198 | 4.1218297 | 2.8504178 | 4.4119685 | 2 | 24990341 | 24995543 |
| si:dkey-5n7.1 | 0 | 0 | 0 | 0 | 0 | 0 | 2 | 25219747 | 25346191 |
| si:dkey-5n7.2 | 0 | 0 | 0 | 1.4092422 | 1.1137714 | 0 | 2 | 25230338 | 25231382 |
| ppp2r3a | 0 | 0.7028197 | 0.9078262 | 0 | 0.569491 | 0.5288859 | 2 | 25393831 | 25437998 |
| fndc3ba | 0.9973604 | 1.7548795 | 3.0401291 | 3.6830717 | 2.1078606 | 4.3501527 | 2 | 25463828 | 25688438 |
| ghsra | 0 | 0 | 0 | 0 | 0 | 0 | 2 | 25699627 | 25701802 |
| pld1a | 2.08607 | 1.7488805 | 2.7108169 | 2.7493452 | 0.6613173 | 0.4386892 | 2 | 25708726 | 25780260 |
| tnika | 1.582548 | 2.3955157 | 3.4274961 | 4.0807687 | 3.4043445 | 2.634677 | 2 | 25806282 | 25963360 |
| si:dkey-153n9.5 | 2.2195189 | 2.06751 | 0 | 1.2718369 | 3.5181138 | 2.3337642 | 2 | 25962722 | 25965002 |
| slc2a2 | 10.886628 | 17.815327 | 15.718902 | 42.319249 | 37.443892 | 30.31907 | 2 | 25965013 | 25986156 |
| eif5a2 | 308.34261 | 296.48041 | 313.94054 | 214.45435 | 244.18627 | 267.21821 | 2 | 25987363 | 25993197 |
| si:dkey-153n9.4 | 0 | 0 | 0 | 0 | 5.196839 | 0 | 2 | 26000849 | 26001969 |
| 5S_rRNA (2 260037 | 0 | 0 | 0 | 0 | 0 | 0 | 2 | 26003798 | 26003915 |
| slc7a14a | 0.0787203 | 0.2933158 | 0.2273241 | 0.541303 | 0.7130166 | 0.772541 | 2 | 26016574 | 26139598 |
| cldn11a | 0 | 0 | 0.5119974 | 0 | 0 | 0 | 2 | 26145596 | 26149343 |
| si:ch211-253d24.1 | 0 | 0 | 0 | 0 | 0 | 0 | 2 | 26182345 | 26187776 |
| si:ch211-253d24.2 | 0 | 0.4242987 | 0 | 0 | 0 | 0 | 2 | 26202283 | 26206082 |
| grin3b | 0 | 0.1294888 | 0.2007115 | 0.3186222 | 0 | 0.1948859 | 2 | 26208810 | 26304333 |
| 5S_rRNA (2 262333 | 0 | 0 | 0 | 0 | 0 | 0 | 2 | 26233332 | 26233449 |
| lppr3a | 12.706833 | 18.183726 | 18.347046 | 17.728422 | 17.681011 | 12.392712 | 2 | 26327266 | 26356309 |
| palm1b | 17.265378 | 15.627741 | 12.464501 | 15.120209 | 18.736434 | 21.236847 | 2 | 26385492 | 26432415 |
| si:dkey-57i11.4 | 0 | 0 | 0 | 0 | 0 | 0 | 2 | 26434635 | 26435675 |
| ptbp1a | 47.807894 | 39.057362 | 46.766407 | 36.611489 | 35.867701 | 39.033089 | 2 | 26436471 | 26448682 |
| efna2 | 4.9232509 | 4.5860705 | 3.554273 | 2.6196293 | 4.6185422 | 3.4511102 | 2 | 26451797 | 26592673 |
| si:dkey-15h14.4 | 0 | 0 | 0 | 0 | 0 | 0 | 2 | 26583106 | 26584283 |
| rabggtb | 14.324962 | 20.355077 | 12.970957 | 16.417085 | 21.331735 | 27.912079 | 2 | 26611284 | 26617263 |
| acadm | 12.981258 | 16.470419 | 14.86536 | 13.08162 | 12.974235 | 18.199257 | 2 | 26618240 | 26624320 |
| hectd3 | 10.370086 | 16.137658 | 14.796926 | 16.638453 | 15.581007 | 17.959307 | 2 | 26627846 | 26643214 |
| rps8a | 1660.9713 | 1758.8465 | 1616.1476 | 1366.5564 | 1413.5321 | 1558.5326 | 2 | 26646616 | 26650038 |

| | | | | | | | | | |
|---|---|---|---|---|---|---|---|---|---|
| SNORD46 | 0 | 0 | 0 | 0 | 0 | 0 | 2 | 26647819 | 26647924 |
| SNORD38 | 0 | 0 | 0 | 0 | 0 | 0 | 2 | 26649203 | 26649273 |
| SNORA47 | 0 | 0 | 0 | 0 | 0 | 0 | 2 | 26649938 | 26650070 |
| best4 | 0 | 0 | 0 | 0 | 0 | 0 | 2 | 26653135 | 26657227 |
| si:ch211-106k21.5 | 7.6230813 | 7.1009967 | 7.1293764 | 9.2327991 | 12.004727 | 9.958607 | 2 | 26662307 | 26675900 |
| glis1a | 0 | 0 | 0 | 0 | 0 | 0.3839389 | 2 | 26677617 | 26712148 |
| ndc1 | 29.540473 | 23.263439 | 29.259322 | 24.3689 | 25.463982 | 26.20929 | 2 | 26725023 | 26738798 |
| zgc:113691 | 1.4027671 | 3.0489557 | 3.3756945 | 0.5358792 | 2.1176164 | 0.655543 | 2 | 26740138 | 26744964 |
| yipf1 | 4.7723604 | 4.4455141 | 3.2955423 | 8.0851275 | 4.6984909 | 4.0725863 | 2 | 26746971 | 26752488 |
| lrrc42 | 3.0361343 | 5.0907557 | 3.5070325 | 2.783643 | 1.9250057 | 5.1078613 | 2 | 26752943 | 26759203 |
| tmem59 | 16.101607 | 14.453438 | 14.371903 | 16.104614 | 17.766191 | 20.932137 | 2 | 26760744 | 26769072 |
| u2surp | 21.611756 | 20.87724 | 29.55358 | 30.272117 | 22.785781 | 20.359581 | 2 | 26771547 | 26791114 |
| mir124-1 | 0 | 0 | 0 | 0 | 0 | 0 | 2 | 26793487 | 26793568 |
| ttpa | 0 | 0 | 0 | 0 | 0.2198004 | 0 | 2 | 26795642 | 26801318 |
| asph | 90.00596 | 80.909522 | 86.63808 | 98.52902 | 95.779292 | 74.056183 | 2 | 26804453 | 26830352 |
| si:dkey-181m9.5 | 0 | 0 | 0 | 0 | 0 | 0 | 2 | 26815850 | 26821017 |
| impa1 | 17.046716 | 14.555962 | 18.972739 | 14.652258 | 8.3634568 | 14.936801 | 2 | 26831083 | 26837078 |
| si:dkey-181m9.8 | 0.9865832 | 1.9215763 | 1.0360002 | 1.850189 | 1.8684516 | 1.1316716 | 2 | 26838033 | 26869024 |
| si:dkey-148a12.2 | 0 | 3.4961981 | 14.45123 | 2.8676 | 6.7990816 | 7.0158917 | 2 | 27148712 | 27158515 |
| cdh7 | 5.3208954 | 8.4365642 | 8.8269251 | 4.670807 | 3.7940371 | 5.0789473 | 2 | 27158567 | 27244127 |
| 5S_rRNA (2 271878 | 0 | 0 | 0 | 0 | 0 | 0 | 2 | 27187858 | 27187974 |
| 5S_rRNA (2 273818 | 0 | 0 | 0 | 0 | 0 | 0 | 2 | 27381836 | 27381951 |
| dsel | 0.3914881 | 0.1215587 | 0 | 0 | 0.2363961 | 0 | 2 | 27460033 | 27463608 |
| tmx3 | 0 | 0 | 0 | 0 | 0 | 0 | 2 | 27466576 | 27478351 |
| si:dkey-40i22.5 | 0 | 0 | 0 | 0 | 0 | 0 | 2 | 27469184 | 27472351 |
| tesk2 | 3.0753692 | 3.8611785 | 2.702877 | 2.9115589 | 2.5433241 | 3.1868029 | 2 | 27478631 | 27527497 |
| toe1 | 10.218697 | 5.892619 | 11.944115 | 12.268783 | 15.205767 | 11.256336 | 2 | 27526657 | 27534104 |
| si:ch73-382f3.1 | 5.1191819 | 3.5136926 | 5.0573048 | 8.0282867 | 8.0533002 | 4.5327845 | 2 | 27534787 | 27539728 |
| sepp1b | 49.029297 | 49.477362 | 42.559506 | 120.07246 | 114.72263 | 69.964762 | 2 | 27542968 | 27549275 |
| zgc:153394 | 0 | 0.9805127 | 0.506608 | 2.412665 | 1.5890084 | 0 | 2 | 27551470 | 27557876 |
| si:ch73-176g19.2 | 0 | 0 | 0 | 0 | 0 | 0 | 2 | 27632729 | 27633479 |
| zswim5 | 17.083667 | 18.07735 | 18.608163 | 26.362536 | 22.735475 | 20.168999 | 2 | 27639236 | 27692112 |
| urod | 39.369404 | 50.131116 | 36.505575 | 26.905945 | 31.733433 | 36.458744 | 2 | 27704338 | 27723973 |
| lyn | 0 | 0 | 0.2041784 | 0 | 0 | 0 | 2 | 27725640 | 27757359 |
| tgs1 | 6.2386878 | 2.8226881 | 3.8605153 | 3.8813372 | 2.5832009 | 1.9991807 | 2 | 27761944 | 27799950 |
| tmem68 (2 2780337 | 1.8741118 | 1.7457589 | 1.3529892 | 4.2956418 | 0.8487475 | 3.941156 | 2 | 27803371 | 27804687 |
| tmem68 (2 2782390 | 0.9314448 | 0 | 0 | 0 | 4.2183258 | 3.9175563 | 2 | 27823907 | 27827694 |
| CABZ01085085.1 | 1.7283476 | 4.0249442 | 3.7432701 | 3.4663443 | 0.3913669 | 0 | 2 | 27829274 | 27849933 |
| dhcr7 | 2.0966476 | 2.5110691 | 2.8110565 | 1.029796 | 3.9337648 | 3.9892189 | 2 | 27967560 | 27989080 |
| nadsyn1 | 0.7777564 | 2.4149665 | 2.245962 | 1.4855761 | 0.9392805 | 2.5442352 | 2 | 27990088 | 28011192 |
| CU693487.1 | 2.1874399 | 4.075256 | 4.2111788 | 4.7352739 | 2.2014388 | 0.6814916 | 2 | 28011847 | 28021904 |
| buc | 0 | 0 | 0 | 0 | 0 | 0 | 2 | 28022981 | 28027100 |
| rnf152 | 2.3568376 | 2.9272321 | 4.5372971 | 0.9003492 | 7.1157617 | 5.5070025 | 2 | 28037861 | 28038454 |
| zgc:163121 | 1.8079572 | 0 | 0.8701532 | 0.6906682 | 0 | 0 | 2 | 28045787 | 28068414 |
| mc4r | 0 | 0 | 0 | 0 | 0 | 0 | 2 | 28100596 | 28101736 |
| C2H5orf22 | 2.0220918 | 3.7672082 | 0.3244047 | 0 | 1.4245227 | 0.6299778 | 2 | 28125521 | 28143409 |
| 5S_rRNA (2 281731 | 0 | 0 | 0 | 0 | 0 | 0 | 2 | 28173131 | 28173245 |
| cdh6 | 23.517786 | 30.629393 | 27.511201 | 29.697658 | 29.7826 | 28.086484 | 2 | 28216925 | 28270171 |
| cdh10 | 0 | 0 | 0.2444806 | 0 | 0 | 0 | 2 | 28604768 | 28649116 |
| CDH12 (2 of 2) | 0 | 0 | 0 | 0 | 0 | 0 | 2 | 28710608 | 28831724 |
| cdh18 | 1.9884052 | 1.1576404 | 0.7177509 | 0.712127 | 0.5628179 | 0.8711477 | 2 | 28964393 | 29067510 |
| cahz | 23.101676 | 26.729488 | 30.195842 | 33.442881 | 40.087064 | 45.001882 | 2 | 29192215 | 29200240 |
| 5S_rRNA (2 292156 | 0 | 0 | 0 | 0 | 0 | 0 | 2 | 29215697 | 29215811 |
| dlgap1a | 0 | 0 | 0 | 0 | 0.2812217 | 0 | 2 | 29324946 | 29407657 |
| 5S_rRNA (2 293927 | 0 | 0 | 0 | 0 | 0 | 0 | 2 | 29392742 | 29392860 |
| ptf1a | 0 | 1.6341879 | 0.8443466 | 0 | 0 | 0 | 2 | 29425689 | 29426593 |
| si:ch211-142h2.1 | 0 | 0 | 0 | 0 | 0 | 0 | 2 | 29427529 | 29441760 |
| C2H10orf67 | 0 | 0 | 0 | 0 | 0 | 0 | 2 | 29441590 | 29476797 |
| si:dkey-188g12.2 | 0 | 0 | 0 | 1.2389361 | 0 | 0 | 2 | 29479227 | 29482122 |
| si:ch211-207d6.4 | 0 | 0.8049888 | 0 | 0.9903841 | 0 | 1.2115406 | 2 | 29557420 | 29614710 |
| si:ch211-207d6.2 | 0.1642569 | 0.5355258 | 1.422996 | 1.6000926 | 2.1572705 | 0.921129 | 2 | 29557670 | 29608149 |
| paxip1 | 16.147192 | 17.586767 | 23.493827 | 16.085504 | 13.725446 | 9.5775231 | 2 | 29617508 | 29638235 |
| htr5a | 0 | 0.9525799 | 0.4921758 | 0.781311 | 0.9262445 | 0.4778904 | 2 | 29640509 | 29653812 |
| eng2b | 19.177555 | 10.718481 | 11.445176 | 7.9122191 | 4.8636718 | 5.0187653 | 2 | 29691251 | 29694788 |
| cnpy1 | 20.209419 | 22.248117 | 27.057653 | 14.738787 | 18.304877 | 20.090586 | 2 | 29701087 | 29710868 |

| | | | | | | | | | |
|---|---|---|---|---|---|---|---|---|---|
| RBM33 | 0 | 0 | 1.3784546 | 0 | 0 | 0.535378 | 2 | 29710422 | 29762469 |
| shhb | 3.6980989 | 3.4448259 | 5.3395829 | 3.7084218 | 2.5121916 | 1.620188 | 2 | 29765373 | 29770264 |
| dnajb6a | 24.229499 | 23.742557 | 29.532205 | 23.801274 | 19.09596 | 24.704644 | 2 | 29818117 | 29833015 |
| 5S_rRNA (2 298284 | 0 | 0 | 0 | 0 | 0 | 0 | 2 | 29828467 | 29828581 |
| trpa1a | 0 | 0.4096056 | 0.1587252 | 0 | 0.1991408 | 0 | 2 | 29831318 | 29850278 |
| kcnb2 | 0.1965272 | 0.3661352 | 0 | 0.450459 | 0.1780064 | 0.4132861 | 2 | 29859811 | 29892301 |
| rpl7 | 2475.6458 | 2763.3818 | 2308.6501 | 2093.4184 | 2163.4189 | 2201.972 | 2 | 29892308 | 29897185 |
| SNORD12 (2 29895 | 0 | 0 | 0 | 0 | 0 | 0 | 2 | 29895962 | 29896051 |
| rdh10b | 23.55269 | 16.876638 | 16.816687 | 21.356702 | 36.102131 | 44.994318 | 2 | 29897263 | 29901139 |
| ube2w | 1.3964704 | 1.7344398 | 2.3523792 | 3.734316 | 1.8972998 | 2.9367018 | 2 | 29908579 | 29915038 |
| tceb1b | 31.935808 | 24.284579 | 38.112345 | 26.14282 | 22.727703 | 26.498219 | 2 | 29960685 | 29964617 |
| tmem70 | 10.467002 | 11.781425 | 15.113016 | 9.4965801 | 20.541275 | 12.228634 | 2 | 29964809 | 29967179 |
| si:dkey-82k12.7 | 16.824332 | 14.022386 | 21.735117 | 11.162963 | 7.2183798 | 7.4485605 | 2 | 29984138 | 29984995 |
| si:dkey-82k12.8 | 0.906124 | 1.6881319 | 2.6166548 | 0 | 0 | 0 | 2 | 29991049 | 29991894 |
| jph1b | 1.2403676 | 2.1824564 | 2.785895 | 1.5794666 | 1.1234749 | 2.3186009 | 2 | 30005551 | 30039129 |
| CR788254.1 | 0 | 0 | 0 | 0 | 0 | 0 | 2 | 30061436 | 30062203 |
| 5S_rRNA (2 300758 | 0 | 0 | 0 | 0 | 0 | 0 | 2 | 30075845 | 30075960 |
| pi15b | 0 | 0 | 0 | 0 | 0 | 0 | 2 | 30083902 | 30087881 |
| crispld1b | 0 | 0 | 0 | 0 | 0 | 0 | 2 | 30094482 | 30111291 |
| socs6b | 9.3117059 | 9.9537382 | 10.579605 | 11.721327 | 10.093348 | 11.984621 | 2 | 30138260 | 30145177 |
| cbln2a | 0 | 0 | 0 | 0 | 0 | 0 | 2 | 30173538 | 30175125 |
| neto1 | 0.2559813 | 0 | 0 | 0 | 0 | 0 | 2 | 30178657 | 30191780 |
| fam173b | 10.251231 | 11.209873 | 7.7225056 | 10.215996 | 8.477747 | 9.3729498 | 2 | 30195739 | 30198041 |
| march6 | 4.3163274 | 8.5897063 | 8.215207 | 14.502871 | 10.840131 | 9.6271249 | 2 | 30204678 | 30241553 |
| ankrd33bb | 0 | 0 | 0 | 0.3906555 | 0.9262445 | 0 | 2 | 30246558 | 30264097 |
| ctnnd2b | 7.2879078 | 6.4351962 | 9.426683 | 7.4822575 | 5.6384337 | 5.8537098 | 2 | 30269088 | 30408574 |
| GPR141 (1 of 2) | 0 | 0 | 0 | 0 | 0 | 0 | 2 | 30428700 | 30436334 |
| si:dkey-94e7.2 | 0 | 0 | 0 | 0 | 0 | 0 | 2 | 30435902 | 30441252 |
| rp1 | 0 | 0 | 0 | 0 | 0 | 0 | 2 | 30441997 | 30448963 |
| tcea1 | 55.248659 | 46.639998 | 63.390261 | 75.189625 | 63.222715 | 81.260305 | 2 | 30454512 | 30462842 |
| 5S_rRNA (2 304668 | 0 | 0 | 0 | 0 | 0 | 0 | 2 | 30466809 | 30466941 |
| rgs20 | 0.4210157 | 0.0784363 | 0 | 0.1930016 | 0.1525356 | 0 | 2 | 30467360 | 30499334 |
| atp6v1h | 24.512945 | 24.948389 | 23.267992 | 29.653718 | 28.987038 | 32.45703 | 2 | 30501605 | 30561705 |
| oprk1 | 0 | 0 | 0 | 0 | 0 | 0 | 2 | 30593696 | 30601399 |
| chmp5a | 0.8991404 | 2.0939016 | 3.2456099 | 0.5152287 | 0.4072025 | 1.2605624 | 2 | 30609427 | 30615952 |
| bag1 | 32.716102 | 24.112454 | 22.840292 | 30.077766 | 28.656016 | 21.169291 | 2 | 30618901 | 30623132 |
| myl12.2 | 106.7374 | 115.27796 | 92.320208 | 80.959795 | 72.859109 | 79.519899 | 2 | 30624964 | 30627754 |
| myom1a | 0.7817957 | 1.4565052 | 1.3545759 | 0.8063774 | 0.5664952 | 1.2056544 | 2 | 30631020 | 30674745 |
| lipin2 | 4.1707839 | 3.6202423 | 3.2847708 | 13.470672 | 11.590756 | 9.8340769 | 2 | 30675183 | 30699340 |
| emilin2a | 4.8490926 | 3.4541697 | 3.2948098 | 3.9227927 | 2.8419552 | 3.199178 | 2 | 30701828 | 30732894 |
| si:ch211-209l5.1 | 0 | 0 | 0 | 0 | 3.0083718 | 0 | 2 | 30816810 | 30817295 |
| si:ch211-209l5.2 | 0 | 0 | 0 | 0 | 0 | 0 | 2 | 30823651 | 30824136 |
| yes1 | 24.003228 | 23.445593 | 30.869116 | 26.506489 | 27.022409 | 25.340927 | 2 | 30986804 | 31079129 |
| adcyap1b | 0.142056 | 0.3969808 | 1.2306641 | 0.1628029 | 0.6433428 | 0 | 2 | 31012872 | 31018341 |
| clul1 | 0.6714444 | 0.4169726 | 1.29264 | 0 | 0 | 0 | 2 | 31079008 | 31092329 |
| colec12 | 10.264484 | 9.8270938 | 11.664365 | 6.971013 | 7.4032906 | 5.0633019 | 2 | 31098312 | 31150880 |
| stam | 15.818775 | 18.711605 | 17.402127 | 19.136235 | 18.421725 | 18.129106 | 2 | 31153269 | 31168328 |
| si:dkey-32m20.1 | 0 | 0 | 0 | 0 | 0.7692015 | 0 | 2 | 31170284 | 31173582 |
| cacnb2b | 0.1353797 | 0.7566475 | 0 | 0 | 0.245243 | 0.1897975 | 2 | 31191494 | 31229079 |
| mrc1b | 0 | 0.0712613 | 0 | 0 | 0 | 0 | 2 | 31231575 | 31256560 |
| CR293515.1 | 0 | 0.2634509 | 0 | 0 | 0 | 0 | 2 | 31289455 | 31295411 |
| si:ch211-106h4.4 | 0 | 0.2943087 | 0 | 1.8104516 | 0.2861721 | 0 | 2 | 31316383 | 31322411 |
| si:ch211-106h4.5 | 0 | 0.5971071 | 0.9255338 | 0.7346256 | 0 | 0 | 2 | 31326756 | 31330113 |
| PLXDC2 (2 of 2) | 0.432621 | 0.5373226 | 0.2082165 | 0 | 0 | 0.202173 | 2 | 31330255 | 31341931 |
| MYRIP (2 of 2) | 0 | 0 | 0 | 0.1235406 | 0 | 0 | 2 | 31342538 | 31377331 |
| si:ch211-106h4.12 | 0 | 0 | 0 | 0 | 0 | 0 | 2 | 31381558 | 31384833 |
| ccrl1a | 0 | 0 | 0 | 0 | 0.3557881 | 0.5507003 | 2 | 31387229 | 31390125 |
| e2f5 | 0.5699589 | 1.1945788 | 1.0286849 | 1.4696997 | 0.1290614 | 1.5981237 | 2 | 31392512 | 31402125 |
| lrrcc1 | 1.6907748 | 2.4937154 | 3.8653332 | 2.906562 | 3.3181106 | 3.7531419 | 2 | 31403044 | 31426473 |
| CABZ01071757.1 | 0 | 0 | 0 | 0 | 0 | 0 | 2 | 31436090 | 31451928 |
| and2 | 0 | 0 | 0 | 0.2670032 | 0 | 0 | 2 | 31480049 | 31484497 |
| BX323064.1 | 3.0367929 | 1.2572494 | 2.4359675 | 0.7734019 | 1.2224909 | 0.4730527 | 2 | 31499718 | 31518539 |
| zgc:153414 | 0 | 0 | 0 | 0 | 0.8263465 | 0 | 2 | 31522457 | 31523479 |
| ranbp9 | 20.926182 | 23.976394 | 21.452463 | 24.941691 | 23.503074 | 28.164243 | 2 | 31524535 | 31544786 |
| nol7 | 85.507723 | 92.785555 | 87.999682 | 85.485784 | 95.98788 | 85.445492 | 2 | 31548060 | 31551114 |

| | | | | | | | | | |
|---|---|---|---|---|---|---|---|---|---|
| epdr1 | 0.3328487 | 2.4804221 | 0.480591 | 0.7629207 | 0.9044427 | 0.9332837 | 2 | 31551907 | 31554777 |
| stard3nl | 2.5182649 | 2.7066872 | 3.6360531 | 2.6640469 | 2.6318571 | 3.5305167 | 2 | 31556322 | 31570969 |
| 5S_rRNA (2 316052 | 0 | 0 | 0 | 0 | 0 | 0 | 2 | 31605285 | 31605399 |
| amph | 1.6031624 | 0.9955774 | 0.7715873 | 1.6841917 | 0.36302 | 0.561894 | 2 | 31609102 | 31654841 |
| 5S_rRNA (2 316338 | 0 | 0 | 0 | 0 | 0 | 0 | 2 | 31633882 | 31633998 |
| fam105bb | 7.2237438 | 8.2991109 | 5.9104264 | 3.5874594 | 4.7981823 | 4.3885525 | 2 | 31660265 | 31669876 |
| ankhb | 3.5126372 | 3.7628759 | 3.2966699 | 3.6230837 | 2.7043644 | 5.1708204 | 2 | 31675429 | 31724187 |
| mycb | 21.001984 | 30.776906 | 31.0638 | 48.138537 | 30.853974 | 31.239394 | 2 | 31734131 | 31736246 |
| si:ch211-139k8.2 | 1.3117466 | 0.9164314 | 1.8939947 | 0 | 0.2970318 | 0 | 2 | 31737582 | 31807885 |
| si:ch211-139k8.3 | 0 | 0 | 1.6275087 | 0 | 0 | 0 | 2 | 31807106 | 31809856 |
| si:ch211-139k8.4 | 0.3451582 | 0.3215192 | 0 | 0.3955676 | 0.3126304 | 0 | 2 | 31833726 | 31837071 |
| fam49ba | 10.233637 | 9.151452 | 8.274597 | 9.1480214 | 11.123059 | 10.043034 | 2 | 31933330 | 31980162 |
| asap1a | 6.6610972 | 8.5463667 | 9.6177744 | 9.6504433 | 8.0824167 | 8.2814164 | 2 | 31985773 | 32070919 |
| 5S_rRNA (2 320112 | 0 | 0 | 0 | 0 | 0 | 0 | 2 | 32011276 | 32011391 |
| si:ch211-160i2.4 | 0 | 2.1734698 | 0 | 0 | 0 | 3.2711595 | 2 | 32073303 | 32073925 |
| ubtfl | 15.118375 | 14.787106 | 16.553662 | 12.99478 | 11.069005 | 8.8314241 | 2 | 32075481 | 32105316 |
| slc4a2a | 1.3753429 | 1.4733217 | 2.4822746 | 5.2014867 | 5.4189262 | 6.6522253 | 2 | 32109772 | 32175798 |
| ttc19 | 2.9457371 | 2.0579935 | 4.6077075 | 0.562659 | 2.6681299 | 3.097363 | 2 | 32193669 | 32203955 |
| faim2a | 2.6132616 | 3.1297966 | 3.2341853 | 3.8506133 | 3.3814099 | 4.7104697 | 2 | 32212087 | 32223025 |
| abcf2a | 87.836172 | 87.046927 | 89.391524 | 70.287706 | 73.249863 | 69.437548 | 2 | 32223429 | 32231413 |
| SNORD89 | 0 | 0 | 0 | 0 | 0 | 0 | 2 | 32226018 | 32226140 |
| smarcd3a | 0.6307838 | 1.8802659 | 2.5501597 | 2.7470507 | 2.0568187 | 1.2380706 | 2 | 32232503 | 32276670 |
| tmub1 | 9.7638344 | 11.235167 | 7.4635046 | 19.417623 | 20.028351 | 19.727608 | 2 | 32279058 | 32292819 |
| fastk | 2.4067381 | 3.8938385 | 3.6579186 | 5.9519825 | 5.5071823 | 5.5052087 | 2 | 32296892 | 32312652 |
| si:dkeyp-73d8.4 | 0 | 0 | 0.8206926 | 0.6514098 | 0 | 1.593744 | 2 | 32322722 | 32325902 |
| si:dkeyp-73d8.5 | 0 | 1.6947133 | 1.3134281 | 0 | 0 | 1.2753058 | 2 | 32328753 | 32329517 |
| si:dkeyp-73d8.6 | 0 | 0 | 0 | 0 | 0 | 0 | 2 | 32339552 | 32342452 |
| si:dkeyp-73d8.8 | 0 | 0 | 0 | 0 | 0 | 0 | 2 | 32354383 | 32355467 |
| si:dkeyp-73d8.9 | 0 | 0 | 0 | 0 | 0 | 0 | 2 | 32360213 | 32361613 |
| puf60a | 35.620475 | 39.148715 | 36.631012 | 36.711107 | 34.584712 | 37.36415 | 2 | 32371845 | 32389729 |
| SNORA57 (2 32384 | 0 | 0 | 0 | 0 | 0 | 0 | 2 | 32384199 | 32384334 |
| SNORA57 (2 32385 | 0 | 0 | 0 | 0 | 0 | 0 | 2 | 32385007 | 32385140 |
| nrbp2 | 3.4080093 | 3.0234322 | 3.6319678 | 4.2777153 | 4.9242407 | 3.7540693 | 2 | 32391243 | 32456410 |
| si:dkeyp-73d8.12 | 0.4980297 | 0 | 0 | 0 | 0 | 0 | 2 | 32419695 | 32437349 |
| klhl18 | 4.3920362 | 2.6593043 | 2.8536929 | 4.5301333 | 4.5748487 | 2.7708645 | 2 | 32461682 | 32474581 |
| si:dkeyp-73d8.13 | 0 | 0 | 0 | 0 | 0 | 0 | 2 | 32476538 | 32478207 |
| bfsp2 | 0 | 0.5016664 | 0 | 0 | 0 | 0 | 2 | 32479005 | 32486826 |
| zgc:136930 | 0 | 0 | 0 | 0 | 0 | 0 | 2 | 32498013 | 32508762 |
| ccr9a | 0 | 0 | 0 | 0 | 0 | 0 | 2 | 32514748 | 32516958 |
| prpf4ba | 6.20445 | 9.4685815 | 12.008114 | 13.010873 | 10.522068 | 13.14016 | 2 | 32528356 | 32543983 |
| si:dkey-154p10.3 | 7.5391467 | 4.1416575 | 6.4196927 | 5.0955138 | 3.8520619 | 4.3362512 | 2 | 32544110 | 32552316 |
| pxdc1a | 0 | 0 | 0 | 0 | 0 | 0 | 2 | 32553506 | 32556769 |
| opn4.1 | 0.4395484 | 0 | 0 | 0 | 0 | 0 | 2 | 32561008 | 32564192 |
| tmem53 | 4.5578513 | 6.2052482 | 4.5560462 | 6.0271308 | 9.2093246 | 4.423807 | 2 | 32564477 | 32568496 |
| rnf220a | 20.52681 | 17.73317 | 19.599385 | 11.762348 | 11.545254 | 7.658621 | 2 | 32770235 | 32934615 |
| si:ch211-236l6.2 | 0 | 2.4698521 | 2.5522296 | 2.0257856 | 3.2020927 | 1.2390756 | 2 | 32990439 | 32999457 |
| klf4b | 26.419787 | 28.355422 | 36.488245 | 27.645429 | 18.727809 | 16.10417 | 2 | 33044397 | 33046563 |
| slc6a9 | 2.2183403 | 2.462108 | 3.4074472 | 2.0014033 | 2.778796 | 1.0587347 | 2 | 33064484 | 33140673 |
| ccdc24 | 0.1916443 | 0.5355573 | 0.2767099 | 0.6589003 | 0.1735837 | 0.8060352 | 2 | 33140286 | 33173149 |
| zgc:113531 | 0 | 0.778324 | 0 | 1.4363672 | 0 | 2.9285224 | 2 | 33175185 | 33184246 |
| b4galt2 | 0.841323 | 0.7837031 | 0.404921 | 0.3213987 | 0.5080243 | 0.3931682 | 2 | 33194963 | 33394369 |
| 5S_rRNA (2 332877 | 0 | 0 | 0 | 0 | 0 | 0 | 2 | 33287797 | 33287914 |
| atp6v0b | 22.954069 | 23.586336 | 21.184023 | 24.136845 | 21.433887 | 30.190214 | 2 | 33399057 | 33405089 |
| si:dkey-31m5.2 | 0 | 0 | 0 | 0 | 0 | 0 | 2 | 33410040 | 33413629 |
| si:dkey-31m5.3 | 0 | 0 | 0 | 0 | 0 | 0 | 2 | 33415215 | 33417186 |
| si:dkey-31m5.4 | 0 | 0 | 0 | 0 | 0 | 0 | 2 | 33440422 | 33443203 |
| si:dkey-31m5.5 | 0 | 0 | 0.5032028 | 0 | 0.3156656 | 0 | 2 | 33440493 | 33446769 |
| si:dkey-31m5.6 | 0 | 0 | 0 | 0 | 0 | 0 | 2 | 33465384 | 33468165 |
| si:dkey-31m5.7 | 0 | 0 | 0 | 0 | 0 | 0 | 2 | 33468967 | 33475440 |
| si:dkey-31m5.8 | 0 | 0 | 0 | 0 | 0 | 0 | 2 | 33474253 | 33475443 |
| kiss1ra | 0 | 0 | 0 | 0 | 0 | 0 | 2 | 33514082 | 33539327 |
| mir23a-3 | 0 | 0 | 0 | 0 | 0 | 0 | 2 | 33618310 | 33618387 |
| mir27a | 0 | 0 | 0 | 0 | 0 | 0 | 2 | 33620200 | 33620330 |
| BX664751.1 | 0 | 0 | 0 | 0 | 0 | 0 | 2 | 33623632 | 33623729 |
| kif2c | 30.895703 | 27.430688 | 28.810272 | 25.818289 | 22.882817 | 20.754943 | 2 | 33644786 | 33668083 |

| | | | | | | | | | |
|---|---|---|---|---|---|---|---|---|---|
| ptch2 | 15.101153 | 16.301846 | 22.211692 | 20.218033 | 16.745981 | 13.355709 | 2 | 33685224 | 33711408 |
| eif2b3 | 26.08624 | 23.808761 | 17.88146 | 29.292105 | 29.833162 | 28.444865 | 2 | 33726830 | 33789927 |
| klhl20 | 6.3082123 | 6.7361083 | 7.1088808 | 6.8768509 | 7.2466748 | 9.7067047 | 2 | 33829975 | 33844754 |
| cenpl | 3.5319118 | 4.9350309 | 6.3745375 | 2.78282 | 3.5989462 | 3.7137099 | 2 | 33846613 | 33856275 |
| dars2 | 3.0020619 | 2.0196646 | 4.3345944 | 2.4848092 | 2.2659556 | 2.8058552 | 2 | 33852924 | 33891364 |
| si:dkey-21n10.3 | 0 | 0.3508426 | 0 | 0 | 0 | 0 | 2 | 33935518 | 33937106 |
| fam5b | 0 | 0.1837253 | 0.2847796 | 0.2260386 | 0.1786459 | 0 | 2 | 34098857 | 34201502 |
| astn1 | 1.1805482 | 1.0996956 | 0.2435087 | 0.966403 | 1.1456711 | 2.0097474 | 2 | 34291015 | 34703231 |
| pappa2 | 0.2774811 | 0.5169543 | 0.600971 | 1.0335211 | 0.7539936 | 1.5560741 | 2 | 34707034 | 34823902 |
| rfwd2 | 7.2759636 | 8.4720653 | 13.609479 | 10.802276 | 10.933865 | 11.823468 | 2 | 34837460 | 34858050 |
| 5S_rRNA (2 349593 | 0 | 0 | 0 | 0 | 0 | 0 | 2 | 34959385 | 34959501 |
| tnr | 0 | 0.1719517 | 0 | 0.5288839 | 0 | 0.1293971 | 2 | 34961597 | 35194948 |
| 5S_rRNA (2 351387 | 0 | 0 | 0 | 0 | 0 | 0 | 2 | 35138792 | 35138906 |
| si:dkey-4i23.7 | 0 | 0.4216236 | 0 | 0.5187269 | 0 | 0 | 2 | 35202989 | 35217751 |
| tnw | 1.9288198 | 2.9196702 | 2.0887273 | 0.967102 | 0.5459523 | 1.1830595 | 2 | 35230251 | 35287808 |
| mrps14 | 32.050402 | 32.243783 | 31.468149 | 40.404406 | 29.029962 | 33.250797 | 2 | 35312256 | 35315017 |
| cacybp | 31.777734 | 33.656343 | 38.340583 | 36.917675 | 29.571565 | 32.955711 | 2 | 35316287 | 35322203 |
| plk3 | 16.959442 | 15.045652 | 20.017372 | 28.074689 | 28.527903 | 23.210419 | 2 | 35324470 | 35332623 |
| si:dkey-4i23.5 | 0.8158284 | 0 | 0 | 0 | 0 | 3.4312862 | 2 | 35333454 | 35335126 |
| si:ch73-27j14.1 | 0 | 0 | 0 | 1.6060282 | 0 | 0 | 2 | 35336228 | 35342700 |
| ankrd45 | 1.4158187 | 1.3188531 | 0.4088523 | 0.3245191 | 0.7694349 | 1.5879415 | 2 | 35336380 | 35452609 |
| 5S_rRNA (2 354073 | 0 | 0 | 0 | 0 | 0 | 0 | 2 | 35407362 | 35407476 |
| si:ch73-27j14.2 | 0.4584026 | 0.4270078 | 0.6618749 | 0 | 0 | 1.2853279 | 2 | 35449422 | 35454585 |
| rasal2 | 15.91265 | 17.852917 | 18.279118 | 18.23665 | 18.951949 | 17.378805 | 2 | 35454168 | 35572494 |
| 5S_rRNA (2 355521 | 0 | 0 | 0 | 0 | 0 | 0 | 2 | 35552162 | 35552278 |
| dhx9 | 39.495323 | 44.268814 | 49.698072 | 70.221894 | 68.621158 | 71.283721 | 2 | 35575075 | 35595362 |
| lamc1 | 41.468506 | 49.756264 | 58.447015 | 95.398549 | 79.80745 | 72.111652 | 2 | 35601433 | 35700011 |
| LAMC2 | 0.1189533 | 0.221613 | 0 | 1.090609 | 0.5387156 | 0.8338413 | 2 | 35714224 | 35742453 |
| nmnat2 | 0.5299873 | 1.4810697 | 0.7652341 | 0.3036953 | 0.4800412 | 1.4860463 | 2 | 35747016 | 35761653 |
| smg7 | 13.667357 | 13.902012 | 14.97056 | 24.935474 | 18.426721 | 16.408105 | 2 | 35767100 | 35799340 |
| CT867973.1 | 0 | 0 | 0 | 0 | 0 | 0 | 2 | 35872701 | 35876576 |
| CR352230.1 (2 3603 | 0 | 0 | 0 | 0 | 0 | 0 | 2 | 36037394 | 36037848 |
| CR352230.1 (2 3604 | 0 | 0 | 0 | 0 | 0 | 0 | 2 | 36045319 | 36045832 |
| CU571384.1 | 0 | 0 | 0 | 0 | 0 | 0 | 2 | 36059920 | 36060430 |
| CU571384.2 | 0 | 0 | 0 | 0 | 0 | 0 | 2 | 36070267 | 36070808 |
| CU693370.1 | 0 | 0 | 0 | 0 | 0 | 0 | 2 | 36120139 | 36120656 |
| BX901889.1 (2 3620 | 0 | 0 | 0 | 0 | 0 | 0 | 2 | 36203683 | 36204156 |
| BX901889.4 | 0 | 0 | 0 | 0 | 0 | 0 | 2 | 36210938 | 36211403 |
| BX901889.5 (2 362 | 0 | 0 | 0 | 0 | 0 | 0 | 2 | 36214682 | 36215141 |
| BX901889.1 (2 362 | 0 | 0 | 0 | 0 | 0 | 0 | 2 | 36217469 | 36217946 |
| BX901889.3 | 0 | 0 | 0 | 0 | 0 | 0 | 2 | 36220719 | 36221180 |
| BX901889.2 | 0 | 0 | 0 | 0 | 0 | 0 | 2 | 36229918 | 36230386 |
| BX901889.5 (2 363 | 0 | 0 | 0 | 0 | 0 | 0 | 2 | 36318046 | 36319659 |
| ing5b | 39.88861 | 35.159071 | 37.776751 | 47.188889 | 46.230214 | 40.288178 | 2 | 36326839 | 36329489 |
| pak2a | 40.207773 | 44.263877 | 48.756058 | 41.890919 | 40.359984 | 25.378634 | 2 | 36337663 | 36357699 |
| klhl6 | 0 | 0.1747162 | 0 | 0 | 0 | 0 | 2 | 36367284 | 36379989 |
| ccnl1b | 28.68973 | 20.23237 | 32.999025 | 44.211241 | 37.143831 | 31.132258 | 2 | 36385006 | 36394838 |
| ptx3b | 0 | 0 | 1.0185769 | 0.8084768 | 1.2779327 | 0 | 2 | 36406835 | 36415977 |
| golim4b | 1.3129329 | 1.3977298 | 1.6248922 | 4.0841402 | 1.8687454 | 0.2629549 | 2 | 36422155 | 36442122 |
| pdcd10b | 28.349874 | 33.010333 | 34.470401 | 33.77281 | 37.165777 | 26.148357 | 2 | 36442190 | 36452796 |
| dre-mir-2198 | 0 | 0 | 0 | 0 | 0 | 0 | 2 | 36474482 | 36474591 |
| slitrk3b | 0 | 0 | 0 | 0 | 0 | 0 | 2 | 36535521 | 36540457 |
| NRD1 (2 of 2) | 16.867007 | 19.92219 | 18.623498 | 20.46747 | 22.26714 | 20.246723 | 2 | 36549076 | 36575333 |
| FAM163A (2 of 2) | 0 | 0 | 0 | 0 | 0 | 0 | 2 | 36583306 | 36613716 |
| si:dkey-193b15.7 | 0 | 0 | 0 | 0 | 0 | 0 | 2 | 36615858 | 36619973 |
| si:dkey-193b15.6 | 7.8994695 | 11.719022 | 10.983388 | 9.3884686 | 7.4200218 | 12.305302 | 2 | 36619815 | 36626028 |
| si:dkey-193b15.5 | 0 | 0 | 0 | 0 | 0 | 0 | 2 | 36627969 | 36635114 |
| zgc:153654 | 0 | 0 | 0 | 0 | 0 | 0 | 2 | 36634957 | 36639542 |
| MAP1S (1 of 2) | 0 | 0.0976621 | 0 | 0.2403089 | 0.1899242 | 0.1469854 | 2 | 36641517 | 36653086 |
| gadd45bb | 4.7136752 | 6.4399107 | 7.2596753 | 13.325168 | 13.377632 | 16.741288 | 2 | 36660582 | 36662821 |
| gng7 | 2.4326004 | 7.1756607 | 2.9269705 | 2.7878753 | 5.5083785 | 3.4104182 | 2 | 36710857 | 36764738 |
| diras1b | 0 | 0 | 0 | 0 | 0 | 0 | 2 | 36780137 | 36781749 |
| zgc:101744 | 30.197618 | 26.254166 | 26.160904 | 56.756955 | 66.738354 | 67.737556 | 2 | 36810195 | 36824455 |
| slc1a8b | 0 | 0 | 0 | 0 | 0.1637645 | 0 | 2 | 36831337 | 36843995 |
| elavl1 | 119.97777 | 118.92338 | 117.14734 | 53.784608 | 51.27351 | 51.855312 | 2 | 36844171 | 36855031 |

| | | | | | | | | | |
|---|---|---|---|---|---|---|---|---|---|
| pex19 | 12.00328 | 18.243023 | 15.202812 | 19.548466 | 20.981221 | 24.208942 | 2 | 36855114 | 36867405 |
| si:dkey-52k20.13 | 2.3843627 | 2.8556538 | 3.4427155 | 1.5614815 | 1.542614 | 2.8652492 | 2 | 36857495 | 36861256 |
| copa | 67.202455 | 76.522164 | 76.38341 | 92.913545 | 91.839561 | 98.436274 | 2 | 36897647 | 36926913 |
| APOD (2 of 3) | 0 | 0 | 0 | 0 | 0.3818214 | 0 | 2 | 36928294 | 36936822 |
| apod | 0 | 0 | 0 | 0 | 0 | 0 | 2 | 36928632 | 36931230 |
| samd7 | 0 | 0 | 0.3090773 | 0.2453245 | 0.7755527 | 0.3001064 | 2 | 36947844 | 36963370 |
| sec62 | 25.883168 | 23.405514 | 26.881609 | 31.918444 | 33.452158 | 31.19432 | 2 | 36966215 | 36986736 |
| nadkb | 10.736185 | 8.1041716 | 9.8890038 | 18.456265 | 20.45478 | 17.387361 | 2 | 36988074 | 37002581 |
| gpr160 | 1.3059343 | 1.2164943 | 3.7712049 | 2.993325 | 1.5771502 | 1.8308729 | 2 | 37015921 | 37016992 |
| skila | 0.4615765 | 2.1498218 | 1.3329152 | 2.9094369 | 2.717503 | 1.6177841 | 2 | 37028246 | 37073405 |
| prkci | 29.520982 | 26.348576 | 27.643524 | 34.11556 | 33.67537 | 31.689899 | 2 | 37086384 | 37122433 |
| si:dkey-31b10.2 | 0 | 0 | 0.9092964 | 0 | 0 | 0 | 2 | 37126184 | 37129990 |
| phc3 | 0 | 0 | 0.4334439 | 0.1720191 | 0 | 0 | 2 | 37145094 | 37168609 |
| MUM1 | 2.2569624 | 4.3048919 | 4.1898417 | 4.6804886 | 4.0885311 | 2.1094534 | 2 | 37170127 | 37182556 |
| si:dkey-57k2.7 | 3.7299153 | 0 | 2.3935652 | 4.7496217 | 1.8768927 | 2.324092 | 2 | 37181473 | 37183295 |
| si:dkey-57k2.6 | 0 | 0 | 0 | 0 | 0 | 0 | 2 | 37184249 | 37186350 |
| dapk3 | 5.4373649 | 8.0443709 | 9.2363072 | 18.327875 | 13.90573 | 17.039625 | 2 | 37190409 | 37199251 |
| sppl2 | 7.2019535 | 5.1840038 | 7.090024 | 4.6896475 | 7.2645163 | 3.6715926 | 2 | 37201502 | 37223842 |
| GIN1 | 0 | 0 | 0 | 0 | 0 | 0 | 2 | 37206978 | 37210544 |
| arhgef18a | 6.377456 | 6.9649364 | 7.4618437 | 5.5446574 | 6.4735994 | 7.8618819 | 2 | 37225077 | 37258737 |
| 5S_rRNA (2 372291 | 0 | 0 | 0 | 0 | 0 | 0 | 2 | 37229108 | 37229223 |
| insra | 4.6372879 | 4.9842605 | 4.4294313 | 5.8868878 | 6.5911904 | 5.9011897 | 2 | 37265784 | 37353729 |
| hiat1a | 5.5853243 | 5.3328704 | 6.4515965 | 5.9209677 | 7.3354943 | 3.9152118 | 2 | 37376911 | 37405474 |
| myo9b | 2.1000644 | 2.0017305 | 2.9617082 | 3.5262027 | 3.8927796 | 4.0397365 | 2 | 37405755 | 37465784 |
| MYADM (1 of 2) | 0 | 0.4911796 | 0.7613431 | 0 | 0 | 0 | 2 | 37475050 | 37475934 |
| ftr66 | 0 | 0 | 0 | 0 | 0 | 0 | 2 | 37483787 | 37490982 |
| NFATC4 | 2.3350655 | 2.0078243 | 2.5934897 | 0.6175605 | 0.6507717 | 0.2518214 | 2 | 37496754 | 37517505 |
| si:dkeyp-66d1.2 | 0 | 0 | 0 | 0 | 0 | 0 | 2 | 37528039 | 37532476 |
| sdr39u1 | 7.4334241 | 9.4888949 | 2.7826078 | 4.4172883 | 5.9847964 | 3.4737977 | 2 | 37536607 | 37561848 |
| RNaseP_nuc (2 375 | 0 | 0 | 0 | 0 | 0 | 0 | 2 | 37551528 | 37551830 |
| mettl17 | 9.1112381 | 7.3802032 | 8.8656396 | 7.2639376 | 7.3555713 | 8.886002 | 2 | 37552359 | 37575883 |
| rpph1 | 0 | 0 | 0 | 0 | 0 | 4.3183624 | 2 | 37565147 | 37565449 |
| RNaseP_nuc (2 375 | 0 | 0 | 0 | 0 | 0 | 0 | 2 | 37575030 | 37575331 |
| parp2 | 32.837947 | 35.549336 | 32.548936 | 15.867241 | 17.202875 | 18.415048 | 2 | 37575232 | 37585260 |
| si:ch211-284o19.8 | 0 | 0 | 0 | 0 | 0 | 0 | 2 | 37589535 | 37603076 |
| zgc:66427 | 9.9080699 | 11.49904 | 15.009562 | 15.450405 | 12.652334 | 9.3364107 | 2 | 37605291 | 37613058 |
| cbln13 | 4.8864277 | 5.3103975 | 5.2915336 | 5.1334044 | 3.6882744 | 0 | 2 | 37613182 | 37616715 |
| hbl4 | 2.9375692 | 7.0364132 | 7.8770251 | 8.6569544 | 8.3622998 | 5.8833804 | 2 | 37618223 | 37621667 |
| hbl3 | 0.8871746 | 0.4132072 | 0 | 0.5083721 | 0.4017835 | 0 | 2 | 37625688 | 37627563 |
| hbl2 | 0 | 0.4911796 | 0.7613431 | 0.6043022 | 0 | 1.4784902 | 2 | 37631017 | 37632321 |
| hbl1 | 8.7011022 | 4.6924766 | 7.934704 | 7.3476974 | 5.8071319 | 3.8521996 | 2 | 37633260 | 37635376 |
| TEP1 | 5.2945284 | 10.137836 | 7.0075714 | 17.866242 | 20.114753 | 16.082599 | 2 | 37635490 | 37657771 |
| cbln10 | 8.0181074 | 4.4813811 | 2.3154248 | 7.3513045 | 5.8099827 | 2.2482196 | 2 | 37658833 | 37659476 |
| cbln14 | 0 | 0 | 0 | 0 | 0 | 0 | 2 | 37662058 | 37663345 |
| cbln9 | 1.140963 | 0 | 0 | 0 | 0 | 0 | 2 | 37666026 | 37670126 |
| apol1 | 0 | 0.3347662 | 0 | 0.4118655 | 0.4882667 | 0 | 2 | 37670743 | 37677483 |
| APOLD1 (3 of 4) | 0.8995737 | 0.6284732 | 0.9741522 | 0 | 0.2036994 | 0.3152925 | 2 | 37679785 | 37693261 |
| pcp4l1 | 1.8444816 | 1.1454387 | 0 | 0 | 2.2275428 | 0 | 2 | 37697938 | 37703032 |
| DCAF8 | 1.6774042 | 2.6042054 | 3.2292768 | 3.7166103 | 2.8360735 | 1.4109962 | 2 | 37704438 | 37725422 |
| hnrnpc | 100.49925 | 69.175697 | 76.443889 | 59.60203 | 65.777929 | 66.999652 | 2 | 37727933 | 37736951 |
| cbln5 | 0 | 0 | 0.6854411 | 2.7202818 | 1.289958 | 0 | 2 | 37736781 | 37738032 |
| casq1a | 0 | 0 | 0 | 0 | 0 | 0 | 2 | 37742615 | 37756145 |
| si:rp71-1g18.1 | 15.871263 | 11.538952 | 14.532148 | 11.756446 | 12.973058 | 12.482234 | 2 | 37758249 | 37768934 |
| tox4 | 11.954131 | 15.672079 | 13.85081 | 17.928395 | 20.719424 | 19.242115 | 2 | 37770447 | 37783417 |
| chd8 | 13.492262 | 14.541734 | 16.663589 | 13.737586 | 14.341703 | 13.913175 | 2 | 37790283 | 37818491 |
| cbln12 | 0.8430964 | 0.3926775 | 0 | 0 | 0 | 0.5909954 | 2 | 37824173 | 37828415 |
| trim110 | 0 | 0 | 0 | 0 | 0 | 0 | 2 | 37830744 | 37844755 |
| SALL2 | 0.9643269 | 0.5239982 | 1.0442737 | 1.2893583 | 0.3639368 | 0.3379879 | 2 | 37850519 | 37859580 |
| mmp14b | 5.3206676 | 6.1224503 | 7.6823651 | 5.1412292 | 3.968791 | 1.9014117 | 2 | 37864076 | 37886399 |
| acin1a | 26.429622 | 25.488452 | 45.254459 | 44.899875 | 35.542205 | 27.637462 | 2 | 37900578 | 37928232 |
| C2H14orf119 | 22.627889 | 13.821748 | 16.068091 | 29.758759 | 14.783589 | 23.402572 | 2 | 37928185 | 37932766 |
| si:ch211-14a17.10 | 0 | 0 | 0 | 0 | 0 | 0.5657814 | 2 | 37936319 | 37964030 |
| dhrs1 | 9.7582582 | 7.1420966 | 7.0448399 | 13.180466 | 7.2603089 | 6.8403634 | 2 | 37964506 | 37972433 |
| homez | 29.096981 | 30.389564 | 36.283374 | 51.535527 | 46.121026 | 51.300187 | 2 | 37974150 | 37978416 |
| C2H14orf164 | 0 | 0 | 0 | 1.2643201 | 0 | 0 | 2 | 37980033 | 37984837 |

| | | | | | | | | | |
|---|---|---|---|---|---|---|---|---|---|
| si:ch211-14a17.7 | 0 | 0.744339 | 0.5768738 | 2.2894153 | 1.8094017 | 2.2405202 | 2 | 37985405 | 37987509 |
| si:ch211-14a17.6 | 0 | 0.2959115 | 0 | 0.7281244 | 0 | 0.8907174 | 2 | 37988172 | 37990782 |
| ap1g2 | 5.8027554 | 6.451535 | 5.6757163 | 6.2211852 | 5.2559019 | 2.8867031 | 2 | 37992902 | 38014304 |
| SLC7A8 (2 of 2) | 0 | 0 | 0 | 1.4204712 | 0 | 0.4344169 | 2 | 38020758 | 38039853 |
| prmt5 | 28.339303 | 32.206071 | 37.372081 | 30.746013 | 27.89321 | 37.611712 | 2 | 38045045 | 38065775 |
| PSMB11 (1 of 2) | 0 | 0 | 0 | 0 | 0 | 0 | 2 | 38073355 | 38074482 |
| psmb5 | 146.63206 | 180.79015 | 163.29586 | 245.73337 | 257.22499 | 272.09645 | 2 | 38076030 | 38095333 |
| FP015916.1 | 0 | 0 | 0 | 0 | 0 | 0 | 2 | 38081665 | 38081773 |
| CDH24 (2 of 2) | 0 | 0 | 0.1327662 | 0.1053808 | 0.083286 | 0.1289127 | 2 | 38257744 | 38413397 |
| 5S_rRNA (2 383421 | 0 | 0 | 0 | 0 | 0 | 0 | 2 | 38342118 | 38342235 |
| gig2q | 0 | 0 | 0 | 0 | 0 | 0 | 2 | 38425302 | 38426037 |
| CABZ01011032.1 | 0 | 0 | 0 | 0 | 0 | 0 | 2 | 38435909 | 38442464 |
| LRRC16B | 0 | 0.0555804 | 0.0861512 | 0 | 0 | 0 | 2 | 38446369 | 38594873 |
| si:ch211-119o8.4 | 0 | 0 | 0 | 0 | 0 | 0 | 2 | 38596827 | 38613597 |
| rem2 | 0 | 0.2770516 | 0 | 0.3408588 | 0 | 0 | 2 | 38629171 | 38681494 |
| si:ch211-119o8.6 | 0 | 0.7361456 | 0 | 0 | 0 | 0.8309465 | 2 | 38689685 | 38696509 |
| copb2 | 68.651747 | 73.070875 | 67.314599 | 78.168083 | 85.462435 | 86.263767 | 2 | 38701553 | 38719326 |
| rbp2b | 2.9287898 | 7.5783467 | 6.5781315 | 3.3565318 | 5.6003128 | 4.1060579 | 2 | 38720262 | 38740708 |
| RBP1 | 0 | 0 | 0 | 0 | 0 | 0 | 2 | 38725478 | 38732248 |
| rbp1b | 0 | 0 | 0 | 0.552487 | 0 | 0.6758594 | 2 | 38732533 | 38740800 |
| si:ch211-119o8.8 | 0 | 0 | 0 | 0 | 1.5890084 | 0 | 2 | 38740928 | 38749816 |
| NMNAT3 | 0.7142661 | 0.8871305 | 1.3750788 | 0.8185828 | 2.5878137 | 1.3351671 | 2 | 38766950 | 38794482 |
| clstn2 | 0 | 0.2978376 | 0 | 0 | 0.4344052 | 0 | 2 | 38812535 | 39253455 |
| cbln7 | 1.0286271 | 0 | 0 | 0 | 0 | 0 | 2 | 39253572 | 39262839 |
| BX322588.1 | 0 | 0.6383171 | 0 | 0 | 0 | 0 | 2 | 39264719 | 39267386 |
| spsb4a | 16.311004 | 13.896866 | 15.221993 | 12.994042 | 13.873005 | 17.011145 | 2 | 39288262 | 39380025 |
| si:dkeyp-55c8.4 | 0 | 0.6062677 | 0.9397331 | 0 | 0 | 0 | 2 | 39314517 | 39316660 |
| zgc:136870 | 0.3220523 | 1.799975 | 1.3950075 | 1.4763489 | 0 | 1.8060232 | 2 | 39323147 | 39325299 |
| slc25a36a | 0 | 0.3455437 | 1.6068091 | 1.4879379 | 0.3359907 | 0.9101 | 2 | 39435480 | 39463255 |
| si:ch211-16k18.1 | 0 | 0 | 0 | 0 | 0 | 0 | 2 | 39631157 | 39730818 |
| 5S_rRNA (2 397610 | 0 | 0 | 0 | 0 | 0 | 0 | 2 | 39761095 | 39761212 |
| epha4a | 22.419818 | 22.382377 | 25.26878 | 31.00647 | 26.990273 | 22.546001 | 2 | 39772017 | 39840214 |
| si:ch211-122l24.4 (2 | 0 | 0 | 0 | 0 | 0 | 0 | 2 | 39903198 | 39903976 |
| si:ch211-122l24.6 | 0 | 0 | 0 | 0 | 0 | 0 | 2 | 39969460 | 39976808 |
| si:ch211-122l24.5 | 0 | 0 | 0 | 0 | 0 | 0 | 2 | 39979678 | 39982185 |
| si:ch211-122l24.4 (2 | 0 | 0 | 0 | 0 | 0 | 0 | 2 | 39984417 | 39985381 |
| si:ch211-122l24.3 | 0 | 0 | 0 | 0 | 0 | 0 | 2 | 39998824 | 39999929 |
| si:ch211-122l24.2 | 0 | 0 | 0 | 0 | 0 | 0 | 2 | 40009077 | 40009724 |
| epha4b | 2.8515359 | 4.2499869 | 6.175881 | 0.653599 | 1.8079644 | 0 | 2 | 40081937 | 40266727 |
| 5S_rRNA (2 403479 | 0 | 0 | 0 | 0 | 0 | 0 | 2 | 40347951 | 40348070 |
| uggt1 | 3.963651 | 4.1844833 | 5.9137732 | 4.9210761 | 4.2483031 | 3.8898273 | 2 | 40583387 | 40642628 |
| HS6ST1 (1 of 3) | 0 | 3.6224497 | 0 | 1.4855761 | 0 | 0 | 2 | 40654960 | 40655805 |
| si:dkeyp-82d1.2 | 0 | 0 | 0 | 0 | 0 | 0 | 2 | 40814872 | 40816884 |
| 5S_rRNA (2 408676 | 0 | 0 | 0 | 0 | 0 | 0 | 2 | 40867655 | 40867770 |
| hs6st1a | 0.6331803 | 2.3592617 | 4.5711575 | 3.6282728 | 1.1470183 | 0 | 2 | 40954856 | 40955592 |
| 5S_rRNA (2 410488 | 0 | 0 | 0 | 0 | 0 | 0 | 2 | 41048843 | 41048959 |
| 5S_rRNA (2 413062 | 0 | 0 | 0 | 0 | 0 | 0 | 2 | 41306249 | 41306365 |
| SSBP4 (2 of 2) | 3.2077222 | 2.273504 | 4.0274274 | 3.0368621 | 2.4632955 | 2.2485555 | 2 | 41487546 | 41530151 |
| znf622 | 28.24835 | 20.719446 | 22.802189 | 23.451993 | 30.018475 | 31.495435 | 2 | 41536204 | 41544585 |
| d2hgdh | 2.2912628 | 3.3200849 | 5.5138184 | 4.0847265 | 3.9200743 | 1.4276746 | 2 | 41544790 | 41561387 |
| acvr1l | 16.246309 | 19.11618 | 20.062419 | 23.886268 | 16.74828 | 23.076434 | 2 | 41563236 | 41583420 |
| otomp | 1.7974149 | 2.0928934 | 1.9464283 | 0 | 0 | 0 | 2 | 41589199 | 41603527 |
| efr3a (2 41686644..4 | 0.6714444 | 0.4690942 | 1.45422 | 1.9237676 | 0.7602091 | 1.8826817 | 2 | 41686645 | 41714376 |
| CR848728.2 | 17.83845 | 19.773923 | 22.408108 | 9.4041057 | 10.825424 | 5.2518616 | 2 | 41720884 | 41787554 |
| 5S_rRNA (2 417895 | 0 | 0 | 0 | 0 | 0 | 0 | 2 | 41789553 | 41789669 |
| 5S_rRNA (2 418354 | 0 | 0 | 0 | 0 | 0 | 0 | 2 | 41835429 | 41835546 |
| KCNQ3 (2 of 2) | 0 | 0 | 0 | 0 | 0 | 0 | 2 | 41838136 | 41842617 |
| CABZ01046038.1 | 0 | 0 | 0 | 0 | 0 | 0 | 2 | 41845658 | 41874524 |
| keap1a | 5.3694481 | 2.7282048 | 3.523999 | 3.5430058 | 4.5686763 | 4.5622866 | 2 | 41936143 | 41962158 |
| crlf1a | 2.9381909 | 4.0249442 | 4.7414754 | 1.9807682 | 1.7220143 | 3.1500054 | 2 | 41977713 | 42021750 |
| ODF3L2 (1 of 2) | 4.4137509 | 1.8688477 | 4.6348314 | 3.6788128 | 3.9977976 | 2.8126909 | 2 | 42027825 | 42035137 |
| ebi3 | 0.4504381 | 0 | 1.3007502 | 8.2595738 | 6.5278184 | 0.631498 | 2 | 42037721 | 42043784 |
| si:dkey-97a13.5 | 0 | 0 | 0 | 1.1354722 | 0 | 0 | 2 | 42047093 | 42048014 |
| HELZ2 (2 of 2) | 0.1086631 | 0 | 0.1568957 | 0.1245331 | 0.0984227 | 0.2285127 | 2 | 42050304 | 42076835 |
| gbp2 | 0.5794543 | 0.359846 | 1.3944301 | 6.1980991 | 4.0238218 | 4.0618702 | 2 | 42106335 | 42118400 |

| | | | | | | | | | |
|---|---|---|---|---|---|---|---|---|---|
| gbp1 | 10.246586 | 10.030155 | 9.5288304 | 12.340178 | 13.528157 | 11.200099 | 2 | 42122033 | 42136425 |
| si:dkey-97a13.9 | 4.6346751 | 3.3728582 | 4.6006671 | 4.6476125 | 3.4107953 | 2.6396694 | 2 | 42140154 | 42172676 |
| vcpip1 | 1.6494019 | 1.4182511 | 1.2823601 | 0.8724427 | 1.1492013 | 1.4230166 | 2 | 42173798 | 42184631 |
| si:dkey-97a13.12 | 0 | 0 | 0 | 0 | 0 | 0 | 2 | 42189574 | 42193044 |
| adhfe1 | 10.033169 | 7.6845088 | 5.7946465 | 8.4322238 | 4.8467414 | 9.3774281 | 2 | 42196605 | 42212732 |
| crha | 0 | 0 | 0 | 0 | 0 | 0 | 2 | 42213621 | 42215154 |
| trim55a | 3.0124368 | 1.7857147 | 1.5816634 | 0.9415623 | 1.4882966 | 1.9196945 | 2 | 42215428 | 42229832 |
| cyp7c1 | 2.4603894 | 2.8011914 | 3.5524883 | 3.7596303 | 6.437951 | 7.2820188 | 2 | 42236837 | 42253253 |
| slc39a6 | 17.642868 | 19.392774 | 17.577094 | 20.421757 | 15.980198 | 17.808959 | 2 | 42257404 | 42275102 |
| tmeff1a | 0 | 0.3027117 | 1.4076364 | 0.7448571 | 1.7660567 | 0 | 2 | 42278040 | 42288236 |
| murca | 2.8872628 | 1.3447609 | 1.0422098 | 0.4136175 | 0 | 0.5059798 | 2 | 42292710 | 42299718 |
| ift57 | 13.879328 | 11.49224 | 10.687989 | 7.7764461 | 10.895158 | 11.242584 | 2 | 42301508 | 42311644 |
| TMEM71 | 1.03242 | 1.6349109 | 1.639751 | 0.8282416 | 1.4961991 | 0.2894831 | 2 | 42314066 | 42328692 |
| apom | 75.093723 | 54.961306 | 57.310756 | 75.610702 | 71.903545 | 78.207032 | 2 | 42329859 | 42335679 |
| ftr02 | 1.0463091 | 0.8354144 | 0.8632782 | 0.5139085 | 0.1353864 | 0 | 2 | 42337236 | 42343872 |
| ftr03 | 0 | 0 | 0 | 0 | 0 | 0 | 2 | 42348696 | 42351372 |
| ftr04 | 0 | 0 | 0 | 0 | 0 | 0 | 2 | 42361094 | 42362671 |
| ftr05 | 0 | 0 | 0 | 0 | 0 | 0 | 2 | 42372948 | 42374493 |
| ftr06 | 0 | 0 | 0 | 0 | 0 | 0 | 2 | 42396062 | 42405281 |
| ftr07 | 0 | 0 | 0.4143841 | 0 | 0 | 0 | 2 | 42410874 | 42415336 |
| ftr08 | 0 | 0 | 0 | 0 | 0 | 0 | 2 | 42419130 | 42422784 |
| ftr09 | 0 | 0 | 0 | 0.6161376 | 0 | 0 | 2 | 42431946 | 42434347 |
| ftr10 | 0 | 0 | 0 | 0 | 0 | 0 | 2 | 42445422 | 42446689 |
| si:dkey-7l6.3 | 2.4537248 | 2.077887 | 3.8649442 | 1.5338645 | 2.4245291 | 0.9381911 | 2 | 42455761 | 42461532 |
| gtpbp4 | 142.93009 | 148.83744 | 128.23165 | 130.40758 | 136.82213 | 150.35661 | 2 | 42462491 | 42476393 |
| si:dkey-7l6.4 | 0.3021391 | 0.8443392 | 1.0906258 | 0.8656643 | 1.2314944 | 0.4235882 | 2 | 42486295 | 42517020 |
| esyt2a | 1.8802902 | 3.5030283 | 2.7148992 | 8.5216565 | 5.341513 | 4.9127304 | 2 | 42520959 | 42593563 |
| 5S_rRNA (2 425248 | 0 | 0 | 0 | 0 | 0 | 0 | 2 | 42524802 | 42524917 |
| si:dkey-7l6.7 | 0.9911232 | 0.5385589 | 0.4770185 | 0.8519056 | 0.2992398 | 0.9263461 | 2 | 42596779 | 42680096 |
| RNaseP_nuc (2 4268 | 0 | 0 | 0 | 0 | 0 | 0 | 2 | 42687856 | 42688153 |
| march11 | 0 | 0 | 0 | 0 | 0 | 0 | 2 | 42693690 | 42701231 |
| myo10 | 6.7726288 | 5.757536 | 6.7407343 | 4.5967665 | 4.4072202 | 4.148293 | 2 | 42705883 | 42806051 |
| ftr11 | 0 | 0 | 0 | 0 | 0 | 0 | 2 | 42809792 | 42816259 |
| ftr12 | 0 | 0 | 0 | 0 | 0 | 0 | 2 | 42817113 | 42819940 |
| basp1 | 1.9047096 | 0.3548522 | 1.100063 | 0.8731549 | 2.070251 | 2.1362674 | 2 | 42825522 | 42849775 |
| adcy8 | 0 | 0 | 0 | 0.3923752 | 0.1550536 | 0 | 2 | 42877342 | 42966373 |
| efr3a (2 42973772..4 | 0.5847793 | 1.9065525 | 1.0554333 | 0.8377309 | 1.1917563 | 1.0247993 | 2 | 42973773 | 43027438 |
| oc90 | 17.038072 | 17.130797 | 23.624579 | 10.383105 | 7.2262817 | 8.3414093 | 2 | 43043670 | 43061471 |
| 5S_rRNA (2 430633 | 0 | 0 | 0 | 0 | 0 | 0 | 2 | 43063346 | 43063462 |
| HHLA1 | 0 | 0 | 0 | 0 | 0 | 0 | 2 | 43081596 | 43082550 |
| 5S_rRNA (2 430999 | 0 | 0 | 0 | 0 | 0 | 0 | 2 | 43099920 | 43100037 |
| kcnq3 | 0 | 0 | 0 | 0.1452886 | 0 | 0 | 2 | 43102630 | 43184873 |
| lrrc6 | 1.0410571 | 1.2121973 | 2.2547304 | 0.2982752 | 1.1786844 | 0 | 2 | 43199355 | 43225067 |
| ftr13 | 0 | 0 | 0 | 0 | 0 | 0 | 2 | 43229480 | 43232048 |
| ftr14 | 0.1874112 | 0.5237277 | 0.5411957 | 1.5034746 | 0.339499 | 0.2627437 | 2 | 43232265 | 43236818 |
| ftr15 | 2.3885034 | 1.6686909 | 0.8621735 | 0.6843345 | 0.5408525 | 0 | 2 | 43245068 | 43252476 |
| CREM (1 of 2) | 0.2584863 | 0.4013054 | 0.9952564 | 1.5799333 | 0.6243371 | 1.3287575 | 2 | 43256633 | 43297713 |
| pard3 | 5.0894929 | 7.0296505 | 6.9684794 | 8.8497653 | 7.3121877 | 5.6590198 | 2 | 43306095 | 43490366 |
| 5S_rRNA (2 433235 | 0 | 0 | 0 | 0 | 0 | 0 | 2 | 43323563 | 43323677 |
| nrp1b | 4.3278381 | 4.1190759 | 4.4828678 | 2.91125 | 3.6643303 | 3.1656382 | 2 | 43570359 | 43633838 |
| itgb1b.2 | 0.8222975 | 0 | 0 | 0.235598 | 0.186201 | 0 | 2 | 43670284 | 43684517 |
| itgb1b.1 | 4.5750377 | 5.9008231 | 5.3354302 | 2.8232668 | 3.8251244 | 4.440488 | 2 | 43687135 | 43704034 |
| EPC1 (1 of 2) | 7.5327498 | 7.952432 | 7.2508863 | 6.0430215 | 4.0937166 | 5.1043113 | 2 | 43717977 | 43737245 |
| CU693495.1 | 0.3716877 | 0 | 0 | 0 | 0.1683298 | 0.2605464 | 2 | 43739588 | 43750519 |
| CABZ01071036.1 | 0 | 0 | 0 | 1.048642 | 1.6575539 | 0 | 2 | 43757513 | 43758022 |
| kif5ba | 37.16173 | 33.576231 | 39.143072 | 45.265466 | 48.558106 | 49.964186 | 2 | 43765423 | 43801060 |
| ftr16 | 0 | 0 | 0 | 0.3112965 | 0 | 0 | 2 | 43804075 | 43806647 |
| arhgap12a | 3.4226003 | 3.5180089 | 2.7265093 | 4.4634912 | 4.2759357 | 4.6329017 | 2 | 43810722 | 43858719 |
| zeb1a | 3.7017066 | 2.4471001 | 5.8620299 | 1.779042 | 2.8120733 | 0.8370418 | 2 | 43868731 | 43913416 |
| nlrb5 | 0 | 0 | 0 | 0 | 0 | 0 | 2 | 43926076 | 43934272 |
| gbp3 | 3.8519272 | 3.9867989 | 3.9137828 | 1.961996 | 2.842824 | 2.2001073 | 2 | 43955869 | 43980375 |
| si:ch211-195h23.3 | 4.5900379 | 5.4633668 | 8.4683815 | 0.2922445 | 0 | 1.0725113 | 2 | 43981962 | 44016911 |
| si:ch211-195h23.4 | 0 | 0 | 0 | 0 | 0 | 0 | 2 | 44002426 | 44004096 |
| kirrelb | 1.651872 | 1.2090098 | 1.36291 | 0.1352231 | 0.6412282 | 0 | 2 | 44023127 | 44100277 |
| cadm3 | 0.9024844 | 0.8406756 | 0.902127 | 1.2729721 | 1.2575907 | 0.5839618 | 2 | 44105656 | 44245114 |

| | | | | | | | | | |
|---|---|---|---|---|---|---|---|---|---|
| sdhc | 56.522995 | 58.970112 | 62.67801 | 63.223356 | 52.83453 | 57.055107 | 2 | 44254153 | 44261341 |
| atp1a2a | 3.702654 | 3.0040279 | 4.4838761 | 0.4106532 | 0.5409217 | 0 | 2 | 44264576 | 44317038 |
| mpz | 7.0906568 | 6.1922218 | 7.465211 | 0.5078893 | 0.8028039 | 0.8284038 | 2 | 44320983 | 44344410 |
| LIG1 | 44.555549 | 41.992341 | 58.845341 | 37.245784 | 34.065735 | 26.639603 | 2 | 44362777 | 44405822 |
| zgc:152670 | 0.3229438 | 0.6016526 | 0.2331448 | 0.5551634 | 0 | 0 | 2 | 44409991 | 44424444 |
| ETV3L | 0.5782576 | 0 | 0.8349301 | 0 | 0 | 0 | 2 | 44427193 | 44432915 |
| TMEM55B (1 of 2) | 2.2566615 | 0.8850984 | 2.4008757 | 2.1778871 | 1.6136787 | 1.8316495 | 2 | 44444676 | 44460112 |
| pih1d3 | 3.8393361 | 5.2020221 | 4.5356002 | 4.8000665 | 3.161378 | 5.382611 | 2 | 44462271 | 44467626 |
| KCNAB1 (1 of 3) | 1.6067767 | 0.8552759 | 2.3199805 | 1.0522526 | 2.4948917 | 2.252643 | 2 | 44488199 | 44572252 |
| pask | 2.9086825 | 5.4189492 | 4.382365 | 2.4638824 | 3.092753 | 2.1275834 | 2 | 44574233 | 44606763 |
| MTERFD2 | 12.467851 | 10.507869 | 12.001324 | 17.010414 | 8.6040965 | 16.647122 | 2 | 44612299 | 44618774 |
| BX323035.1 (2 4462 | 0 | 0 | 0 | 0 | 0 | 0 | 2 | 44623829 | 44625766 |
| 5S_rRNA (2 446258 | 0 | 0 | 0 | 0 | 0 | 0 | 2 | 44625879 | 44625995 |
| BX323035.1 (2 4462 | 0 | 0 | 0 | 0 | 0 | 0 | 2 | 44627715 | 44628827 |
| klhl24 | 1.030596 | 0.5760079 | 1.7856589 | 1.6535565 | 0.1866945 | 0.2889717 | 2 | 44632701 | 44665956 |
| YEATS2 | 4.3920362 | 3.5497501 | 4.569639 | 4.4413072 | 3.7441217 | 3.9842496 | 2 | 44677192 | 44756745 |
| MAP6D1 | 0 | 0 | 0 | 0 | 0 | 0 | 2 | 44759069 | 44782214 |
| acsm3 | 0.738766 | 1.3763397 | 1.0666838 | 1.8344317 | 1.6728611 | 2.0714466 | 2 | 44787051 | 44808224 |
| ncapd2 | 37.344653 | 49.449314 | 47.830673 | 40.794392 | 37.086672 | 47.307774 | 2 | 44811404 | 44839093 |
| snoU85 (2 44824582 | 0 | 0 | 0 | 0 | 0 | 0 | 2 | 44824583 | 44824876 |
| snoU85 (2 44835775 | 0 | 0 | 0 | 0 | 0 | 0 | 2 | 44835776 | 44836096 |
| si:ch211-224b1.4 | 6.3367157 | 5.4811073 | 13.070584 | 5.7059956 | 1.639869 | 6.3456052 | 2 | 44842735 | 44984354 |
| si:ch211-224b1.5 | 4.1480342 | 0.6439911 | 1.9964107 | 0.7923073 | 1.8785611 | 0 | 2 | 44985300 | 44986177 |
| camk2n1a | 1.5372127 | 1.4319331 | 0.634154 | 2.2650667 | 0.9945323 | 0.9236215 | 2 | 45004897 | 45007595 |
| mul1a | 0.9625036 | 0.5977229 | 1.3897324 | 0.6434603 | 0.9444467 | 0.899597 | 2 | 45022377 | 45033052 |
| alg3 | 9.6322929 | 8.9726028 | 13.31598 | 8.2205794 | 7.239514 | 7.4703686 | 2 | 45034221 | 45042845 |
| vwa5b2 | 0.2486169 | 0.1157949 | 0 | 0.5698534 | 0.2251871 | 0 | 2 | 45046044 | 45083205 |
| PRSS56 | 0 | 0 | 0 | 0 | 2.3560549 | 0 | 2 | 45110791 | 45119741 |
| si:dkey-76d14.2 | 0.6554127 | 0 | 0 | 0 | 0.5936464 | 2.7565951 | 2 | 45129867 | 45131943 |
| 5S_rRNA (2 451352 | 0 | 0 | 0 | 0 | 0 | 0 | 2 | 45135295 | 45135411 |
| chrng | 4.6004076 | 8.0350087 | 13.284803 | 5.272285 | 1.5625739 | 2.4186022 | 2 | 45143065 | 45158422 |
| eif4e2 | 5.4395234 | 6.9337688 | 6.6138759 | 13.452211 | 12.446908 | 12.041078 | 2 | 45178320 | 45197119 |
| capn10 | 2.847493 | 1.2482239 | 1.4510882 | 0.5758874 | 1.3654294 | 0.7044852 | 2 | 45200971 | 45216123 |
| CU929150.1 | 0 | 0 | 0 | 0 | 0 | 0 | 2 | 45221499 | 45222824 |
| zgc:195195 | 0 | 0 | 0 | 0 | 0 | 0 | 2 | 45241704 | 45243634 |
| mir181b-1 | 0 | 0 | 0 | 0 | 0 | 0 | 2 | 45252630 | 45252738 |
| CU571393.1 | 0 | 0 | 0 | 0 | 0 | 0 | 2 | 45255036 | 45255102 |
| CAMSAP2 (1 of 2) | 0.5523838 | 1.6465681 | 1.9141722 | 0.8862812 | 0.1000654 | 1.5488445 | 2 | 45346799 | 45431744 |
| 5S_rRNA (2 453859 | 0 | 0 | 0 | 0 | 0 | 0 | 2 | 45385914 | 45386046 |
| si:ch211-141h20.6 | 0 | 0 | 0 | 0 | 0 | 0 | 2 | 45387428 | 45392266 |
| wdr47a | 0.497423 | 0.1985811 | 1.3338285 | 0.6515089 | 0.6436367 | 0.1992483 | 2 | 45437773 | 45478472 |
| si:ch211-66k16.20 | 0.271074 | 0.3787633 | 0.1956981 | 0.4659954 | 0.8593476 | 1.1401079 | 2 | 45487179 | 45503631 |
| si:ch211-66k16.27 | 0 | 0 | 0 | 0 | 0 | 0 | 2 | 45496903 | 45503631 |
| si:ch211-66k16.2 | 0 | 0 | 0 | 0 | 0 | 0 | 2 | 45509755 | 45512232 |
| si:ch211-66k16.24 | 0 | 0 | 0 | 0 | 0 | 0 | 2 | 45516473 | 45517314 |
| si:ch211-66k16.28 | 0.1186207 | 0.1104967 | 0.1712732 | 0 | 0.1074419 | 0 | 2 | 45535181 | 45543193 |
| gpsm2 | 4.7350724 | 6.8483162 | 6.4770067 | 7.2830915 | 4.6274302 | 5.240843 | 2 | 45546372 | 45566044 |
| si:dkeyp-51f11.3 | 0 | 0 | 0.4291647 | 0 | 0 | 0.4167082 | 2 | 45566799 | 45593275 |
| fndc7 | 0 | 0 | 0 | 0 | 0 | 0 | 2 | 45604230 | 45623042 |
| FNDC7 (4 of 4) | 0 | 0 | 0 | 0 | 0 | 0 | 2 | 45649123 | 45653991 |
| FNDC7 (1 of 4) | 0 | 0 | 0 | 1.084802 | 0 | 0 | 2 | 45675289 | 45682103 |
| si:dkeyp-51f11.9 | 0 | 0 | 0 | 0 | 0 | 0 | 2 | 45691374 | 45694499 |
| si:dkeyp-51f11.8 | 0 | 0.3253697 | 1.0086656 | 0 | 0 | 0 | 2 | 45698462 | 45707715 |
| prpf38b | 35.576206 | 36.13636 | 34.427155 | 30.145267 | 31.537887 | 20.693466 | 2 | 45710634 | 45719285 |
| fam102ba | 1.5562539 | 1.0354787 | 2.2470321 | 1.0191661 | 0.6041109 | 0.6233748 | 2 | 45720833 | 45746468 |
| CU467828.1 | 0.2974212 | 0 | 0 | 0 | 0 | 0 | 2 | 45752083 | 45931456 |
| si:ch211-170p16.1 | 0 | 0 | 0 | 0 | 0 | 0 | 2 | 46050492 | 46053459 |
| itgb1b | 26.528126 | 41.840699 | 50.49062 | 58.732079 | 53.2441 | 47.757238 | 2 | 46095866 | 46102453 |
| 5S_rRNA (2 463194 | 0 | 0 | 0 | 0 | 0 | 0 | 2 | 46319414 | 46319550 |
| GPC6 (4 of 4) | 1.0301409 | 0 | 0 | 0 | 0 | 0 | 2 | 46421273 | 46423420 |
| gpc1b | 0.496969 | 0.4629329 | 3.5877988 | 2.8477498 | 0.4501344 | 0.6967326 | 2 | 46430883 | 46442745 |
| EPHB1 | 0 | 0.2934148 | 0 | 0.1804952 | 0 | 0 | 2 | 46498776 | 46805910 |
| CABZ01044741.1 | 0 | 0 | 0 | 0 | 0 | 0 | 2 | 46965059 | 46987692 |
| 5S_rRNA (2 470393 | 0 | 0 | 0 | 0 | 0 | 0 | 2 | 47039302 | 47039418 |
| si:ch211-226f6.1 | 0 | 0 | 0 | 0 | 0 | 0 | 2 | 47181435 | 47182794 |

| | | | | | | | | | |
|---|---|---|---|---|---|---|---|---|---|
| si:ch211-284d12.1 | 0 | 0 | 0 | 0 | 0 | 0 | 2 | 47255728 | 47256686 |
| si:ch211-284d12.2 | 0 | 0 | 0 | 0 | 0 | 0 | 2 | 47291965 | 47292601 |
| 5S_rRNA (2 473032 | 0 | 0 | 0 | 0 | 0 | 0 | 2 | 47303227 | 47303341 |
| si:ch211-284d12.3 | 0 | 0 | 0 | 0 | 0 | 0.6379638 | 2 | 47392211 | 47413529 |
| CU633981.2 | 0 | 0 | 0 | 0 | 0 | 1.204847 | 2 | 47419289 | 47422014 |
| CU633981.1 | 0 | 0 | 0 | 0 | 0 | 0 | 2 | 47425941 | 47432044 |
| wu:fb63a08 | 0 | 0 | 0 | 0.4818085 | 0 | 0.8840972 | 2 | 47429190 | 47435272 |
| CABZ01078499.2 | 0 | 0 | 0 | 0 | 0 | 0 | 2 | 47440933 | 47444779 |
| CABZ01078499.1 | 0 | 0 | 0 | 0 | 0 | 0 | 2 | 47441887 | 47444778 |
| si:dkey-20f20.6 | 0 | 0 | 0 | 0 | 0 | 0 | 2 | 47466663 | 47470565 |
| acsl3b | 15.982569 | 18.263725 | 19.454271 | 18.529767 | 13.298058 | 16.153874 | 2 | 47496912 | 47540359 |
| si:dkey-20f20.4 | 0.529086 | 0 | 0 | 0 | 0 | 0 | 2 | 47547466 | 47580215 |
| pax3a | 8.9525918 | 7.2970211 | 9.233143 | 8.0615025 | 8.3985002 | 6.7238633 | 2 | 47581097 | 47641979 |
| si:dkey-20f20.3 | 0 | 0 | 0 | 0 | 0 | 0 | 2 | 47663520 | 47667261 |
| BX085193.1 | 0 | 0 | 0 | 0 | 0 | 0 | 2 | 47682925 | 47685999 |
| scg2b | 0 | 0.3218763 | 0.2494589 | 0.5940104 | 0.1564888 | 0.2422184 | 2 | 47704725 | 47707925 |
| ap1s3b | 10.822214 | 17.33937 | 15.00086 | 19.844431 | 19.996742 | 21.241295 | 2 | 47709967 | 47743534 |
| CU467824.1 | 0 | 0.8889447 | 0.6889454 | 0 | 0 | 0 | 2 | 47745990 | 47756677 |
| cul3a | 37.597703 | 31.049569 | 32.617391 | 31.320813 | 30.90659 | 32.33509 | 2 | 47766773 | 47804182 |
| mbnl1 | 0 | 0.1640974 | 0.5087117 | 0.2018903 | 0 | 0 | 2 | 47877473 | 47943654 |
| ftr19 (2 47957087..4 | 0 | 0 | 0 | 0 | 0 | 0 | 2 | 47957088 | 47958388 |
| 5S_rRNA (2 479671 | 0 | 0 | 0 | 0 | 0 | 0 | 2 | 47967141 | 47967256 |
| CU929191.1 | 0 | 0 | 0 | 0 | 0 | 0 | 2 | 47967748 | 47968739 |
| CU929191.2 | 0 | 0 | 0 | 1.4732986 | 0.5821987 | 1.8022917 | 2 | 47983693 | 47984626 |
| ftr19 (2 47990291..4 | 0 | 0 | 0 | 0 | 0 | 0 | 2 | 47990292 | 47991634 |
| ftr22 | 0 | 0 | 0 | 0.2209948 | 0 | 0 | 2 | 48011265 | 48018262 |
| ftr23 | 1.6692705 | 1.2094029 | 1.3390076 | 1.0628128 | 0.6719813 | 0.7800857 | 2 | 48020412 | 48047591 |
| ftr24 | 1.4643949 | 6.1384609 | 4.9335986 | 6.7130637 | 7.5162093 | 5.8169154 | 2 | 48030376 | 48037975 |
| ftr26 (2 48041197..4 | 0 | 0 | 0 | 0.6295555 | 0 | 0 | 2 | 48041198 | 48047579 |
| ftr26 (2 48049647..4 | 0 | 0 | 0 | 0 | 0 | 0 | 2 | 48049648 | 48052583 |
| ftr26 (2 48056362..4 | 0 | 0 | 0 | 0 | 0 | 0 | 2 | 48056363 | 48057664 |
| ftr27 | 0.5732848 | 0 | 1.6555003 | 0 | 2.0770331 | 0 | 2 | 48059139 | 48060047 |
| fzd8b | 2.414047 | 1.729781 | 3.2174546 | 2.7666121 | 3.0275258 | 6.5084749 | 2 | 48069333 | 48071845 |
| BX548047.2 | 0 | 0 | 0 | 0 | 0 | 0 | 2 | 48096548 | 48098223 |
| si:ch211-77g15.32 | 0 | 0 | 0 | 0 | 0 | 0 | 2 | 48100834 | 48102714 |
| BX548047.1 | 0 | 0 | 0 | 0 | 0 | 0 | 2 | 48108982 | 48110499 |
| klf6b | 0 | 0 | 0.5926021 | 0 | 0 | 0 | 2 | 48188415 | 48196354 |
| pitrm1 | 12.74016 | 15.936518 | 15.767284 | 19.467768 | 17.474135 | 13.438461 | 2 | 48203229 | 48233581 |
| pfkpb | 0 | 0 | 0 | 0.5028276 | 0.1589606 | 0 | 2 | 48233666 | 48286440 |
| si:dkeyp-12a9.5 | 0 | 0.4730076 | 0 | 1.7458348 | 0 | 1.4237909 | 2 | 48296776 | 48310264 |
| gigfy2 | 15.104639 | 18.235671 | 21.378727 | 25.852062 | 22.141899 | 17.553922 | 2 | 48317450 | 48370015 |
| si:ch211-195j11.8 | 0 | 0 | 0 | 0 | 0 | 0 | 2 | 48377202 | 48379013 |
| 5S_rRNA (2 483910 | 0 | 0 | 0 | 0 | 0 | 0 | 2 | 48391058 | 48391172 |
| lpar5a | 0 | 0.2766989 | 0 | 0 | 0 | 0 | 2 | 48396762 | 48398332 |
| itm2cb | 27.005961 | 18.121132 | 22.470635 | 45.638297 | 40.837283 | 41.711695 | 2 | 48398577 | 48420960 |
| hes6 | 39.90093 | 51.977435 | 47.259722 | 56.088686 | 33.599514 | 36.273379 | 2 | 48402633 | 48418868 |
| per2 | 1.033182 | 1.4436331 | 1.3674677 | 2.4668238 | 2.2615518 | 0.9656559 | 2 | 48423772 | 48489514 |
| si:ch211-195j11.24 | 0 | 0 | 0 | 0 | 0 | 0 | 2 | 48519216 | 48520377 |
| si:ch211-195j11.27 | 0 | 0 | 0 | 0 | 0 | 0 | 2 | 48528118 | 48529568 |
| CR381673.2 | 0 | 0 | 0 | 0 | 0 | 0 | 2 | 48574935 | 48582050 |
| CR381673.1 (2 4859 | 0 | 0 | 0 | 0 | 0 | 0 | 2 | 48595934 | 48629072 |
| CR381673.1 (2 4871 | 0 | 0 | 0 | 0 | 0 | 0.6333319 | 2 | 48710962 | 48717469 |
| CR381673.3 | 0 | 0 | 0 | 1.2252174 | 0.4841652 | 0 | 2 | 48732060 | 48735452 |
| CR391991.2 | 0 | 0 | 0 | 0 | 0 | 0 | 2 | 48853842 | 48854551 |
| CR391991.1 | 0 | 0 | 1.7144748 | 0 | 0 | 0 | 2 | 48911934 | 48912326 |
| wu:fj40e01 | 0 | 0 | 0 | 2.9821974 | 0.7856436 | 1.2160444 | 2 | 48937401 | 48943912 |
| BX005223.4 | 0 | 0 | 0 | 0 | 0 | 0 | 2 | 48983386 | 48993667 |
| BX005223.3 | 0 | 0 | 0 | 0 | 0 | 0 | 2 | 48999689 | 49001537 |
| BX005223.5 (2 4900 | 0 | 0 | 0 | 0 | 0 | 0 | 2 | 49009151 | 49012479 |
| BX005223.2 | 0 | 0 | 0 | 0 | 0 | 0 | 2 | 49041004 | 49045324 |
| BX005223.1 | 0 | 0 | 0 | 0 | 0 | 0 | 2 | 49095879 | 49097256 |
| BX005223.5 (2 491 | 0 | 0 | 0 | 0 | 0 | 0 | 2 | 49119527 | 49186410 |
| zgc:123035 | 0 | 0 | 0 | 1.5637643 | 3.0897386 | 1.9129588 | 2 | 49286037 | 49286720 |
| CABZ01044732.1 | 0 | 0 | 0 | 0 | 0 | 0 | 2 | 49295576 | 49298552 |
| 5S_rRNA (2 493095 | 0 | 0 | 0 | 0 | 0 | 0 | 2 | 49309547 | 49309676 |

| | | | | | | | | | |
|---|---|---|---|---|---|---|---|---|---|
| svilb (2 49311117..4 | 6.5169309 | 5.9414419 | 7.1072636 | 13.745607 | 14.882524 | 9.5252899 | 2 | 49311118 | 49391635 |
| svilb (2 49462106..4 | 0 | 0 | 0.1449943 | 0 | 0 | 0 | 2 | 49462107 | 49467357 |
| KCNJ9 | 0 | 0 | 0 | 0.3537086 | 0 | 0 | 2 | 49512969 | 49528843 |
| cdc34b | 18.75803 | 13.015857 | 17.964011 | 22.15568 | 20.804409 | 24.151301 | 2 | 49569956 | 49594175 |
| ABHD17A (2 of 2) | 17.820438 | 13.2237 | 12.647165 | 13.153839 | 13.405266 | 20.749102 | 2 | 49601523 | 49626268 |
| SEMA6B (1 of 2) | 1.069081 | 1.4937937 | 1.5436165 | 0.8168116 | 0.3227768 | 0.7494065 | 2 | 49802577 | 49912504 |
| GPR35 (3 of 3) | 0 | 0 | 0 | 0 | 0 | 0 | 2 | 49936705 | 49937559 |
| rorcb | 0 | 0.408739 | 1.9006731 | 0.7543123 | 0.5961583 | 2.1530904 | 2 | 49987900 | 50017464 |
| sh3gl1a | 23.806391 | 17.65025 | 19.992687 | 21.43684 | 27.723689 | 27.586041 | 2 | 50027864 | 50081696 |
| stap2a | 3.4506809 | 2.4580346 | 1.465395 | 4.1872698 | 3.4931921 | 2.8457238 | 2 | 50092416 | 50139542 |
| si:ch211-209f23.3 | 0.4557166 | 0 | 0 | 0 | 0 | 0 | 2 | 50126006 | 50128170 |
| sema4e | 4.4811131 | 7.0529816 | 8.255026 | 4.0730365 | 4.1987706 | 1.7330646 | 2 | 50139218 | 50173919 |
| cactin | 23.755843 | 25.448199 | 24.867795 | 25.523739 | 34.6963 | 30.182509 | 2 | 50179034 | 50197341 |
| BX323861.1 | 0 | 0 | 0 | 0 | 0 | 0 | 2 | 50201281 | 50203389 |
| BX284666.1 | 0 | 0 | 0 | 0 | 0 | 0 | 2 | 50313783 | 50315978 |
| si:ch211-190k17.13 | 0 | 0 | 0 | 0 | 0 | 0 | 2 | 50337816 | 50339790 |
| si:ch211-190k17.17 | 0 | 0 | 0 | 0 | 0 | 0 | 2 | 50362746 | 50363555 |
| si:ch211-190k17.19 | 0 | 0 | 0 | 0 | 0 | 0 | 2 | 50369708 | 50371249 |
| wdr48 | 10.076665 | 10.5194 | 8.7798218 | 8.3625879 | 8.9696745 | 7.5507032 | 2 | 50376130 | 50404290 |
| blvra | 13.702723 | 8.154944 | 13.739572 | 7.8519847 | 8.6190098 | 4.8026812 | 2 | 50414868 | 50430961 |
| si:ch211-190k17.31 | 0.5690901 | 0 | 0 | 0 | 0.5154588 | 0 | 2 | 50425984 | 50427031 |
| rpl37 (2 50430959..5 | 5.1927357 | 5.4820456 | 3.4989023 | 3.1739312 | 4.3898126 | 1.9413409 | 2 | 50430960 | 50437026 |
| hecw1 | 0 | 0.2763471 | 0.1427821 | 0.2266613 | 0.1791381 | 0 | 2 | 50454934 | 50548465 |
| ccr12.3 | 0 | 0 | 0 | 0 | 0 | 0 | 2 | 50554397 | 50555589 |
| si:ch211-106n13.3 | 0 | 0 | 0 | 0.127822 | 0.2020441 | 0.1563652 | 2 | 50557326 | 50623244 |
| ZCCHC2 | 3.4881503 | 2.6841682 | 3.5036132 | 3.3023532 | 5.4946538 | 3.6145409 | 2 | 50632528 | 50670220 |
| exoc3 | 9.0037953 | 10.543845 | 11.143141 | 10.466187 | 7.805763 | 6.6721555 | 2 | 50675049 | 50696575 |
| ahrrb | 0 | 0 | 0 | 0 | 0 | 0 | 2 | 50703832 | 50768566 |
| mcm6l | 0.3610475 | 1.177121 | 0.5213065 | 1.0344437 | 1.7986223 | 2.5308778 | 2 | 50769976 | 50795733 |
| si:ch211-120f16.3 | 0 | 0 | 1.871635 | 0 | 0 | 0 | 2 | 50772876 | 50773533 |
| cul1a | 21.503047 | 26.824284 | 27.234786 | 26.805222 | 25.513198 | 26.796887 | 2 | 50799421 | 50842315 |
| cntnap2b | 0 | 0 | 0.3441208 | 1.0925585 | 0 | 0 | 2 | 50849183 | 50870460 |
| viml | 0 | 0.2621797 | 0 | 0.3225618 | 0 | 0 | 2 | 50896948 | 50915380 |
| CNTNAP2 (4 of 4) | 0 | 0.1140031 | 0.7068331 | 0.7012948 | 0 | 0 | 2 | 50964823 | 51035413 |
| CNTNAP2 (2 of 4) | 0 | 0 | 0 | 0 | 1.1007194 | 0 | 2 | 51134319 | 51138378 |
| CABZ01053212.1 | 0 | 0 | 0 | 0 | 0 | 0 | 2 | 51171499 | 51189739 |
| 5S_rRNA (2 511969 | 0 | 0 | 0 | 0 | 0 | 0 | 2 | 51196969 | 51197085 |
| neurod6b | 0 | 0.271514 | 0 | 0 | 0 | 0 | 2 | 51233146 | 51235788 |
| CABZ01053219.1 | 0 | 0 | 0 | 0 | 0 | 0 | 2 | 51304861 | 51308734 |
| CABZ01053221.1 | 0 | 0 | 0 | 0 | 0 | 0 | 2 | 51313236 | 51314231 |
| 5S_rRNA (2 513161 | 0 | 0 | 0 | 0 | 0 | 0 | 2 | 51316103 | 51316219 |
| XCR1 (4 of 4) | 0 | 0 | 0 | 0 | 0 | 0 | 2 | 51324849 | 51325907 |
| FYCO1 (1 of 2) | 0.1133756 | 0.6336647 | 0.6547994 | 0.9095364 | 1.2322923 | 0.7947423 | 2 | 51329179 | 51371813 |
| scn12aa (2 5147041 | 0 | 0 | 0 | 0.1734136 | 0 | 0 | 2 | 51470415 | 51506531 |
| ACVR2B (2 of 2) | 5.3844675 | 6.9662495 | 19.004294 | 17.141263 | 17.340564 | 13.000762 | 2 | 51541542 | 51569913 |
| 5S_rRNA (2 515447 | 0 | 0 | 0 | 0 | 0 | 0 | 2 | 51544760 | 51544891 |
| crygn1 | 0 | 0 | 0 | 2.0055278 | 2.6417265 | 0 | 2 | 51587030 | 51598133 |
| CABZ01040054.1 | 0 | 0 | 0 | 0 | 0 | 0 | 2 | 51627068 | 51627372 |
| CABZ01040055.1 | 0 | 0 | 0 | 0 | 0 | 0 | 2 | 51633350 | 51641977 |
| si:ch211-9d9.1 | 0.7055547 | 1.4459128 | 2.6487003 | 1.9406377 | 2.3006267 | 2.9674867 | 2 | 51648981 | 51671212 |
| tgm1l1 | 0.1095946 | 0.1020888 | 0 | 0 | 0.794131 | 0 | 2 | 51686652 | 51738436 |
| abhd4 | 1.1942517 | 0.5562303 | 1.2932603 | 2.3951707 | 0.8112788 | 0.8371489 | 2 | 51748981 | 51760936 |
| dad1 | 152.05575 | 144.57238 | 169.58275 | 210.31752 | 167.17083 | 132.31656 | 2 | 51762047 | 51765500 |
| pigr | 0 | 0 | 0 | 0 | 0 | 0 | 2 | 51771670 | 51792638 |
| si:ch211-9d9.8 | 0 | 0 | 0 | 0 | 0 | 0 | 2 | 51794747 | 51795507 |
| si:ch211-9d9.7 | 0 | 0 | 0 | 0 | 0 | 0 | 2 | 51812256 | 51813699 |
| CABZ01040076.1 | 0 | 0 | 0 | 0 | 0 | 0 | 2 | 51905766 | 51907213 |
| si:dkeyp-104b3.1 | 0 | 0 | 0 | 0 | 0 | 0 | 2 | 51949325 | 51956701 |
| si:dkeyp-104b3.2 | 0 | 0 | 0 | 0 | 0 | 0 | 2 | 51963213 | 51966978 |
| 5S_rRNA (2 519904 | 0 | 0 | 0 | 0 | 0 | 0 | 2 | 51990489 | 51990603 |
| si:dkeyp-104b3.18 | 0 | 0 | 0 | 0 | 0 | 0 | 2 | 52033367 | 52034083 |
| si:dkeyp-104b3.21 | 0 | 0 | 0 | 0 | 0 | 0 | 2 | 52087439 | 52096308 |
| si:ch211-215e19.2 | 0 | 0 | 0 | 0 | 0 | 0 | 2 | 52101894 | 52103230 |
| si:ch211-215e19.8 | 0 | 0 | 0 | 0 | 0 | 0 | 2 | 52114144 | 52117464 |
| si:ch211-215e19.3 | 0 | 0 | 0 | 0 | 0 | 0 | 2 | 52127474 | 52136302 |

| | | | | | | | | | |
|---|---|---|---|---|---|---|---|---|---|
| si:ch211-215e19.4 | 0 | 0 | 0 | 0 | 0 | 0 | 2 | 52142208 | 52144286 |
| si:ch211-215e19.6 | 0 | 0 | 0 | 0 | 0 | 0 | 2 | 52158710 | 52160911 |
| si:ch211-215e19.7 | 0 | 0 | 0 | 0 | 0 | 0 | 2 | 52196883 | 52197526 |
| lrrc24 | 0 | 0.0588219 | 0 | 0 | 0 | 0 | 2 | 52212554 | 52234826 |
| 5S_rRNA (2 522441 | 0 | 0 | 0 | 0 | 0 | 0 | 2 | 52244131 | 52244247 |
| 5S_rRNA (2 52267( | 0 | 0 | 0 | 0 | 0 | 0 | 2 | 52267095 | 52267209 |
| THEM6 (2 of 2) | 14.3724 | 17.571845 | 14.266939 | 26.251374 | 23.188206 | 25.816658 | 2 | 52308605 | 52319443 |
| THEM6 (1 of 2) | 0.7436715 | 1.0391091 | 0 | 1.2784241 | 0 | 0.5213003 | 2 | 52321434 | 52327464 |
| ftr67 | 17.032991 | 13.066485 | 11.211727 | 18.085275 | 17.015952 | 16.856216 | 2 | 52331004 | 52352304 |
| eef1da | 59.059578 | 55.7896 | 54.047215 | 52.114328 | 57.260953 | 64.140382 | 2 | 52386821 | 52418406 |
| BX255896.2 | 0 | 0 | 0 | 0 | 0 | 0 | 2 | 52443979 | 52445580 |
| BX255896.1 | 0 | 0 | 0 | 0 | 0 | 0 | 2 | 52446230 | 52447144 |
| ghrhra | 0 | 0 | 0 | 0 | 0 | 0 | 2 | 52451957 | 52511984 |
| adcyap1r1a | 0 | 0 | 0 | 0 | 0 | 0 | 2 | 52526137 | 52556894 |
| CU929148.1 | 0.3686049 | 0.3433602 | 0 | 0 | 0 | 0 | 2 | 52560070 | 52636980 |
| THEG | 0 | 0 | 0 | 0.4848662 | 0 | 0 | 2 | 52669981 | 52687014 |
| shda | 2.8877095 | 1.344969 | 1.6677936 | 1.3237807 | 1.0462283 | 0.8096929 | 2 | 52716172 | 52743793 |
| ccdc94 | 23.902327 | 15.190359 | 16.449602 | 23.041008 | 19.424088 | 23.801639 | 2 | 52746237 | 52760438 |
| scn12aa (2 5278962 | 0 | 0 | 0 | 0 | 0 | 0 | 2 | 52789628 | 52789894 |
| CABZ01040021.1 | 0 | 0.579592 | 1.7967696 | 0 | 1.1271366 | 0.8723092 | 2 | 52814258 | 52828309 |
| PPAP2C (1 of 2) | 0 | 0.503118 | 0.7798479 | 0.6189901 | 0 | 0.7572128 | 2 | 52861686 | 52935538 |
| TLE1 | 0.2439382 | 1.5906209 | 0.7044314 | 0.2795648 | 0.4418988 | 0 | 2 | 52954905 | 53014572 |
| chico | 0.3703602 | 5.8649186 | 7.4865401 | 10.186808 | 12.747379 | 8.3077067 | 2 | 53035303 | 53084360 |
| gna11b | 3.3615885 | 2.7834331 | 2.8987404 | 5.3507494 | 3.2139464 | 3.4037077 | 2 | 53090568 | 53178564 |
| C2CD4C (2 of 2) | 0.3677335 | 0.3425484 | 0 | 0 | 0 | 0 | 2 | 53375472 | 53376740 |
| RAB31 | 3.174516 | 0.7392755 | 2.291798 | 0 | 0.7188371 | 7.788475 | 2 | 53411307 | 53432629 |
| ralbp1 | 0.8539665 | 0.867797 | 1.4572038 | 2.0463434 | 2.5314165 | 1.3060693 | 2 | 53435983 | 53470581 |
| zgc:165603 | 0 | 0 | 0 | 0 | 0 | 0 | 2 | 53510777 | 53543519 |
| sirt6 | 15.147316 | 17.90874 | 16.403094 | 19.362565 | 27.175813 | 19.602454 | 2 | 53554287 | 53645654 |
| si:ch211-155i14.1 (2 | 0 | 0 | 0 | 0 | 0 | 0 | 2 | 53588514 | 53590833 |
| CR384061.1 | 0 | 0 | 0 | 0 | 0 | 0 | 2 | 53626191 | 53626272 |
| rxfp3.2a | 0 | 0 | 0 | 0 | 0 | 0 | 2 | 53781623 | 53782819 |
| 5S_rRNA (2 537952 | 0 | 0 | 0 | 0 | 0 | 0 | 2 | 53795271 | 53795385 |
| CREB3L3 (1 of 2) | 2.8435238 | 3.2373958 | 5.0180601 | 7.6038968 | 9.1575083 | 9.7448218 | 2 | 53806113 | 53854173 |
| CELF5 (1 of 2) | 0 | 0 | 0 | 0 | 0 | 0 | 2 | 53876129 | 53912269 |
| CABZ01117637.1 | 0 | 0 | 0 | 0 | 0 | 0 | 2 | 54003041 | 54008440 |
| HSD11B1L (2 of 2) | 0 | 1.0132727 | 0 | 2.4932746 | 3.9410372 | 4.5750482 | 2 | 54045004 | 54068709 |
| admp | 0 | 0.4676643 | 1.4497872 | 1.1507421 | 0.2273675 | 1.4077072 | 2 | 54071703 | 54087723 |
| im:7138239 | 0 | 0 | 0 | 0 | 0 | 0 | 2 | 54088548 | 54112518 |
| CU694222.2 | 0 | 0 | 0 | 0 | 0 | 0 | 2 | 54130893 | 54144217 |
| CU693369.1 | 0 | 0 | 0 | 0 | 0 | 0 | 2 | 54170996 | 54179310 |
| RANBP3 (1 of 2) | 63.522267 | 66.079009 | 66.736478 | 82.713857 | 60.67016 | 66.878579 | 2 | 54206171 | 54261877 |
| 5S_rRNA (2 542732 | 0 | 0 | 0 | 0 | 0 | 0 | 2 | 54273252 | 54273369 |
| ctnnbl1 | 57.348937 | 54.713711 | 49.749506 | 52.47367 | 54.248338 | 56.410481 | 2 | 54307209 | 54402862 |
| capsla | 6.3061331 | 4.0667835 | 2.1012119 | 0 | 1.3181172 | 0.6800747 | 2 | 54479382 | 54485838 |
| abhd8 | 0 | 0 | 0 | 0 | 0 | 0 | 2 | 54597840 | 54609170 |
| 5S_rRNA (2 546242 | 0 | 0 | 0 | 0 | 0 | 0 | 2 | 54624264 | 54624380 |
| CABZ01050249.1 | 0 | 3.2931361 | 8.7505015 | 2.3151836 | 0.9148836 | 0 | 2 | 54634186 | 54644845 |
| CU179656.1 | 0.1888675 | 0.0351865 | 0.0545401 | 0 | 0 | 0.0529571 | 2 | 54798988 | 54950572 |
| napg | 0.4974988 | 0 | 0.7183247 | 4.5612572 | 0 | 0 | 2 | 54956218 | 54979500 |
| rab12 | 6.3312972 | 5.8976835 | 9.1415853 | 5.5486802 | 8.7706164 | 12.009045 | 2 | 54979658 | 55010979 |
| SOGA2 | 0.3499466 | 0.1629899 | 0.7579174 | 0.6015831 | 0.3169676 | 0.2453063 | 2 | 55022580 | 55094860 |
| SOGA1 | 1.3115623 | 0.2443474 | 0.7574914 | 0.9018675 | 0 | 0.3677526 | 2 | 55139956 | 55151816 |
| CABZ01087853.1 | 0.6029119 | 0.8424302 | 1.7410558 | 1.3819313 | 2.1843733 | 4.2263043 | 2 | 55186164 | 55226184 |
| ndufv2 | 63.735383 | 82.085916 | 68.01904 | 92.098662 | 96.382231 | 91.685706 | 2 | 55227956 | 55277424 |
| ankrd12 | 2.4989114 | 3.2588746 | 4.329731 | 4.5821932 | 4.2681505 | 2.202127 | 2 | 55283371 | 55388809 |
| CU570683.1 | 2.1755424 | 7.0929086 | 6.2824113 | 12.466373 | 8.8673338 | 1.5250161 | 2 | 55398717 | 55415486 |
| twsg1a | 0.3658117 | 1.249447 | 0.1760618 | 1.1179669 | 1.3253501 | 2.2223712 | 2 | 55427608 | 55462812 |
| rnf126 | 0 | 0.8676526 | 2.6897749 | 2.1349597 | 3.3746606 | 3.9175563 | 2 | 55472895 | 55479329 |
| HCN2 (2 of 2) | 0 | 0 | 0 | 0 | 0.1553386 | 0 | 2 | 55503950 | 55663728 |
| CABZ01112877.1 | 0.9150075 | 3.4093645 | 0 | 0 | 0 | 1.2828076 | 2 | 55803426 | 55814829 |
| calrl | 67.04605 | 60.279221 | 57.634051 | 40.139226 | 51.26095 | 53.934772 | 2 | 55840231 | 55854933 |
| EPS15L1 (1 of 2) | 1.3826781 | 1.5026458 | 1.6636756 | 0.2641024 | 1.043645 | 1.9384649 | 2 | 55859792 | 55896981 |
| CABZ01118627.1 | 0 | 0 | 0 | 0 | 0 | 0 | 2 | 55936475 | 55962017 |
| rab8a | 2.2824332 | 2.5126819 | 10.785411 | 4.5181595 | 3.1949738 | 5.5270814 | 2 | 55967916 | 55993632 |

| | | | | | | | | | |
|---|---|---|---|---|---|---|---|---|---|
| tpm4b | 22.810524 | 18.432253 | 20.237467 | 24.567125 | 29.124335 | 24.273622 | 2 | 55995983 | 56012592 |
| rx2 | 1.7986658 | 2.0478088 | 4.6169669 | 1.3742374 | 2.172212 | 1.6811098 | 2 | 56045666 | 56074438 |
| cplx4c | 0 | 0.2343364 | 0 | 0 | 0 | 0 | 2 | 56106356 | 56216886 |
| atcayb | 0 | 0 | 0 | 0 | 0 | 0 | 2 | 56129980 | 56169710 |
| nmrk2 | 0.4668873 | 0.2174557 | 0 | 1.0701499 | 0.2114438 | 0 | 2 | 56196027 | 56203416 |
| loxl5b | 9.7473389 | 9.0797696 | 12.314674 | 14.801458 | 18.319649 | 15.715231 | 2 | 56225103 | 56254977 |
| jund | 0.467589 | 1.3066953 | 0 | 1.6076375 | 1.2705699 | 0.655543 | 2 | 56343402 | 56344399 |
| pgpep1 | 4.5638518 | 3.1884643 | 4.9422148 | 4.7945244 | 7.2340677 | 7.997945 | 2 | 56353059 | 56375691 |
| upf1 | 17.081161 | 17.93052 | 21.533192 | 24.246192 | 24.895646 | 19.084803 | 2 | 56382690 | 56448130 |
| uba52 | 3241.1744 | 3609.888 | 3124.423 | 3129.0546 | 3032.8725 | 3321.2821 | 2 | 56552145 | 56561814 |
| CERS4 (1 of 2) | 0 | 0 | 1.1146214 | 0 | 0 | 0 | 2 | 56569739 | 56598459 |
| CTXN1 | 0 | 0 | 0 | 0 | 0 | 0 | 2 | 56644028 | 56644297 |
| rab11bb (2 5667394 | 0 | 0 | 0 | 0 | 0 | 0.5544338 | 2 | 56673949 | 56688176 |
| ftr28l | 0 | 0 | 0 | 0 | 0 | 0 | 2 | 56697521 | 56704428 |
| si:ch211-117a23.3 | 0 | 0 | 0 | 0 | 0 | 0 | 2 | 56708560 | 56718784 |
| pip5k1cb | 0 | 0 | 0.334885 | 0 | 0 | 0 | 2 | 56725357 | 56798009 |
| si:ch1073-382c16.2 | 0 | 0 | 0 | 0 | 1.40424 | 0 | 2 | 56859963 | 56863678 |
| slc25a42 | 0.610005 | 0.4261706 | 0.6605771 | 1.1360288 | 1.3122302 | 0.5345032 | 2 | 56873542 | 56912362 |
| ARMC6 | 2.8924825 | 0.598752 | 0.9280835 | 0.7366493 | 1.746596 | 2.7034376 | 2 | 56921908 | 56945353 |
| eef2b | 1799.6928 | 2107.0052 | 1872.635 | 1809.886 | 1706.1561 | 1791.6066 | 2 | 56950887 | 56976839 |
| BTBD2 (1 of 2) | 2.3930967 | 3.2199553 | 3.8392514 | 2.4378685 | 1.445047 | 1.8639085 | 2 | 57008133 | 57029578 |
| CABZ01117751.1 | 0 | 0 | 0 | 0 | 0 | 0 | 2 | 57034232 | 57068290 |
| CABZ01117755.1 | 0 | 0 | 0 | 1.1247264 | 0.888909 | 0 | 2 | 57118915 | 57130311 |
| CABZ01117756.1 | 0 | 0 | 0 | 0 | 0 | 0 | 2 | 57136132 | 57140954 |
| rab11bb (2 5719002 | 2.3829133 | 1.4798093 | 1.7203114 | 0.4551552 | 1.4388978 | 1.1135862 | 2 | 57190023 | 57203845 |
| CU469531.1 | 0 | 0 | 0 | 0 | 0 | 0 | 2 | 57214524 | 57220517 |
| CU469531.2 | 0 | 0 | 0 | 0 | 0 | 0 | 2 | 57223990 | 57231924 |
| gpx4b | 201.78156 | 206.20099 | 167.2692 | 339.08535 | 351.24494 | 342.36611 | 2 | 57368919 | 57382529 |
| POLR2E (1 of 2) | 0 | 0 | 0 | 0 | 0 | 0 | 2 | 57385084 | 57398104 |
| HMHA1 (1 of 2) | 0 | 0 | 0 | 0.4853062 | 0.1278513 | 0 | 2 | 57410229 | 57483531 |
| GBGT1 (4 of 10) | 0 | 0 | 0 | 0 | 0 | 0 | 2 | 57491838 | 57501211 |
| CABZ01024428.1 | 0.57399 | 2.4060552 | 1.6575366 | 2.6312787 | 2.0795879 | 1.6094266 | 2 | 57537989 | 57551652 |
| ncana | 0.4741346 | 0.4416624 | 0.6845899 | 1.2075128 | 0.3817351 | 0.1477155 | 2 | 57606632 | 57707460 |
| si:ch1073-325m22.2 | 141.97141 | 117.29837 | 110.51559 | 94.793905 | 105.10997 | 152.30796 | 2 | 57733064 | 57733441 |
| mbd3a | 54.959773 | 56.597108 | 56.058048 | 78.877591 | 84.489578 | 72.810249 | 2 | 57737888 | 57762598 |
| tcf3a | 6.5725894 | 4.6291697 | 13.193387 | 8.4510961 | 7.4051832 | 5.3938734 | 2 | 57776875 | 57853745 |
| map2k2b | 1.2525218 | 2.3334799 | 2.3251908 | 2.6658345 | 1.7827602 | 1.0034232 | 2 | 57891760 | 57918126 |
| pias4b | 1.5624571 | 0.2425748 | 0.3759981 | 0.2984416 | 0.4717369 | 0 | 2 | 57922021 | 57948907 |
| ONECUT3 (1 of 2) | 0 | 0 | 0 | 0 | 0 | 0 | 2 | 57978989 | 58038804 |
| 5S_rRNA (2 580744 | 0 | 0 | 0 | 0 | 0 | 0 | 2 | 58074471 | 58074585 |
| LINGO3 (1 of 2) | 0 | 0 | 0 | 0 | 0 | 0 | 2 | 58084207 | 58099551 |
| ncstn | 4.899253 | 3.9932517 | 5.3054221 | 8.4221639 | 5.5469323 | 2.5757161 | 2 | 58109732 | 58124624 |
| pvrl4 (2 58153474..5 | 1.2114586 | 1.2789542 | 1.1661277 | 1.2032704 | 1.3167484 | 0.4529124 | 2 | 58153475 | 58191051 |
| CU984580.1 | 8.792029 | 6.9299039 | 8.7885471 | 5.4255824 | 1.2251485 | 3.7926487 | 2 | 58192923 | 58201113 |
| CU915267.1 (2 5822 | 0 | 0 | 0 | 0 | 0 | 0 | 2 | 58222375 | 58233774 |
| CU984580.4 (2 5825 | 0 | 0 | 0 | 0 | 0 | 0 | 2 | 58253742 | 58258254 |
| CR774186.2 | 3.8657715 | 2.0577229 | 1.5947659 | 0.6329082 | 2.5010429 | 1.5484779 | 2 | 58303299 | 58311045 |
| CR774186.1 | 0.3002921 | 0.5594517 | 0.8671668 | 0.6882978 | 0.5439849 | 0 | 2 | 58313646 | 58339136 |
| si:dkeyp-68b7.5 (2 5 | 0.2464504 | 0 | 0.1779215 | 0.4236658 | 0.1116124 | 0 | 2 | 58340428 | 58353694 |
| arhgap30 | 0 | 0 | 0 | 0 | 0 | 0 | 2 | 58361519 | 58399320 |
| si:ch211-155e24.5 | 0 | 0 | 0 | 0 | 0 | 0 | 2 | 58369801 | 58370736 |
| si:ch211-155e24.6 | 0 | 0 | 0 | 0 | 0 | 0 | 2 | 58378004 | 58382083 |
| si:ch211-155e24.3 | 3.8483977 | 3.7176027 | 4.3217962 | 4.7371456 | 4.0021269 | 2.5977443 | 2 | 58414089 | 58434168 |
| si:ch211-155e24.2 | 0.8562456 | 1.5952072 | 0 | 0 | 1.5511055 | 0 | 2 | 58431639 | 58433101 |
| pvrl4 (2 58439049..5 | 0 | 0.8781696 | 0 | 1.080419 | 1.2808371 | 0 | 2 | 58439050 | 58448431 |
| si:dkeyp-68b7.10 | 0 | 0 | 0 | 0 | 0 | 0 | 2 | 58620785 | 58637964 |
| 5S_rRNA (2 586233 | 0 | 0 | 0 | 0 | 0 | 0 | 2 | 58623333 | 58623447 |
| sf3a2 | 96.099281 | 80.838696 | 52.273389 | 128.74428 | 136.71274 | 162.71826 | 2 | 58644257 | 58656663 |
| plekhj1 | 0.6833843 | 0.7275213 | 1.2686396 | 1.5663815 | 0.530556 | 0 | 2 | 58667669 | 58690078 |
| TPGS1 (1 of 2) | 2.4668926 | 4.5958834 | 7.7174026 | 5.6543514 | 4.8412257 | 5.7641577 | 2 | 58698172 | 58710329 |
| si:dkeyp-68b7.7 | 1.2868772 | 1.498428 | 2.5548689 | 1.4748222 | 2.1855028 | 2.029675 | 2 | 58711491 | 58719262 |
| si:dkeyp-68b7.6 | 1.0159373 | 0.3154528 | 2.9337675 | 0.3881041 | 0.920195 | 1.8990766 | 2 | 58732315 | 58737139 |
| si:dkeyp-68b7.5 (2 5 | 0.8717846 | 1.3921344 | 0.8991041 | 1.5700244 | 0.7896274 | 1.2222106 | 2 | 58738265 | 58760688 |
| CU984580.4 (2 587 | 0 | 0 | 0 | 0 | 0 | 0 | 2 | 58773987 | 58790437 |
| CU915267.1 (2 5879 | 0 | 0 | 0 | 0 | 0 | 0 | 2 | 58791605 | 58794724 |

| | | | | | | | | | |
|---|---|---|---|---|---|---|---|---|---|
| KCNJ10 (2 of 2) | 0 | 0 | 0 | 0 | 0 | 0 | 2 | 58822161 | 58829594 |
| si:ch1073-185p12.3 | 0 | 0 | 0 | 0 | 0 | 0 | 2 | 58838190 | 58838833 |
| pnp5b | 1.6189206 | 1.8850562 | 1.753136 | 2.3191995 | 1.0997647 | 2.2696683 | 2 | 58850206 | 58864548 |
| si:ch1073-185p12.2 | 0 | 0 | 0.2941024 | 0.9337537 | 0.1844942 | 0.5711322 | 2 | 58868462 | 58904922 |
| CABZ01052486.1 | 4.7136752 | 6.8999043 | 5.8336676 | 6.1738229 | 3.6595346 | 3.7762303 | 2 | 58908628 | 58919029 |
| CABZ01081294.1 | 0 | 0 | 0 | 1.9066218 | 1.5068672 | 0 | 2 | 58958976 | 58966658 |
| epb41l3b | 17.077229 | 15.734746 | 15.008816 | 32.547945 | 24.210572 | 27.584957 | 2 | 58969930 | 59038058 |
| FOXL2 (2 of 2) | 1.0839811 | 2.0194842 | 0 | 0.6211468 | 0.9818263 | 0 | 2 | 59124201 | 59125061 |
| AL954696.1 | 19.026107 | 17.962561 | 20.046603 | 19.742202 | 21.89067 | 25.232084 | 2 | 59216223 | 59239826 |
| vapal | 42.986714 | 48.898259 | 47.147299 | 55.896611 | 62.521641 | 51.57364 | 2 | 59246438 | 59296669 |
| LRRC30 (1 of 2) | 0 | 0 | 0 | 0 | 0 | 0 | 2 | 59394955 | 59398361 |
| zgc:112425 | 1082.3593 | 919.87647 | 1042.1773 | 536.32244 | 590.01053 | 766.35946 | 2 | 59487737 | 59495440 |
| CABZ01088877.1 | 0 | 0 | 0 | 0 | 0 | 0 | 2 | 59505728 | 59505816 |
| fem1a | 1.0877712 | 3.7153331 | 4.7118085 | 2.4932746 | 2.9557779 | 3.0500322 | 2 | 59522037 | 59523994 |
| CABZ01085657.1 | 4.1480342 | 0.7727893 | 1.7967696 | 1.9015374 | 3.3814099 | 0 | 2 | 59530550 | 59532984 |
| CABZ01092973.1 | 0.831825 | 0.3874278 | 0 | 0.4766554 | 0.3767168 | 0 | 2 | 59550923 | 59562634 |
| sugp1 | 19.648583 | 23.086617 | 21.922309 | 34.033199 | 31.346803 | 26.607517 | 2 | 59583003 | 59597403 |
| mau2 | 14.336524 | 16.025584 | 26.910144 | 18.073369 | 18.179623 | 24.119148 | 2 | 59600501 | 59615379 |
| PRKAB2 | 5.1850428 | 2.1466369 | 1.6636756 | 1.3205121 | 3.6527576 | 0 | 2 | 59619446 | 59623198 |
| ndufa13 | 0 | 0 | 0 | 0 | 0 | 0 | 2 | 59673089 | 59676041 |
| GATAD2A | 0 | 0.1037208 | 0 | 0 | 0.1008533 | 0 | 2 | 59673268 | 59681384 |
| ptprsb | 0.6877728 | 0.3203345 | 0.6206601 | 0.4926376 | 0.6229569 | 0.2410582 | 2 | 59697319 | 59715893 |
| CABZ01100278.1 | 0 | 1.4205685 | 0 | 0 | 0 | 0 | 2 | 59810152 | 59822032 |
| si:ch1073-391i24.1 | 0 | 0.4819224 | 1.4939881 | 0 | 0 | 0 | 2 | 59848058 | 59864896 |
| 5S_rRNA (2 59870 | 0 | 0 | 0 | 0 | 0 | 0 | 2 | 59870375 | 59870492 |
| stk11 | 16.304242 | 12.109039 | 16.860622 | 26.260609 | 19.15813 | 25.0202 | 2 | 59873261 | 59895753 |
| C2H19orf26 (2 of 2 | 0 | 0 | 0.7107475 | 0 | 0 | 0 | 2 | 59908055 | 59915834 |
| CABZ01030469.1 | 0 | 0 | 0 | 0 | 0 | 0 | 2 | 59926861 | 59928467 |
| mir9-7 | 0 | 0 | 0 | 0 | 0 | 0 | 2 | 59942577 | 59942660 |
| ftr65 | 4.5416433 | 2.9085363 | 3.2787767 | 4.8796296 | 2.313921 | 1.5918051 | 2 | 59976754 | 59984723 |
| si:ch211-150h8.1 | 0 | 0 | 0 | 0 | 0 | 0 | 2 | 59989683 | 59990633 |
| si:ch211-150h8.2 | 0 | 0 | 0 | 0 | 0 | 0 | 2 | 59997251 | 59997921 |
| si:ch211-150h8.3 | 0 | 0.5519923 | 0 | 0 | 0 | 0.4153853 | 2 | 60000855 | 60004784 |
| ftr30 | 0 | 0 | 0 | 0 | 0.5415455 | 0 | 2 | 60016649 | 60031424 |
| ftr29 | 0.8888645 | 0 | 1.2834069 | 2.3769218 | 0 | 1.246156 | 2 | 60047931 | 60057000 |
| ftr37 | 0.1823579 | 0.3397374 | 0.2633015 | 0.6269723 | 0.1651724 | 1.0226368 | 2 | 60071068 | 60171903 |
| ftr32 | 1.0057195 | 0 | 0 | 0 | 0 | 0 | 2 | 60071223 | 60073794 |
| ftr33 | 0 | 0 | 0 | 0.6782592 | 0 | 0 | 2 | 60081176 | 60091504 |
| ftr34 | 0 | 0.2528761 | 0.3919655 | 0 | 0 | 0 | 2 | 60104663 | 60111068 |
| ftr35 | 0 | 0.2582852 | 0 | 0.3177703 | 0.2511445 | 0.3887296 | 2 | 60119016 | 60129389 |
| ftr36 | 0 | 0.25391 | 0.3935681 | 0.3123875 | 0 | 0 | 2 | 60138604 | 60153282 |
| si:dkey-264b2.2 | 0 | 0 | 0 | 0 | 0 | 0 | 2 | 60148113 | 60150671 |
| si:dkey-264b2.3 | 1.9203862 | 0 | 0.9242642 | 0 | 0 | 0 | 2 | 60161764 | 60162946 |
| ftr38 | 0.3277064 | 0 | 0 | 0.375567 | 0.2968232 | 0 | 2 | 60193412 | 60202382 |
| ftr39p | 1.6656294 | 1.8101474 | 0.8016521 | 1.5907418 | 1.50866 | 2.7243446 | 2 | 60205149 | 60216447 |
| BX537288.1 | 0 | 0 | 0 | 1.0674799 | 0.8436652 | 1.3058521 | 2 | 60234130 | 60235990 |

Zv9_scaffold3504

| | | | | | | | | | |
|---|---|---|---|---|---|---|---|---|---|
| PTCHD3 (1 of 3) | 0 | 0.9790405 | 0 | 0 | 0 | 0 | Zv9_scaffold | 554 | 997 |
| rab18b | 9.8242916 | 13.218764 | 12.214881 | 0 | 0 | 0 | Zv9_scaffold | 8949 | 22048 |
| mib | 18.719097 | 19.726793 | 23.205848 | 0 | 0 | 0 | Zv9_scaffold | 38978 | 65722 |
| yme1l1b | 6.1890431 | 7.686896 | 8.3404426 | 0 | 0 | 0 | Zv9_scaffold | 69385 | 82038 |

Zv9_scaffold3540

| | | | | | | | | | |
|---|---|---|---|---|---|---|---|---|---|
| WAC | 0 | 0 | 6.3564963 | 0 | 0 | 0 | Zv9_scaffold | 3299 | 7062 |
| cables1 | 1.0548233 | 0.786065 | 0.8122828 | 0 | 0 | 0 | Zv9_scaffold | 23857 | 79366 |
| gata6 | 10.104709 | 11.808616 | 9.815031 | 0 | 0 | 0 | Zv9_scaffold | 164734 | 175324 |
| MIB1 | 13.837895 | 13.666691 | 20.70233 | 0 | 0 | 0 | Zv9_scaffold | 221356 | 278892 |
| mir133a-1 | 0 | 0 | 0 | 0 | 0 | 0 | Zv9_scaffold | 268599 | 268697 |

Table S4. Summarized results of genomic PCR for the deleted genes in nn2002 allele.

| predicted position by synteny | Feature ID | AB - AB1_S1_L001_R1_001 (paired) RNA-Seq - Expression values | AB - AB2_S2_L001_R1_001 (paired) RNA-Seq - Expression values | AB - AB3_S3_L001_R1_001 (paired) RNA-Seq - Expression values | 1207 - 1207-1_S1_L001_R1_001 (paired) RNA-Seq - Expression values | 1207 - 1207-2_S2_L001_R1_001 (paired) RNA-Seq - Expression values | 1207 - 1207-3_S3_L001_R1_001 (paired) RNA-Seq - Expression values | contig | scaffold | LG | Chromosome region start | Chromosome region end | amplified in mibnn2002 or not (in black means results shown in Fig. 7; in red means results shown in supplementary Fig. 10) |
|---|---|---|---|---|---|---|---|---|---|---|---|---|---|
| | cldn1 | 4.42325923 | 8.9273643 | 13.837681 | 0 | 0 | 0 | | | LG2 | 88773 | 91785 | not amplified in nn2002 gPCR |
| | znfl2a | 69.331429 | 74.932960 | 57.753310 | 0 | 0 | 0 | | | LG2 | 161051 | 164976 | not amplified in nn2002 gPCR |
| | | | | | | | | | | | | | |
| | genes not shown | | | | | | | | | | | | |
| | | | | | | | | | | | | | |
| | foxc1a | 16.336109 | 14.216155 | 18.000801 | 0 | 0 | 0 | | | LG2 | 868215 | 870389 | not amplified in nn2002 gPCR |
| | | | | | | | | | | | | | |
| | genes not show | | | | | | | | | | | | |
| | | | | | | | | | | | | | |
| 1 | pth1ra | 0 | 0.6168789 | 0 | 0 | 0 | 0 | | | LG2 | 2092256 | 2240623 | not amplified in nn2002 gPCR |
| 2 | crhr2 | 0 | 0 | 0 | 0 | 0 | 0 | | | LG2 | 2681130 | 2785846 | not amplified in nn2002 gPCR |
| 3 | aqp1a.1 | 1.98407249 | 1.4785509 | 2.291798 | 0 | 0 | 0 | | | LG2 | 2888643 | 2900295 | not amplified in nn2002 gPCR |
| 4 | thoc1 | 61.949203 | 64.445908 | 62.678010 | 0 | 0 | 0 | | | LG2 | 2923616 | 2941632 | not amplified in nn2003 gPCR |
| 5 | usp14 | 56.795291 | 63.368194 | 61.503865 | 0 | 0 | 0.29710804 | | | LG2 | 2941647 | 2964335 | not amplified in nn2002 gPCR |
| 6 | rock1 | 13.554978 | 13.430877 | 14.585248 | 0 | 0 | 0 | | | LG2 | 2970496 | 3052905 | not amplified in nn2002 gPCR |
| 7 | wacb | 4.95123447 | 2.3060688 | 2.6808565 | 0 | 0 | 0 | cabz0165389, cabz0165390 | | LG2 | 3070270 | 3086434 | not amplified in nn2002 gPCR |
| 8 | mkxb | 0 | 0 | 0 | 0 | 0 | 0 | | Zv9_NA916 | no | (1686) | (9444) | not amplified in nn2002 gPCR |
| 9 | rab18b | 9.82429156 | 13.218764 | 12.214881 | 0 | 0 | 0 | cu694527 | scaffold 3504 | no | (8949) | (22048) | not amplified in nn2002 gPCR |
| 10 | PTCHD3 (3 of 3) | 0 | 0 | 0 | 0 | 0 | 0 | | scaffold 3504 | no | (61359675) | (61362524) | not amplified in nn2002 gPCR |
| 11 | yme1l1b | 6.18904309 | 7.686896 | 8.3404426 | 0 | 0 | 0 | cu681854 | scaffold 3504 | no | (69385) | (82038) | not amplified in nn2002 gPCR |
| 12 | mib | 18.719097 | 19.726793 | 23.205848 | 0 | 0 | 0 | cu681854, cabz01065400 | scaffold 3504 | no | (38978) | (65722) | not amplified in nn2002 gPCR |
| 12 | MIB1 | 13.837895 | 13.666691 | 20.702330 | 0 | 0 | 0 | cabz01065398, BX842700 | scaffold 3540 | no | (221356) | (278892) | not amplified in nn2002 gPCR |
| 13 | gata6 | 10.104709 | 11.808616 | 9.815031 | 0 | 0 | 0 | BX842700 | scaffold 3540 | no | (164734) | (175324) | not amplified in nn2002 gPCR |
| 14 | cables1 | 1.05482335 | 0.786065 | 0.8122828 | 0 | 0 | 0 | BX842700, cabz0165391 | scaffold 3540 | no | (23857) | (79366) | not amplified in nn2002 gPCR |
| 15 | npc1^ | 13.884070 | 19.095981 | 20.854080 | 0 | 0 | 0 | | | LG2 | 4310671 | 4353828 | not amplified in nn2002 gPCR |
| 16 | egfra^ | 0.35688593 | 0.6648875 | 1.0305955 | 0 | 0 | 0 | | | LG2 | 4190333 | 4307017 | not amplified in nn2002 gPCR |
| | | | | | | | | | | | | | |
| | genes not shown | | | | | | | | | | | | |
| | | | | | | | | | | | | | |
| | selt1a | 33.694727 | 30.712074 | 25.633262 | 0 | 0 | 0 | | | LG2 | 9473203 | 9485168 | not amplified in nn2002 gPCR |
| | dvl3a | 22.177302 | 22.825406 | 23.064216 | 0 | 0 | 0 | | | LG2 | 9490950 | 9532978 | not amplified in nn2002 gPCR |
| | ap2m1a | 107.013837 | 96.070221 | 90.231648 | 120.536461 | 119.681194 | 120.825322 | | | LG2 | 9578708 | 9607608 | amplifiable by gPCR |
| | | | | | | | | | | | | | |
| | genes not shown | | | | | | | | | | | | |
| | | | | | | | | | | | | | |
| 20 | anxa13l | 0.648130 | 0.8049888 | 0.9358175 | 1.2379801 | 0.97841723 | 0 | | | LG2 | 9610654 | 9631137 | amplifiable by gPCR |
| 19 | apbb1ip | 2.12954297 | 1.586957 | 2.2548448 | 0.65081522 | 2.18603472 | 0.99518086 | | | LG24* | (6123408) | (6161601) | amplifiable by gPCR |
| 18 | abi1b | 1.12497538 | 1.7028843 | 1.5228008 | 1.12811567 | 1.08264218 | 0.88716093 | | | LG2 | 9651729 | 9704711 | amplifiable by gPCR |
| 17 | acbd5b | 0 | 0 | 0 | 0 | 0 | 0 | | | LG2 | 9707575 | 9730294 | amplifiable by gPCR |
| | | | | | | | | | | | | | |
| | genes not shown | | | | | | | | | | | | |
| | | | | | | | | | | | | | |
| 21 | greb1l (2 1154822 | 5.16710144 | 11.732225 | 13.056112 | 9.99294113 | 14.0404565 | 18.1102256 | | | LG2 | 11548229 | 11568413 | amplifiable by gPCR |
| | greb1l (2 1155547 | 20.305225 | 20.669535 | 23.499861 | 27.4091044 | 21.9467422 | 19.0759553 | | | LG2 | 11555478 | 11624584 | amplifiable by gPCR |
| 22 | abhd3 | 0 | 0 | 0 | 0 | 0 | 0 | | | LG2 | 11646751 | 11703651 | amplifiable by gPCR |

^ The relative position of npc1 and egfra is reverse according to Zv9.

* The position of this gene is located on LG24 according to Zv9.

Table S5. DAVID functional annotation results.

| | | Count | P_Value | Bonferroni |
|---|---|---|---|---|
| Annotation Cluster 1 | Enrichment Score: 1.11 | Count | P_Value | Bonferroni |
| GOTERM_BP_FAT | somitogenesis | 3 | 2.80E-02 | 1.00E+00 |
| GOTERM_BP_FAT | segmentation | 3 | 3.30E-02 | 1.00E+00 |
| GOTERM_BP_FAT | anterior/posterior pattern formation | 3 | 7.00E-02 | 1.00E+00 |
| GOTERM_BP_FAT | embryonic development ending in birth or egg hatching | 3 | 8.60E-02 | 1.00E+00 |
| GOTERM_BP_FAT | chordate embryonic development | 3 | 8.60E-02 | 1.00E+00 |
| GOTERM_BP_FAT | regionalization | 3 | 1.50E-01 | 1.00E+00 |
| GOTERM_BP_FAT | pattern specification process | 3 | 2.30E-01 | 1.00E+00 |
| Annotation Cluster 2 | Enrichment Score: 1.03 | Count | P_Value | Bonferroni |
| KEGG_PATHWAY | Hedgehog signaling pathway | 3 | 3.50E-02 | 8.30E-01 |
| KEGG_PATHWAY | Melanogenesis | 3 | 1.20E-01 | 1.00E+00 |
| KEGG_PATHWAY | Wnt signaling pathway | 3 | 1.90E-01 | 1.00E+00 |
| Annotation Cluster 3 | Enrichment Score: 0.99 | Count | P_Value | Bonferroni |
| INTERPRO | Zinc finger, C2H2-type/integrase, DNA-binding | 5 | 4.90E-02 | 1.00E+00 |
| SMART | ZnF_C2H2 | 5 | 1.20E-01 | 9.90E-01 |
| INTERPRO | Zinc finger, C2H2-like | 5 | 1.30E-01 | 1.00E+00 |
| INTERPRO | Zinc finger, C2H2-type | 5 | 1.40E-01 | 1.00E+00 |
| Annotation Cluster 4 | Enrichment Score: 0.96 | Count | P_Value | Bonferroni |
| GOTERM_BP_FAT | protein amino acid phosphorylation | 5 | 8.80E-02 | 1.00E+00 |
| SP_PIR_KEYWORDS | kinase | 5 | 1.00E-01 | 1.00E+00 |
| GOTERM_MF_FAT | protein kinase activity | 5 | 1.10E-01 | 1.00E+00 |
| INTERPRO | Protein kinase, core | 5 | 1.10E-01 | 1.00E+00 |
| GOTERM_BP_FAT | phosphorylation | 5 | 1.30E-01 | 1.00E+00 |
| Annotation Cluster 5 | Enrichment Score: 0.85 | Count | P_Value | Bonferroni |
| INTERPRO | Serine/threonine protein kinase, active site | 4 | 1.20E-01 | 1.00E+00 |
| GOTERM_MF_FAT | protein serine/threonine kinase activity | 4 | 1.30E-01 | 1.00E+00 |
| INTERPRO | Serine/threonine protein kinase-related | 4 | 1.90E-01 | 1.00E+00 |
| Annotation Cluster 6 | Enrichment Score: 0.84 | Count | P_Value | Bonferroni |
| SMART | EGF | 3 | 1.20E-01 | 9.90E-01 |
| INTERPRO | EGF-like | 3 | 1.20E-01 | 1.00E+00 |
| INTERPRO | EGF-like region, conserved site | 3 | 2.10E-01 | 1.00E+00 |
| Annotation Cluster 7 | Enrichment Score: 0.74 | Count | P_Value | Bonferroni |
| GOTERM_MF_FAT | ribonucleotide binding | 9 | 1.70E-01 | 1.00E+00 |
| GOTERM_MF_FAT | purine ribonucleotide binding | 9 | 1.70E-01 | 1.00E+00 |
| GOTERM_MF_FAT | purine nucleotide binding | 9 | 2.00E-01 | 1.00E+00 |

Cluster 1
1517339 hypothetical protein LOC100148408
1537586 mind bomb
1526949 wingless-type MMTV integration site family, member 3 like

Cluster2
1546713 protein kinase, cAMP-dependent, catalytic, beta
1526949 wingless-type MMTV integration site family, member 3 like
1526580 wingless-type MMTV integration site family, member 9A

Cluster 3
1538660 si:ch211-241e1.5
1521138 zgc:113452
1533153 zgc:153115
1533539 zgc:171570
1539599 zinc finger-like gene 2a

Cluster 4
1521068 epidermal growth factor receptor
1518006 myosin light chain kinase family, member 4
1546713 protein kinase, cAMP-dependent, catalytic, beta
1519322 receptor (TNFRSF)-interacting serine-threonine kinase 1, like
1518369 receptor-interacting serine-threonine kinase 2

Cluster 5
1518006 myosin light chain kinase family, member 4
1546713 protein kinase, cAMP-dependent, catalytic, beta
1519322 receptor (TNFRSF)-interacting serine-threonine kinase 1, like
1518369 receptor-interacting serine-threonine kinase 2

Cluster 6
1525846 complement component 8, alpha polypeptide
1523183 complement component 8, beta polypeptide
1532880 protein C (inactivator of coagulation factors Va and VIIIa)

Cluster 7
1546722 ADP-ribosylation factor 1
1532072 RAB18B, member RAS oncogene family
1521068 epidermal growth factor receptor
1547472 eukaryotic translation initiation factor 4A, isoform 2
1518006 myosin light chain kinase family, member 4
1546713 protein kinase, cAMP-dependent, catalytic, beta

| Annotation Cluster 8 | | Enrichment Score: 0.41 | Count | P_Value | Bonferroni |
|---|---|---|---|---|---|
| | SP_PIR_KEYWORDS | gtp-binding | 3 | 3.00E-01 | 1.00E+00 |
| | GOTERM_MF_FAT | GTP binding | 3 | 4.30E-01 | 1.00E+00 |
| | GOTERM_MF_FAT | guanyl ribonucleotide binding | 3 | 4.30E-01 | 1.00E+00 |
| | GOTERM_MF_FAT | guanyl nucleotide binding | 3 | 4.30E-01 | 1.00E+00 |

| Annotation Cluster 9 | | Enrichment Score: 0.36 | Count | P_Value | Bonferroni |
|---|---|---|---|---|---|
| | GOTERM_MF_FAT | ATP binding | 6 | 4.00E-01 | 1.00E+00 |
| | GOTERM_MF_FAT | adenyl ribonucleotide binding | 6 | 4.10E-01 | 1.00E+00 |
| | GOTERM_MF_FAT | adenyl nucleotide binding | 6 | 4.50E-01 | 1.00E+00 |
| | GOTERM_MF_FAT | purine nucleoside binding | 6 | 4.50E-01 | 1.00E+00 |
| | GOTERM_MF_FAT | nucleoside binding | 6 | 4.50E-01 | 1.00E+00 |

| Annotation Cluster 10 | | Enrichment Score: 0.26 | Count | P_Value | Bonferroni |
|---|---|---|---|---|---|
| | GOTERM_MF_FAT | metal ion binding | 11 | 5.30E-01 | 1.00E+00 |
| | GOTERM_MF_FAT | cation binding | 11 | 5.50E-01 | 1.00E+00 |
| | GOTERM_MF_FAT | ion binding | 11 | 5.50E-01 | 1.00E+00 |

| | |
|---|---|
| 1519322 | receptor (TNFRSF)-interacting serine-threonine kinase 1, like |
| 1518369 | receptor-interacting serine-threonine kinase 2 |
| 1538421 | zgc:112335 |

Cluster 8

| | |
|---|---|
| 1546722 | ADP-ribosylation factor 1 |
| 1532072 | RAB18B, member RAS oncogene family |
| 1538421 | zgc:112335 |

Cluster 9

| | |
|---|---|
| 1521068 | epidermal growth factor receptor |
| 1547472 | eukaryotic translation initiation factor 4A, isoform 2 |
| 1518006 | myosin light chain kinase family, member 4 |
| 1546713 | protein kinase, cAMP-dependent, catalytic, beta |
| 1519322 | receptor (TNFRSF)-interacting serine-threonine kinase 1, like |
| 1518369 | receptor-interacting serine-threonine kinase 2 |

Cluster 10

| | |
|---|---|
| 1537586 | mind bomb |
| 1523243 | phosphotriesterase related |
| 1532880 | protein C (inactivator of coagulation factors Va and VIIIa) |
| 1516098 | ring finger and CCCH-type zinc finger domains 1 |
| 1538660 | si:ch211-241e1.5 |
| 1516089 | ubiquitin specific peptidase 33 |
| 1521138 | zgc:113452 |
| 1533153 | zgc:153115 |
| 1533539 | zgc:171570 |
| 1540346 | zgc:66286 |
| 1539599 | zinc finger-like gene 2a |

Table S6. List of primers used in this article.

| primers for probe synthesis | | |
|---|---|---|
| dystrophin (dmd) | sense | GACCACCCAAAGATGGCAGA |
| dystrophin (dmd) | T7 antisense | TAATACGACTCACTATAGGGCTCCTTGGCTGTGAGGGAAG |
| fgf8a | sense | GGACTTTACACATTTGAGCTGG |
| fgf8a | T7 antisense | TAATACGACTCACTATAGGAATCAAAGCAGAACGAGAGAGG |
| foxc1a | Sp6 sense | GATTTAGGTGACACTATAGCAGTCTTCTTGACGACTGTTCTTC |
| foxc1a | T7 antisense | TAATACGACTCACTATAGGTAATCGAAATACTGGTTTGGTC |
| p53 | Sp6 sense | GATTTAGGTGACACTATAGATGGCGCAAAACGACAGCCA |
| p53 | T7 antisense | TAATACGACTCACTATAGGGTTAATCAGAGTCGCTTCTTCC |
| papc | sense | GATCACTCGCCGCAATTCAC |
| papc | T7 antisense | TAATACGACTCACTATAGGGCAGCAACAGCACACAACCTC |
| tcf15 | sense | GCTAGTGCTTGACGACAA |
| tcf15 | T7 antisense | TAATACGACTCACTATAGGGCTCTCCCTGAGTGTCACT |
| **primers for real-time RT-PCR** | | |
| mib/herc2 | sense | TGACCACCGGCAGGAATAAC |
| mib/herc2 | antisense | TGATTCCTGTAGGCGCACTG |
| mib zinc finger | sense | CAGTGCGCCTACAGGAATCA |
| mib zinc finger | antisense | TTCGACTTGCGACGAGACTC |
| mib/her2 -2 | sense | GAGTCTCGTCGCAAGTCGAA |
| mib/her2 -2 | antisense | CCTCCTTTGGCATCTTGCAC |
| mib ank | sense | AGACGCAGGAGTCTGGAGAC |
| mib ank | antisense | CTTGTTGACAGCGATGTGCA |
| mib ring finger 2,3 | sense | GCAAGGAACAGGTTCAGTCC |
| mib ring finger 2,3 | antisense | GTAAAGCAGGATTCGGCGCT |
| **Primers for genomic PCR** | | |
| fih | sense | TGTAGGAGCCTGTTGTACTGA |
| fih | antisense | GAGTTTGTGGACAAGATGCA |
| vhl | sense | TAAGGGCTTAGCGCATGTTC |
| vhl | antisense | CGAGTTAAACGCGTAGATAG |
| mib exon1 GTP2F GTP4R | sense | TAGCCTAGACTCGCTAACTG |
| mib exon1 GTP2F GTP4R | antisense | GGTTCAGCCAGCTTTGAGAA |
| mib exon2 GTP5F GTP1R | sense | GGGACCTTTAAAGAGCCAAC |
| mib exon2 GTP5F GTP1R | antisense | GAGCAGACTTATCCACAAGG |
| mib exon8 and 9 | sense | GCATTCTTGCCCTTTCTGAG |
| mib exon8 and 9 | antisense | CTTGAAACATCCAGGCAGGT |

| | | |
|---|---|---|
| mib exon 20 and 21 | sense | TGGGGTAAGCGAAAACATTC |
| mib exon 20 and 21 | antisense | TCCCATCTGCACACTGTCAT |
| mib exon 22 (ta52b) | sense | GGTGTGTCTGGATCGTCTGA |
| mib exon 22 (ta52b) | antisense | GACAAGGAGAGCCTTGCAGA |
| nn2000 genotyping | sense | CTGATGAAAAAGTGTGTGCAGAG |
| nn2000 genotyping | antisense | AAGGCGGAGAGAGAGGAAAG |
| tfi91 genotyping (tfi91) | sense | GCTCGTAAATGACCACCGGC |
| tfi91 genotyping (tfi91) | antisense | CAACTGCAGTATTAGAAACGCG |
| foxc1a | sense | TATCAGTTCATCATGGAGCG |
| foxc1a | antisense | TCATGGCGTCCTTCTTTTTA |
| pth1ra | sense | TCCATGTTTATTTTCTACAG |
| pth1ra | antisense | CTGGACTGAGTTAAAGAG |
| crhr2 | sense | TTCTTGTGTTTGTGTGTTTG |
| crhr2 | antisense | CTGAAAAGACTGAAGAAAC |
| aqp1a.1 | sense | TGCTTTTATTTGTGTGCAG |
| aqp1a.1 | antisense | GGCTGTCAGATGTCC |
| thoc1 | sense | TTGCTGAAAGAAATCCCTC |
| thoc1 | antisense | CCTCTGTGTTCAGGATGTG |
| usp14 | sense | TGCATATGTCTGCGTC |
| usp14 | antisense | CCAGGCTTCTTATTCGG |
| rock1 | sense | CTTTATTTTATTTGTGTCTTCCAG |
| rock1 | antisense | CTGTGAATGAAGCCCATG |
| wacb | sense | GTTTGTGTTGTTTTGTTTTCTAG |
| wacb | antisense | GCCTTGAACATGCTTAATGA |
| mkxb | sense | ATGAACACGGCAGTC |
| mkxb | antisense | GATGGAGCTCAAAACGTAC |
| ptchd3ex | sense | TGGCTCTGAATGGCAGAAGG |
| ptchd3ex | antisense | CCCTCCAACACAGCAGAGAG |
| gata6 | sense | TTCTGCGTGTGTCAG |
| gata6 | antisense | ATGCGCTTCTGAGGTT |
| cables1 | sense | TCTGTCAATCTCTTTGCAG |
| cables1 | antisense | CTGTGTCAAGGCTACTC |
| npc1 | sense | TCTGATGGCACAGATTTAATAG |
| npc1 | antisense | GGAAAAAAGCACACACGA |
| egfra | sense | TGATCATGAAGTGTTGGTGCT |
| egfra | antisense | TGAGAAATGCCTTGCTCTGTT |

| | | |
|---|---|---|
| acbd5b | sense | GCATGGAAAGACTTGGGAAA |
| acbd5b | antisense | AATGGGTCAAGAGCATCCAA |
| apbb1ip | sense | ATTTGTGGTTTGTTGTGATAG |
| apbb1ip | antisense | CAGTCTGTAAGTCAGGGT |
| anxa13l | sense | GCAAAAAGTTCTGGGACTGC |
| anxa13l | antisense | TCCTCCAACTCCTCCAACAA |
| greb1la | sense | ATCATGGGAAATTCATACGC |
| greb1la | antisense | GGACAAATAGCCTTGCTTAC |
| greb1l1b | sense | TCAAGTCATCTGTGCACA |
| greb1l1b | antisense | GAAAATGTATTGGAACACCAAC |
| abhd3 | sense | CGAAACCCTGGGTCAACTAC |
| abhd3 | antisense | GCACGATCTGGCACTGTGTA |
| *cldn1* | sense | GTCTTTCGTGGGCATGAAGT |
| *cldn1* | antisense | GAGCGCTGTCATGTGTGTTT |
| *znfl2a* | sense | GGAGAATGAAGAGCCGAGTG |
| *znfl2a* | antisense | TGTGATCTCGAAGGCTTCCT |
| *selt1a* | sense | GGGCCTTATGTCTTCACAGC |
| *selt1a* | antisense | TGTGCTCCACTAAAGGCAGTT |
| *dvl3a* | sense | GTCACCCTTTTGGACCTGAA |
| *dvl3a* | antisense | GGAAGATCATGGCAGTCGAT |
| *ap2m1a* | sense | TCAACGCTGCTATGGTGTTC |
| *ap2m1a* | antisense | TCGCTGGGTCACTGGTATAA |
| *abi1b* | sense | CAAAGCCTACACCACCCAGT |
| *abi1b* | antisense | AATTTGCCCCTGCATTTTT |
| *rab18b* | sense | TCGTCATTTCACCTTTGCAC |
| *rab18b* | antisense | ATGGTTCTGGATGCTGCTTT |
| *mib* | sense | AACCGAGTGATGATGGAAGG |
| *mib* | antisense | TTAGCTCTGCAGCCTCACCT |
| *yme1l1b* | sense | CCAGCTGAGTCACCTCATCA |
| *yme1l1b* | antisense | AAGTGGCCGGAGAGTGTAAA |

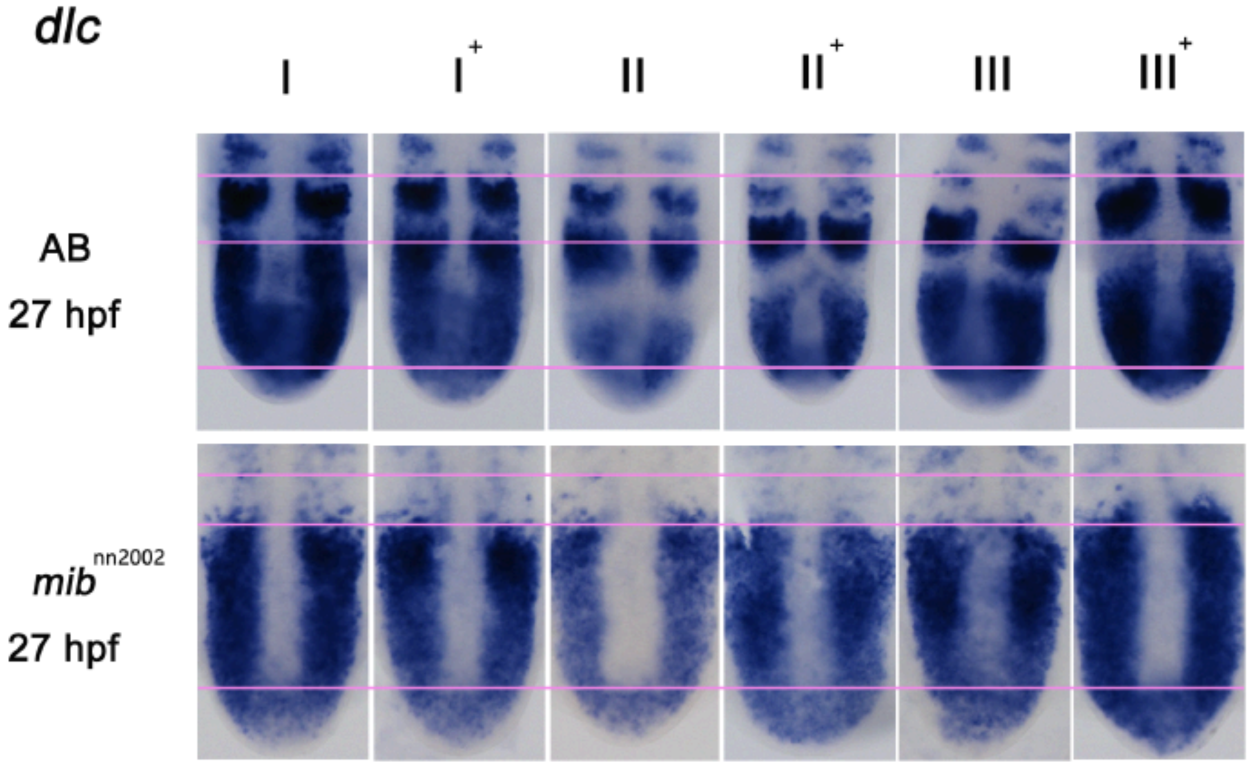


**Figure S1.** Cold-sensitive *dlc* cycling expression pattern in *mib*$^{nn2002}$ mutants.
Embryos were raised at 22 °C before the desired stage (siblings are with 16 somites or at 27 hpf). Typical salt-and-pepper pattern shown in previous Notch-related mutants was observed in *mib*$^{nn2002}$ mutants. Although *dlc* was still cyclically expressed in the PSM of *mib*$^{nn2002}$ mutants, the sharp-striped pattern of *dlc* was not maintained in the region where somites are about to form.

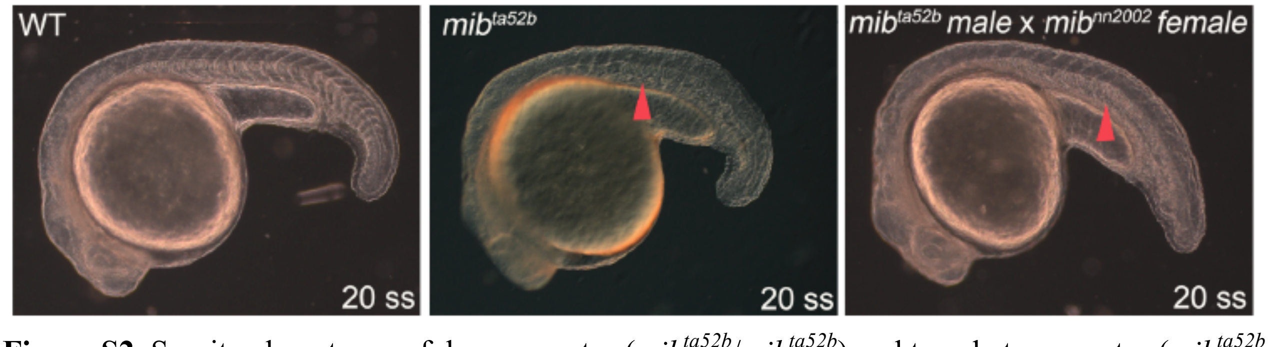


**Figure S2.** Somite phenotypes of homozygotes (*mib*$^{ta52b}$/*mib*$^{ta52b}$) and transheterozygotes (*mib*$^{ta52b}$/ *mib*$^{nn2002}$). Embryos are in lateral view with head to left. While 22 visible somites were observed in the WT embryos (left panel) at 22 ss, only 3 and 7 visible somites were observed in *mib*$^{ta52b}$ homozygotes (middle panel) and transheterozygotes (*mib*$^{ta52b}$♂ x *mib*$^{nn2002}$♀, right panel), respectively.

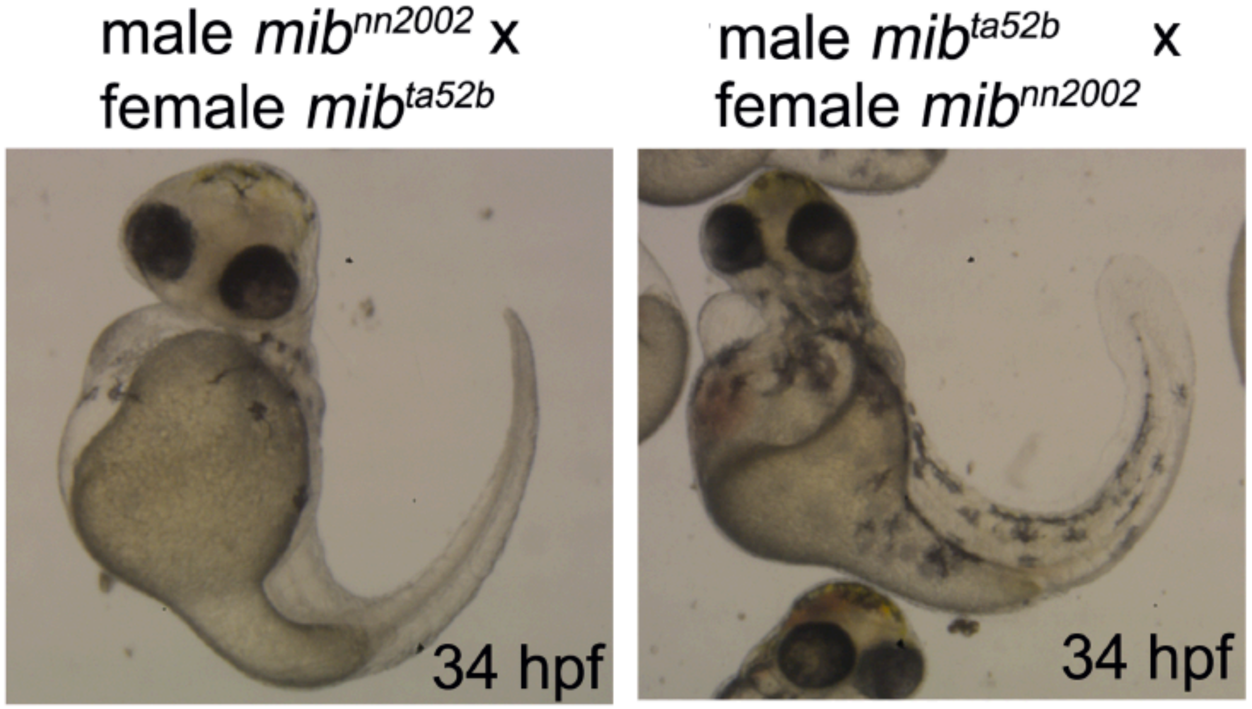


**Figure S3.** The maternal effect on phenotypes of transheterozygotes ($mib^{ta52b}/mib^{nn2002}$) at 34 hpf. The transheterozygotes have typical *mib* phenotype at 34 hpf, including less pigmentation and curly tail. Note that the transheterozygous embryos from incrosses of male $mib^{nn2002}$ carrier and female $mib^{ta52b}$ carrier (left) showed a more severe pigmentation phenotype (typical white tail phenotype of *ta52b* allele[17]) than those from incrosses of male $mib^{ta52b}$ carrier and female $mib^{nn2002}$ carrier (right).

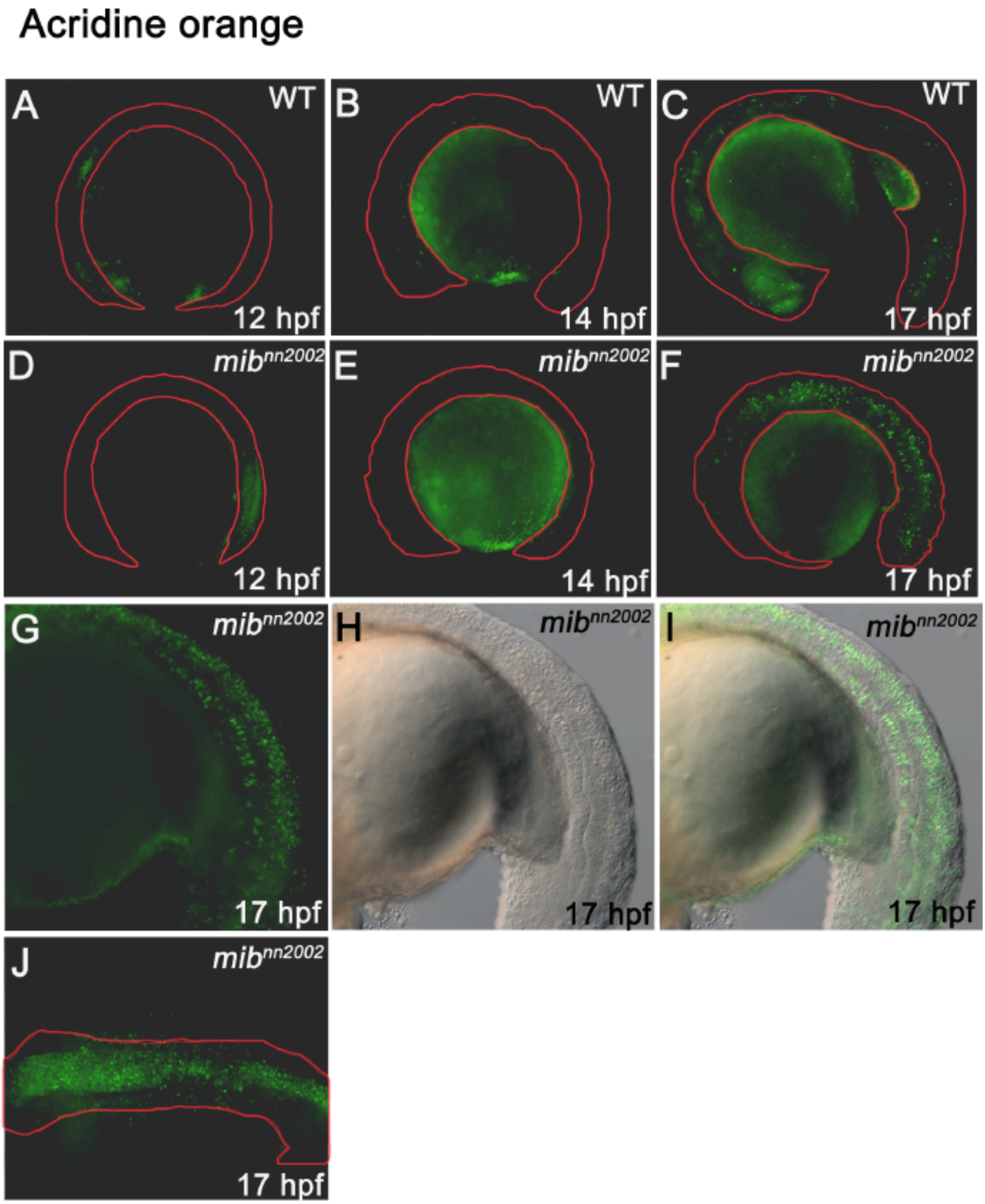


**Figure S4.** Cell death in *mib*$^{nn2002}$ mutants.
All the embryos are treated with acridine orange. A–C are wt embryos; D–J are *mib*$^{nn2002}$ homozygotes. A and D are at 12 hpf; B and E are at 14 hpf; C and F–J are at 17 hpf. A–I are lateral views with head toward left; J is dorsal view with head toward left. The contours of embryos are outlined by the red lines.
(F) The amount of cell death is highly increased in 17 hpf *mib*$^{nn2002}$ homozygotes. Cell death was mainly located around (G–I) neural tube and notochord.

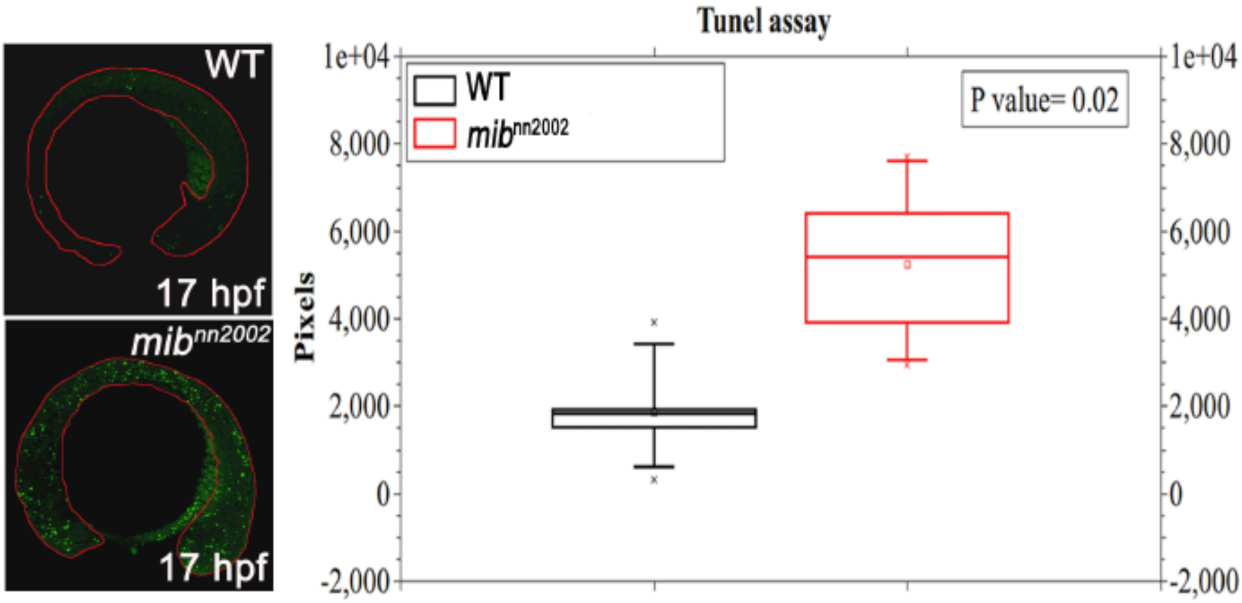


**Figure S5.** Apoptosis is highly increased in *mib*$^{nn2002}$ mutants.
TUNEL assay was employed to study the apoptosis in wt embryos and *mib*$^{nn2002}$ homozygotes. The lateral view of embryos is shown with head to the left. Apoptosis is increased in *mib*$^{nn2002}$ homozygotes at 17 hpf. Analyzed and calculated by Image J, the fluorescent signal increases by 2~3-fold in *mib*$^{nn2002}$ homozygous embryos.

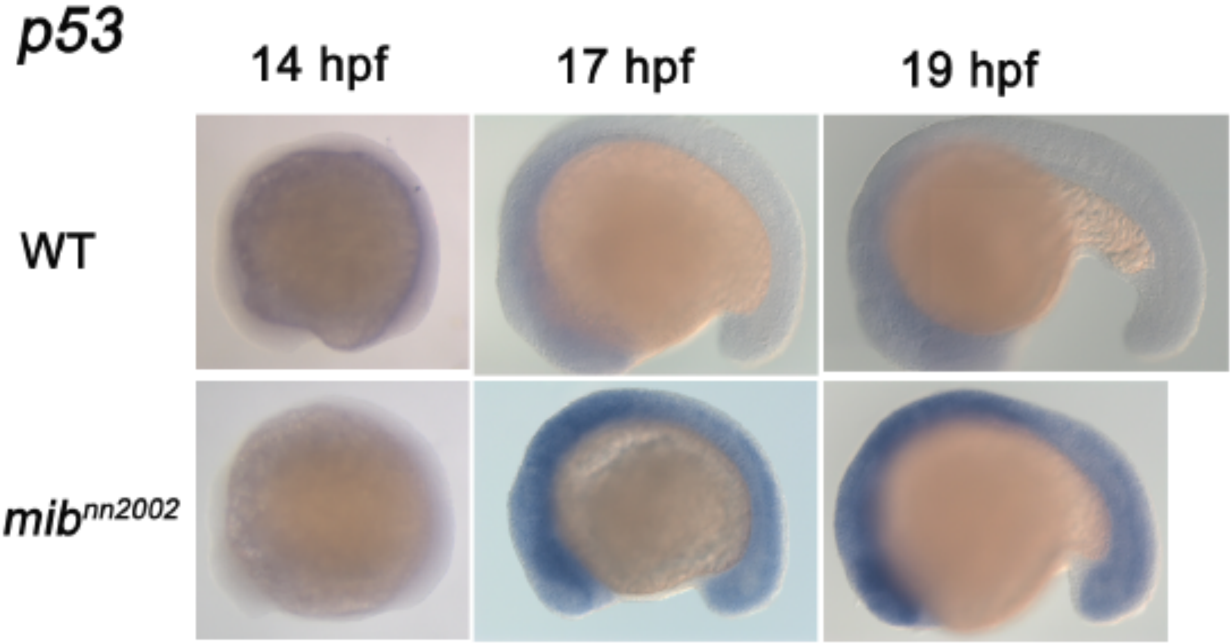


**Figure S6.** *p53* is highly up-regulated in *mib*$^{nn2002}$ mutants.
*p53* WISH was performed in the wt embryos and *mib*$^{nn2002}$ homozygotes. Embryos are shown in lateral views with head to the left. The staining of *p53* was highly up-regulated in *mib*$^{nn2002}$ mutants compared to wt embryos.

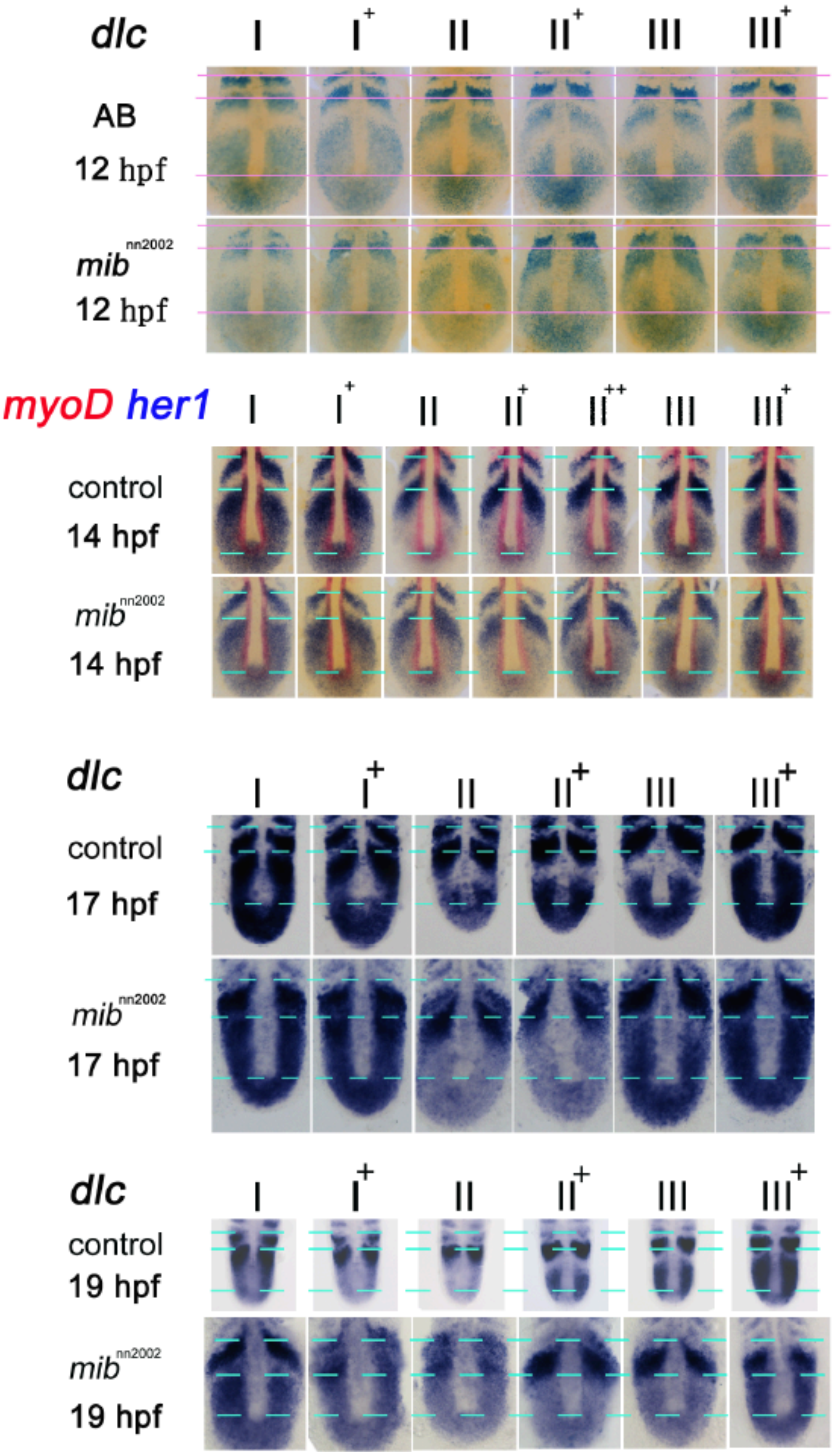


**Figure S7.** The expression of *dlc* in *mib*$^{nn2002}$ mutants during the stages of 12 hpf, 14 hpf, 17 hpf and 19 hpf at 28.5 °C.
*dlc* WISH was performed in the wt embryos and *mib*$^{nn2002}$ homozygotes. Embryos are flat-mounted with head to the top. The cyclically *dlc* expression was detected in both wt embryos and mutants. No salt-and-pepper pattern was observed.

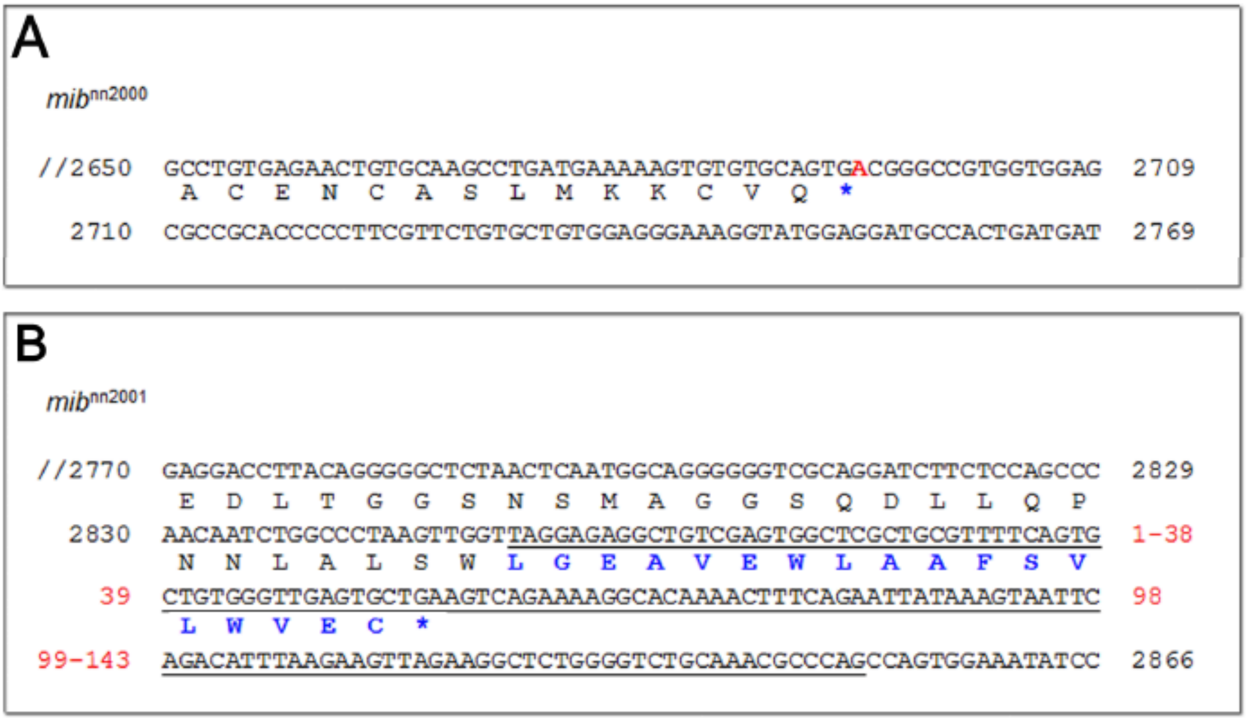


**Figure S8.** The mutations of *mib*$^{nn2000}$ *and mib*$^{nn2001}$ alleles.
This panel shows the mutations of *mib*$^{nn2000}$ and *mib*$^{nn2001}$ in nucleotide sequences and amino acids. *mib*$^{nn2000}$ mutation is a nonsense point mutation that leads to a truncated protein without ring finger domain 3 (RF3). *mib*$^{nn2001}$ is a nonsense mutation caused by a failure to splice a 143 bp long intron (the underlined nucleotides) out from *mib* mRNA. The stop coden is marked by blue asterisks.

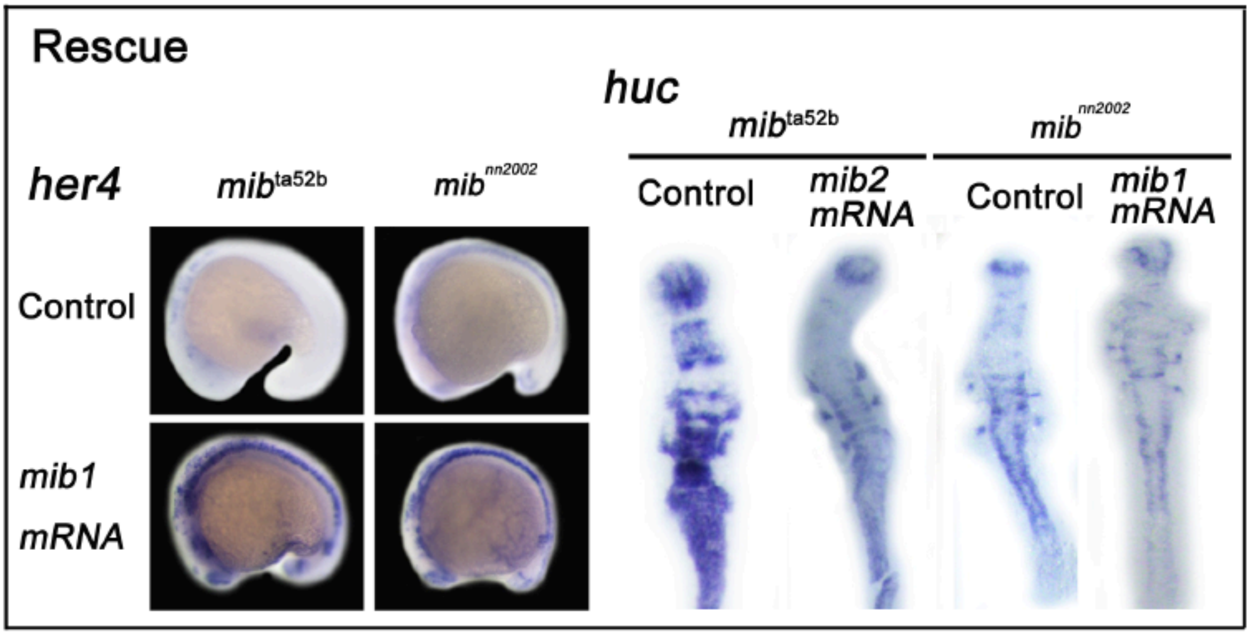


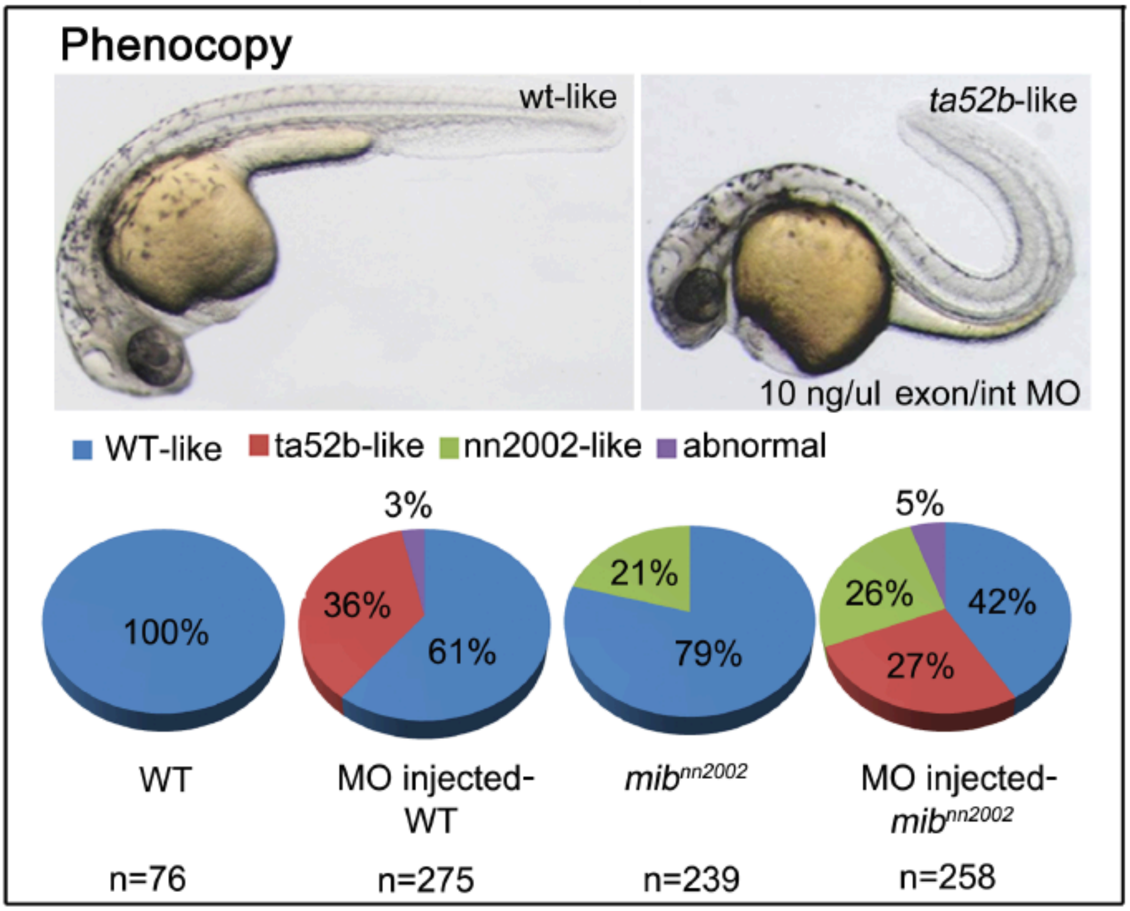


**Figure S9.** The rescue and phenocopy of *mib*$^{nn2002}$ phenotypes.
In rescue experiments, *her4* WISH was carried out in the *mib* mRNA (1 ng/μl) injected *mib*$^{ta52b}$ and *mib*$^{nn2002}$ embryos. 19 hpf embryos in lateral views with head toward left were shown. The expression of *her4* was rescued (increased) by *mib* mRNA in *mib*$^{ta52b}$ and *mib*$^{nn2002}$ homozygotes. *huc* WISH was carried out in the *mib2* mRNA (1 ng/μl) injected *mib*$^{ta52b}$ and *mib* mRNA (1 ng/μl) injected *mib*$^{nn2002}$ embryos. 19 hpf embryos in flat mount with head toward top were shown. *huc* was rescued (decreased) by *mib2* mRNA in *mib*$^{ta52b}$ and *mib* mRNA in *mib*$^{nn2002}$ homozygotes.
In phenocopy experiments, *mib* morpholino injected-wt and -*mib*$^{nn2002}$ embryos were examined. Morpholino can successfully convert 36% of wt and 27% *mib*$^{nn2002}$ siblings into embryos that exhibit typical *mib* phenotypes (ta52b-like). However, the morpholino failed to convert the *mib*$^{nn2002}$ homozygous embryos into embryos having typical *mib* phenotypes. The ratio of embryos carried *mib*$^{nn2002}$ phenotype is 21% without injection and 26% with injection. The ratio of embryos that exhibited *mib*$^{nn2002}$ phenotypes was not reduced with the injection of *mib* morpholino.

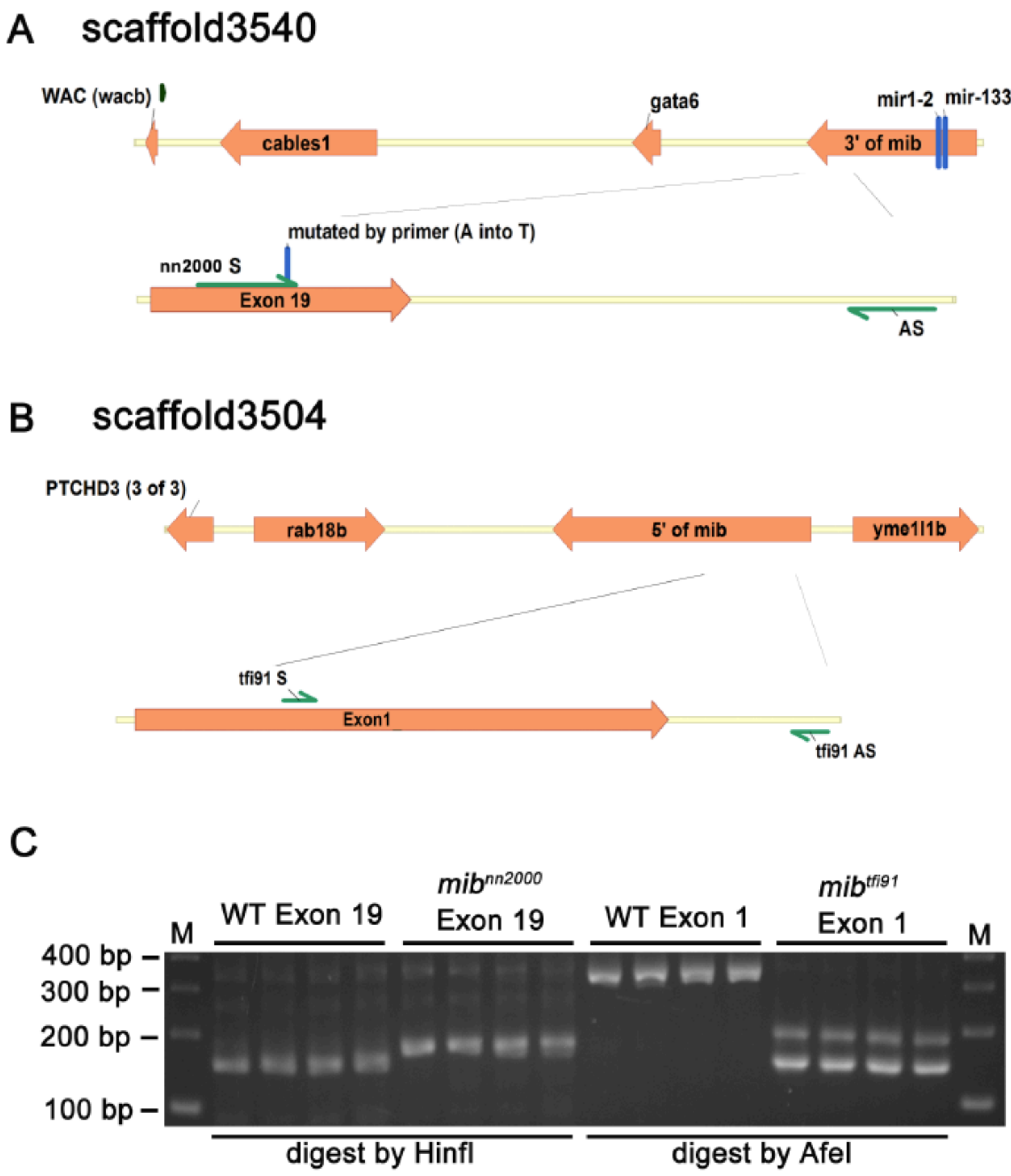


**Figure S10.** The continuity test of scaffold 3504 and scaffold 3540.
Panels A and B illustrate the design of (A) *mib*$^{nn2000}$ and (B) *mib*$^{tfi91}$ genotyping primers related to scaffolds 3540 and 3504, respectively. The *mib*$^{nn2000}$ genotyping PCR products of wt and *mib*$^{nn2000}$ are 173 bp long. Hinf1 cutting site was created by using CTGATGAAAAAGTGTGTGCAGA**A**G instead of CTGATGAAAAAGTGTGTGCAG**T**G. Only the wt PCR products will be recognized and digested into fragments of 152 bp and 21 bp long. The *mib*$^{tfi91}$ genotyping PCR products of wt and *mib*$^{tf91}$ are 335 bp long. Only the *mib*$^{tfi91}$ PCR products will be recognized and digested by AfeI into fragments of 189 bp and 146 bp long. The genotyping results were shown in panel C. The digested PCR products were analyzed on 3% agarose gel. The digested PCR products of *mib*$^{nn2000}$ and *mib*$^{tfi91}$ can be easily distinguished from those of wt with the expected size mentioned above.

## Supplementary File 11

>Zv9_scaffold3504 dna:scaffold scaffold:Zv9:Zv9_scaffold3504:1:82506:1 (encode 5' of mib genomic sequence)

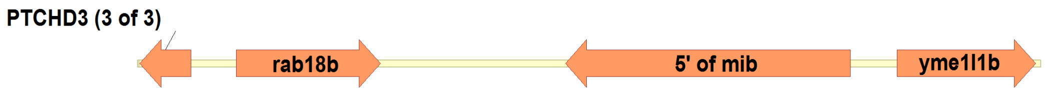


Loci (4 total)

*PTCHD3* (3 of 3) Start: 18 End: 4706 (Complementary)

*rab18b* Start: 8883 End: 22055

5' of *mib* Start: 38977 End: 65063 (Complementary)

*yme1l1b* Start: 69307 End: 82031

#Genomic sequence was compared with mib mRNA sequence (NM_173286.3)

# **Yellow color**: Indicate the site of CpG islet

# **Red color**: Indicate the site of Exons

# **Blue color:** Indicate the site of introns

# **Green color**: Indicate the site of polymorphism

# **ATG**: indicate translation start site

# A← ：The variation on mRNA is A

# →G: The variation on chromosome is G

CpG islet CTGCTGTTTACAGACCCGAGCGTGCGTTTGTTTCGAGCATGAAGGACTGCGCTTTCTTCACCTACCGCCA

TTCATTCAGGAATTATATCTGAACTCGAGTAAACCAGAAGGTTTTAATCAAAGTGCTTCAGCTGGACTGT

TTTTGCAAACGCTTTTCCTGCACAGACATTCTGAACCGCGTCGCGCGAAGTTGGACCATCTGTTGAACGT

TTGCGAGCGCACCGCAAAGTTCTCAACGCGTTTATTTTTTTAACATTTAATCGCAAAACAACTGTTGTTG

TTTCCTAAAGCGAACGTTTTTGTTGCGTTTATGCTAACGCCAAGCAACTTTGGAGAGGTTAGCTATAGCT

GGTTAGCCTAGACTCGCTAACTGGCCTCTGCTAAAAGGGCCGTTAGTGACCTAAACGTATTGTTTTTCTA

CATTTATTCATAACGTGTAATAATAACAACTACAAAGTTTGTTTTCTAATAATCTCTGTACGTTATATTT

CACTAAAACAGTTGCATTTATGAGTCGACAACTTGAGCAGTTGTGAAAAGCATTAGCACTCCAAAAAAAC

TTCAGTTAAACATTTTCCTTAAGGAATTAACGTAAGCATTCTCTCTGTGTTTTGCTATACAGCAAGAGTG

Mib **Exon 1** ACCTCCATCATTGGCCATATGTAAGACGAACCAGTCCTTTTAAAACCCAATATTCTCAAAGCTGGCTGAA

CCGCCAGCTAAAAGTAACTTAAATCGCAACTTTTGCTCGTAA**ATG**ACCACCGGCAGGAATAACCGAGTGA

TGATGGAAGGAGTGGGTGCTCGAGTTATCCGCGGGCCTGACTGGAAGTGGGGGAAGCAGGACGGCGGAGA

AGGGCATGTGGGCACCGTCAGGAGCTTCGAGAGCCCGGAGGAGGTGGTGGTGGTGTGGGATAACGGCACC

GCCGCCAATTACCGCTGCTCCGGGGCTTATGATGTGAGGATATTGGACAGTGCGCCTACAGGTGAGGCTG A←→G

CAGAGCTAATTGACTTCATCTCTCACCTTATCTAAACATCAGCTAACTAATTTATTAGAATAAAACACGC

GTTTCTAATACTGCAGTTGAGGTGTATTTGTATTTTGTGAACTGTATAATGTATAATAAAGTGCTAGTTC

ACACTTTAATAATAATTGCAAATTTAATCCATTGCCATTGTCATTCATTCAGTCATTTTTAAAGTCCCTT

TATTAATCTGGGGTCGCCACAGCGAAATGAACCGCCAACTTATCCAGCAAGTTTTTACGCAGCGGATGCC

CTTCCAGCCGCAACCCATCTCTGGGAAACATCCACACACACACTCATACACTATGGACAATTTAGCCTAC

CCAATTCACTTATACCGCATGTCTTTGGACTATGGGGGAAACCAGAGGAAACCCACACGAAGGCAGGGAG

AACATGCAAACTCCACACAGAAACGCCAACTGAGCCGAGGTTCGAACCAGCGACCTTCTTGCTGTGAGGC

GACAACCTACTGCGCCACTGCCATCCATCTGCGGTCCATCAAATATAAATTTTACATAACATAATTAAAT

GTAATTTGAGGATCCTTCCACTGAAATTTTCTTCACGCAAAATGTTTGCTTCAAATGCGATCTTAAATCA

GTATGAATGGTTTCATCCAAGTCCACCACTTTTCTGATGTGAATATTGCATTTTATGCTTTATGTAATGC

TTAGAGTGCTAGTTCATCCTAAAATCACATGTAATTGAGCGTCACATGAAATGTTTGAAATAAATGTCTT

ATGCATTCGTAATGACATGGGGTAAGTAAATGTTAACTTATGTTTATCTCAAATATTTCATGTGATGCTC

AAGAATGAACCTGATTGGTTCTTGCGGAGGCTCAGATGTGCAGGTGTGACACAACAATGAACCTCATTAG

TTCTTGCTCATGATTGAGTTAAAGTCTAATATTTTACAGTCTATCAAAGTCTATTAATGAAATATGAGCT

CAACTCTTCAGAATACTTTGATTAAACTGCACAATTCATATAGATTTAGTTTTTTTTTTTATCTCTTTAA

AAAGTTTTGGATGAACTGACTATGCAACAAATATTAGATTTCTCACACACTCACGCACAAAAAGTCTGCA

TTGTGATATGATATATGTACAGTAAGAATAATGTTTTGTTTACCCCCACACACACACACACACACACACA

TACAAAATAAGAAAACTCTCATTATTATTCCTCTGTTTCCAAACCTGATTGAGTTTCTTTTTTCCATTGA

CTTAAATTGATATTTTGAAGAATGTTGAAAACTGCTAGCAATTGGCTTTATTTTTTTCTCTACCATGAAT

TTCTGTGGGTACCATTTTCCAAAATTCTTCAAAACTTCTATTTTTTTTAATGTTCAATGTGACAAAATAG

TAATAAAAAAACGTGAAGTTTTAGAACCACATGTGGGTGAGTACAGTTTAAATTGTGAACTGTCCCTTTA

ATTGACATTAAAGGATCAATACATTATGAATAATATACAAATGTGTTTGTTATGCATTAAAGGGGCCCAT

GAAATTAAAATAACGCTTTTTAGATGTTAGTATCAGTATTGTTCATTTTTAGGATATTTATAATCTAGTG

TGCTCCAAACCCGTGGCGAAATTCGCGTTTAGAAGATATAAAACCGATATCAACATGTCATTTTATTCAC

GTCACCTCATACTACAGTTTCTCATCAAATCATAACCAATCGAATGCTCTCTAGTATCTGACATGCCCCG

CCACCTTCAAGATGCTTCTCTTTGGCTGCGTCCAAAACCGCCTACTACTCAGTACAGCATTTGAATTTAA

ACGTACTACTCGGTCTAGAGCTGCACGATTCTGGCTAAAATGAGAATCGCGATTTTTTTTTTTTGCTTAA

AATAAAGATCACGATATTCTCACGATTCTGTAGATGTAAATTAAAGGTATGGTAAAAACAAACATATGGC

ACAACATCGATAATAATATTATGTGTAGGTCTACTGGTGTCTATAAATAATTCTATTCTACTTATAATAA

TTCTGATGTTGAATTATTATGTTTGCGATTATATGCTTGCAATAAGACAATTTTATTCACTGGTAAAATC

AGACATTTGCCATTATCAGATTATATTCAGCAATATATAGGCTAGAGTAGTTATACTGACATTGTAAACA

TTTATTTTCATGCGCAACTGTGTGTTCGCTATGAAAGAGAAAACACATCAAATGCTTGCCGCAATCATAA

CTCTAAAATGCGATATGATCATTTAAATGAAGTGCACACTTTCGGTTTCATTCTGACTGCTAAGTGCTGT

TCATACTTCGCGTGCGGCTCCCAAACGATCACTCTGTGCCACAAACTGAATATTATTTACATCTGTATTA

CCTGTTACTCGCAACATATGCAGGTTCTGTTCACTAAAGTAGACGTGCGCGTCTATCATTAAGTAGAGTA

CGCATGCGCCCTCGCTGTGATAACAGCTCATGGTAAGTAGCTCATGTCACGTGTCATTTCCCGTTCGCGA

AAACATAATTATATTAAGAGAAAAATGACATTTTTAGGTGAATTATACCTTATAAATACAGGTGTCAATG

TCACTGAGCAACGTGTGTGAAACAATGAAAAGACTTGTGCAGCCGCCTTTTCTTCTCTCCTGAACATGAA

AATATGATTGATAGAATCGTAGAAATGCTGGATTAAGATCGTCTGGGGGGTCGAATCGAGATCGCAATCT

TTTAACGATTAATTGTGCAGCTCTAACTCGGCCGTTAGAAAAGTACAATCTATACAGTATGAATGTGAAA

AGTATGAATGGAATTTGGACGAACTACATCCTGACCTACCTGCGTCAGTTGCGTCGCGTCAGTCCCATTC

ATGTTTTGGTTGAGATCGCGTCAAACATCGAATAAAAATGCACATTTCAAAGCACTTAATGGGACCTTTA

Mib **exon 2** AAGAGCCAACTTAAGTTTAAACAGGTGGAAGTCAACCCATTAAATATGAATCTAATTGCAATGTTCCAGG

AATCAAGCATGATGGCACCATGTGTGACACCTGTCGGCAGCAGCCCATCATTGGAATCCGATGGAAATGT

GCAGAGTGCACCAACTACGACCTCTGTACCACCTGTTACCATGGCGACAAGCACCACCTGCGCCACAGGT

TTTACCGCATCACCACCCCTGGCAGTGAAAGGTGAGTGTGGCGTTTCTCCATGTGCTCTTCACTCCTTGT

GGATAAGTCTGCTCTGGCCGGTTTCACACAGCTATGGACCCTTTTCACATTTCTGGGGTTTTCTGAAGTC

GTGTTAGTTTGGTAAACTTAAATGTTTGTGCACACCGGGACGCTTTTTGCTTGCGTTTACCGTCAACGTT

TAACACCTCGTGACTAAATAAAGGGCGCCAATGTGACCGTACACACCAACGCGCAAAACGGCAAGCGCAA

AAGCGTCATTTTTTTAAGAAACGCTTCATGCTCGTTTTTTTGTTTTGATACACTGCGTCAAAATCTTCTC

CACCAATCAGATTGGCACTTTTGTTCACGTGCACGGAGCTGTTAAAGTTACAGTGAACAACACTTGGAGG

CGCTCAAGCGCAGCTGTCAATGCAAGCGCACATAGAAGAAGACGCCCAGAGGCGATATGAACATTCAGCT

GCTTATTGCTCTCTAAACAATAATCATTTCTTCATCGCTAGAGCTGGAGTTGCTGCTTGAACCATCCATG

CTTGTTCGAGTCACCAGTAACTTTAACAACAGGCATATGAACTTCCTCTCTTCCTCTTCTTTTGTGTTAT

AAGCGGGGTGTCAGAAGCTTACAGTTGTTTAGCGCCATCTAACATCAAGGAGTGATTTTGCATACTCTTC

TGCTTGACGCCAGTGAAAATTGCTTGGGTTTGAATCCGGAAAAAAGGTCTCGTGAAACTCTGACGATGAA

CGCAAACGAAAGCGTCCTGGTGTGTACGAGCACTAGGCCCGGTTTTCTTGAACTATCCTCAGGCACAATT

GTGCCCTTTCCCCTTGACCTGCACTCACACTGCATTATTTACCTTCCGAGCCTGAGCGCGCTTGCGTCGT

TGATAATGCGACTGTTCAGTTTAACAGAAGAGAAGATTGGAGTAGAGATTGCTTTAGTTGTATTGTTTTG

TATTATTTTAAAATGTTTGTGATGCTATGACAAACAGTCAAATATTTTGCTGAACAGATCCTAAACTTTT

GACGCTCATAAATGTTCATGAAAGTCATTGTGCTGCTCGTATTAAGAGGGATCCTGGGATTTTTAACGAC

TACAGAGAGTCAGGACCTCGGTTTAACCTCTCACACCCCATTCACACGGGGTGTCAGCGTCAACGCTTAC

TATTCACTTTGAATGGGTGACGTCAGGCGTTGCCGAACTGCATAGTGGATCCGTCGGCTCCGCTTCAGAG

GCGTTGCTCAGTGCAGAAGTTGGGACTAGCTCAACTTTTCAAGTGCCGACAGATCGCTATATGCAAATAC

ACCAGCTAGACAGTGGCCTATTGCTGACTGAATTTCATTGGCTGACGCTGCTATGATGATCGCTTTAGCC

CCAACTTCAGACACGACTTCAGTCAAGCGTTGACGCTGAAGCCCGTGTGAATGGGGCGTCATCCGAAAGA

CTTCGCTCACTGAGAAGTATAGAGTCCCCGTCACTATACTGGGGCATTAGGACCAACACAGACCACAGGT

TGGACGCCCCCTGCTGGCCTCACAAACACCACTTCCAGCAGCAACCTAGCTTTCCCATGTGGTCTCCCAT

CCAGGTACTGACCAGGCACAGCCCTGCTTAGCTTCAGTGGGTGACCATGTGAGAGTTGCAGAGAGCTAGC

TGCCGGCTGATTGATATGTTGAGCTATCTTATGCTGCTTTGACCCAATCTACATCGTAAAAGTGCTATAG

AAATCAAGATAAATAAGATCGCAAGCAAGATCTCAATCGCAGCAAGTCTTTGGTCATGATTAGGTTTGTT

TTTTACCTTATTTTATGAAGCTAGATAAACATTTGTTTTACATTTAGTAATATTGTGAGAGAAGCTGCTG

TTTTAGTTGTGTATCTGCTGTCTATTTCTTTTTAATGGCTTGGGTAACGTCTCCATATGGCAAGGCTATT

ATGAAGTCTAGCAGTTGTTTAGACGTCACTCGTGGCAGCCATCCGTTACTGCCCTAAACATGAGCTCAAG

CAAACAATAGGTCAGCTCAACAGCTGGTCTCCTTTCAACTGAGTCTGACTCTATTTCCCCAGTCAGATTG
GAGAGTGAAGTGCAGCACACAAAGACAGTGGCCTTTTTTTTTAAACTGGGTGTTTCCTCTGGCAGCTGCT
GTGGTTTCCAGCTGCTTTAAGACACATGTGATCTTAATACAACCAGAATAAAATTCTCTAGTGCTTTTAT
TTAATTTTTTCTTGTCCTGAAGTTTTTTAATTTTTTTTAATTTTGCTATTTAAAGGCAAAATTGTCTTTT
TAAAATACAAAAAAACCCACCTGAACAGTGTTATATATTTTACTGGCTTATACATTTGCATTATCCTAAA
TGTTTTGAAGAATGTTCAAGTCAAGAGAAAAAGCTTTCCTCCTTACTGACGGCTGTTGCAATTTCAAGCC
Mib **exon 3** AATAATTTTAGTTTTGTACGTTTCATATTTCCTTTTTTTGCTACTTCAATGTGTTTGCTCAGAGTTCTTC
TGGAGTCTCGTCGCAAGTCGAAGAAGATCACAGCCCGCGGCATCTTTGCAGGAGGACGAGTGGTGCGAGG
GGTGGATTGGCAATGGGAGGATCAGGATGGCGGGAATGGAAGAAGAGGGAAGGTAATGCCTCGACACGTC
TGACTATGCTCACCTTCCTGTGCTCATCTTTTATTACATGAAAAACATTCCTCTTATCACGTGCATGCAG
TGTTTTGTGCTCAATTTAATTTCAACGTTGTTTTTATTGTTTTTAATTATGATACAAAACCCAGAAAACA
ATACAAAATATTAACTTGTATAATATTTCAGAATTTACTTTCCCACAGCAACATTATTTAAACAGAAATA
GCATTTAAAATAAGTTTAAAATAAAACACAATGCTATTATATAAAAATAAAATTAAGAGTAAATAAATAC
CTAAAGAGCCCCTATTATGTTTTTTTTTTTTTTTATATTTCCTTTCATTCAGTGTGTAACACAGCTGTAA
GTGAATGAAAACATCCAGCTGAGGGTTAACTCTGAAAGTGCACCTTGCTTAAAAATGCAGATTTTTTAAA
ATAAAAGATTTGACTTAGTCAAATCTTTTAAATGTCGTTTAAATGATTTGTTGTTCACTCGGATATTTTG
CCTGTTTGTATTGACGTCAACACAAAACAATACCAAATATGTTTTGTGCCGGATTCGGCAAAACGTGAAC
GTGCCTTTCCCTTCAAACACTTGCTTCGTGCAGGTGTGGGACTGATCATGACTGAAGATGAGTGAACATT
AATAAGTAAATGTTCTGGTGATTAATGAAATAATCTACCCTGCAACGGGGGATTCGTCGGCTGTTTGTTT
GAATTCTAGTGTATGGGACTTTGTCAGAGCTTGACGGGTACTTTACAGTATACAGTTCATGGATCAGTTT
CTTCCTCCATTCACAAATGTAAGTAAGTGCGAATAAAATTGCTGCCTCATTTTCTCTAGCTTGCAAAATG
TGTATTTAGTCGGCGCAATAGCCTAGTAGTTAGCGCGCCGACATATGTTGCAATAGCACGTCAGGGCGTC
GCGAGTTCGAATCCCGGCTCGACGACATTTCCCTACCCCCTCTCTCTCTCCCACTTCGCTTCCTGTCTCA
ATACTGTCCTATCTAATAAAGGCAAAAAGGCCAAAAATAAAATCTAAAAAAAAAAAAAAATGTGTATTTAA
TTGTAATGTTACTTGTAATGGCTCGTCGTGGCTTGTAATGTATCTCGTTAACTCTTTATATTCTTATCAA
ATCTAAAACCATGTTGAAAAGGCAATGTGTGTCTCTTTGTTTACAGTTTTAAATGCGTTTGTAAGCTCAC
GATCCTCTGCCGTTTGTCATTGCATCAGCTGTTCCTCACACGTGTAGCAAAGTTTCAAAATAGTTAATCT
TTAAAACTGTGTGGATTACTACTGTGTGTCGTGGTTAACAATAAAGCAATTTGCTGGTGTTAAACTGCAT
TGCTTTAATGCACTCCTACGTTGGGCTCACACATACACTCTGCACTCTGACTACTTCTACAATGTTGATG
AGTCGTGGGGGCATTTCCATCTGTCTCGTGCTGAACGCAGTCGACCAATCAAAACAGACTTGGTCATCGG
ACCAACTATCGCAAATTATCATCGTGCTAAGGAGGGGTTTGGGAACAAATGATGGGGGGTTGTGGCGAAC
TGAAGATTTTGAAAGGGCACAATCTATCCAAGACTAAAAAGGGCATGTTGAGGTTGAGGTTGAGCCCCAT
GTGTGCACGTGCCTGAGGTCATTTATGTATTTCTTACACAAATTCTTCTTTTTCTTAAATTAATTGCATT
TTTGAGGTTGTAAACATGCTCGAAAACTGACAAAACTTTGCACACACATTAGAAGTGGCTAAAATTTTGA
TATGGTTTCGGAAGAGGGTGTGGCAAAACACACAATAAACATGTCAATACTTGCAAAAAGTCTCTGAGGC
CATGTCTACGCTAATCACGTCCACTTCACGTCCACACTGTCGTTTCTATTCATTTTCCAAAACATTGTCA
TTCCCACTGAAACGACTATAAATGCTTTCAACCTGTACTGTGCATGCATGAAACTTAAGACGCTTCGGCA
CGCGTCATTTCCGCCTGACGTTTATTTTCTTTCCGGCTCTTTGAAACGTTACGGCAAAATGTCAAGGAAA

AGCACAGAGTTTTTTTAATGGACTGACAATGAAGTAGAGTTGCTGCTTCGAGTAGAGTGAAAATGTAGAC

TGGGACGTGCCAAAACAAATACGTCGATATACTAGACCGCTTTAAAGAACAGTATTCTGCTGCTGATGCT

GCTTACCCTCACAGTAAGGACGACATCACTAAAATCAGAATCACAACAAAATTAAAGTAAACGAATTAGT

AACATGACTGTCACATGACAAAAACGCGTCCGTGTCTACAGTCTACACTACGACACGAAAATGGCGTTTT

CAAAATTATCCACTTTGGAGAGCGTTTTCAAAAAGATACGTTTTTGTTTCCTAAAAACACGTCTCAGTGT

GGACGGATGAACACGCATTAAGGCTGATTTATACTTCTGCGTCAAACGCCGGCGTATGCTACGGCGCTGA

CGCATAGCCATGGCCGTCGGCGTCGCTGACATGCACCTCTCAAAAAATGTAACTACACGTCGCAACGACG

CGTAGCGCAAGCTCTGTGATTGGTCGGCTTGGTAGCACTGACGAGTCTGGGCGGGACCGAGAGCCGCGCG

AGCTGGACGCGAGCCCAATGGAGCGATTTTTTTTTTTTTTTTTTTTTTTTTTTTTTTTATACAAGTGTGG

AGTCCCGTGAAAGAGCTCCGGATGGAAAGTTTTGTTTTGTGTTTACCTCATAGTTAATGTTGTTGCACGT

CCGCCGGTTCCTGCCTCAAAATGAGTGAGTTTGAGCCACTTGTACTTCCAGGAAGTGTTCAGGAAAAGCA

AAACAGTAGAGAAGAAACTCGACACAGAGGAACGTTTACACCTCATTGCCCACTAGCGTTTTGGAAGTGT

TTATGCATACTTCGCAGACTTGTTAAGACGCCGGTCACTTGACGCAGAAGTATAAACCAGGCTTTAGTGT

GGACATGGCCTGAGTTAAATTCAAACCTGAACCGGAAGTTGGAAATTTTTAACTTTCCTTTCGAAAAAAG

AGCAGCTTAAATTTTTTTCTTGTAATTTTGATGCAAGATGATAAAATCCGTGCATAAATACATAAATAC

ATAAATAAATGTTTTTTTTATTTAATAAAATCTAAATATTAAATACTTTAAAGGATTTAAGATGCTAATG

ACCATTAAACGTGTCTTATGAAAATGTCCATAGTGCATCATTTGGGACACTAAATCAAGAGGTTCATTAA

CTCTTTTGAAGGTTTTGTTTAGTCAGTAATCACTACAGCTACTTTTCTTTTCTCTCATAAACTGTCCATT

Mib **exon 4** TCAGGTGACGGAGATCCAGGACTGGAGTGCTGCCAGCCCCCACAGCGCAGCCTACGTCCTTTGGGACAAC

GGGGCCAAGAACCTCTACCGAGTGGGATTCGAGGGCATGGTGAGCCACCCCCATTCCTACACACACACAC

ACAAAAGACTGCAGTTAACAGGCCTTTGTGTTATTACCCACCCCCCCGTCCTACCCAACACTGCACCACA

CGCAGGCCAGGCGTCCCATAAGCTGGCCTTCTTTATAAGGGGCAGCCATTGTGAAGGGCAGCTGGATGAG

CTATCTGCATGATAAAAGAAGTCTGTTGGCTTTACAAGAGAGCGAGACTCGACACCAAGACCAAAAGCGT

GCCTCAGTTGTTGAAGGCCGCCCTTTGATTGGCTGATCCACTTTCTGCAACATCTGAATTCCAGCCAATG

GCTCTGCAGATGCGCTCCGGCTTTTGTCTGTGATTGCAGTGTGTCCGGAGGGTGTGCGCAGCCATCTGCC

ACTGGAATTATTAATTAGATCTGGATATAGAGGAAGATATATAAGGAGGAATAGAGACGTTCAAGTCATC

TGGTGAATTAGTACTCATGTCGCAGAAACACTAAATATCACAATATCAGATTTGTCCAATGCCATGCAGC

Mib **exon 5** CATAATCAGCTGTCTGTTTCTGTTTTTTCAGTCTGATTTGAAATGTGTGCAAGATGCCAAAGGAGGGACT

TTTTACAGAGACCACTGTCCAGTATTGGGTAAGATTTAAAATATCTGATATTACCAAAATATACCATGCC

GGTTCAATGAATATACAGTGGGGGAAATAAGTATTGAGCACGTCACCATTTTTCTCAGAAAACAAATTTC

TAAAGGTGCTGTTGACTTGACTTTTCACCAGATGTTGGTAACAACCAAAGAAATCCATATATGCCAAAAA

AACAATATTAATTCATTTACTAATGAACTTCTGTGTTATAAAATGAAATGACAAAGGGAAAAAGTATTGA

ACACATGAAGGAAGGAAGCTGTAGAAAGACATGGAACGCCCAGACAGCAGCAGAGGTCTTTCTTATTGTA

AAGTAGTATTAGCTGCTTTTGTGCAACATCTACATAAATCAGGAGGATGAAGATGAAACCAGGGTGGACA

TTTCAGCAAGACAATGATCCAAAACACAGCCAATGAAACTCTCAAATGCTTTCAGAGAAAATAATCTAGC

TGTAGAATGGCCCAGCCTATCACCTGACCTGAACCCAATAGAAAATGCAAAACAAAAAGCTCAGATTTTA

TAGACAAGACCTACATAACTGTCAAGGTTTTTACACTGTTGAAGTCTGTGAAAAAAAATCACACCTGAGC

AATGTATGTGACTATATTCTCCATATGAGAGGCGTCTTTAAGCTGACAGCATCAAAAAAGCCTTTTATAT

AAAGTATTAAATACATTTCTGTAGTTCAGCACCTTCTCCTTGTGTCTATTGTTATTATTAAACAACACAT

TTTTTCAGATTTTTTTGGTTTGGTTTTATTTTTATGTTTGTAATGTTTGGGTTTTTACTAAAATTTGGTT

TAATCCCATGTCAACAGCTCCTTTAGAATATTATTCCCAGAAAAAAAAACATGACTAGTTGAGTACTTTTC

CCCCACTCTAGTTATCTTTGTCAAGTAAATGTCCTCCAGTATATGTTAATGTTGTATGTGATGCCTCCAC

TAGGTGTCAGCATTGGATGCCTAATATAGCTGATCTTCAAACATTCATCGCTACAGTGAAACATTCCTGT

TTAAAAGCTATTTTGGCCCCAGTCCATAATTTACTCTTTGCCGTCCAAACCAATAACATAAAGCTGTTTG

CAATGATGGTCAGCTCAGATTAATTACACTTGAGGCCTTAAAAAAAAAAAAAAAGGTCCAATCATTTTTAA

AGATGTTAAGCTACATGCCACTTCACAGGATGTTCACTAAGTTTTCTACACTATACTGAAACGAATAAAG

ACATGTCTGTTTAGTAAAAAATGTATTTTTGAAGAAATGTTTATTAAATTTTTTATTTTTGTTTATTAAT

TAATTAATTTATTTATTTGTTTATATATTTATTAATTTATTTATCTATTTACTAATCTATCTATTTATTT

TATTTATTTATTTATTTACATATTTATCTATTTAGTAATTTATCTATTTATTTATTTATTTATTTATCTA

TTTACATATTTATCTATTTGCTTATTTATATATTTATTTATTTATCTATTTACGTATTTATCTATTTTCT

TACCTATTTATTTATCTATCTGTTTATTTATTTATCTATTGATTTAATTATTTATCTATTATTTATTAAT

TAGTCCATTTATTTGTTTATCTATTTATTTAGCTATATATTTGTTTATATATTTGTTTATCTAATTTATT

TATTTATATATTTATTTAATTATTTATTTATCTATTTATTTATCTATGTATTTATTTATAAATTTATTTA

TCAAATTTATTTATTTATTTACTTATCTAATTTATTTATCTATGTATTTATTTATTTATCTATTTATTTA

TTTTAATCTATTTTATTTATATATCTATCTATTTATTTATCAACTTAGAACCGAAGTACCGCTTCTTTTG

ATAACACCAGTTAGCTCCAACACATTACATAGAACATAAATACCAAATTCGCTAGAGACTGCAAATACAT

CGAAATAGGCTATATTTTACCTCCCATAATGATAGTCAGACAGGCAGGGTGGATTAATTTTTGGTAGCCC

AACTGGAAAACGTAATGACGTTGGCCTAGCAGTATTTCGAGCCCTGGTTCAGCTGAATTTTCCTAGTTTT

Mib **exon 6** GTTTAGCCTTAGATGATAATGATCATCATATTCGTCCTGTTGTTGGTTTTCTGTAGGTGAGCAGAACGGC

AACAGGAACCCAGGAGGCCTGCAGATCGGAGACTTGGTCAACATTGATTTGGACCTGGAGATCGTTCAGT

CACTGCAGCATGGGCATGGAGGATGGACCGACGGCATGTTCGAGACGCTCACCACCACAGGCACTGTCTG

CGGCATCGATGAAGACCACGACATTGTTGTGCAGTATCCTAGCGGAAACAGGTCAGGCACACAGCATTGC

ACTTATTCATGGGACATTATTTATTATTAGGGTTGCACAGTTTACCGGTACTATGGCAGTATCGCAATAC

TATGGCTAGAAAATACTGGCAGTGCCAATGTAGTTTGTAAACGGTAGAATCGTCATTAATGGTTTATCTA

TAATAGATGTTGTTGCATGTGGAAAAGTCTCTAAAATGCTTATACATTTGTTCAATAATAGACAATTTAT

TTTAATAGTCTGTAGAGCCCTTTAACGTTGCAAAACTGTTTGCCGGCAGCTAGCTCTCTGCAACTCTCAC

ATGGTCGCCCCCTGAAGCTAAGCAGGGCTGCGCCCGGTCAGTACCTGGATGGGAGACCACATGGGAAAGC

TAGGTTGCTGCCGGAAATTGTGTTAGTGAGGCCAGCAGGGGGCGCCCAACCTGCGGTCTGTGTGGGTCCT

AATGCCCCAGTATAATGACGGGGATGCTATACTGCTCAGTGAGCGCCATCTTTCGGATGAGACGTTAAAC

CGAGGTCCCGACTCTCTGTGGTCGTTAAAAATCCCAGGATGTCCTTTAAAAAGAGTAGGGGTTTAACCCC

GGCATCCTGCCCAAATCTGCCCACTGGCCTCTGTCCATCATGGCCTCCTAACCATCCCCATATTCAATTG

GCTTCATCACTCTGTCTCCTCTCCACCAATCAGCTGGTGTGTGGTGTGCGGTCTGGCGCAAAATGGCTGC

CGTCGCGTCATCCAGGTGGATGCTGCACACTGGTGGGGGTTAAGGAGATCCCCCCCCATTGTGTAAAGTG

CTTTGAGTGCCCAGAAAAGCACTATATAAATGTAAGTAATTATTATTATTAATTATTAGAATTCACACCT

TTTATGGTAGCCTATTGCACGTTTTCTGAGGCTTTAGTTCAAACGCACATCTGAATCAGTTCATTTCTGT

TCCTGTTAATATGATTTAGTAGCTACAACAACCGTGTCGATCTCTTTAAAAGAACGACTCACAAAGAGAC

TTTTATTTGGATTGTTACCTGATAAGACCAAGAAAAAGAATAAATGGAAAACCCAAAATAGCAACACGAA
ACATTGAATCAATGTTTGACCACTTCATATAGGCTAATTTTGTTGGAAAAATTTTGTAAATAAATTTATT
TTCATGTATTTTTGTTGGTCAGTTATCTTCATGTATTTTTGTTGGTCAGTTATCTACAAAATAAACAAAA
AGAATGAATACCTTTTAGGTGAAGTAAGGTGTAAATAATACTTTTTTTTGTCTTAAGGGAATGTGGAAAT
ATAAATTTCTATCATCTAATTCCAAATGAACAATAATCTACGTATAACTAAAAAGTTATATTCTTTGGTT
TTTAATTATATCATTTCATTGACATGCCAGCTAGGAGGGACATTTAACTTTTGCCTTGCTGACAACAGGC
TAAACCAAAGTGATGGAATTATCGGTTTCACAGCATGGCTGTATCTATTGTCTTCTCTGTCTCCAGAACA
TCATTTGTACACTGTAGTACTTATATACTGATGAGATCTGGAGAAGGCATGGCACCCTGCAGACAACCAG
ACTTCATTTGCATGCTTTGTTTGACCGTCCCCACCATGACAGACCCCACCTACATGCTGTAAAATGTACA
AATGTATAATGTTTGCTGTGGAGTTTAGAGTTTGCTTTCGATGACGCCACAGCCACGCTGAGTTCTTCTT
ATTAAAAAGGATTCTGCTTTGAAGACAACCGAAAGATTCTCCCTGGTTTTTGGGGCTCTTAGAAGTTTAC
AACAGGAATTATAAATTACATTCAAGTAGGCCTATTATAACATAAAATATTAATTAAATTACATATTTCT
TTATTATTTGAGTTATGAATTACAGTATCGCTATACTACTTGGTATTTTGATACTTCAGCTGGTTGAGTA
TTGTAAGATTAATATATGGTATTGCGACAATCCTATTTATTATCGTAATTTTTTGCCATTATTTCTCGGT
GAGTGACAAAAGCAAACATTATAACCAGGGCCAGATAGAATCTACAAACATTTATTGGTATTTCTGCACA
AAATGTTGTCAGTATTATAACTTAACACATAAAGTGTTTTTTTATTATATATTTATTTTTTTACCGTCAT
CTGGGAAACGGTTAGGATTTCAGTTCGGTAAAAAAACTATTAGTGTTCCAATTGGGATGACTTTAAATTC
ATATGCGTGTAAATGCATAAGCAGGGCTTAATTTATGCCGGAACAAGCCTGATCCGGATCCGGTACCTCT
AAAATCTGATCCGACACCTCATTTTACCCATCCCCCTCCTCATACGTACCCCCACCCCTAACTTTCTTCC
GCAACTCCCCAAACCTCTTCACTTTCGTACGCGATCGTATATTAAATCTACTGAAAGTATTACTTCCACT
Mib **exon 7** CTGTGGCCTTCTAATAATCGCAGAGCTCTGTATGTGTTCTAGGTGGACGTTTAACCCAGCGGTGCTGACC
AAGGCAAATGTTGTCCGCAGTGGAGAAGTGGCAGCTGGCGCTGAGGGCGGCAGCTCGCAGTTCATGGTGG C←→T
GAGATTTAGTCCAGATCTGCTACGACATCGACCGCATCAAACTGCTCCAGAGAGGACACGGAGAGTGGGC G←→A
CGAAGCCATGCTGCCAGTGAGCATCCCTAGTTTAATGCAGTAGTGCTTGTCCGGTCTGAAACCCACTTTA
TATCATATATCAATCAGGCATCAGAGATCACTGAGTTTTAAAGTTATCAGATCTTAAAAGTGGCGATTTT
GTGATGAAAAAACTAGTTCATCGGAAGCACTTGGGGTGTCTGACTGAAAATCAAACTCAGTGTGCATATC
CTCCATTGTGCCACCTCGATTTGACGATAACCCCAATCCAACGTTGGCCAATGCAGACCGAAGGCCTGAC
GTCTACCGACCCTTTAAAGGTGCAATATGTAAGTTTATAACAACACAGATCACTGCCTATTCACGAGTCC
CGCAGAAAAAGTGATTGACAGGTGGTAATTATGTGTGTATCTTGCCTTTATTCATTTACTGTATGATTTG
TTTATGGGTGAAACAAAGACCATGCAGGTCAGGTAGTTTAAACGGTAGGCTACAAATAATTAATTGGTCA
TTAATTATTTAATTATTCATAATCGTAAATCGAATCGTGCCTTTAGAATCGAAAATGTAATAGAATCGAG
AATTTGGAGGATCGTGACACCCTTAGTCCGTATTATGGTATTATCATGCGTTAGAAGAGTCATAAACTTA
CATACAGCACCTATAATGAGCATTAAACTGCATCTGCTGGCCTCCTCTACAAGTAGCACACTTGTTTTAT
CTGCTTACTGTTTAAGCGGCTCAGCCTCCTGAAGCACTGGATGTTATCCCGCCAGTGCGCTCCTGGGCTG
GGTTTCTATATTTTTGGATCGCCTCCCTTTTGTTGCCCTCGATGGACAAAGCTTAACAGAATTCCAATAG
CTTCGGTTATGAGCAGTATAGATGAGAAATGGTCCTGCAAGAGCCTTTGGGTGTTTTCCGTTTAAATGAG
CCAATCTCAATTTTAAAAAGCCAGACCTTACACTTATTGATTTTTCTCCACGTCTCTCTTCACAGGGCTC
TCAGAGGGGCGTTTTGTACCTGTGTGTAACAGGCAGTGTGCACACACAAACACACAGTTGAAGTTTTCTG

TTGAACAGATTTCTTCCATCACATTTCTAAACAATAGTGTTACTAACTAATTCCTAATAACAGATTTGTT

TTATCTTTACTGTGATGATCGCACAATATTTGACTAGATATTTTTTAAGATGCTAGTATTCAGCTTAAAG

TGACATTTAAAGGCTTAACTAGGGTAATTAGGTTAAAGTTAGGTTAATTAGGCAAGGCATTGTCTAACAG

TGGTTTGTTCTGTAGAAAATTGAAGAAATATATGTTGCTTAAAGGTGATGCAAGTCATTTTTTAACTCTT

CTAAAGCAGAGGTTCCCAAACTTTTCAGCCCGCGACCCCCAAAATAACAATGTCAGGGAATCACGACCCC

TATTTTTCAAGGTGGTTATACTGTGAATACATTGCAGACACAACTACAGGCACATATAAACATGAGGATA

TTGAAAACACAAAAATGCAAATCTATTAACCATATAATGTTTTTTTATAGTAATTTATTAATTAATATTA

AAAATTACATTTTAATATAAAATTAAAGGCTGATGGCTGATTTTTATTTGTATGTTGTTTCTTTAACACG

CTTAACTCATCTTCTGTGGGTTATGAATAAAATAGCTTAATAAAGTTTATTTGAGTTTTGTAAATCACCA

AGGGACCCCTTGTCATTGTACCGCGACCCCCACTTTGAGAACCTCTGTTCTAAAGCATAAAAATACCGTA

ATATGTTTGCAGATCTTTAAGAAACATGTTAAATGAACATTCTTGTTCATCTGAAAAACAATGCTGAAAT

CATGTTATTATCATCAGCTATTATCAAGAAGTTTGTTATTTCAAATATAGACGGGCGGCTAGTGCAAGTG

AGAAAGCGAAACCGCTCGTGCTTTAGACCAGCGGTCCGTAAAACAATTGGTACCGGGCCACATAAGAAAT

CATAAATTATTTCTGTAGAAAATATTCCCCCCGCGACTTGGTTTCTTCCACTCACCCCCGCCATCTCGCG

TGAAATGGACTATGCCAAAATCCACCACGGAGCGGGGAGACAGATCGCTAGAATATCGCTCAGTGGTGGT

TGTTGATAGTTTAAAGGTATAAAACAGGTTTTATAGAGTCCAAATCAGCAGAGTATTTAAGGATTGTGCA

TACATGTGTGCGTGCATCGCTAAGTAATTACCCGAAATCATTGCAATATGTATCTATTTGGAGTTTCACA

TACTCATTAGGGCTGTAACATGCCAATGCATACATTTAATTCGGCTAAACGATTGTATTATTTTTAATAT

GTTCGTCAGTTGAAATGTTTGGCACAGATGTTTTGTATTTGATGCATATAAAACTTGATTGAAACATAAT

CAGACCGCGATCAATCTGGCAACCTGCGCTTGCGAGTAGGCATGCAATACCTAGTTCAACCACTGGGTGT

CAAACTTGCATACTGTACCTTTAAATGGGCTAATAATATTGACCTTAAAATAGTTTAAAAAACTGCCTTT

ATTCTAGTCGAGAAAAAAAACAAAACAAATAAGACTTTCTCCAGAAGAAAAAATATTATCAGACATACTG

TGAAAATTCCTTGCTCTCCTCATTTGGGAAATATTTGTGGAAAAAAAGGCTAATCATTTTGACTTCCACT

GTGTCTAGCATTCTTGCCCTTTCTGAGTGTTTGCGAAAAGGCTTGTGGGTTTAAATGCAACCGCTCTCGT

Mib **exon 8** ACTCAGATATTCATGTGTGTCTTTTTTTTTTTGCAGACCCTTGGAAAGGTGGGCAGAGTGCAGCAGATTT

ACTCTGACAGTGACCTGAAAGTGGAGGTGTGCGGGACGTCCTGGACATACAATCCCGCTGCTGTCACTAA T←→C A←→T

AGTGGCCCCTGCTGGATCTGCGGTTACTAATGCTTCTGGAGGTAATGCACGCGTGCGCATTTTGTGTGAA

ATAATCACAAAAGCACAATATAAAAAAGCATTATATTTTATTGTAGTTTTTAAATTGTTGTTAATGTGCT

GCACTTTCTTTTCAAAGTTTAAAAACAATCTAACCTATAATTTCCAAGTCATATTGTGTTCTTGATTGTA

TTCTGGCAAGTTTTGCAGCCCTAATTCCAATGTATTCTGTCATTTAATGTATATGTCAAATGCAGAAAAT

GCCCTTTAATGCCTGTGTGAGTCGAGCACAGTCAGACAAGCATCAATCCGTTTCTCATCCGTCATTGTGT

Mib **exon 9** GCTCTGTGTGACACTGCAGCAGTGTTGACAGAGAGTCCTTCTGTGCCCGCAGAGCGCCTGTCTCAGCTCC

TCAAGAAACTTTTCGAGACGCAGGAGTCTGGAGACATTAACGAAGAGCTGGTGAAGGCCGCAGCCAACGG

TGACCTGGCCAAAGTAGAGGACATCCTGAAGAGACCCGATGTGGATGTAAGAACCCGTCTCTAACACACA

CTTTATTCTGTGCAATAACTGAATGTTTGTTATAACTTTAGCCACACTTTAATTTAATTTACAGTTTAAT

GTTATTAACAAACTATTAACTAAGACTTACTTCAATAAAATACTCTTTAAATACTCTTCTCTTTAAGGCA

GGGGTCACCAATCTCAGTCCTGGAGGGCCGGTGTCCCTGCAGGGTTTAGCTCCAACTTGCCTCAACACAC

CTGCCTGGATGTTTCAAGTATACCTAGTAAGATCTTGATTAGCTTGTTCAGGTGTGTTTGATTGGGGTTG

GAGCTAAAATCTGCAGGACACCGGCCCTCCAGGAACAAGTTTGGTGACCACTGCTTTAAGGCAAGCCGTT
AGGTCAGTATGCTCAGGCATTCTGTTTGAATCGTGAAACATCATATTTTCCAAAACTGCTTGCCGAGCTT
GCGAGTAGATTACATATTAAGAATCGTGAATAAAATTGAACGACTTTAGTTTCATTTTTAAACGTTCGAA
TTGCACAAAATCTGCAGAAACGCACGTCTGGTCCCAGCCCCTCCCCAGGAGAATCGTCAGTCTGTAGTGA
TCGATGATTGGCTCCTGTACTACAAGGCGGGGCTTAATTTGCCTTATTGACCGTTATCAAAGAGCATAGA
AATACCAAGAGGCGACACTCTAGTGCAATTTGGCAAACAGCCACTAGATGGCACGGCGGCTATTTACAGC
ATATTATAAGTCTGTAAAATAATCTATTAAAAGTGCTGATGATTGTGATAGTAAGTGCTGTATTGTCGTC
TTTCAGGTTGTATCTCAGCTTTAAAGCGCTTTTAAAATAAATAAATAAATAAAAAACAAAGCAGCTGCTT
GCCATTGCGACAGCAATAAGATCCAATGGACAGCCGATTGCCTTCACTCCGAAATAACAGAATCCGGGGC
TGTTGCTGGGCACTGCTGTTGCAATGGAACGTTTTATTGAGTGTCACCTCTAGGTCATATGATTGGATGA
TTAGAAATTTGCATTGAGCAGTTGGCAGGTGTACCTAATAAAGTGGCCGGTGAGTGTAAATTATCCCAGA
TCAGAATGTGCGCCAAAGAAGAAAAAGAAAAAAAAAATCTGTTCAAGTCTTTTGTACAAGAACACATTCA
ACCTGATGGGTAGCATTTCTTACATTTTAAGAAGTGCATGATTTAGAATTGAGGAGCTTATGAAGCAAGC
GGTGCTTTGATTGAGCAACACACTGCTTCTAGTTTATGGCAAGTCTGCTTTGAACCGCTCTACCTTCAGC
TTTTACTGGAATCTCACAGATCAGATCAAACACACCATCAAAGAATGACGATTGCTCTTATATGTATGAG
ATTTCCTTTGCTCTTCTTGCTCAGAGTCTGAAATTCACAGTTGAAATGTGAAGCTACTTCAGTTTTAACA
AGTCTCTGAAAGTATTCACATGAAAACACGGGCCATGTTGGTTGTTTCAAATCAGACCGGACGAATCTAA
TACAGTACTCAACATTTGAAGCGGATCAGAACCTTTAAAGGTGCAGTATTTAAGTTTGACACCCAGTGGT
TGAACTAGGCATTGCACTCCTGGTTCAAAACGTACACAAAAGCAGGTTGCCAGATTGACATCACCAACAA
AGGCTGATTCAATTTGTGTTCTAACTAAAATCAACGGCACGCGATAGAAGGAATATTTTCCATATTAAAA
GGAGTTTTTGTTCTAAACAACAACTGAAATGTTAGACAGCTGTAAAATGCCATTTTACATCACATTTGTT
GCCATACTACTGAAAGCAGCAGCAGATAGTTCACCCCAGATCTGGAACTTAAAGTAAACCATTTGAAATT
GATCTTCAGAACCGCGACTCAGGGCAAGCCAACACATATCAGTGATTCAGCACCTACATTTAATCATGTT
AAAGAGGTTTAATATGTATTAATTACATTATAAACCTTACCATTTCGCTGGAGTGCAGTGAGTGCACTAT
TCTGTGCTTCTGAATGGTTGTATTAAAATTTCTGTCGTGTTTAGTCTGGTGCAAACAGCCAAATTGCTCA
TCACTGCAAATCTCGTTTTGCATCGTGTTTTTAGCACACAGGGTTACAATGTGACCTGCTCACCTAATCT
TTACGTTCGTAATATTTATAATTTTTGCTAATTAATAACCACCTCATGAGGAACTCTGAATCTGCATCAC
ATTTCAGAGGCCTCTACTGTCCATGTAGGTCAAGAGCCTTCCTGACTGAATGAATGAAACGCGTTGTTCC
CTAACAAGGCAACCCGGAGTGCTGAAATATAATTAGCTAAACAGGCATTGGGCGGGTTAAATAACCAAAA
CAAAGACAGACGTTCTGGCACGTAAAATAGTGCTATTCATGTGCATATACATACACATACATAAATATAC
ACACAGTTAAAGACTAATATAGTTCTCCCAAATGATGTTTAACAGAGGAAGGAATGTTTCACAGTATTTC
CTATAATGTTTTTTCTTGTGCAGAAAGTATTCTTTGATTTATTTTGGCTAGAATAAAAAGTTTTTAAAAC
CCTTTTACGGTTTATGTTATTATCCCCCTTATTAGATATATTTGTTTTGATTGTTTACAGAACAAACCAT
CATTATTCAATGACTTTACCTTATCTTGCCTAATTAACATAGTGAGGCCTTTAAATGTCACTTTGAGCTG
AATACTAATATCTTAAAAAATATCTAGTCAAACATTATGTACTGTCATCATTGCGAAGTTAAAATAAGTC
AGTTATTAGAAATTGTTGGAATATCAAGCAAATTCCACGTGTGTTCAATAAGAAAAGGTCTGGAGAGTGT
TTTCTTCAATGCCAAAAATAATAGTAATTTATTAGACACAAATGAATAATCGATGACAGAGCTCTGGGTT
ACGTTACCCCAATGCAATACAAGAAATACAATTAAGAGGTTCGCAGCTAACTTACATTTCCTGACTCCAG

CATATATACACAACTGATAATAACAGGTGGAGTTTTGGTTTAACGTCACTGATCAGTCATTCCGCAGTCT

CTTGGCTCTTTGTAACCCCTAGACATACTTCCTCTTTCTGCAACCCTTCTCTGCATACCAGAACTGAACA

ATTACGTGCATCTTTAATATATTTCAGAACTTCTACAAAGATCATGCTCCTTGTGACATGTCCAGACAGC

AGTCTGGAACAAACCTAAATTTATTCTTATTCTGCTTTTTTCATTAGTCAGCAGTTCTTCTAAAAAGTAT

GCAGCACAGTAAAAAAGAGGAAGTGTGTCTGGTCAAGATGTGTTATAATACAATAGAAAAGATTGATTAA

TCTGTTAGTCTAAACATTTTTCTAATAAACAATAAAGACACAAAAGTAATAAAAATAAATTCCTTTTTTC

CTACAAAATTAATAATTAAAACTATAATGTTTAGAAATGTGTTAAGTCTTTTGTCTGTTAAACAGAAATT

GGGAAAAAATTTTACAGAAGTGCTAATAAATCCAGATGTATACATGCATACGCACCTTTAAATATTAAGCA

AATGTCCTCTGTTCTTTTTGGTTCTTGTGTTGGTGCCAGGACTGGCCCTGCGCCCTGCCGCCCCTCACTC

AAGTAATTGCTTTGAGCTCGGAGAGAAAGAAATGGAAGTCAGATCGGCTTGGGTGACTGGCCAAATTATA

TGCTCGGCAGGACAAGACATGTCCTGCAAATACACCAGGGGCTCCGGCATTAATATTCAAGCCTTTGCAC

GCTGGTTTAGCTTACCGCAGTGCTGCAGATCACAAACATCACACTCGTGCACACCCTCATACAGTGGGTG

AAATATCAAGTGTTGAACGCATCACCATTTATCTCAGAAAACGTGTTTGAAGAGGTGCTGCCGAGTTGAA

ATTTCAGTAACACGCAACATAATCCGTACACACAAAGAAAACAATTAATTAGTTAAGAAATTAAGTTATA

CTCCGGGGAAAAAAAATTGAACACATGAAGATAGGAAGCTATAAAAGGGCATGGAAAGCCCAGACAGCAGC

TGAAATCTCTTGGTTTTAGTCATTAAGTTAGTGTAAATTAATATTAGCCGCTTCAGTCTCAACACCTACA

TTACGAGATGGTGAAGATGAAACCAGGGTGGACATTTTTGCAAGACGATGATCCCAAACACAGGCAAGGA

AACTCTGTTTGTTTCAGAAAAGAAAACAAAGCTGCTAGAATGGCCTAGCCAGTCACTTGATCTGAATCCA

ATAGAAAACGAGAACTAAAGATCAAAGTTGATGGATGACATCCACAGAAAGGTCAAGATTTTTACACACT

GTTGAAAACACTCACACCTGAGCAATGCATGGGACTTCATTCTCCATATGAGAGGCGTCTTGAAGAGGTC

ATTACCAAAAAATCCATAAATGCATTTCAGTAGTTCGACATTCATTAATGATAAACATGACTGCTTTTTT

CAGAAATGTTTTGTTTTATTTGTGTTGGGGTTTTTACCCAAGTCAATTTCATGCCAACACATCCTTTAGA

TATATCATGACGTCTTAAATGCCTGATGATGTATTAAATACTTAATATTTTACCTTCTGTGTTCACACAC

ACATCACTGAAAGAAGGACATGCAATCAGAGATGTTCATAGCACAACGTGATCAGCTATATCTTTTTATT

Mib **exon 10** TTCCTGCAGGTGAACGGGCAGTGTGCAGGACACACTGCCATGCAGGCGGCGAGTCAAAATGGCCATGTGG

ACGTCCTCAAACTGCTGCTGAAACATAGCGTGGATTTGGAGGCGGAGGTATGTGTCCTGCTCCTCAAACA

CCTCGGTTTTCCCTGAAGTTAATTGCATTTAGAGCGTTCACGTTTTGATCCTCAAGCCAGCAAATGCAGT

GTATATATATCACTTTCACCATGTAACAAACTGATTTATTATGTAATTAATAAACTTAAAAGTTCATTAT

AATTATTAAAATTAGGGTGCTTCATAAAAGTTATTAATTACTGACCATCTCATTAAATATTTAATTTAAA

TACATTTTTAAAATCTTTTGATAATTACTCCATAAATAATTTAATTACATGTAGGCCTGGCATGATTATT

GCACAACACGTGGACATGTCTTTATGTGGCCATAAAAAACAATTCTTGCCACATTTCAGCTGACTGTGTA

GCTTCTCTATCTCTATTGTGTCAATATGTCAGTGTCAGTATGGTTAAAGAAACACTGTAGTATTTACTAT

AAATTACTAAAGTGTATTTTCATGTGGGTGCCTGATGACTGAAAATAAATCTGTTACTTTTTTTCTGGTT

CTTTTTACTTTAGATGTTATTGTTAACATCATATAATATTTATTATTTAGAATAGCCGTTTTCACATTCT

GATTCCAATACCGTACTATTAAATAGTTAAAGTGACTATTATTAGACGAAATGGTCTTTAGTATAAAATA

TGATGTTTTTTGGAAGGGTGCACTTGCATTATGATGCTGTTATAGTGTCAAAATAGTCTTAAAGTGACAA

TAATATTGTTTATTGCAATACATTGTTGCTATATATCTGCCCTAGCTTTTTCTAATGAAGGCCCAACACC

AAACTTGATTAAGGCTAGTGGACTGAAGGTAAATATAGCTGCCATAGGTAATTTCCTAGTTTATTTAATG

ATGTTTAGAATAGAAAACATTGCTTTATATTAAGTAATACAGTTTATTTTACATTTAATAATGAAGTTAT

TACAACAAAAACAAAATTTAATTTATAACAGAGGTACACTAGTTTTTGCCTTGATTTACTCCTGCATAGT

CCTCTATCCGTAAAGGGACAGTGTTTACATTTGAAAAATGACTCATTTACAACATTTAATTGAAATATTT

AATTTCAGTTTTTCTTTTTTGGAGTTCAGCAATAAAAACAAAAACAAAAGGTTGTTTTAAATTAGAAATG

TTGATCTCTATATTGGCATTCACCCCAACCCTCTCCATCATACTCACTTATCCTCTCAGATGGGATAGTG

GGTAATATCAAAGGTTACAATGGGCTATTTTGAGCTTCTCTGGTGTATATAACTATGTATGTATTTATGT

ATAGTATGTGTGTATTGTGTATTGTGTGTGTGTGTGTGTGTGTATATATATATGTGTGTGTATATATG

TATATATATATATATATATATATATATATATATATATGTGTGTGTGTGTGTGTGTGTGTGTGTGTGTGTG

TGTGTGTGTATATGTGTGTGTGTGTATATGTATATATATATATATATATATATATATATATATATATGTG

TGTGTGTGTGTATATATATGTATGTGTGTGTATATTATATATGTATATCACAGAGCTTGCGCTACGTGAC

GTTGCAACGTGTAGTTACATTTTTTGAGAGGCGCACATCAGCGACGGCCACGGTGTAGGGCTATGCGTCA

ACGCCGTAGCATACGTGTGCGTTTGATGCAGAAGTATGAATCAACCTTTACACACATATTTTACTTAAAG

GTGTTTTCTTGTGGGCTATTGTATTTTCATTTGAGGATGTCTAGATGGTAGTGGTAGATTATAGTGGTCA

TGTGTCCACTGAATACATGTTTCTTGCACCAAAACAAAAATCCTAATTTAGAAAAACACTGATACTAATA

AAAACTAAACTAAAACTAAGCAATTTCAAACTATAAAAACTAGTAAATCTGCCTCTAAAAACTAATTAAA

ACGAACTAAGTTTAAAAGCAAAAAGTCAACACGAAATAAAAACTAAAACTAATGAAAAGTCCCAAACTAT

TATAACCTTGTACCAGAATAAGTATCTGTCACTGTGATGACTTGTTTTAATCTGGAGCTCAGTTTTACAG

TAATAAAGACTTCTGAACTGATGAAATTAAGCTGATGTCATGTAATGATTAAGGGTGTTAATTTTTTTAG

CTGTGTGGTCAGTGAGTACAGTAAAAGAGTAAGTTAGCGTTAGCATGCAGTCAGCATGGGAAGCTAGCAT

TGTTATTCGAGCCACGCGTGGGCTCACAATCAAGCCGGAATATTTATGTCCCGGTGTCTGGCGACGTTTC

AGGAGCAGAAAATGGCTCGTTGCTGTGGAGAACATCAGACTTATTTATTTTAATACCTTTAGATTTAGTT

TTGATTGCAGGCTGCCATTAATAACTTTTTCTCACATCATTTTAAAGGGTTTTGTTTGGATTCTGATTTA

CATTTTCAATGACAGCAAAATGTTTGTTAAAATCAAAAGACTTTGAAGGTGAAAATGATGTCTGATGTCA

TTTTCTTTACATTTACAAATGTTAAATATACATAAATGTATATAAAACAGGACAGAAAGTTATGCAGTTG

ACGTCATAACTATTAGTCCTTCTGAATTATTGGCCGCTCCAGGCAGTGAGTGACATGTGGCTCTTTTCCA

Mib **exon 11** GGATAAGGATGGAGATCGAGCTGTCCATCACGCATCCTTCGGGGACGAGGGTTCGGTCATCGAGGTGCTT

CATCGAGGCGGTGCTGACCTGAACGCCAGAAACAAGCGCAGACAGACGCCGCTGCACATCGCTGTCAACA

AGGGCCATCTGCAGGTGGTCAAGACTTTGCTTGACTTTGGCTGCCACCCCAGTCTGCAGGTGAACTATCT

>Zv9_scaffold3540 dna:scaffold scaffold:Zv9:Zv9_scaffold3540:1:281342:1

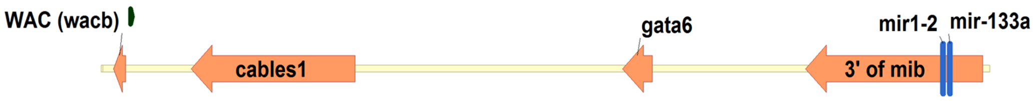


Loci (6 total)

*WAC* (*wacb*) Start: 3350 End: 7247 (Complementary)

*cables1* Start: 27985 End: 79954 (Complementary)

*gata6* Start: 164736 End: 174290 (Complementary)

3' of *mib* Start: 222692 End: 278952 (Complementary)

*mir1 -2* Start: 266415 End: 266501

*mir-133a* Start: 268599 End: 268697

#Genomic sequence was compared with *mib* mRNA sequence (NM_173286.3)

# **Yellow color**: Indicate the site of mir

# **Red color**: Indicate the site of Exons

# **Blue color:** Indicate the site of introns

# **Green color**: Indicate the site of polymorphism

# A← ：The variation on mRNA is A

# →G: The variation on chromosome is G

Mib **exon 12** CTGTATACATTACAAAGTGTGCAACTCTTTGCCGCCCCCTGCAGGACTCTGAGGGAGACACACCTCTGCA

TGATGCCATCAGCAAGAAGCGGGATGACATGCTCTCTGTGCTCCTGGAAGCAGGCGCAGACGTCACAATC

ACCAACAACAACGGTTTCAATGCGCTCCACCATGCAGCACTGCGAGGGAACCCGAGGTATGGCTGACTTC

AGATCTGACACAACTTGGCCTTACAGTTGAAGTCAGAATTATTCAATCCTTCTGTATTTTTTTTGTTAAA

TTTTTGCCCAACACATTTCCAAACAATAGTTTTAATAGCTAATTTTAAAAATACTAATATTCAGCTTGAT

GTGACATTTAGAGGCTTAACAAGGGTGAAAAATCAAAAAACAATATTGCTTAGGGGCTAATAATATTGAC

CTTAAAATGTTTTTTTTTAAATTAAAAGCTGCTTTCATTCTAGCCGAAATAAAACAAATGAGACTTTCTC

CAAAAGAAAAAAAAAATATTATAGGAAATACTGTGAAAAAGTCCTTGCTCTGATAAACATCATTTGGGAA

ATATTTAAAGGAAATTCACAGAAGGGTGAATAATTTTGACTTCAACTCTCCATTGGGTTATTTAAAGGTA

TGCTTCGTTGACTTTCTTAAACCAATTTCAGCTCATGTTTGGAGCTTGGCAATTTTCTGGTGTCTGACAA

ATCTACAAAGTATGGTATTTCATCTTGCGTTTGCACTGTAATAAAAAAAAAAAAAGACTGAACAGTAGAT

AAATAATAGATCAGGACTGCTGAGGTGTATGGGATTATTTTTTATTGTCTTTATCTGTCTTTAACGTTGC
TTTGGATCTGTAGTGCGTATATCATAATAGTGCTTGTAAGTTTTAGGATGAGACTGAAAATGTGCAAATC
AAGGTAGTTTAGAATGGAGTTTGTTTTTGTAAGTGCTGCTTTTCCATTGTACGATGTTGTCGTAATCTGT
CATAATATTATGCCAAAGCTGATATATCGGCCGATATTTGTCACATTTTATTTTACACATTTATTTTTTG
TACATTTTTCAAATCATTTAAAATTTAATTCAGTTTTAATTTATTTTATTATAAAATAATTATATTGGCT
AAATATCGGCCACCCTACTTTCCAATATATTAGCGTCAGCTTTCAAAAAACCCTTATCGGTCAGCTGCTA
TTGTTACACATATTTTACACAATTTTTTTGTCAAATAATTTTACAGTTAATTCAGTTTTTAATTTAGTTA
TTAAATAAAATAATGATATTGGCTGAATACTGGTTATTGGCCACTCTGCTTTCCAAGATATCAGCCTTAG
CTTCAAAAAAAAAAAAAAAAAACATATCGGTTGACCACTAATGTTAAACACAGATATTTAACACTTATTTT
TTGTCAAATCATTTTAAGCTTGATTCAGAATTAATTTAGTTATTGAATAAAATAATATCGGCTAAATATC
GGCCACTCTGCTTCCCAACATATCAGCATCAGCTTTCAAAAAACTCACATCAGTCGACCACTAATGTTAA
ACACAGATATTTAACACACTATTTTTGTCAAATCGTTTTAAATATAATTTAGTTTTAATTTAGTTATTAA
ATAAAATAATAATATTGGCTAAATATCGGTTATCGGCCAGCCTGCTTTCCAAGATATCAGCCTCCGCTTT
AAAAAAAACATATCGGTCAACCACTAATGTTAAACACAGATATTTAACTCTTTTATTTTTTTGTCAAATC
ATTTTAGATTTAATTCAGTTTTAATTTAGTTATTAAATAAAATCATAATAATATCAGCAAAATATCGGGT
ATCGGCCACCCTGCTTTCCAAGATATCAGCCTCCGCTTAAAAAAACAAAAAAAAAACATATTGGTTGACC
ACAAAAGTTAAACACAGATATTTAACACATTTATTTTTTTGCTAAATCATTTTTAAATTTAAATCAATTT
TAATTTAATTATTAAATAAAATATTTATATCAGCCATCGGCCACTCTGCTTTCCAAGATAGCATCAGCTT
TAAAAAAAAAAATATATATATATATATATTATTTCCTAATAAAATCAAAACATTTTTGCTGCATTTTTGT
GCTTTACAGAATAACGGTGTCCAGTTTAGTCACAAATCCCTCAGTGAAACGCTCAATCTTAGCTTCAATC
TCCCAATGGCAACGTGGGGATCCTTTTTATTGACTGTGTAAATGAAGAACTGCCCCAGGGTTGGTATTTA
TGGGCCGCTTGTTCAGTAGAGCCTTGCTTGGGAGCAGAGCTTGTCCCTCCGCCATCAGAAAGTCCTAGAG
ATATACAGCTCTCCTTCAGCTCACCATTTATTTGTGTTTTTCGCATGAGGTCCCACTGCTGCAGTCTGCT
CATTCCTCTGCCAAACTCACACTCAGTCCAATCCTGAAGCATAGACTCGAGTGCACATGTTTCACGTCTA
AAAACTGTTTTTCTAGGCCATGAAAAACACTTTATTGAATATTATAGTTGTTATAGATGTTACTATTTTT
TCTACCAGTTGTTACTATAGTAAAGTGTATTTTTTCCATTCCAAAATATAAAAATGGTAAAAAAAAGTGT
CTAATATAAGACTTAATATAAGTCTTCTATAATATTATTAATAATATTATTATTGTTATTAATATTAATA
TTAATATTAATAAGAAGAATACTAGTAATAGTAATATTAATTAATATAATAATAATAATTATTATTGTTA
TTATTGCTATTATCATTATTGTTATTTTTCTTTGTAAAGACAATTAGATCATATTCTGATTTAGCCCTGT
TAAATAAAATTAAAAATTAAATAAAAACTCTAAAAACACCAGTAGGTGGCGACAGTCATTAGCTCAAGTG
ATTCGTTCACTCCTTAATTGAATAAATGTGTTCAAAACGGACAATACATTTAGAAAATTATCAAGTTGAC
TCTTTACAAATGGATCATTGATTTGTTAAAGACAATTCTGGAGGGCCAAGAAAAACACTTTAATAGAGAA
GTAATAGAGAGCCGTTATAGATGTTACTATTTTTTCTACTAGTTGTTAATATTGTAAAGTGTAGTTTTTT
TTTTTTTTTTTACAAAATATAAGAATGGTAAAAATGTTGCTAATATAAAAGTACTATACTACTATACTAT
AAAACTATAAAATACTACTACTACTTTAACTACTACTACGGTGTGTGTTAGTTCCAACTGTGATGTGCGC
AGTGCATCTGCTGTGCTACGCCCATAAGAATGGAGTCTGCTTTAGGTTTATGGATCAGTGACTGCAGGAT
AAAGAACGTTACGCTGGATGCCTACATTATCCGCACTAAATCTAAAAAGCTTTATGAACCTTTCGCTGAC
AGCGATGAATGTTTGCGCCTGATAACAGGTTTTGATCTTTGGTTTTATTCTATAATACTGGAGTTTGTTT

TCTACAAAAGTTTGAACTTTGTGAGTGTTTAAATGAGAAAAGTGTGAAAATGTAAAAGCCATTTTTGTGA
GAGTGTAGAAAGTGTGTAGTGAGGAGTTTTACAGCCTTGAAACATCTAGAATAATTGTAAAAATAAAGCG
GACTACTTCGCGGATTTCGCCGATTGCGGGTTATTTTTAGAACGTAACTCCCATGATAAATGATGGACTA
TTGTATTTTAAAATGTTTGCACATCTTGCCATTTCTGTTTTTTTTATGATAATCTGTAAAATAATTTTGT
ATTTTATGTCCTTAGTTTGTTATAAATAAATAAATAAATAAAAGTGCACCAAAATAAATAAAATAAAATA
AATAAATAAACTCAAAAATTATAAATAAATAAAAACGATTAAATTAATTGCAAATAGATAAAATAAAAAA
TAAATAAAAAAAATAAATAAAAAATAAATAAATAATAAAAAAAAAATTAACAAATAATAATAATTATTAT
TATTTAATAATAAATAAATAAATAAATAAAATAAAAAGTAAATAGATAAATTGAAAAAAAAAAGATTTAA
ATACAATTTAATTTAATTTAATTGATTAATTATTATAAAATTTTAAAGTACACAATGCGAGCCAAATAAT
ATGTTGAAAAACAGTATTCGAGAATTACCCGGATGACCTACTACTTCCAGCAAGATTCTAAAGAGCGCAT
CTAATGCATGCTATGCTATCCCATGCTATGAATGAGAGTGAAGCGATGCAGCTGAAGCGGGTAGGTCACG
TGATCCTGACAAAAGGGTGGATGTAGTATGTCCGAGTTCCATTCATGCTACTCACATTTATACTGTTTAG
AACATACTTTTCGAATGGCTGTGTAGTTAAATGAAATTCAAATGCAGTACCTACTGAGTAGTAGGCTGTT
TCGGACGCAGCCTTTGACTTGCTATGTGTGTCCACACAGATTTCTACATTCCATCTCTTTCTGCTAAATA
TTTTAAAGTCTTTATTGAATATTGCATTACCCGTGTCTATCTATGCATTGCGGATTAGCACCCTGGTGGT
TTTGGAAGTTGTAGTTGTTGTTTTTTTCATCATGTGTTTATGAGAATCCCAGGCTGAGAGCGGGACTGGT
TCCTCTCAGTGAATCTCTGGAAGGCAGTTGTGCTCCAGCACTGTAAACAGTACGATTAATAGCCCATGAC
TCATAACACAGTCCACAAACAGGCCCCGCTGAGGCCGTCGCAGCACTACTGTACAGATCCTCCAAACTGT
TTGTTAACCGCCACGTGGAGAGTCCCACCCTCAGCGCACTGTTTACTGCCTCTATTACAGCTTTATAGTT
CCGGCTTTAGCCAAATAGTCACATCTAATGTTGCTCATAGAACTCGCTATTCGTTGAGGAAGAGGTTTGG
CACCCATGCTGATGATTAGAGGTTTTATACTGTTGAAGTAGAAATTATTAGCCCTACTGTGAATTTTTTT
TGTGTGTGGCTTTTTATTTGTATTTTATTTAAAGAAACACAAATATATAAATGTATGAAGGGTTCAACAA
CAATTTTTCTTACTGGCCCGATTAGACCAGTGGTTCATATTTTACTTGCCCTGCCAAAAAAAAAAAGTTA
ATTGCTATTTCTTAGCCACATATATTAAATAATGCATCACAAAATAATGTCTGTGAATCTAGAATTTAAA
TGTTTGAAAAAAAATTTAATATTTAGCAGAAGTTTGGAAAATGTGCAGGTATTTTATTGCAAAACAAAAA
GTGGCTGACGTAAAAGTGACAGCAAACTAAGCAAAACATCATTTCAGTTTTTTCATAGTGTTGTACCCAT
GTAAATGTTTCCACAACGTTATAAGCAGACGTTTTCTTTTAGCCTCATAATTTGATGTTGCGGTCATGTT
GCAGTCTATTAAAATTTAGTGACAAGGCAGAACTGCCCCAATCAGGCCAGTAATGATTCTGTCTGCTGTC
CTGAGCATCTCTCACACTGGCCCCGGGCCATCAGGCAGTCCTTATTGTTGAGCCCTGGTATGTAATGTGT
GTGTGTGTGTGTGTGTATATATATATATATATATATATATATATATATATATATATATATATATATATAT
ATATATATATATATATATATATATATATATATATATATAATTACATTTTTAAAAACTAATAAGTTAACTTTTGC
TTTATGTTGGCCCAACTCAAATTGATTGTGTGGAAACCAGCATTTCTTACAGTGTAAGCACACTCTAAGA
ATTTTATTTGCTGTTTGTTCAAGCTACATACATAAAATGAGCTGCAACAACACAATTCTTGAGGGTTTTG
TTGTGACAGCCTATTTGTTTTACAATTAATCCACTTAAATTTTCAAGAACTAATACGTTAACATAATTCC
TTTATGCTGTCCTAACTCAAACCGATTGTGTGGAACTTTGTGGAGCATTATTACGGTGTAAACACATGTA
AAAAAAGTTTTGCTGATTGTTCAAACTGCTTTTATAAAGTAAGATTAAACAACACAATTCTTGAGTTTGT
TTATGAAAACCTATTTATGTTCAAGCTAAATTTTTAAAAACTAGTAGGTTAACTTAATTCCTTCATGTTG
TCCCAACTCAAATAGGTTGAGTGAAAACCAGCATTCTTGTAAACACGCTCAAAAAATAACTTTTGCTGTT

TGTTCAAGCTACTTATTTAAAATGAGCTAAAACAATACAATCCTTCAGGATTTTCTGCAACAACCAATTT
GTTTTATGTACAAGCTAGTTACATTTTTAAAAACTAATACATCAACTTAATTCCTTCATGTTGTCCCAAC
TCAAATCGGTTGTGTGGAAATCAGCATATGCGTGTGTAAACGCATATAAAAAATAACTTTTGCTGTCTGA
TTAAACTACTTATTTAAAATGAGCTGAATCGACACAATTCTTGAGGTTTTTTAGCAACAACTTAATTGCT
TTGTTCAAGTTGCTTAAATTTTTTTAAAACTAACACGTCAACTTAATTCCTTCATGTTGTTCCAACTCAA
ATCATTTATGTAAATCCCAATAATTCTTTACAGTATGAACTCGCTCAAAAAAACACTTTTGCGTTTTGTT
CAAACTGCATATTTAAAATGAGCTAAACCAACACGATTCTTGAGGGTTTTTGCTACAACTTAATTGTTTT
GTTCAATCCACTTCAGTTTGTAAAATCTAATAATTTAGCTTGATTCCTTTATGTTGTCCCAACTCAAATT
TGTGTTTAACCCAAGTTTTTTACAGTGTAAACACTCAAATTACTTTTGTAGTTTGTTCAAACTACTTACT
TAAGCTGAAGCGACGCAATTATTAAGTTTCTTTTTCTGTTGCGAAAAACTTAATTACGTTCAATCCATGT
AACATTTTTAAATGAATGTCTACTTAATTCCTTCATGTTGTCCCAACTTAAATTAATTATGTGGAAAACA
GCTTTTTTAGTGTAAACGCGTAAAGTCATTTTTGTTGTTTGTTTAAGCTACTTATTTAAAATGAGCTGAA
GTTTAAGTTAACTTATTAGTTTTTACAAATTTAAGTGGATTGAACATAAAACAATCAAGTTGTCTCAAAA
AACCTTAACAATTGTGTCGATTGTGTCGAACGCTGCTTTTTTGCAGTGTAAACACTCAAAAAATATCTTT
TCCCATTTGTTCAAGCTACTTATTTAAAAAAAGCTGAAATTACACTATTAAGTTTTTTTCTTTTGCGAAA
ACTTAATTGTTCAATCCACTTAAATTTGTAAAATATAATAAATTAACTTAATTTCTTCATGTTATTATTT
TTTGCCGTTTGTTCAAGCTACTTATTTTAAATAAGCTGAAACACGATTGTTGATGTTAACTCGTACGTTA
ACTAGTCATTTGTATGAAATCCAGCATATTATTAGTATTATTTTTCTACAATGCATGTAAAATTCCTCCC
TATCTTAATGAAAAAGTCTTGTCATCCGCTTCTGCATACAATGGATGTGGATGTATGAGCAGCACAGGGG
TGATCAGCGTGACATGGTGGCTGATCAGTATAATAATGTGAGAAATTATGATGCAGCGTTGCAATCCATG
TGACTCGGGGAAGGATGCTGCTGTGATGCAGAGGAGAGGGAGGTTCACACCCGCATGCCTGCGCTGCGTC
AGTGTGCCAGGGGCCATAATTGGTGGATTTTTCTCAGTGTATGATTTGTGTGTACACCCCCCCCCCCCCC
ACACACACACACACCTAGAAGCATCTGGCTTTCATCTCAGCTCGTCTGTCTGCAGGCTGAGACGGGCTAC
CGAAAGGCACACGGGCTACCATTCCCACATGTGTCGCAGTGAGCTGGTGCTGCAGACGCTCTTCTTTCCC
TCTCCTCTGACTTTCTTTTGCTTTCCATTTTCCCGAGTGCTAGGATATTCGTTTATAGGGATGCCAGTTT
ATATGTCAACTTTACACCCCCATTGCGTCACAGTGGCACATTTTAAGAACTTTTTGTCATTGCTTGCTTA
CTAATGGAGATGGATGTGTAATAGGGCTGCACAGTATATAGTGATAGCATGGATTTTGCATTATGGAGTC
AGCATTACAGTTACAGCAGAGAATTGTGGAGTCATTTTAGTTTTAACATAATAAATTGAAAGTGTTTTTT
ATAGCATGTCAAGTTTAAAAGCATTTGGGCACAAGGTATAAATTATTTTGTAGCTTTAACAAAGATTAAT
TGCATTTATTTAAGGAAATAGAGCTGCACAATATATCAGTTTAGCATTGATATTGCAATGTGCGCATCTG
CATTAGTCACATCACGAAGATATGCAATGTCCAGTCTGGATCATAGTTGACTAGGAGCTACAGAATTGTA
TGTAATCACTGAATTCATATGATTTATCAAAAATATATATATTTTTTAGTCCAAATGTCTGCAATTCAAA
GCAAAAACAAGAGTATTTTTAGTTGTCCCAACTGAAATTGATTGTGTGGAAACCAGCTGTTTGATCAAGC
TACTTATTTTTTTCTTATTTTTGTATATTTTTATACTTTGAAGTTGATATATTACATATTTTATACATGT
CTAACATGCTTTGGCAATACTGGACAAAATGTCAGCAGCAGATTTTACCTGTCAGTGGATGCGTATGGCT
CCTGAATAGAATTAAAGTAAAACAAATAGTGGATTTATTTCATTTAGGCAGTCTTCTGTTAACTTCAATC
ATATTGAATGAAATATTTGTAAAAATTATATTTTTTTGTCGCAGGTAGGTAGCTTTGTGCTGACCCACAC
GGAGTCTGCGCGTGCAGAATTTTCGCAGATTTTTAGCCCATCATTGATTGTTTATTTACTTTATATATTA

TATTTGTCATATTTATTCAGTTATAAATTAAAAATGGTAATATTATTGACTATTATGAAAATATTCATAT
GATTTATTTACAATACAGCTTGTAAAGTAATATTTTCTGTCTTTTAGTAGATATATTATATGAGAGACTT
GCTCTGTTTACCAAATAAAGTGGATCTATCACTGTAAAAAGTAATATCTGCATATCTGTGTTGAATTTAA
ACAAACAAGTACAATTGACTAATGTTCAACTTAATTTGTTTGTTTTAATTCAGCCCAAATAAATAGTTTG
CAACCACTTAACTTGAAAAATAATTTGTAAACTCAAGGAATCATCATTGAATCTCTTTTTTTATAAGTGT
AATTGGATTTGCATTGTAAATATTAAATAAAAGTTAAAAAAGGTATTACTTTTATTTCATATATTAAGGT
TTTAGTTATGATACTCCCAAAATAAATCAGCATAAATCCGCAGATTTTTAACACAACTTTGCGCAGAAAA
AGCAAAAAAATATCAGCAGATTCCATCTGGACCTAGCAATGGCTCCTGAATCAAATTAAAATAAAACAAAT
AGTGGATTTATTTTATTTCAGCAGTCTTTTTTTTAACTCAAATCCATTGAAAATTGTATAACACTGTCAT
ATTAAATAAAGGATTTTTAAAAAAAAATCTTTTTTTTTTTTTTTGTCGCAAGTAGGTAGCCATGTGCCGGCC
CACACGGAGTCTGCACGTGCAGATTTTCGCAGATTTTTAGCCCATCATTGATTGTTTATTTGCTTGTAAA
TTTATATTTATTCAGTTTTTAAATTAAATACCGTAATATTATTGACTGATATGGAAGTATTCATATGATT
TATTTACAATACAGCTTGTAAAGTAATATTTTCTGTCTTTTAGTAGATATGTTATATGAGAGACTTGCTC
TGTTTACCAAATAAAGTGGATCAACACTGTAAAAAAGTAATATCTGCATATCTGTGTTGAATTTAAACAA
ACCAATTAAATTTTAGTAATGTTCAACTTAATTTGTTTGGTTAAATTCAGCCCAGATAAATTATTTGCAA
CCACTTAAAAAAAAATTGAGTAAGTTCAAGGAATCATCTTTGAATCTCTTTTTTTTTGAGTGTAATTGGAT
TTGTATTGTAAAGATTAGATAAGTTAAAAATGTATTGCTTTTTATTTTATATATTAAGGTTTTAGTTATG
ATACTCCCAAAATAATTCAGCACAAATTCGCAGATTTTTAACACAATTCTGCGCAGAAATAGCAAAAAAT
GTCAGCAGATTTCATCTGGCCCTAGCCATGGGTAAAAGGTGTGTTTCAATCCCGCTACCACCTCAAGTTT
ATTTCAAACCTGTGGGACGCACTGATCTTGTAAAATCCCCTGTAAAGGTTTATTTCAGTATAAACATTTA
AATAGGTTAGTTAAGAAATGTTCAGACAGTCAAAAGTGCATTGCATATACATTTATTCAAATAAGTTTTA
TCAATTACAAATCAAATGTAATCTTGGTTTCCTGCGAAAGCAAAACAGGCAAGATGAAAACCGTTCATGA
Mir133a TGTCATCAAAGCATAGCTACAGCTGGTTGAAGGGGACCAAATCCATTGAAGAGGTGATTTGGTTCCATTT
TACCAGCTTTAGCAAAGCATTGTGGTTTGATGTTGAGATTTGCTTAACAAAACAAGAACTTGTATGACGA
TTAACAGATAAACTTGTGACAAGAAATATTGAATAAGCTCAGATACATCTATCGAGTAATACATTTCACT
GCACTCTAAACGTTTTCCTTCTCTTTGGTTAAAAAAAGAGAGAAATGCACTCAGCAAAAAAAGATAATAA
TCCATCGTGCCGCACTCCAGTCAAGTGTCCACGAGTCTTGTAGCAATTACAGCAGCTGATGGATTAAGCC
TGCTGAGGACAGAGTGTCGCTTGGCCGATCAAGATGTCAGCATGTGCTGCTTTCTGTAGTTGTCACTTTC
TAAATTTTGAAGAGATTCAGTCATACTCTGCTACATCGCATGGCTAATTTGCCCACTTAATCCAATAAGG
AAGTGTGGTGGGAGCTTCTCTGAGATTGATCATGACAAATGTAGATATTTGTACCACTTGTCAGTCACAA
CACAAGGCTGAAGCAACTCCAGAAAACAATTCTGAGTGCGCTGCAAGCCATACTTTTGTAACTATACGCT
TAGAGACAGCCTATTTAGCTTCTTAGCAGGTGGCTTGCTTGGACCCAGCAAGGGGTCATTGCTGCTCAAT
ATGGCGCTCATTAAACGGGGGTCAGTAAAGTGCTATTTACACCACAGATCCCCCTCCCTCTGTCGAACAG
ACACTCGCTTGCGGCATTCTGAACAAAGTTCGGCCCCCAAATACACTGGCCATAAGAGGGCAAAAGCCAG
GAGCAGCAGCTGAACATTCGATAATGTCAGCATCTGCTGTGTGTGTCACTCAGGCTGGGGTTGAGGGCGA
GAAAGAGGGAACACAGCCACGCGACTGACAGGAGCGAACCATACAGCAAGCAGTGCACAAAAAATGGAAAG
AGCACAGCCCTACAGAGGACAGATGGGAGTGTCTGACATTCAGAAAAATGGGCCACAACTCTCCCAGGCT
CCAGTCATGACCAAAAACTCTTACACCGTCTTATGCAGATAACACAAGGTGAGCAAGTAAAAGGTTTAAA

ACGCAAGCGTTTGGAATTGCTGCACTGCTCTAGCATAAGCCATTGCCACAAATCAAATATCACCAGAACA
GATTTGGTGGTGATTTGAATGCTAACAAATACCTGGAGAAAAGCTCTCAAAGCCCTAATGTCTTTGATTT
ACCTAGATGATCACCTGTTGAGGGTTAGCATTCAGTTGTATTCCTCGTGCACTTGTTTGCCAGTCTGCAT
TTGTTTCAGAGTGATCGCTATTGTAACCTGCAGTCAAATAGGCCCAAATGGATGTCCGCCTCTGAAGGCC
CCCGCATATCTTTTGTAGAGGACAATTGGAATCAGCTGACAGGCGGACCCAACTCTGATCCTGCATTGTC
ATTTTTCCATGCTCTAATCTGACTACTCTGTGGCCCAGCAAAGCATTTGGCAAGCCTAGCAAACATGAAC
AATGGGGACACAAATGGGTGGCAGGGTAAAACAGACAACCGTGCTGTCCTGCAATGTTAGAGAAGCTTCC
TTTTAGTAGCTGAGTGCTACCCCTCTTTATAAAGCCGTTAGCTCAGCAGCTTCCCTGCAGCGTAGTTCAA
CTCTTAAAGGACACAAACGAGTTGTCCCGAACTTACCCACTCTAGGCAAGTTTGAAAAGATTAGGGTATT
AATGGTTGTGACGCATGCCACTCCTGCAGAAACAGGAGCATAGTTGCGTTTGGCTGCAGTTCAAAGCGTA
GAATGCAGCAAACTCTCCAATAATCAAATAAGCCTCTGTTCTTATGGTGAGGTCTCAGGCTGTCATGAGA
TCTTTCAGGTTGCAGTGACCGAACTCATTCAAGAAAGTGAACCCTAAAACAATCATTGTAATGTTAGTCA
ATAAACTAAAGCATGCCAATCTTGCTGATTGAACTATCAAGTATTTCATTTTCTAAATTAGGGATGAGCG
TTGTGGACTGCTGGTGAAGTTACTGAAAGAGCAGCGACTTTTCTCAGAATCCACTGCTGCAGTAAGTTCT
GGCGCCCTGGTTACACAATTCATCACACTCTCTCAATATACACACACACACACACACACATTCAGTGATT
Mir1-2 CGGGGAAAGTTTACAAACTGAGGATCAGCACCTGACCAAGAATACATACTTCTTTACATTCCATAGCTTT
TATATGTTCATATGGGCACATAAAGAAGTATGTACACCAAGTAGGCAGCCCCAGACACAGCTGAGTTTTC
TTACCTTCGTTTTGATTTGATGTTTGGTTAGCGTGTTAAGTCATCGTCGATAGATCAGTGGCGCTTTTTT
TTTTTTTTTACCCTTCTGCACAATGCAGTGGATTAGCATTATACATAAGGTGACTTTCACAGATTCTTT
AGGTCACGTTTTCTTTGCCTCATCCCAACCTTTTTTTCCCCAGTAAATAAATATTCAAGTTTCACAATCT
TGTATCTTCTGCTGACCTTTTCTGTCCTCAAGAAAGTGGGGGCTATCCGTGAGGACGTTTTAAAAAGCAG
TGCCATAGGTTTCTTTTGGTAAAAAGTTGTCTCTTTTTCTGTATTTGAGGAAAAAAATATTGCTGAATAG
TAGTGTTCTTCTGCAATCTCCATATTCATGAAACTTCCAAAAAAACTCCTTCAGAAAAAAAGGTGCGTTT
CTATGTAAAAGTGTCCTGAACACATCTCCTTCCCACTCGATGTCTGTAGTTCAACCATGTTCTGAGGTTG
TATGTCAGACTGGTTGCTGAAGTCCAAAAATGAGGTTGATCCAAGTGGAACAGGAAGCTATTGTGAGCAG
GATTTGGGCTTTAGCCACACAGACCAGGCCAGAGGTCACATGCAAGAGCTAATGCCCACTCAGCAATAGT
AATACGGAACGAGTGTGCTGATTGCTCTTTCATATTTCTTCTTTTTAACCCTTTTGTTTTTTCAGGTAAA
CCCCCCTATCCCCCCCAAATCCTTTTTTTTCAGTTGCTCTGGGTGCGTGTATTCTCGAAGCTTCTCTAAT
ACTTTGTGCAAGACATCAATAAATGTTTGGATGATGGCATGAAATGTTCAGTAGCTCTCTAATGAATCTT
TGCTCCAACTCCACACATTCCACAAGTCAACACCGCCCCGTCTCTTCCCCCTCCTCTCAGGAGCCTATCC
AACACCAGGCCATAAGGAGAGGTGCACGGGTGGAAACATAACAGAAGGACACTTAGGCGGGCTATTCTGG
GCAGGGGTGGAAATCCTGGATGAGTTTGTGGTGCTTGACGGGGTTAGATATGATGGCAAGGTATGGCTGG
ACTCTTGTGGCGCCACAGTGTTAGTAAAGATTTAGGGATGAGGGTGTGTTGAGAGGGGGAGACGGCACTG
CAGCACTACAGCCCTGCCTAGTGATTGGAAAGCATGCAGAGAGACAGAGAGAATCTGGGTTACTACCCGG
GGTGTTGTGGGTTCACTCACTGACACACATATCCAGCAGTCACCGAACCGCCCTCCCCAGAGAATTAATA
TGCCAGCACATCTGTTTTTCCATCAATCCGATTCACCACCACCCCCCCCACCTGGCCTGAAACTTGCGT
CCCACCGTGACACCACGTCCCTTCCTTGGCTAGTCGCACCTGTCCCCCTGCTGAATCGGAAACAAGCTTA
AAACACCAAATATGGTTCCAGTAATTTGAGCCGACCACCAGAGGAATGAGTGTTGGCAAAGTGTGTAACC

ATCCCGAGTTGTATTCCATCTGTAAGTGCCTCCGAGTCAAAGGATCCCAAATAAAAGACGTTTCGGGGAA
GTGAGTTTCAGTCCATATTTGAGATGTCTTCGGCGCTGCAGCCACGAGGAGGTAATGAACCACAGAGTGC
TGTGCTGCAGCAGCCTGCTGCCTGACCAGCACTGGAAAAGCAAGAGACAGAGAGAGAGTGAGGTAGGGCA
CAAGAGCGAGCGAGCGAGTGAGCGAACGAGGTGGAGACAGTATTCTGGTGAGCAAGCAAGCAAATCCATG
CAGATGACAGCAACTTTTCCCTCATTCCACCTACACTTGTCTGTCCGGCTGTTTCAGAAATCCCTGATTT
TTTTTTTCCTTGTGAGGGGGACTTACATATCCTGAGGTGTTCCCATAGAAGTAGTTTCCCCCCCTCATGC
TGCTGCAGAGCTGTAAATTCCACTCCCTTTCACCGACCATGCCGCACACCGCGGCTGACCGGCCACGCGC
TTAACCCCCAGTGCATCCGAACGCAGAAGAAAAAAAAGCAGAGACGTCAGTGCGGACCGGCCGGCATGGG
GGGCAAACTCCCGTCCGGAGGGAGGAAGGCACGGGGAGCATATATTACCTGTCAGCTCAGTTTCCTCCGC
TCTCTGCTCCTCCCTCTTTCTTCTCTCCTGTGTGTGAAGAGCCATAATGACCTCGAGTTCTCCAGCTGCT
GCAGCCATCGCATGCCCGACTCGCTGATTCAGCTTCTCTTACGGGGGGTGGGGTGGGGGGAGATAGTGAA
CGAGTGTTTGTGGAGAAGGAACAAGAGTGGTGAAGCGGGAGGGGTGGAGCTGGGTGAAGAAAGCATACTC
ACGGCTTACATTTAGGCAGGGATAGACATCATGCTGACACGGGGAGCTGTCGCTGCTGCTCTAAAAACGG
GCAGAAAAAAACTGCAGCACTTTCACCTCCTCCCTCCCATCTCCAGCATGTGCATTCTCAGTGAGTCCTG
TGAAGATGTGCATGACTGCTGTAACTCCCTCTCCAACGCTTTGGCTTTTCGGCTAGTTTATTTCGGATGA
GTTTTGAATCGTTTTCAAAGAGAAAACCTTGCTGTTCTTATTTGGGAATAAAAGTCATCAGCAGAGTACG
CAAGTTGATGCTAATCTAAAGCTTGCAGATAACTTCATTAGACGCTGTGGTAACACTTTAATATAGGGAC
CAATTCTCACTATTAACTAATGGCTTTTACCTGCCCATTATTACGACATTGGCTCTTTATTGGTTCTTAA
ATAGTACATATTCTGCATGATCTACATCTCCAATAGCCCAAATACTACCTTATTAACTATTAATAAGCAG
CAAATTAATATCGATAGTTGTATATTACAATAAAATGTGTGATGGAAACCATTTTACCCATTGTTGGTAA
ATCTTGTCTAAGGTCACCAGAGGTCATGTTTTTTGTTTATTGCTCTTCTGAAAGTTATTGAAAGTGAACG
AAGAGCCACTGATCACAAAAAAAGCCAGCTGTTGCGCCTTGAACAAAATCATTAGCACTTTCCAGCAAGG
GAAGCATTTGAAAATCAGCTCCTCTTCAGTGTCTCTCTCACCTCCATGTGACCATATTTAGTCAACCAGT
GCAAAAATAACCCCCCTCCTCGCACCAAGGCAGCTGTCCCCCCGACAAGATCTGGGAAGTCTCCCGTTCA
GCGTTCCACCTATCGATTGTGGGTCCAGCTCTGCGAGCAGCTAGCCTGCATTAGCACAGCACTGCCTTGC
CTGCCTGCTATTTGCTGTTATTTTTAGTAGCACCAAATAAAGTGGTCTCTCATGGTTCCACTTCTATTTC
CCTCACTCCTTTCCCCTCCTCTGCTTTTCTAACACCACCCCCCCCTCCACCCCCCACTCTTATGCATCTA
AACACCCCCTTCCTACTCTGTTCTCTTTTAAATAGTGCTGGAACATGCTGCATATGGTAGATCTTGCCTC
TCCTCAGTACTAAACTTAGTATCATATGCAGTTAGGTGTGAGATTACAGGAGCAGCAAGTAGTGCTTGAA
GCTTTCTGAAAAGTATGTAATAGAGTGCAATTTCAATCAAACCCTGACACTAGCTTAACTCTTCATCTAC
AACTGTCAAACTCATATAGAGCAAGGGGTTTATCTTGGTTCTAGGACTTGCTGGAATATCGCTCAAGGTT
CATGTTTGTATTGTTCCCTCTTGAGCAGGGGTGCCCAAACTCGATCCTGATGGGCAGGTACCCTGCAGAG
TTGAGCTCCAACCTTAATTAGACACACCTGAACCATTTAATCAAGCCCTTATTAGGTGTATTAGAAACTT
TCTGGCAGTTGTGTTGAAGCAAGTTGCAGCTGAACTCTGCAGGACACCAGCGAGTTTGGGCACCCCTGCT
TGTTAGATTTTCAGTCATTTTCAGGGTCCACATTCTGGCTAACTGAGGCCTAGTCTACATGGTAACGGAT
TAAATCGATTTGCAAAAAAAAAAAAAAAAAAAAAAAAAAAAATATATATATATATATATA
TATATATATATATATATATATATATATATATATATATATATATATATATATATATAAAAA
AAAAAAAAATATAAAAAAAAAAAATATATAAATAAATANNNNNNNNNNNNNNNNNNNNNN

NNNNNNNNNNNNNNNNNNNNNNNNNNNNNNNNNNNNNNNNNNNNNNNNNNNNNNNNNNNNNNNNNNNATA

TATATATATATATATATATATATATATATATATATATAATTTATATATATATATATATATATATATATAT

ATATATATATATATATATATATATATATATATATATATATATATATATATATATATGTATATATGTAT

ATATGTATATATTTATATATATAATATATAAATATAAATTTTATATAATATATATATATATATATATATA

AATGTTGGCCTTTTGACCACTAAACAGAAACATTGTTGGTACCCTGAAACAAACTTCAAACTTTTTGATT

TGTAGCTATGTGGCTACAACAAAACATTTTGCAAACAATTACGCCATTGTACCATGTTACCAGAAAAATG

AGGAACCCTGCACACGTAGGAACCAGTAACAGCAACAATAGTGGATTGCAGAGATGTGTTTGTGCTGCAG

GCACCGCTAAGTTATTAAGCACTATTACAGCAGATCCTGGTGATGCTAAATCAACTAGACAACCATTAGA

GACACATTAACAGTTACACATTAGCGATGTATTACACCAGTTTAACCCATTTTTTTCTGTTTCATCTGAA

AGCAGACATTTCTCAAAGGGATTCACGTATGGATTAGAAACCTTTGGAATCTGCAAAAGAAATAAAGATT

GTTGCTGTGTTTACGTACTTGTATTCTGCTTTTATACTTCAGTTTAAGTCCAGGTGTTCTACATTGAACA

GTAAGTGTTAAAGTTGTATGTTTTATGTTAAGTATAATGTATATCACAGGCTTATTGACAGCCTGGCGTG

AAGTGAAGCGTTGTTGTATGTGACTGAAGGGCTTTGTTCTATAACAACCGCCTAGATGTACTTTATCCCA

CTTATTACATGGGTACTTGCCAATAAATGTAAAAGTTAGACAAAAACAACCACTTGAATGTACTTTATCC

CACTTTTTACATGGGTTACTTGCCACAAAATGTAAAAATTAGCCGATAACAACCACCTGGATGTAGTTTA

TCCCATTTTTTACATGGGTACTTGCCACAAAACATAAAAATTAGACAAGAATAATCGCCTGGATGTAATT

CATCCCACTTTTTGCATGGGTACTTGCCACAAAACTTAATTAGACACTAACATCTGCCTCTGTACTTTAT

CCCACTTTTTACATTGGTACTTGTCATAAAGCGTAAAATTAGACCATAACAACCGGCTGCATATACTTTA

TCCAGCTTATTACATTGGTACTTGCCACAAAATGTAAAAGTTAGGCAATAACAACTGCCTGGATTTACTT

TATCCCCCTTTTTTACATGGGTACTTGCCACAAAATGTAAAAATTAAAAGATAACATCTGCCTGTATGTA

CTTTGTCCTATGTTTTACTTGGATATTTGCCACAAAACGTAAAAATTAGACAGTAATAGCTGCCTGCATG

TACTTAATCCCACTTTTTACATGAGTACTTGCCACAAAATGTGAAAATTAGGCAAAAACAACCCCTCAGA

TTAACTTAATTTAGATGAAACAGAAAAACTGGGTTAAACCATTGTTATATATACTTAATGTGTAATCTCC

GCTGGTTATCTAGTTAATAAAGCAGACATTTCTTAAAGGGATACACGTATGGATGTGAAACTTTTAGAAT

CTGTAAAAGAAAAATGTTGTTGCTGTGTGGACTTAGCCTGAGGCAAAGCCACAGGAATTTTTAATGAGGA

GGTTCCATCATTCAGTGTGGTGAGCTGGTTTCAGCCATCAGCGTCCTCTGTCATATGACTGCTTTGTCCT

CCTCTACTCTGCTCTCTACCTGCAGTAACACCGCTTCCTCTGCATTGTACCCACCCCCACACAGAAATAA

TTACGGTGCTGAAGGCGCTATCTGATCTCTCAAAGTAATGTTTTTACACCATTTAGTGTAATGTCTCTGT

TAAATAATGTAAATCTGTAGTTTGAAATGGTCCTGAGGCTTCTCCTTTTCCACCTTGCTTGCAGCAGTCT

TCGGCAAGTTTTCAGGGATAACATTCATAAAGTCACTTTAAGCACTGTAAAAACATCTCTAGATAAAAAG

TTGAAAATCCTCAAGACTTTCAGAGCTCTTGCCTTTCTTTTCGTAATTTTGATGTACAATTAAATTAAAA

CCTTTTGCAACCTTTTTAATGTATTGGCATTCAAATGCTCTGTATTTATGGCCATTACTGAAATTGATCC

TTTGCCAGGTGTAATGCACTCTGAGTAATGCACTAGCTTAAGGCCCAACTTTCAGCCAAAAAGAGACACA

TTTTATAATTCATGCAGCACAAAAAAGTGAGTTAGCCATTCATTCAGGAATTTCTTAAAACAGCACCTAG

AAACTGGGGACGTTAAAAAGTTCAACTCCAGAAAGAGCAGCGCCCAATCAAAATGTCACCAGTAGCTGTT

TTTATTTTTGTTGAAGTTTAATATCAAGAGGTTGACGAGCTTTGCTATGATTAATGCTATTTTGTTGCAT

ACGATGTCAATGGAGACGCTCATCAGGAGGATTACCTCTGTCTTCTCAATCAGTGAGGTGATCTATTGGT

CCAGTCAGCCTTTCTTTCATGTCAATATTCTCTTGGGTTAAATTCCACTTACTCTAAGATGAAGAAAGTG

TACTGTGCATTAAACAAATGTGCACCCTCTAGTTTTCTTTTTGGTCATAAATTGTGAATGTTTAGCTAAT

TACCAGGTTATCGATTAGAGAAAGCAGGGCGACTAATTGATGAACTTGTAGTCTGCTTTTCTACTTCAGT

TTAAGTCCAGTTGTTCTACATTTCACAGTAATAGGAAGAACTTTGTTTTATGTAAAGTATAATGTATATC

ACCAGTTTATTGGCAGCCTGACGTGATGTGAAGCACTGTTGTATGAGACTGAAGGGCTTTATTTTATGAT

AACAACAGCCTGGATGTACTTTATTCTGCTTATTACGTGGGTACTTGCCATAAAATGTAGAAATTAGACA

ATTGCAACTGCCTAGATGTACTTTAACCCACTTTTTACATTGGTGCATGCCATAAAACATAAAAATTAGA

AAATAACAACTGCCTGCATGTGCTTTATCTCTTTTTTTGCATGGGTACTTGCCACAAAACTTAATTAGAC

ACTAACAACCACCTGGATTACCTTTATCCCACTTTTTACAATGGTACTTGCCAAAAAAACGTAAAATTAG

ACCATAACCAGCGCCTGCATATACTTTATCCCGCTTATTACATGGGTACTTGCCACAAAATGTAAAAATT

AGACAATAACAATCACCTGTATGTGTTTTATCCCACTTTTTACATTGGCACTTGCCATAAAACGTAAAAT

TAGACAATTATAACCACCTGGATGTACTTATCCTACTTTTTATATGGGTACTTGCCACAAAATGTAAAAT

TAGACAATAACAACCGCCTGTATGTACTTAATCCCACTTTTTACATAGGTACTTGCCACAAAACATGTTC

TGAGCCTTGATAGTTTTTTTTAAACAATTTAATTAAATGCAAATCTTACAGATACGAAAACTGCCGAGTG

GAGCATACACTAACCTGTGTATTATTCAGTCACAAGATAGCGCCAAACAGTCAAAAACAGCGCCGTGACA

GACTTGTGTGTAAATAGATGTGAACGTTTTCGATGATGATCAAGATGATTTAAAGTACTCGAATAAACCT

GTGATAAAAGTTTCAGCGGTTGTTATCTGGGAATAACATACGTTAGAATGTTGTGACTGAGCAGTCAGAA

TCAAGTTGTTCTAGATAACAGTATATTAAATGTATACAACATTGTAGCTATGTTGTGAAAATTATATTTA

TGATTGATTGACAGTAATTTATCTTACAAGCATATACAGCTAAAGAATTAATTTTGACTTCAACTGTATC

TATAGCTCAGACTTTCACCCAATGAAGTCTGTTCACTTGGCAAAATGGCTCTAAAATCTATTCTCAAATG

TGATGTGTGCTTTCATTCACTGCAAATGTTGCACAGCTAAGATTTCCAGCAACGATTTGAGAGCTTTACA

CTGACAATCGGCGCTTTGACTGGGTGCAGCACCCGTGGGTAATTCTGTGGTTTAGAAAGCTGTCCTGGAA

GTGCTGAGTTAAGGTACCTCTGTAACAAGAGATCTCATCAATCACACCACAGAACCTGACTGACTGAATG

GTGATACGTATTGAGTTAAGCATGGCTTTTAATTACTATAGGCCTTGGGGTCTGCATAAAAAGACGAATT

ACTTTTTTAAATGTTAATTGTCTGATTAATTTTGGCACAGCTCTTAAGTCTAGATGGGACTGATTACAAA

CAACAGACCTGTCTGGGGAGCCAGATTTGATTTCTTATTTGGCATTGTATTGCATTTAAAAGGTAGGAGG

GTGAGTCAAGATATTTGATGTTATTCCACCTTCACCTAGGGTTTGAATCTATTGTTTCATATATGCTCCA

TCGTTTATATTGTGACGCATTGATTAACAGTAATACTTTTTCGTAATTTATTTGAGCGCAATATTGCACA

CCATTTCAGGCATGGAGACAGAATCATGAATAAAAACATTGTAAAAAGTCGCAATCTAGGAAGTACAGTG

TTTGTTTTGCATTTAATTTAGCTATATTTTATGTTGGGTCTGTAATCTAGTTTTTGAAAAGTGTTTATTG

CATTAGTTTTTATTTAGTATTTTGTCATTATTCAAACATTTGCCCTGAATGTTTAAATAAAGTGATAAAT

ATGATCACATATGGAGTAGATTTCCTGAACACAAAAGTTAATTAAATGTACATAAAAGGTATCTATTGTG

GTTATATATAATTATCAGAATATGAGATGACCTGTATCAATACATGAGATTTGTCATATTACCCAGTTCA

GTACCTTTATCTCAAAATATATACAGTGCTCAGCATATATAAGTACACACCGCACAAATCTGTATTTTAA

ATTCATATTTTAATAAGAAGGTATACAATATTATATGTTTGCGTATACATTAGTCAGTGCTAAAGCTAAA

TCTAGAGCTTATTTAACAAAATAACTTATGGTAACGGTCCAAAAACTAGTAAAGCCAAATTTATGTGGTA

TAGAGAAATATTAAACACAAAAAGGAAATTCAAGAGAAGCAAAAAAAAAAAATTTTTTTTGTGTGCAAAAT

TTAGCTTGAATTTAAATGTATTTTCTTTCAATTTCTATATTTGCTTGGTGATAAAATGTTATTTTAATAA

ATATATCTGTTTAATAAATCTGTTTTATTTAAATGTTCCAAAATACATTGCTTATATTTACTGAGAAATG

GATAAAAATATTCATTTTCAAAATGTGGTGCGTATTTATATGTGTAAATTAGACTCATAATGGTACGTTC
ACACCAGCGCAAGTGATTCACATGTAAAGTCAATGCAATGATACGTTGTACGAATAGACATCTTGCTGTG
TGATTTGTGCAAATAAGGCGTGTGAATGACGTGATTCGTACGAATGAAGCGTTTTACGTGTTTTGCTTAA
CGCATGAATCACTCAAGTTGGAAAACCTGAACTTTATCGGACATTCGCGCCATGGTAACCAACCAGGAGC
TTGCTCTTGTAGGGGCGTGAATATTGACGTAGTGCCTGTTGTTGGTGTCCTGAGGGGAAATTCTGTTGCC
GACACAGGACAACAGCTCATCAAACTGGGCTCTGAAGCACCTCTGAAAGGTCATCCGTGTACATTTACTG
GAAGGGTTGATGAACTCACAGAGCTAAGTGCACTGCTGAAAAGATCTAGTGGACTCAGACACTGTGCCGA
ACGAATATACGCTGTTTTTCAGCATTCCTAAAGCACATATAGCACAGCTAATCTCTCAAAAAACTCCATG
TTAGCCATTTAGCAATGAAGCTAGAGTCACCGGGCAGACCGAAGCCCTGCCCGTGACTCAAATCTGCATC
TATTGTGACGTTAATTTGACGGAGAATGAAGCAAGTAAAATCAAAATGCTCATGAATCTATTTACGCTTG
AATAACACCATTTATTTAGGCATGCCACGTCTGATGTGAACACAGCATTGGACTCTTGTGGACTGTAAGA
GACTCTTGTAGGCTCCTTTAGACTCATGTGGAATTCTTGGAAGATGATGACTGGTTGACTGCAGAACAAC
CAACACGTCTCAAAGATTTCTGCTTGTAACAAGTCTCCTGGTTTGGGTTCTGAATTGTCCAACACATAAT
ACACTCCAGCATGTTTGTTTCTCTCTCTCCATCTTTCCTCCGTCCTTTAAGCATTCCATCATGGTGCGGG
TAAAGCTGCTGAGCGGCAGACGGCATAAAAGTGATTTATGGAGGGCCGACCCGAGTGCTGTCGGGGGAAG
CCTGCGGCAGCCGGTCTCTCAGTTCCTCAGCATTGTTCCCGTCAGCCCCATTGTGGCTGGACCTCTGCTG
ATCTCCAACCCCCACTACACACTCACTGTGAGACCCAGCAGTGGAAACACCCCAAAATAAGAGCAGCGTT
GGCTCCTATTGACTCCTCACTTTGATTTTGGATTGGGGAAACCGGAGCACCTGGATGAAACCCACCTCAA
CATGGGGAGAACATGCAAAGTCCACACAGTAATGCTGACTCAAGCCAGCGACCTTCTTGCTGTGAGGCAG
AAGTGCTAACGATTGAGCCACCATGCTGCCTGTAAAGCTATTCAGAAGACATTTATTATAACACAGTCAA
GTTGTATATTATATGGTCTAATATTTGCTGTGTCCACCAGAATAGGCACAAGCTTATCAACAAGGGGCAA
AGCTTTTTACTTTCATTACAAAATATTCTAAATATTTTTATGTTCTACATATCAAGTCACCTCTCACAAA
TCTGCTGGTCTCACTGCAAACCAAAAACGGAAAACAATGTAGACCATGCACATTTCAACCTTTCCCCCAC
ATGCATATATCCACCTCCACTGGGGTAAAGGACTGTAATCAATCAAGCCAGACTATCCAAGGACAAACTG
ACACGAGCCAAATTGCGTTTTGCCCAATCAGAGTGTCTGTACATGTTCTGGGGGTTGGCCAAGTGGCGAT
TCTGAGCCAATAGTTGTCGTAGCCGCTGTCGTAACGTGAAGAGGCATGACTGGAGGATATCAATAGTAGC
AGTGAGGTAGGGAGGTGTGTGTAAAAGTCAGAGAAACCATACAATAATTTGTATAAAATAATTACTAACT
TGGTTAAGCATATTATGCAAACACGTTCCCCTGTACAGTTGTATTTAGTATAGACCAGAGATGTCAAACT
TAGTTCCTGGAGAGCTGTGCTCTGCACATTTTAGTTCCAACCCTAATTAAACACACCTGGTCAAACTAGT
TGAACCATCTGGCTAGTTTTAAACCTACAGGTAAGTGTGTTGGAGCGGGGTGGGAACTAAACTGTGCCGG
GCTGTGGCCCTCCAGGAATTGAGTGTGACATCCCTGGTATAGACCATTTCAATGTGGTCATGTCATTGGC
TCATGAATATTTCATAACTGTACAAGAAGCAATCCAATCATAACTTAATGTTAAACACAGCTATCATAAC
ATTATTTATCAATATGTGGCTCTTTTTGGATTCTCGGAAGCTTTAGAAATTAAAAATGTAAAACAGGAAG
TATGCAGGGACCATTATTTTTGTTTGCATCCATCAAAATGGTCTATAGTTTGTAGAATTTCTCTTTATGA
TGCTCTTTTTGTTGATAATGTAGACTCATTGCATCGATCAATATTCAGAGTTCATGTTTAGTGGCTTCCA
GTGCCTTTTATTAATGAATCAACGTTTATTAGAGCACAAAATTAAATGACTGTCAGCGCGGGTTTAGAGT
TTGCATACTGACCCATGGTTATAACTGACCCATGCTAAAGAGTTGAATAATTCATATATGGTTGCCATAG
AAACGGATATGATCACCTGGAGGGTCACTGTATCCACTTAACTCGAACTGACAGGTAAGAAAATGGAGAA

GTTTAAATCTGAGGGAACAGTCTAGCATCTGCATCTTGAGTTTCAAAAGAAATGGTTTCTATTCTCTCAG

TTACTTCTCGGTCACTTGAGCGCCAAAATTTAACCCACAAGCAAGCAAATTGAGTTAGTAACAGATGACT

TTCGAAAGTTTTGTTGCGAAGAGGAAAAGGATCAGTTAAGGACCCACGAACTGCCAAGGAATGGATCCGC

AGCCCACTTGTCAACAAATCAGGTGAATCAGGCATGTCTGCGCAAAAAGATTTACTGCTGGAGATCGCAA

ATGATATCGGTTTTAGGGGTCGTTCACATATTGTGAATTTTCTGCGCACAAGTGGAGGCCTGCTGCGTGT

GCGCACATTTTGGGAGTGACATTCTTGTGCTTTCCAGGCACTCCCAGTTGAAAACATCTTCAGAATGCTG

CAAGCACACCACAAGTTATGTGACGAGAACAGACTGATCAGCTTCAGTCTTTCTTTGTTGCTTTAAAACA

TATTAATTATTGATGCAGTATCACATTTATTGTTTTTATAATAAACTTATAATAGTTTAAACATAAACAA

TCCCGTTTAAAGCACAATGGAGTTCAAAGCTGAACACACTAGTTGGGCAGCTTACCGATGTCTTGTGCAT

TTTCCTCTGTCTGACAGTATTTACAATTTAAACGTCTTATTCATTTACAACATTTAAATGAAACATTTAA

TTTTAGTTGGCTTTTTTTGTAGCTCTGCAATTAAACAAGGAAACAAAAGGTTACATTAAATTCAAAATGA

CGATCTCTTTCAAGGTCGTTCACCCTAAGCAGCTCCCCATCATCCTCCCTCTCTTTTCAAATGGGTTGGA

GTGCCAAACCAAAGGTTACAATGAGCCAACTTTGGCCCACGGGCCCTACTTTGGGCACCGCTGCTATATA

TGAAGAGATGGTATGGATCTTAAAGGGTCACGAAACACCAAAACACATTTTTTGAGCTATTGACAGTCGT

ATATGTGTCCCACACTGCTAAAAACACTATTAGGACACCTATATTTCACTAAAAAGTGTAAATTGGTTGT

TTTTGCGTAATTTCAAGCAAATTCGTACTTCCTGTTTGAAACGAATTTTTGAAGCTGCGTCACGGTCATG

ACATAATAGCGTGTATTCCAGCGTGCAGACTGGACGTCTGTGCCAGAGTGTGTCTTATTACGTCTTACAG

TGTGATGCATTAATGCATGAGTAAAGCTTGGTTCAAACCAATCAGCGCGCTCTATTGTGCAACTTCATTA

ATATTCATTAGTGTCACCGTCTTTACACCACAGAGACGCCACGTTGTGTTGGCAAAACAAGCGTGAAGTG

TTGATTTTATAGTTTGCTGCCGTTAAGTTTCGTTTTCATTTTCTCTCTGTGAGAGCTCAGTCTCATGTGT

GGATTAACAGTGTATGCGACGCTCCACAACAATAACTTACGTGTCTAAGGGGGATTATTGTTTACCTGAG

AGCTGTTCTCATCTGCAAACGCTGAGATACGGATTCCGGATGTAGTCTTCTCTTCATAAAGACGCGGCTC

TAGTTGCTGGTGATTGTCCTGTCTCTACAGATTTGGTAAGTGAGCGATCAGTACTCTTTGTATATTCAGT

TTGTTCGTATCGAATTAAGTTAACTATTGCACTGAGTGCAAACTGGTTAGCACCGCATTTTGTGACCGGA

ATAACACACGCGGCTTTCTGACGCTACCTGCCGTGTGCATCTAAGTTTCCGGGAATGCAGAGGTTTTTTT

TCTCTCATTCGCCGTGCGGTGTCAAACATTGCATGAAAAATACACGCTTAGAGCAGATCCTCGAATCAAA

TATCTCGTTTGTCGCGAGGGGCATGAATGAATTCCCTGAATGAAAGAGCCAAACTGCAGTTAAAGTCCAC

CATTTAATAATTTGGCAAATAATTCGACTACAGATGTCCATGTAGGTTAAACACCATCACTTTCTCATGT

GTGTGTATTTTGACTGAAACTCGCGCGTGCCCAAATAGACACTCCCACACCCTCCCACTTTAGTTCCTCC

GACACTCCCCCCTAAACAGAGCTGGACACGCCCACTTTTCTGACTTTTTCCAAAGTGGAGGTGTGAAAAC

ACCCTGCTGAAACGAGGGGGTTTCATGGCCCTTTAACATCACGATTTTTAGTGCCTTGATTCCAGCTCCA

CTTTTTTTTTCTAGGCCTCCCCTACGTAGTCGCTTCACTTTTTGCTGCACTGAACCATTGAACTGTGCTG

CCACCAACCTACTTGTGCTGCAGTAAGGTAGCATTAGCCGTTTCCTTCCTAGTAACAGTGAGCTCCAAGC

CTATGACAAATTACCATGTGGCTACCTGAGACAACAGGCCTCGTCTGCATGCTCCTGAGGGATCTTCGTA

TCTCACAGGGTGACCCAGTTCCATCATGCTTCTCCTTCACCTCACTTCTCCTCCTGCCTTTTCTTTCACT

TCTCCATTTGCCCCAAGTGCTCTCATTGCTACATTCTCTTTATATAGGCACTGTTTACCTATGTCAAGTC

GATATCAAGGTTTCCAAGCCATTCATCATCTCGATTTGCTTTTGTTCACAGTAGAGCGAATCATTACCAA

GGAGGAATTACAAGAGGTTTATGCCACAGACTGCATATTTTTAGTGTTCTTTGAACTCCTCCTCAACACA

CAAAAAAAGTTGACTGCAATGCAGTGGTGCCAGCGAGCTGCATTAATATTAATGCCTGCCCATTTCCACC
ACCGTGGTAGGCAGCCTGTCTATCTACACCGCATCTGGAAGGATTTGCAAGTCACAACACCAGTGCCCTA
CTGGCACCCGTCCATCTTATTGACGCTTACACAAAGGGCGCAGTAGTGCCAGTCATTTTCTGCACTGCCT
ACTACCTAACATTTCCACCACTGCATGTTAAACCCAATTCTAGACAATGCACCAATTGCTCCACAACTTA
CTACACTTAAGAGATTCACGTGGCTTAATGGAAGTGTGGAAACTTTATCTCATCTTGCCCATTTTGGTAT
TGACCTTATTTACATGCCCACATACTGGCGTGAGTATTTCAGGTATACAGATGTAACTGGGCGTATGTGC
AATGCGCGCTGTGTGTACTGCAGTATTGCTGCTTTGCTGCACAGCGCTCCAAATGTGTACTGAGCTGATG
CTCTTCCTCTGCACTACCTAGGAAACTGGAGACTGGCTTTGGGTTTACAAGAATCCACCCTGCAGCAAAA
AAAAGAAAAATAAGTGGCACTCCAGAGGTTTCCACAAATCCTCCTCATCTCTGAAATTACGTTCATCAAT
TTCTTTCTTCTCTTCTTTTCGATGCTTCTTCATCTGTTTCCGCTTATTCCATCAATGGTAGCTTAAGAAG
TCCCTTAACACTCAAGTTCTTTTAAAAATGCAAACCCTGCATTTTTTTAGCACAGTGATGTGATATTACC
TTGATTCATCACAAACTGATTGTGAGATTGCTTGTGTGTGTCAGTTTTCTGGGGGTTTGGATGTGTTGCA
GTCAGCAAGTGGGGCTTTTTTCAATGCAGTCAAAGAAACATTTTACATTTAAGAGCTGGTCTTAAGCTCC
GAGTTTTTTTTAAAAGGGACAATATGTCATTTTCACTGCTTGATGGAATGTATTCATAACAAACAAAGGT
GTAATTTGATGGCGCTGTGAGTTTAGAAGCTTGCTGCATTTATTCGCAATGCAACCAAATGTAGAAATGG
GCTGCAGATATTCGTAAACAACAACCAAATATAGAAACCTGCTCCAAATATTCGCAAANNNNNNNNNNNN
NNNNNNNNNNNNNNNNNNNNNNNNNNNNNNNNNNNNNNNNNNNNNNNNNNNNNNNNNNNNNNNNNNNNNN
NNNNNNNNNNNNNNNNNNAAGCTTGCTGCATTTATTCGCAATGCAACCAAATGTAGAAATGGGCTGCAGA
TATTCGTAAACAACAACCAAATATAGAAACCTGCTCCAAATATTCGCAAAGCAACCAAATATGGAAACTC
GCTGCAAATATTTCCAACACAACCAAATATAGAAATGCGCTGCAAATATTCGCAACACAACCAAATATAG
AAATGCGCTCCAAATGTTCAGTGCGCTGCAAATATTTGCACTGCAACCAAATACAGAAATGCACTGCAAA
TATTCGCAACACAACCAAATATAGAAATGCGCTCCAAATGTTCAGTGCGCTGCAAATATTTGCACTGCAA
CCAAATACAGAAATGCACTGCAAATATTTGCAACACAATCAAATATAGAAAACGTGCTCCAAATATTCAT
AACTCAACCAAATATAGAAAATGTGCTGCAAATATTTGTAACGCAACCTAATATTGAAACTCGCTACAAA
TATTTGCAAAGCAACCAAATATAGAAATGCGCTGCAAAAATTCTCAACACAAATATATAAACTTGCTTTA
GATATTTGTAACACAACCAAACATAGAAATGCGCTGCAAATATTCGCAACACAACCAAGTATTAAAACTG
CAAATATTCGCAATGCAACCAAATATAGAAATGCGCAGCCAATATTCGCAACACAACCAAATGTAGAAAC
TTGCTGCAAATATCCACAACGCAATTAAATATAGAAATGCGCTGCAAGTATTTGCAACACAACAAAATAT
AGAAACTTGCTCAAAATATTCATAACGCAACCAAATATAGAAATACGCTGCAAAATATTCATAACGCAAC
TAAATAAAGAAATGCGCTTCCAATGTTCGTAACGCCACCAAATTTAGAAATGCGCTGCAACTATTAGCAA
CGCAACCACATATAAAAATGTGCTGTGAATATTCATAATGCAACCAAATATAGAAATTCGCTAGAAATAT
TTGCAACAAAACCAAATATAGAAATGCTCTGCCAATATTCGTAAATATTTAATAATAATTGAATCCACTG
CTTCCGGTTCTTCTAGACGTGGAGAAGTAGTAGTGCTGTTGTATCAGCACACACAGTCTTACTAGTTAAA
ATTGCTTATTTTTTCTGTATTTTAACATTCTTCGAAACATTTGAGCTAACGTGAGTACATTTGCCTTTGA
GGTTTGCATCCTAAAGTATCACCAATTAGAAATCTCAAGATATTCTTCCTCTTTAATAGTCATTGTTAAA
GTGCTCTATTAGCCTTTATTGCTCTATTTTCTCTTGTTTACCGTTCTATTTCTCTTATTGCAGCCATCCT
TTTTACTCTTATCTTGTACATTGTCACCGTTCTGCCTGTTTTTCAGCTCGTGGTCATAGATTGTCTCCAC
TAATTCTTTTCTGGTTTACTTGTTACCTCATGTTCTTATTATAGGAGCTGCATCAATTTCTGATCAGCTT

CCCGTTTGCGTTGTGAACATATGTCGTGGTCCTACCTGCTGATTCCAGGTCAGGCTCCAAGTTTTGCTCT

GCAGATCTGAGCAGGTGCTAGAAAACGATTTGTCGATCCCTGTAGATTTGTGGTTTTATATCAATTTATC

ATACTCGTTTGATTCTCGGTGCCATTCACAGAAATATGCAGATTTCTATGCTTGCAGTTTCCATGTACTC

TTACTTATAACATGTTCATTGGGTCATTTTTTTCTGTCCATTGCTAAAACTTTGTATTCTGATCTTTGTT

Mib **exon 13** CTGCAGTGCCATGCGTGTGCTGCTTTCCAAACTGCCACGGCCATGGATAGTGGACGAGAAGAAGGACGAT

GGCTACACCGCTCTTCATCTGGCTGCGCTCAACAACCATGTGGAGGTGGCCGAGCTACTAGTTCACCAGG

TCAGCAGCTCTAACACTCACACATTTTACTGTATCAACACCCCATATTAAATAGGAAAGTTGAAAACTTC

TTATTCATACGTCTTAAACTCAAAGTCAAGGTGTTCAAAAGAGTCATGAACACCGTCATTAGTTGCATCA

GCTGTGTTTGATCAGGATTGGAGCTAAACTGTGCAGAGCCATCCAGGAATTGAGTTTGAGACCTATGATC

TAATTGATCTAACATCATTAGAGCTGACAGAGTTTGCTTGTAATGTAATTCTCATTGGACCAAATCAGTC

AGGACAAATTTCAACTTTAGAATAGTGGTCCATTAATGTATTTACAGTGCATCCGGAAAGTATTCATAGC

GCTTTACTTTTTACACATTTGTTTATGTTGCAGCCTTATTCCAAAATGGATTATATTCATTTATTTCCTC

AACATTCTACACACAATACCCCATTATGACAATGTGGAAAAAGAGTTTTTGAAATTGTTGCCCATTTTTA

CCAACAAAAAACCTGAAGTCACATGACATCAGTATTCACAGCCTTTCCTCAATACTTTGTTGATGCGCTT

TTGGCAGCGATTACTGCCTCTTGTCTTTTTGAATATGATGCCACAAGCTTGGCACACCTGTCTCTGGGAA

TTTTTGCCCATTCCTCTTTGCAGTACCTCTCAAGCTCTATCAGGTTGAATGGGAAGTGATGGTGTACAGC

CATTTTCAGATCTCTCCAGAGATGTTCAGTAGGATTTAGGTCTGGGCTCTGGCTGGGCCACTCAAGGATA

TTCATTGAGTTGTTGTGAAGCCACTCTATTGATATTTTGGTGGTGTGCTTTGGGTCATTGTCATGCTGGA

AGACGAACCGTCGTCTCAGTCTTCATTCAGGATGTCTCTGTACATTGCTGCATTCATCTTTCCCTCTATC

CTGACTAGTCTTCCAGTTCTTGCTGCTGAAAAACATCCCCACAGCATGATGCTGCCACCATGGTATTAGC

CTGGTGATGAGCGGTGTCTGCTTTTCTCCAAACGTAATTTATTTTGGAATAAGGCTCTAACATAAAAAGT

GAAGCGTTATGAATACTTTCTGGATGCATGTACTAACATTAAGTATAAATAATATTCTAAAGTAAGATTA

CTCTTTCGGTTCTTCAGCACATACACCTTTGAAAGTCTCTGCACGGGAGTTACAGCAGCCCTCATAACGT

TTGGCAGTCACTGAAAATGTCCCCCCACCAATTTGAATTTGAGACTCCTGTTCTAGATGGTTGCTAAGGT

GTTGCTAAGCGGTTTGCTAAAGTCTTCTGAGTAGTTGCTTGGCAGTTGCTAACATAAATTAACATGGTTC

TAGTATGAATTATCATGTTGCTAGCATGTTTCTAGTATAAACTGGCATGTTGTAAGCATGCATTTAGTAT

GAATTAGCATGCTGCTTGTTTGATTATGTTTGTTAGTATGTTTCTAGTATGGATTGGTCTGGTGTTAGTA

TGTCCAATGTAAAGTGAATGGCAGCTTCTTTTTTTTTTACAATGGTAGTCTATGGGACAGTTGCTAGGGT

GCTGTAAGTGCTTGCTAGGGTGTGGCTAGTGGCTAGTAACTTTAAATGTCATCAGTGATTGGTAGATTGG

TAGTCTTAGTTAAATGAGCCTAACCGTAAGTATGTATGACAGTCTGGCAGAAGGTTATGAGGTCACAAAT

TTGGTCCAATGTTAAGTCAATGGGACTTTTCCAAGTTTTTTGAAAACAGTAAGTCGGATCAGTCGGAAAA

GATATAGCAACTTAATTCAGTATAGTTGTGAGGTTTAAAGTTAGTTTGGTGGTTGTAGTATAAATGGTCT

AAGAGGAGATGCGTTCTTAAAATAGTCTAAGAAGAAGACGGAAGAAGAGTAAGTTCATGTAGTATAACAG

TATGTTGGCTTTTTCAAGCCACCTGAATTTTTTCAAAGTGTTACCCCTAATTGTGCTTTTTTGGTGACTT

GCTTTAAATTCAAATGAGATTTTTTTTAAAGAGGGCCGAGTTATGAATGCCTGTTTGTCAGCATAGTGGC

AGATTCAGAAACAAGACTAGCATAGTAAGGGTCAAGAATCTTGGTGTGAGTCAGATCTGAGTTTCAATAG

TCATGCCAAAGCAGTTAGTAAATCAGTTTACTATCATCTCAAAAACGTTGCAAGAATCAGATGCCGAGTT

TCCAGTGAAGACTTAGAGAAACTTGTTCATGCTTTTATCAGCAGTAGGGTGGATTACTGTAACGGCTCCC

TCACGGACCTTCCTGAAAAGACAGTCAAACACTTGCAGCTCACGATGCGGCCTGGATTCTGACCAGAATC
AGGAAATCAGAGCACATCACACCTGTCCTCATGTCTTTACACTGGCTCCCAGATACATTCAGAATAGATT
TTAAAGTATTATTACTTGTCTATAAATCACTAAATGGCCTACGACCTAAATTTTTCAGATTTGCTCACTG
AATACAAACCTAACAGATCACTCAGATCTTTAAGATCATATAAACTAAAAGTTCCAAGAGTTCAGTAAAA
CCAGGGTGACTGCTGTCAGCTACTAACAGCGCCCCCGCTGCTGGAATCAGCTTACAGACATGATCAGAT
GTGCTCCAACTGTTTAGCTGTGCCTTTACTGTATGAGCACTGTGCTACGTCCGACAGATCACACTACTAT
GTTTTTCTCTTCTTTTTCATTCTTTTATAACACATTTTAACACATTTTTATCTGTTTTATTGAATCAATT
TTTATTATTTGTTTTCATTTTCTTATACTTGTCTCTTTCATTTCTGTTTATGTGAAGCACATTGAATTGC
CACTATATAAAAAAACTTGCCTTGCCTATAAAAAAAAATTGTGATTAATGAACGGGGCTTTCCACCGTTG
ATGACACGTACAAAGGGAGAATGTCAATCACAGCGTTTCTGCAGACTGTTTTTAATCAAGTCTGATTATA
AAGAAATAAAAGTAATACATTTTCACCAACATGAGCTGGTTATTCACACACTGCTGCCACCCAACTGTGT
GTAAACCCTTCACAAAAGTGATTTTTGGATAATAATTTTAATAATTCTATTCTAGCGAATGCGTTCAAAA
TGTGTTTAATGCTACACACTGCAAGGACACAGTGTTATCAAGTCGAGCAGATTCCAGTTCAGTGATTCTC
ATTCTGCGCGTGTGGATTTGGCTCCTCACCTTCACACGCATGACACTATGGCTCAGCGTTATATATCACA
GCATAGCAATAATGTTATTGCAACATTCCAGCAGCGTTACGAGAACGCTGCGCCAGCCTTTTGTCAACTA
CCTGCCATGATGGAAGAGAGGCAGAGACAAAGAAAAAAAAAAAAAAGGTTCACGCCAGAGACTTAAAGCCC
ACGAACCACAGTAGAGCTGCACTGCAGTAAATGCAGAGAGATGTTGCTATGGGCTTTCAGTCAGGGAATA
TCTGAATACAAATCACCATTAGCTCGCACAACCTTCGGGACTAGAAGGTGATTCATCTTCAGTACCTGAG
GACACTGAATTTCAGTGAATGTAGCATGTAACACACACTAAAACAAGTGCATTTATATACATAAGAATGA

Mib **exon 14** GTTTAAAGGACAACCCTATTGATTTGCTCTTCCTCCATAGGGAAATGCCAATTTGGACGTCCAGAATGTT
AACCAGCAGACGGCATTACACCTTGCAGTGGAGCGCCAGCACACCCAGATAGTGCGAGTAAGTAGCTTCG
GTTCTGTTTGTTTTCTTTCCTTTTTTTTTCAATGTTTGACGTCTGCACCCCTCACCCCAGCGCTCAGTCT
TCCCACTCAACGCATCAATGAAGCAATGTAGCTAAGTGCTCCGTCTTGAGATTAAAATGCAGGGAGTCCT
GCTGCAGATTTATCCCCTCCGATTCAATTCATTTAGTCCTGAGCAATCTGGACCTTGCCAGGAAAAGGTA
CAGGGAGCAGCAATGGATGCTGATTGGATGCGCCATCTTGTGGTTATGTGACAGCAGTGCAGTTTTAGTC
TTTTCTAAGCACATGTGGTATATTTGTATGGCCTCCCAGATAAAAACGAAAAAATATGTTACAAGTGCAC
TTGAAATGCAGAGAGTATGTTTAAAGCACAAATAACATGCATACTAATAGTACATCCTAAGTGTATTTCA
ATGTGTTTATTATAAAGTAAACTACACAAGTGGTAAACATTTTGCTTAATTGCACTTTTTAAAAATCATG
CTATTTACACTTTTTAATGCATTAACAGAAAACAAACTAATATGATTCTTTCTTAAAGTATGTTTTATGT
GCACTTTGTATAAGAACGAAATGCAAATTTGAGTGTTTAAAGTGTACGCTACCTGAAAAAGGTCTTGTTG
CCTATCTAAGCTTTTAGGAACAACAAATAGTAGTAATTATAAATTAAAAGAATAGTAATAGTAACTTCTA
ATTGATCATTTGGTATCAGAGGTGGCTTATATGAAAGGCAAAGGCCTCTAGACTCTACGCTTCTTTTACC
ACAATAATATATGTTCATGCTTTGACTTTTAATGATTTAATTAGGACAGTAAGGTCTGTCTTTGGTTAGA
CAAAAGTCTTGTCACTTAACAGAAATGATGTGCAGTATAGAATATAAAGTCCTGCTGCAGTGGAAACAGA
ATGAATATTTGACTCCCATGAGCTTGGAGGACTGCATCCATACATTTCTGCAATGACTCAAATCACTGAT
TAATAAAGTCATCTGGAATGGCAAAGAATGCCACCAAGAATGACTCCTAGAGCTCAAGATTCTTTGGATT
CATCTTTTAATTCCTCCTCCTTCATCTCACCCCAGACATCCTCAATAATGTTCATGTCTGGTGACTGGGC
TGGCCAATCCTGGAGCACCTTGACCTTCTTTGTTTTCAGGAACTTTGATGTGGAGGCTGAAGTATGAGAA

GGAGCGCTATCCTGCTGGAGAATTTACCCTCTCCTGTGGTTTGTAATGTAATGGGCAGCACAAATGTCTT
GATACCTCAGGCTGTTGATGTTGCCATCCACTCTGCAGATCTCTCGCACGCCCCCATACTAAATGTCACC
CTAAACCATCATTTTTCCTTCACCAAACTTGACTGATTTCTATGAGAATCATGTTTTTCCTCCTGTATTT
CAAAATATATGTTCATTTTTTTTTAATAATACATATTTTGTAAAGTATAATAATTCACAAGTATTAAAGG
AATGATGAAATCTGGAATGCTTCAGATGTTATTAAAGTCATAGTTCACGCAAAATATGAAAAATGTACTT
ACTATTTGTAATAGTAACAGACTGGAATTCCACCAAGGGATTTTAAACAGAACGTCAGTATTTCCTATTA
TATTTTTCATCTGCATAAAGTCATTTTTTCCTTGTTTGACATAATAATATATCAGTAAGATAATTTAAAA
ACTGCTAGGGGGAATATTATGAGCCCCTTAAAGGGATAGATTACCCAAAAATAAAAATCGTCACCTTAGA
ATTATAAGGTTCTATCTGACATTTTAGTCAAAATTGAGTTATTGACGTATTCTTATTAAATTAAATTTAA
AAATTATTTAAAAATTTATTTACATTATGGGATGTTTTATTATAAGTTGTTTACATTTTAAGACTTTTTA
TTTAAAAAAAACAAACGTTACACACACACTTTGCTATGGAACGCAAAAACTCAATATTTCAAAATCATTT
AGAACGCAGATAGAACCTTATACTTTTTCAATTCCAAGGTGATGAAATGTTCTTACTATTTACTCTCAAG
TGGTTACAAGCCTTTGAGTCTCTCTTCTGTTGAACGCTTAAGGAGATATTTTGAACAAGCTATTGACTCC
AAAATAGGAAAAACACATGCTATTGAAGTCATTGGTAACAACTTTTTTGCATTGAACAGAAGTAAAAGGT
GAGTAAATGATGACAGAATATTTCATTTCGGTTGAACTATCCCTTTGAAAGCTTGTTCTATTTGAAAATA
Mib **exon 15** TGAGCGTTACAATCAGCACTGCTAACAACACAAGGTTTAAGTTGAGTCCTGTGTGCTTGTTTCCAGCTTT
TGGTAAGAGCCGAGGCAAAGTTAGACGTTCAAGACAAGGACGGAGACACGCCACTGCACGAGGCCCTGCG A←→G
TCACCACACGCTGTCCCAGCTGCGCCAACTTCAGGACATGCAGGACGTCAGCAAAGTGGAACCCTGGGAG
CCCTCCAAAAACACAGTACGTACAGAGAAGTGTCTAAAGGTTAGAGGTGTTCAGTAAATGAATAGAATGT
GAGTTTGATGCACACTTTCACTGCTGTGCACTTGATTAGAAATGGTGTTTTCATGAAATTTTTCGCAATT
CTAAATAGATCCACGATAAATGTTAATATGCTGCATGTGTTGTAAATATAAATATTTGTTTATTAAAACT
GCATAGACTCAAAAAGGATTACAGATGATATAGGAGCCTATGAAATGTATTATAATTTTTAAGGATGCT
TAGAATTTTCAGCTGCGAAAATTATCAGGCAAATATTTATTTGCTGGCTGAAATAAAAAAACGGGAGAAA
ATATGAGCTGAAAGGAGCAGACGCAGAATGCTATGCGCTATAAAACCCTTTCATTTCAATGGCCATGTAA
AGACGGGTTGTTGAAGGACCAATCAAAGCACACGAGCAGCACACCGTCGACTAGCTAGCATGTGCACTGT
GATTCAAACTACACTTTGGGCCCATTAGTACCAATTGAGCATCGTGTCAACGCCACAGCCTACCTGAGTA
TTGTTGCTGACCATGTCCATCCCTTTATGACCACAGTGTCTCCATCTTCTGATGGCTACTTCCAGCAGGA
TAACGCGCCATGTCATAAACTGTGAATCATCTCAGACTGGTTTCTTGAACATGGCAGTGAGTTCACTGTA
CTCAAATGGCCTCCACAGTCATCAGAGCTCAATCCAATATAGCACCTTTGGGATGTGGTGGAACGGGAGA
TTCATATCATGGATGTGCAGCTGACAAGTCTGTAGCAACTGTGTGATGCTATCATGTCAATATGGAGCAA
AATCCCTGAGGAATATTTCCAGTACCTTGTTGAGTCTATACCACGAAGGAATAAGACAGTTCTGAAAGCA
AAAGGGGGTCCGACCCGGTACTAGTAAGTTGTACCTAATAAAGTGGCCAGTGAGTGTATGTATATGTGGG
GTTTGGGAAAGCACACAGGACACATATAAAGACCTTAATATGAGCACATTTAAGCATATCAAAAGAACAT
CACACCAGCTCTGTGAACCCTGTATTTACATTAAATACTATTTAATATAAAATATAACAGTTAAAAAATG
ATCAGGTGTATTAACAATACACTAAACCACCATTCAAGCAGACTTGGTATGGTTTCCCTAATTTGGAAAG
CACCCTTTTATAAAATAACTGCACTCTTAAGTCAAATACTCAAGTCCCCGCCAAACAAAAGCCATAAGAT
GAGCAGTAAAACGCGGAGGCAGGGCAGATGCTCCACAACTGAACAGGCTTTTTCCAGTGTTAACAATGAT
AGATCGTCTTCTGCTATAAGAGCCATTTCCCTGCTGTCACATGAGTAACCGGCCCAATGAGCCACACTTC

CACTGCAGTCCTCCGTCTGTACACTACACCATCCTTCAGGTGAGACGTTCCCCCCTGATTATCACTTCCT

CCTGTATGGGATTGCATATTTGAGGAAAATTGAGTGGGTGGGAGAGCAAGCCGAAAGAAGACAGCTTTGG

CAGACATGAGCCCTAATCAAGACATTTACTGTACGCTCGTTCCCAGTAATTTATTCCCACAATGATGGCA

ATAGAATTACAGAGCCTATGAGCTCTATTAGAGCAGATTTAAAACGTAATGAATACCATGTTCGCAGGTA

AATGGAGCGAATTGTGACAGTGTAGAGAGTGAGCTCCTCATAGAGTGCACCGGTGATGTGATCGGTCTCA

CTTTATCAGTCATGCAGTTTTTAAAAAAAAAATACATTTCCCAATTGTTTAACAGAGCAAGAATTTTTTT

TACAGTATTTTCTATAATGTTTTTTTTTTCTCTGGAGGGTTTTATTTGTTTTACTTTGGCTAAAATAAAA

GCAGTATTTAATTTTTTAAAAACCATTTTAAGGTCAATATTATTAGCCCTTTTAAGCTATGTTTATGTTC

AATTGTCAAACCACTGTTATACAATGATATGCCCAATTACCCTAAATAACCTGGTTAAGTCTTTAAATTT

TAAGGCCGTGTTTACACTAATACGTTTTAGATTTAAACCGCATAAGTTTTACTACGGTTACGCCGTCCGT

CCTCAGTACCCAGGATTTTTAGAGTACGGGAAACGGATCCAATTCATAAACACACACAACACAATTCGTA

AAATGTGCAAATTCTATTCGTAAATTTAAAACACAATTCATAAATATGCAAATCGACTTCGTAAGTTTAA

AACACAATTCATAAATACGCAAATGCAATTCGTAAATTCAAAACACAATTCATAAACATGCAAATCCAAT

TCGTAAGTTTAAAACCCAATTCATTAATACGCAAATCCAATTCGTAAATTTAAAACACAATTTATAAATA

CACAACACAAAATTCGTGAATACAAAACTATTCATGAATATGCAAATTAAATTTGTAAATTCAAAACACA

ATTCATAAATACGCAAATCCAATTTGTAAATGTAAAACACAATTGATGAATACACAATGCACAATTCGTA

AATTCAAAACACAATTCATAAATATGCAAATCCAATTTATTTGGGGAAATGTTTAATTCATATATTAGAT

TTTGTAAATGTGAATTGTGTTTTGCAGGTATGAGATTTTTTTTTGCAGATGTGATATGATTGAGACTAGA

TTTTAGAATATTGAAACATTCATGCTTCTCATTCAAAGCCTTCTCTTATGTTTAAAGGACAGATCAAAGA

TACTTTAGTTTTTAGGTTTATTATTATTGGGATAATGTCTTTGTATGATTAAAAAAATGCTAGTTAGAGA

CGGGACATATTTGCAAACTCTGTAATTTGGCTACAGCTCATACATCACATCACTACTGATCTGCAGGTTT

TTCTCTTCACTGCTGTTGTTACACTTTAATAGCCTTAAGCATCCCGCAGCACTTATCGGCGACCTCCCAT

CCCGCAGCCTTTCTTTATCTTCATTAGCACTTTTCTCTATCAATGCACTGTGGATCCGCTGTCAGAAAGT

CACACTATAATGTCTGACACCAGACAGACTGACGATGCTCAACCCTATCTGTTACAGCTTTTGTAGCTCC

TTTTACGCCTGACCCCAGCCCTGCCAGTTCACATCATCGCCTCTCCATGACTGTCGTAAATCAAGCGTTG

TGTGACAGGAGGCTGCTTCCAGGCCAGCCGTCTGCCATTTATTAACACAAACAGTGTGACAAATCTCTCC

ACCCTATCAAACCATCACACTTACCACATGCAGCAGATGGAGAGGCGAGCCATCCATTCAAAACACTCCA

CCGAAGCCATCAGTATTCAGAAACCTGCCCACTTCTTCAGCTCAATGTGCTCCATAGCATTTCATCACTT

CTGTGGCCTGTTGCACAAAACCAGTTGAGGGATGTAGAGTAAGGCTGGGACACACCAAGCTGATGCCAAA

GAACTAGCACCAACTGTGTTTTTACCTTGTGTCGGATGTGTCTAGATTTGTCTGGACCAAAAAGATGAAC

GTGAACACACTAAGGCCCAATCCCAATTCTATTTTTGTACCCGTACCTCTTCCCTTTGGCCCTTGAAACA

GAGTGTGAAGGGCTTCAATATTTACGCCTGAAAATTGGAACAGCATTGCAGCTGCACACATAGTCATATG

TCATCGCAGGCTCTAGCTTCATATGAGATCAGATGACCGCGTCTGCTGTAGTTATTCCAGTTATGTTATT

TTTTGGTAATTATCTTCAGCAAATCACTGAAGGCAGATATCATGACGATATAATGTGGCAATAAGAGCGT

AACTATACTGTGCATTTAACACAGTGGCCATATTCATCAGTGTAAACACAAAAAATAACATTACTATTAT

AGCAGACACTGTAAAAACCCCATTCTCAGACTTTTCTGACAGGGTGTTTGAGCGTCATCGAGTGTCAGAA

TGTTGTGAAACGACTGTACAGGAGTTATTGTTATGGATTTATTAGGGATTATAGATAGCGAAATTTTGCA

GGTTTTATTTTCTTTTATTTATTTATTTATTTTTATTTATTTATTAAATTTTTTTAAAGCATGATGGTTT

AAACATGTGCTCGTCTGTTGTAAAAATTTGCAATATTGACAAATAAATAGTAATTTGTGGCTCTCCTTAT
GGGTGCAGCGATCCTGCTGTTATGGCTAGTGTATTCTGGGAAATTTTCTTACCCCTGGGTTTCGAATGTG
GTCCTGAAAAATTTCAGTTCGAAGGGGTCCCTACACCTTTAAGCTAAAGAGAATTGGGACACCTCTACCG
TTTCACGTGAACGCGCAAAACGAGGGGTAAGGGCTAACGCACCATTCACACGGGGTGTCGGTTGACGTCA
GCCATTGCCGAACTGCATTGTGTATTTGTCGGCGCCGCTTCGGAGGCGTTGTTCGCTGCAGAAGTTGGGA
CTAGCTCAACTTTTCAAGCGCCGATGGAAGCGTCAGCCAATCAGATCGCTGTATGCAAATACACCAGCTA
GACAGTGGCCTATTGCTGACTGAATTTTATTGGCTGACACTGCTATGACGATCGCGTTAGCACCAACTTC
AGACACGCCTTCAGTCAAACGTTGACGCTGAAGCCCTGTGTGAATGGGGTGTAAGGAGTAGAATTGGGAT
TGGGCCTAACCAGGGGTGGCCAAACTCAGTCATGAAGAACCGGTACCCTGCAGAGTTTAGCTCAAACCAC
AATTAGGGTGTACTCGCACTTGGCACAGTTGCCATGAACCTTGCCGAAGCATGATTGCCCCCTCTTTCCT
GACGACCTGCACTCACACTGCATTTTGCTTGGCACAATGAAGATTGCTTTAGTTATATTGATTTAGTCTT
TTGGGATGCAGTGACACAGTCAAATATTTTACCAGATCCACCACCTTTGATGCTTGGAAATTATCAAAAA
AGTCCTCGTGCTGCATGTGTTAGGAGGTTTGATAAAGGTGCAGCTGTCGTGCAATGAGGGGTTTGCGTCT
TTATTAAACTATGACAGCTCATTGTGAAGAATGATTAATAAATTCATTTGAAACGGTCCTTTAAAAGTGA
CGTTGCGTCTTCAGTTTCAGGCTTGGGCACACTTTGCACTCACACTACAAGCGTACCACCAAAGCCCAAT
CAAACCGCGCTTTGGCACACCTCTTCCAACCGGGCCAGGACCGGCCAACTGAAGCATGCCTGGGCCTGAT
TCTGAGCAAACAAATCAGAAAACTCTCCTGGGCACGGTTTGATAACATAGTGTGAGTGCACCCTTAGACA
CACCCAAACCAGCAAATCAAGTTCTTACTAGGTGCACTAGAAACTTCCTGCCAGGTGTATTGGAGCAAGT
TGGAGCTAAACTCTGCAGTACACCAGTCCTCCAGGATGGAGTTTTGGCACTCCTGACCCTTTAGTACCAT
TATCACTGTAATTGTCACTCACCACAGAGGGGTTTGATCATACAAATATGCCTGATCTATTAATCCATAT
CAGTTGCAAATATTTGTTTGTGACCTACAGCTTAAATTATGACATGTGCTGCAAACAGCCAATCAGACGA
CACATTAGTCGATCAAACAACTAATCAAACAGCCAATCACGCTACACATCATCGCAGCAAACAGCCAATC
TCAAGACACTCAAGTGCAGTAAACAACCAAGCACACGTCACAACAACACAGCAAACAGCCAATCACACAA
CACCTGAGAGCATCAGTTTGAGTTATTTCTGGCGAGAGAGTCAACAATTTACTTCTCTGTTGAAAATTAA
GTGATACCGTTATGAGAAATATAATGTGTTTGCATGAATTTAATAAACACTGCTAGTGCTGTAATGTTTA
AGGCAACCTAGACAATATATTATATCAGTACAAATCTGTAATTTTAATAATGTAGGTTAACCAACTCTTT
AAAAGACAGTCTTTTAAAATGCTTTATTAAACAGAAGTCTACATAAATATTTAAATGTATGTTTTAATTT
TAAAATACAAACATTAATTGGCAAATTGAAGCTGTTAATGATGATGTTAATCCACAGTATTTGACTGAGT

Mib **exon 16** GTTTGTGTTGTACCTTCACAGTTGATCATGGGACTGGGCACTCAGGGGGCTGAGAAGAAGAGCGCTGCCT
CTATTGCTTGCTTCCTGGCAGCCAACGGAGCTGACCTTACCATCCGTAATAAGAAGGGCCAGTCCCCTCT
GGACCTGTGTCCTGACCCCAGCCTCTGCAAGGCCCTAGCTAAATGCCACAAAGAGAAGACAAGGTTTGTC
TATCCATTCCTAGCTCTACTAGGTGATCTAGTTAAGCTTTTGCTACTGATTAATATCTCCTCTCCTTCTG
TGCTTTACACTTTTTTTATATTAAAGTGTGTACCAAAGTGTTTTTGTTCTCTCTCATTCTCTCTGACCCG

Mib **exon 17** CAGCGGGCAGGTTGGCTCTCGAAGCCCGTCTCTAAACAGTAATAATGAGACTCTGGAGGAATGCATGGTG A←→G
TGCTCGGATATGAAAAGAGACACTCTTTTTGGCCCCTGTGGTCATATCGCCACCTGCTCTCTCTGCTCGC
CTCGTGTCAAGAAGTGTCTTATTTGCAAGGAACAGGTTCAGTCCAGAACTAAGGTACAGAATGCCAGTGG
TTTCTGTGGTTTTTCATTCATGTATTCTAATGTGTCATATGTGATCCTGAACTTCAAAGCCAGAGGTGTA
TTGTTGGCAATCGATTTTTCTTTTATGCCAAAATGTATTGAAATGTTAAGTAAAGTTTAGAGTTGTAAAA

ACTCTACTGACTACTGACATTTTAAAAGCGTCCACATCTGCATTGAGCGTGTTCTTAAATATCGCCAGTT

GGCCATTGTCTTCACGTTTTCAGCAGAAATGACTGTGATTGACAAATAACATCAGTTTCAAAGCAGTGTC

TATCAATGGATTGCTCATATGTCAGCTCATTGACAGATCAGCTGATCGACACGACTTTGAAACACTTTTT

ACATTTGTAAAATTTCTCTACCAACTAATACCAAAGTCAATACTTTTGACCCAGTGTGCTCAAATGTTAG

CTTGAAATGGACAAATGTTAATTCATTCATTTTCTTGCCGGCTTAGTCCAGGGGTGTCAATCTCAATTCC

TGGAACTAAACTGTGCAGGGCTTCGGCCCTTTTGGAACTGAGTTTGACACCTGTGGCTTAGTCCCATTAT

TAATCCAGAGCCGCCACAGCGGAATGAACCGCCAACTTATCCAGCAAGTTTTTACGCAGCGGATGCCCTT

CCAGCCGAAACTCATCTCTGGGAATAAAATGTTAAATTTCAGTATTAATTGATTCCAAAGTCGATGCTTT

TAACCCATTGTGCTCATCAGCATTGTTTAAAAGTTATCATGAAATGGGTGTTTGTAACATTTTTGTGCAA

ATTGATACCGAAGTCAATACTTTTGACCTAATTTGCTCATCACCGCTCAAACGTTAATGTGAAATTGACG

TTTGTAAAATTTCAGTACCAACAGAGACCAAAGTCGATACTTTTGACCCATTGTGCTAATCAGCATTGTT

TAAATTATACTGTGAAATGGGTGCTTGTAAAATTTTTGTACAAATTGATACCAAAGTCAATACTTTTGAC

CCAATGTGCTCATCAGCGCTCAAATGTTAGTGTGAAGTGGACGATCGTAAAATTTCAGTACCAATGATAC

CGAAGTCGATTTGAACCAATGTGCTCACCAGCAGTGGTCAAATCTTAGCATGAAATGGACGATTGTAAAA

TTTTACTACCGATTGATACAAAAGTCAATACATTTGACCCAGTGTGCTCATCAGTGCTCAAATGTTAGTG

TGAAATTGACGTTTGTAAAATTTCAGTATCAACTGATACCAAAGTTGATACTTTTGACCCAATGTGCTCA

TCAGCAGCACTCAAATGTTAGCGTGAAACGGACAATTGTAATTTTTAGTACTAATTGGTAACATAGTTGA

TGTTTTTGACCCAACATGCTCATCAGCATTGCTCAAATGTTTGCGTGAAATAGGCGATTGTAAATTCAGT

ACCAATTGATACCAAAGCCAATAGACCCAATGTGCTCATCAGCAGTGCTGAAATGTTTGCATGAAATAGG

CGATTGTTAAATTTTAGTACCAATTAATACCAAAGTCAATATTTTTGACCCATTGTGCTCATCAGTCCTC

AAATGTTAGCAAGAAATTAACGATTGTAAAATTTCAGTACCAATTCATGTGTCTTGGCCGATTCGTCCTA

CTCTTAACAAAACCAAACATCAATGGAAAATGTATTTATTCAGCTTTCATCTGATGTATAAGTCTCAATG

TCAAAAACTTGACGCCTAAGACTGGTTTTGTGGTCCAGGGTCACACATTTAACCTTTTTTCTATTGCTCA

Mib **exon 18** CCCCTAGATCGAGGAGTGTGTAGTTTGCTCTGATAAAAAGGCAGCGGTGCTCTTCCAGCCCTGTGGTCAC

ATGTGCGCCTGTGAGAGTAAGTCGGTTTTCTCCACACCTCTTCCCTTGTCATCCCATATGAAAAACAGCT

CTTTCTGTCATCTGGCTTGTCAGAATATCACACTTCACATGACTCTGATACAGAGACATGCTTCTAGATG

GATAAGAATAGGATACGGATGAATCAGACATAGTTTGAGGACATCTGTTCATTTGTCATTAGGTTATCAA

AAGAAAAGCCACATTTTTCTGATCATCTTATTATTAGTGACTGTATTTGATGTCTCTGTTAATAGGAAGG

GATGAGTTTGACAGATTTGAGATTTTTTCCTTACCTTACACAACCTGACAAAAGTCTTGTCGCCTATCCA

AGTTTTAGGAACAACAAATAATAACTTGACTTCTAGTTGATCATTTGGTATCAGAAGTGGCTCATATGAA

AGGCAAAGGCCTCTGCTTTTTTTGGGTTCATCTTCAATGCCTCCTCCATCTTACCCCAGACATTCTCAAT

AATGTTCATCTCTGGTGACTGGGCTGGCCAATCCTGGAGCACCTTGACCTTCTATGTTTTCAGGAGCTTT

GATGTGGAGGCTGAAGTATGAGAAGGAGCGCTATCCTGCTGGAGAATTTGACTTCTTCTGTTGTTTGTAA

TGTAATGGGCAGCACAAATGTCTTGATACCTCAGCCATACCCCTGCCATACACTTCGCAGATCGCTTACA

CTCCTCCATGCTGAATGAAACCCCAAACCATGATTTTTGCTTCACCAAACTTGACTGATTTCTGTGAGAA

TCGTGGGTCCATGCGGGCCCCAATAGGTCTTCTGCAGTATTTGTGATGATTGGGATGCAGCTCAACAGAA

AAATCCACCTTCTCAACTTTTCCAAATGATCAACTAGAAGTCAAGTTATTATTTGTTGCTCTTACAACTG

GAATTAACAAGACTTTTGTCAGGTAGTCTACATTGGGAAAATCCTGCATTTATTGAAGCTCAGAGCTGAT

GTAAATGTCTTAGAGGTGAGGAATAGTTTGCTCTGAGCTTACATCCCTCCTGTCTGATCCCCTGAGCTTC

ATTCTCTTTAAATACATTCTTCACTTGTCAGCAAGACCCTCTTTTTTCGTTTTATTCTGGCTTTACCTCA

GGCTGCATCCTACTGTTCTGTGGCAATTGACTATTTCTTTTTATGTTTAACAAGTTGGCTTGACAGGATA

ATGTTAGGTAATATACAGGAAATTTCCCAGGTGATATTAAACAGAGCAAGGACATTTTTCACAGTATTTC

ATATCATATTTTTGTTCTGTAGAAAGTTTTTTTTTTTCTTTGTTTGGAATAATAATATATTTAGAAAAA

TATTTTTGAATATAATTAGCCCCTTAAAGGGATGGTTCAAGTGGTTACAAACCTTTATAAGTATTTTGTT

GAACACTAAATAAGATTATTTAAAGAATGCTGAAAATCTGTAACAATTAACTTTATTACTGTTGTGTTTG

CGATGAGGACAAACGCTAAACACATGCGGGAATTTCTTCTTTCTTTCTGTTTACTAAACTCATCAGCAGT

AATGAAAACAGTTGACTGATTTGAAACAAACAGACGGCCAACCAAAAAAGTTACTCATGGATATACAAAA

GGTGGCCAACACAAAATACATACATTCCTAATATAAGAGTTCCATCTTACACTCAATACACTACATTCAC

TATAGTATAAATACAGCACTGCAACATATAAAAGAGAGATCGACTTAGAATGTCACTTACCTGTTTAATA

ATGTTTGGATCAGCTGTAGTTAGAAATTACTATCATCTTTTCTACTCTAAAGGCTGATTTATACTTCTGC

GTCAAACGTCGGCGTATGCTAGGGCGCTGACGCATAGCCCTTCACCGTGGCCGTCGCCGTCACTGACGTG

CACCTCTCGAAAAATATAACTACACGTCGCAACGACGCATAGCGTAAGCTCTGTGATTGGTCGGCTTGGT

ATCGCTGACGAGTCTGGGCGGGATCGAGAGCCACGCGAATGGCGCGAGCGATTGTTTACAAGTGTGGAGT

CCCGTGAAGGAGCTCCGGATGGAAAGTTTTGTTTTGTGTTTACCTCATAGTTAAAGTTGTTGCACGTCCG

CCGGTTCCTGCCTCAAAATGAGCGAGTTTGAGCCACTTGTACATCCTGGAAGTGTTCAAGAAAAGCAAAA

AAGCAGCGAAGAAACTCGACACAGAGGAACATTTACACCTCACTGCCCACTAGCGTTTTGGAAGTGTTAA

TGCAGACCGACAGAGACAGCGCGCAGAAGTATAAATGCACAGCTACGCGCGTTGCATGCGCCGTGGGTTA

CGCCGGTCACCTGATGCAGAAGTATAAATCAGGCTTAAGGGCTTATTATGCGGTCTCTGTCGCCATCTTG

AGAACTCTGGTCTATAGACATCAGCATTCCTTTAAAGAGTATTTATTTAATTAAAATGACTGGAAAATTA

TAATAGAACAAGCTTTTGATCATGCTGATAATTCCCTATCATAATGATAAGTCCCAAAATTAAATGTCAT

ACAGTGAGGTTATAGGGATAAGGGAGCTAATCCTTTGCTTTCTGTGCGTGTACGCGTGTGTCTGTGTGTG

Mib **exon 19** TGTGTCCTGCAGACTGTGCGAGTCTGATGAAAAAGTGTGTGCAGTGTCGGGCCGTGGTGGAGCGCCGCAC A←→G C→T

CCCCTTCGTTCTGTGCTGTGGAGGGAAAGGTATGGAGGATGCCACTGATGATGAGGACCTTAGTGAGTGA

ACTTCCACACGTTCCCGTTTTTTTGTTTTCATTTCTCTTTCCTCTCTCTCCGCCTTCGTTCTCATGTCTC

TCATCTTCAATATTTACGTTGGCTTTGTGTTTAACGCTTTCTCCGTTCCTCCTGCCCACACACATCTCTC

TCTCTCTCTCCCCATTGTTATGACTAATCTGTTATTTCCCAAATGTGCTTTCATGACTAAAATCATAGCC

CCTCCCCTTAATGCATAATATCAACACCCTGGAATGAGCTTTGTAATGCCTTTGGAATGTCTTCATAACA

CTAAATATAAGGATTTTTAGGAGGCCAGTCTTAGGGACAGGGTGTTCACAGAGTCCTAAAAATATCTTAA

ATTTTATATAAGCAAAATTTTAGGCCTTAAAGTCTTAAATTCAATAATGTTTTGTCTTAAATTGTAAACC

ATTTTAATGTCCATATAAAGTCAATTATTGTTTCCAATGGAGGCACGTGGATTACGGCGCATTTTGGGAG

TGACGCGCTCGCACCTTCCAGGCGCACCCACTTGAAATTTTCTTAACTTTTAAGAATGCTGCGAGCGTAC

CGGAAGTCACTTGACAAGAACTGACACATCAGCTTCATACTTTGCATTGAATATACACATTTCCATAGAC

AAATTATGAGACAAACACAGAACACCCTTTTAAATTTACTCCATAAATAAAACTTGTATCAGAGCTGCAG

CAATGTTTTGTCAGCTTGTCGTCCTCTGAGAAAACAGTCGATGATAGTCTTGCAATCTACGTACACCTAC

ATCCACAGAAGTTGCAATTGATAATCATATTCTAGAACAATTTGCGTCATGGTAAACCTTCATAACATTA

CATTAAATTATTCTTTATCATTCAATCACAAAGGCGTTAGGGTTCATAAGTTCACAATACACGCTTTAAT

AAACTTTTTTTTATATCATTGAAAGTCAAGGAATTTTAACTCAGGGAGTTTTTTGTCCCAAGTCATGGAA
TATCAGGGAATTTAGTTTGTCATTTTAAAATTTGACCTCCCAATTATCAAAATGTGTAATTATTACAGTC
AAAAATGACTGATTTGGTGGCCAATTTTCTGAATATAAAGCAATTCTTGCAGCATTTCTCGTATTTTGCT
TTGAAAATACACACAAGCGAACACATATTAGTTCTTCATTACTTTTTTGAGCTCAAAAATCATTGGTCTC
TTCAATTTATGGAATGGTAATACTGAAAGAAACAACGTTTTTAGCTATTAGCAACATAAAGTGTGTTGCA
TCATATTTGACGCGTAAATGACACTAAATGATCTGTTAAAATCTCGTCATCAACAGCATTATAAAAGATC
ATGTTAAAACGTTAACGTTTTAGTTATCTATACAACCAGCATATAGGGATGCAACAGTTAGAGATTTTGG
TTGTACGATTATAGTCTGAGGAATAATCACAGTTATCACGATTATTACGTGTTCTTTCATTTCAAAATAC
TACTAGTTTAGGAAAATCACATGAAAACTTCTTACATTTTAAAGTGTTATTAGTAACCAATGAATACATC
CCAAAATAACAATAAAAAAGAAAGAATATTATCCATTAATTTTTAGACTTAAAATAAATGAAAAATGTTT
TTAGCATTTAAACATAAGTAATAATTTTACATTAAAAATAAATGACAGAACAAAAATAAATAAATAGTTT
TTGTCTAATAGAAATTTAAGCATCGTATTAGTTAAGTATTTCCCTTTTAAATAGAACAAATGTAGTCGAA
GACTGTTGAACTCCTGTCTGAAAGGCACTAATATGCACGCAAACCACATGCCTCCATTGGAAATAACAAA
CTTGTGCACGTCAAAGATGTGATATGTGAATGGCCTTAGTTAAAACCCTTCTCAGCCATAGTTAAAATTA
GCCTTTGATCCAATGTGCGTGCGTATTGTTTACAGGCTCAGAACTTTAACTAGTGCACATTGGCATAACT
TTAGGTTGCAGCAGTTTTGTTCCATGCGGAAATACGGTGTTGAGAAGCCTTTATGATTAATTAACCGTGA
CAAATTAAACTGCTGTTAATAGTGAAATCGGTTAATTGTTGCATCCCTACCAGCATAGCCTTGTTCTTAT
AGGCTTTGGCTGAATGCGTGTTGCGATAATGTCATATGACGCATCTAAAACAAGCCACACACTAACTTGA
AGTATCGTACACTAGATGACCAGTTTCTGTACGCAAATATAATGCCATTTAATATAAAGCAATGAAGATG
ACGTAGAAGGAATTTGAACTGGTCCAAGGCATATCTGTGATAGAAATATGGCTCAAATGTTAGTGGGAAA
GCGACGTTGGTACTGAAATTCCAGTATCATAACAGCACTAATCTGCGCTAATTACAAATATTGCAAGCTC
TCCTAAATTTGACCTAATTTGCGTTAATTTAATCACAGAATTGGCTTTTTAGTCGATTGAAAGCCCGAGA
TATTTGGTTTTTAGAGTGGGAACCCTGTTATTTGTGACGTCATCTTTCCTTTTGTACATGATCATGACAC
TGCCCATGTTCTTTATGTTGGTTTAGTTTGAGTTCAAAGGGATACTGGTGTAAGTTCACTTTTTCTTTTT
AAACGGTGTTTATTTATAGAGAGGAGGATCTGTTTCTTTTTAGCCTCGCTTAGATGTCCAGCACATCCAT
CCACACTGACGTCTCTGCTGACCTACAGACATGCTGATCCACACACACACACACACACACACACAGTTCA
GCCGACAGAGGAATGTAGCCGCTCGCTCTTATCTTGGTCTGTCTTTAGTGCCTGAGATCTGTTTGTTGTA
AGGCGCCACTTTTTTCCACACACACAAACCAAGCGGCATCCCATTTAGACCTAACGCACTCTACCCCCGC
CTTTTCTCCACGAACACCGAGCAATGAGAGAGAAGTGCCACCATAGATGGGATTCTCTACTGCTTCGGTC
ACTTTGAAGATTCCACATATTAAAGAGGATAGACGGGTAAACCGGCTAGAGTTACGTGAAGACTTGAATA
TGAACATGGTAAACATCCGCTGTCAGATTTGACATCATTTTGGCGGAAATAAGTGTGTTAAATGACCCAA
AAAATGATTTAGTCTTGTTAGCTGACCCTTGAAACCAAGAAAAAAATTAATGTCGCAATAAATTTCCAGT
TTGGTGAAAATACATCTAATCTCACACACAAACACGCTTGTCAAGCTTAAAATTAACCAGTCTGTCTTCT
TCTTTCACACTAATCCATCATATAAAATCTCCCTTTTAACACCCATGGCTTCTAAAACCTTTGTTGTAAA
TGTTTTTTTTCCATTGAACGAAAGCCATAAAATTAGGGATGCACAATATATTGATACCTTATTGCTTGGC
TCACACAGAATTTGCTTGCGCAGATTTTTGAGTACATCATTTAGTCTATTTATTTATGAAAATGTGTGTA
AATATAAACGATTCTGCGATCACTGAATCCAACGCAAAATCAACAAAATGTCAATTTTTTGCGATCTTGC
AAAATTCTTACTTTAAATGCAAAAATAAATCGCAAAAAAATGTAATCGCAAAAAAAATTAATCATCCTAT

AAAAGATCCAGCTCTTAACCATATAATCAAATTATATTTATTTCAGTTTACAATTAATTGTTTAACGTGA
CTTACATTGTATACGCAGCGCACATGATGTATTTGAGTGTCACTCGAAAAATGACGTCTCACATGCACCC
TCACAATCAATTACATTTCATGAAAGTGGGGGAAAATAATTTAGCAGCCTGTGTAATATATACCAAACCC
CCTAGTTTTTCGTGATTTTGCAATCAATAAATCCAAGGCAAAATCAACAAATGTCCCAATTTTTACACAA
AATTGAATCGCAAAAATATTAAATAATCTTGTAAAACATCCAATTCTAAACCGTTGAATCAAATTTTATT
TATTTCAGTTTGAAATGAATTGTTTCACGTGAACAATTGACCTTATTCTAAAATGCAGAAGTGCGCCTGT
TTTCGCAATTGTTTTAGAACTTCCGATTCTGTCGCCTATGGAAGAAATGACTAGGAATGATAAACGGCAG
AAAACGGTCAAACTACTTGCTCTACAAACAAATGTTTGCATAACTATACAGACCAAGTAGGATAATATAA
TAAGAAAATGTCAGTTTGCAACATCAAACAGCGAAACGAGCAGTGTTTAATGTCTAAAAATGGATGGAAG
ACAATCAGACCGGAAGTCTCGAGCCACAAACATTCAAATGACTGCGTCCGCTTGTCGGCAAAGAATCAGG
TGAATAGACGTTTCATATGCAGCGCACTTGATGTTTTTGAGTGTCTCTTGATAAATGACGTCTCGCCTGT
GCCCTCCCATTCATTACATTACATAAAAGTGGGGGAAAATAGTTTTGCAGCCTGTGTAATGTATATCAGA
CCCCCTAGATTTTCGTGATTTTGCGATTGCTGATTCCAACGCAAAATCAACAAAATGTCACAATTTTTTT
GCGATTCTGCAAAATTCTTCATTTAAACTCAAATTAATCGCAATAAAACTTAATCATCTCATAAAACATT
CAATTCTAAACCATATAATCAAATATATTTATTTATGTTTGAAATAAATTGTTTTACGTGACTTGTAGAC
ATTGTATATGCAGCGCATGTGATATGAGTGTCTCTCGAAAAATGACGTCTCTCCTGCGCCCTCACAATCC
TAGCATTACATGCAAGTGGGGGAAATGGTTTTGCAGCCTGTGTGTGAATATATATCAGACCCCCTAGTTT
TTCATGATTTTGGGATCGCTTAAACCAATGCAAAATCAACATATTTAGCAATTTTTTGCGATCGTGCAAA
ATTCCTAATTTAAACAAAATTTTAACGGAAAAATATTAAATAATCTCAATAGCATCCAATTCTAAACCCT
ATAATCAAATTATATTTATTTCAGTTTGCAATGAATTGTTTTACGTGACTTATAGACGTTGCTCGAAAAT
TGATGTCTCACCTGCGCCCTCACAATCATTACATTACATGAAAGTAATGAAAAAATAATTGAAATCAATA
AGCTAAACTGGCCATAGTGTATGAGAGTGTGTGTGTTCCCAGTACTGGGTTGCAGCTGGAAGGGCATCTG
CTGTGTAAAACGTATGCTGGATAAGATGGCGGTTCATTCCGTTGTGGCAACCCCTGATAAAGGGACTTAG
CTGAAAGAAAATGAATGAATAAATGAAAGATCATGCCTCAGCCGGCTCAGAAAAAGTAACTCAAAAGTAA
CGTAACGCATTACTTACCATAAAAAGTAACTAAGGGTGTTTTCACATCTGTAGTTTGCTTCATTTGGTCC
GGACCAAGGGCAATAAATGATACATTGTTGCATTTTCTGCCGTCTTTGAGTCGTTTTCACACCACGCTGC
TGGCTTTGGTCCGAACCAGTTGAAACGAACCAAAATGCAGTCATCTGGCAAAATCCACATCTCTCATTGG
CCAGATGTTGTTGAACATTTCCTAAACTGCTTATTGATTGGTCAGAATTTACGTGCGAGAAAATGCCAGT
GAACTCCCGCACGTAAACAAAACCGGCAGACACAAAATGTCGTTTTTACACTGGAGGGACGACTGCGCTG
CTGATTGTATCCCTGCTTTAGACAAACTCTACATTTCGAGAATGCAGGTCTTTTTGGTCCACTTTTGGTG
CGCGCTCAAGTATGATTGCTGCATTTTCCCCTCCCCAAACGAACCGCACCAAGAGGAAAAACGAACTCCA
ATGCGATTCAGTCGAACTAAATAAGACAGGTGTGAAAGCACCCTTAGTAAGGCAGCTAGTTACTTTTTTG
GGGGAGTAACTCAATATTGTAATACACTACTTTCTAAAGTAACTTTCCCAACACTGGCCATAGTGATGGC
AGATAATTAAATCCAACTTGGATTGGAGAGAAAAAAAGCACAGAAGTTGTAGTAGTATATAAAGATTGAT
CATAAAGCATTGGGTGATCACAGACCACAATACTAGACTGCGAAAAAATAATAAAAAAATGCTTTTCTTG
TAGTTTTTGCCTCGTTTCTAGTCCAAATATCTAAAAATTCTTAAATCAAGAAGCGTTTGAAAGCATAATG
AAAACTAAGTGAGCTTTCCCATAATATAAGCTAAATAATCTGCCATTGGGGTAAGCGAAAACATTCTTTT
GTCAATAGTATATATTTCGGATTTATTGATTAGTGACACTAAATTTTAAAAAACAAACAAAAAGACCCTG

AAACATTAGCTCGAGTTTAAATATTGCTGCATCCCTACATCAAATCCTTCTATTTTTGAAGGTGGCTGTC
ATCCCTTGTGCTGTGAAGGATAGTCCTGCAGCCATAAATGATGTATACCTTAATCTACACCGAAGCCTCC
CATCCATAACAGCTTGTGTCAAAAACTTGCTCTCCCCCTTTGCTCCCTCTACTGACTATTTATAAGTAGT
ATATGCCAAAGGCCAGGCTGGATTGTCTCCGGATCCAGCAGCCAGGGGTTGCATGCATTACGTAACACTG

Mib **exon 20** TGACTGGTTGCTTCTCAGCAGGGGGCTCTAACTCAATGGCAGGGGGGTCGCAGGATCTTCTCCAGCCCAA
CAATCTGGCCCTAAGTTGGTGTAAGTATTCAATTCCTCACCCCAACTTCTTCCTGCCTCAACTTCTTCCT
GCTCCTTTGGGCTCAAGAGTCCGCATCTTCACCCCTGCATTGGTCATTCTCTCCAGTTGGAGAGGCTGTC
GAGTGGCTCGCTGCGTTTTCAGTGCTGTGGGTTGAGTGCTGAAGTCAGAAAAGGCACAAAACTTTCAGAA
TTATAAAGTAATTCAGACATTTAAGAAGTTAGAAGGCTCTGGGGTCTGCAAACGCCCAGGCATGAACCTT
TATAAGCTACATGATTGGCTGTGGGCCTGGTGTGTATATTTTGTTCTCAACGCATTTTGCTTTGCATTTC

Mib **exon 21** AGCCAGTGGAAATATCCCAGCCTTGCAGAGGGACAAAGACAACACTAATGTCAACGCAGATGTACAGAAG
CTGCAACAGCAACTGCAGGATATCAAAGAGCAGGTGAGTATGACAGTGTGCAGATGGGATGGACAATTTT G←→A
AATTCCTTTATTTTTTGTAGAATAATAATCAACTGTATTTTATAGTTCTTTTACAAGTAGGGGTGGGTAC
CGAAACCAGGTACCTATGTAGCCAGTATGCGCCAGATTGCATCAGCATATGCATTTCAGTGCCACTTGTG
CTGCGATATGAACATAAAAGCATCAAAAGCATCTGTCACACGATCTCTAAACATTTAATCCGAAACAACT
TTGAGGGATACAGACACCGTTGGTGATGTTAGGCATTTGTTCTTGTTGCTTAGGGAAGGCACGTAGGAAA
CAAGCGCAGTATAGCGCATCCGGTGATCGACTCTCTTCAGATTCATCAACACGCAGGATCGCGTTCATCA
AATTTGCTTAAGCTAAGCGTTCTAAAGTATGCAAGGATTCTGACAGGGCAACGTGAAGCACATGCGTTAC
AAAAGTTGCGTGTGGGGGGAAACACGTCAGTCAGTCAGCATGTCACCTTAAAGGGTTAAATGAATAAGGC
TCAGCACTACTACCGTTAAAGTTTGCGCTGTTATAATTCACTTACCTTTTAATCAGATTATAATCTGCTA
TTTAAATATAATGCTAATATTCGGCACAAATCCATCAGTTTCCCTTTCCGGCTGTTCTATCTATGAATAA
ACTATGTAAAAGCACTGTCATGCGTGTTTCCTCGTCGGTTAGTAACGCTATATTTCATTGCACAACAATT
AATCTATCAGGCTTTTTGGCAAAAAAATAGAGAAATCACAGTTTCATTTATTGTTATTAGATTATGTTTA
AATTTCACCTCTCCTGCTGAGCTAAGCTGTTGAATGGTTAGCGTATGAGGCGGCTAGGCTAAGCTAGCTT
TCATTTTGATTGAATTATTCTCTAATCGGTTGCCTATTATGTAGTGAATATACCCCTGTACTGCACACTG
CAGTCCTTTTTTTGTCTGGCTTTCTCGGATATCTCACCTGTTCGCCAAAAATGACATTATATCCCTGGCG
ATGTCTGAAACCGGCGCACACACATTGAAAAAGCCGTGCCAGGCACGAAAGCTTGACAGGCGTAGCAACA
GTAACTAAGGAGGGCGGGGCTTAGCGAAGGGTCCACTGCTGTAAGATAGTGTTAGATTTAGCTGAAAGTG
ATGCAGTTTAAACAGTGAATTGAATCTATTTACCGTCTGTCTACCCACAGATTTGCACCTCTAGATCTTC

Mib **exon 22** AGGATGTTTTGTTTTATTGCCGTTATGGCTCATTGAACATGTTTCTCTTCCTCTCAGACTATGTGCCCGG
TGTGTCTGGATCGTCTGAAGAACATGATCTTCATGTGTGGCCACGGCACCTGTCAGCTGTGTGGAGACCG
CATGAGCGAGTGCCCGATCTGCCGCAAGGCCATCGAGCGCCGAATCCTGCTTTACTAGAGCGCCGCGTCT
GTGTGGTTCTCATAAGCACCTTCCCCGGATCTGCAAGGCTCTCCTTGTCTCACGCTAAGCTAAGAGTCAT
CAGTGTTACCACATCCATCTTTGGTTTCATCTTCAATATTAACGTTTTAATTATGTTCGAGGAGACAGAA

**Figure S11.** The assembled *mib* genomic nucleotide sequence from scaffolds 3504 and 3540.

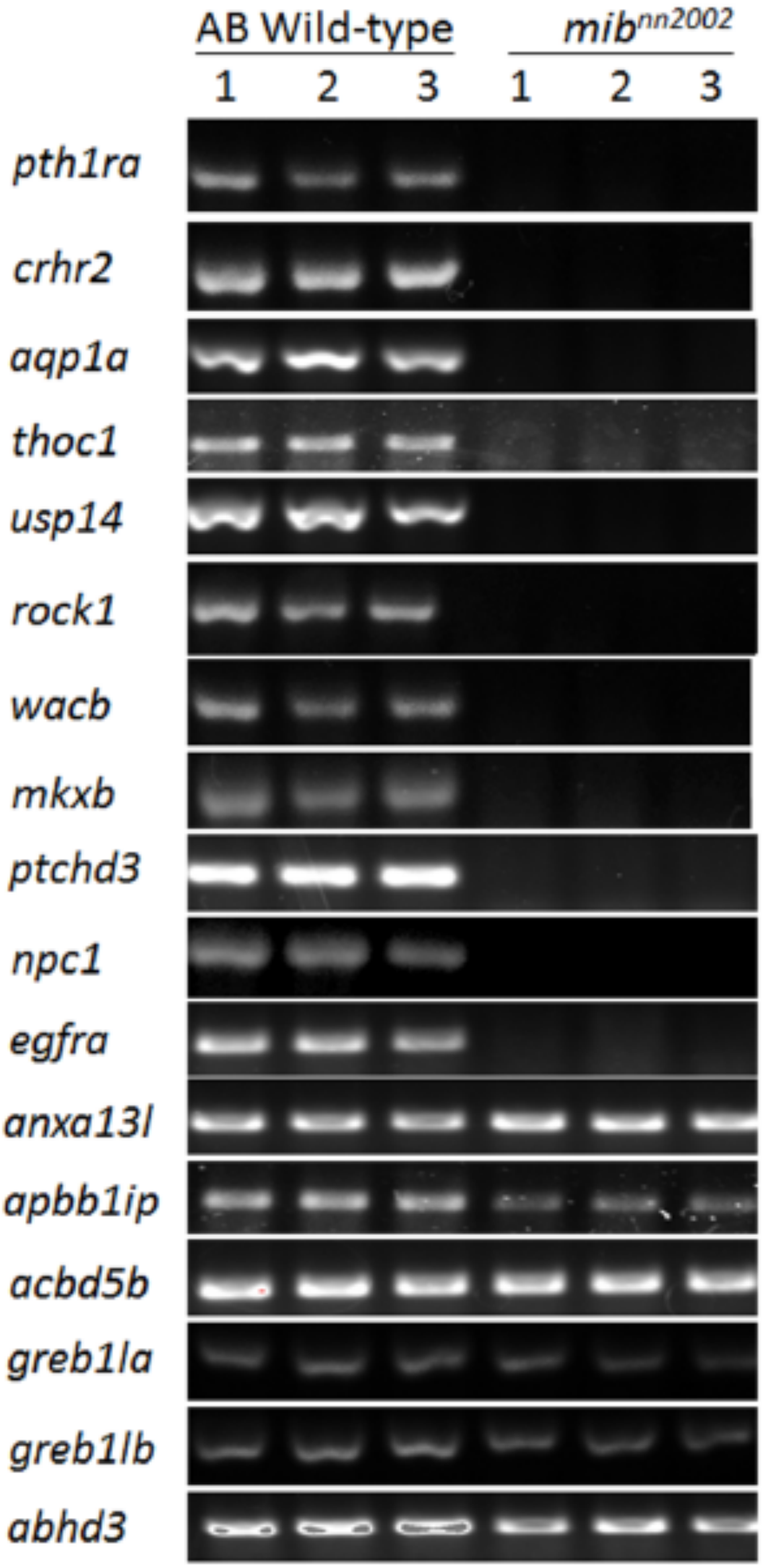


**Figure S12.** Genomic PCR of genes around *mib* locus on LG2 in wt embryos and $mib^{nn2002}$ mutants. Genes listed in the LG2 hypothetical genomic sequences were examined by PCR. The genomic sequence located much close to the terminal (*cldn1* end) of LG2 than *ap2m1a* were not amplified. The not-amplified genes include genes not assembled in LG2, such as *mkxb*, *rab18b* (in Fig. 7), *ptchd3*, *yme1l1b* (in Fig. 7), *mib* (in Fig. 7), *gata6* (in Fig. 7) and *cables1* (in Fig. 7).

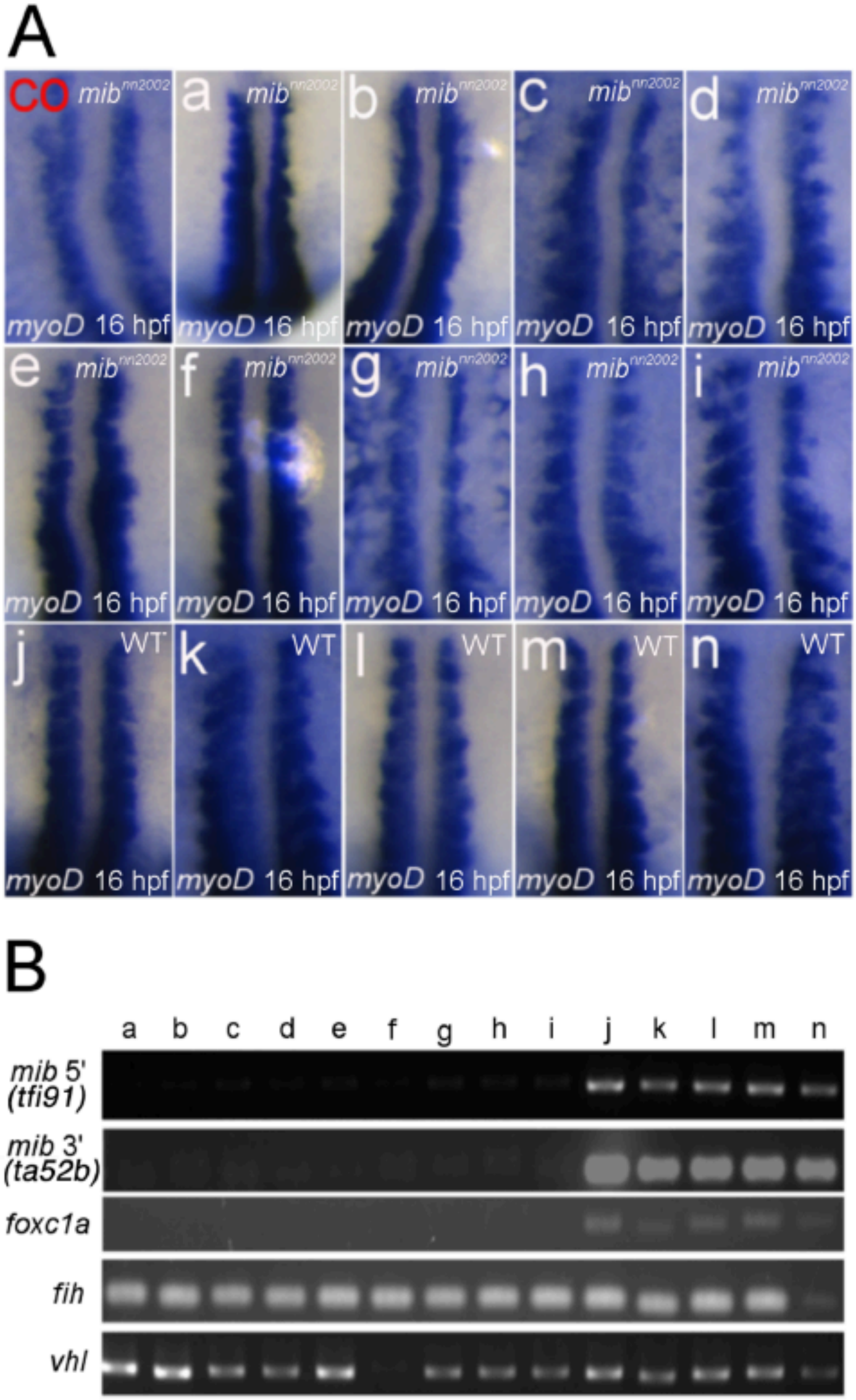


**Figure S13.** (A) The *myoD* WISH results from 20 pg *foxc1a* mRNA-injected (a–i) *mib*$^{nn2002}$ homozygotes, and (j–n) WT (siblings) and (co) non-injected *mib*$^{nn2002}$ homozygotes and (B) the corresponding genotyping results were shown.

Two major rescued features of *myoD* expression in the anterior somites are segmental and laterally-extended *myoD* pattern, compared (a–n) to (co). The *myoD* expression pattern in the anterior somites was asymmetrically (c, d, h, i) and symmetrically (a, e, f) rescued in *myoD*-overexpressed *mib*$^{nn2002}$ homozygotes, while (b, g) *myoD*-overexpressed *mib*$^{nn2002}$ homozygotes were still indistinguishable from control (co). The genomic fragments of *mib* 5' (amplified with tfi91 primers), *mib* 3' (amplified with ta52b primers), and *foxc1a* (amplified with *foxc1a* primers) were not detected in the homozygous mutants, while the control fragments, *fih* (amplified with *fih* primers) and *vhl* (amplified with *vhl* primers), were detected in the embryos except for *vhl* PCR sample f and *fih* PCR sample n, which might be due to the DNA damage or cross-links that are generated by paraformaldehyde fixation during the WISH procedure[1].

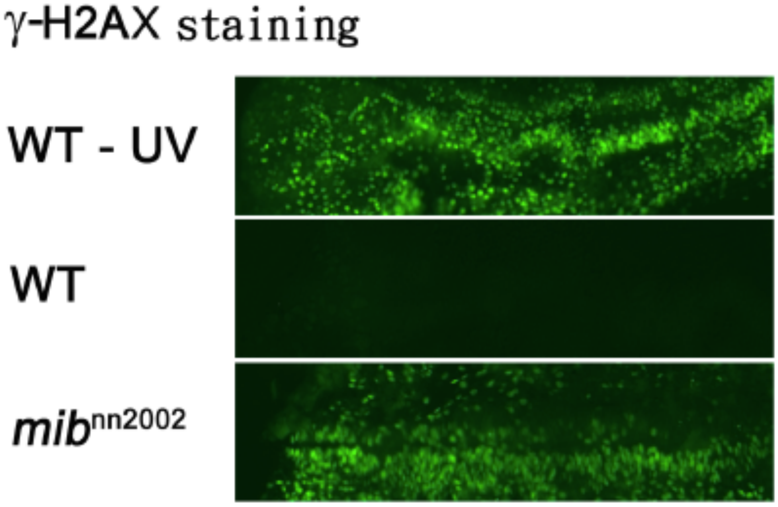


**Figure S14.** γ-H2AX staining is highly increased in *mib*$^{nn2002}$ mutants.
γ-H2AX, representing the event of DNA damage, was only detected in *mib*$^{nn2002}$ mutants and UV exposed wt embryos (WT-UV).

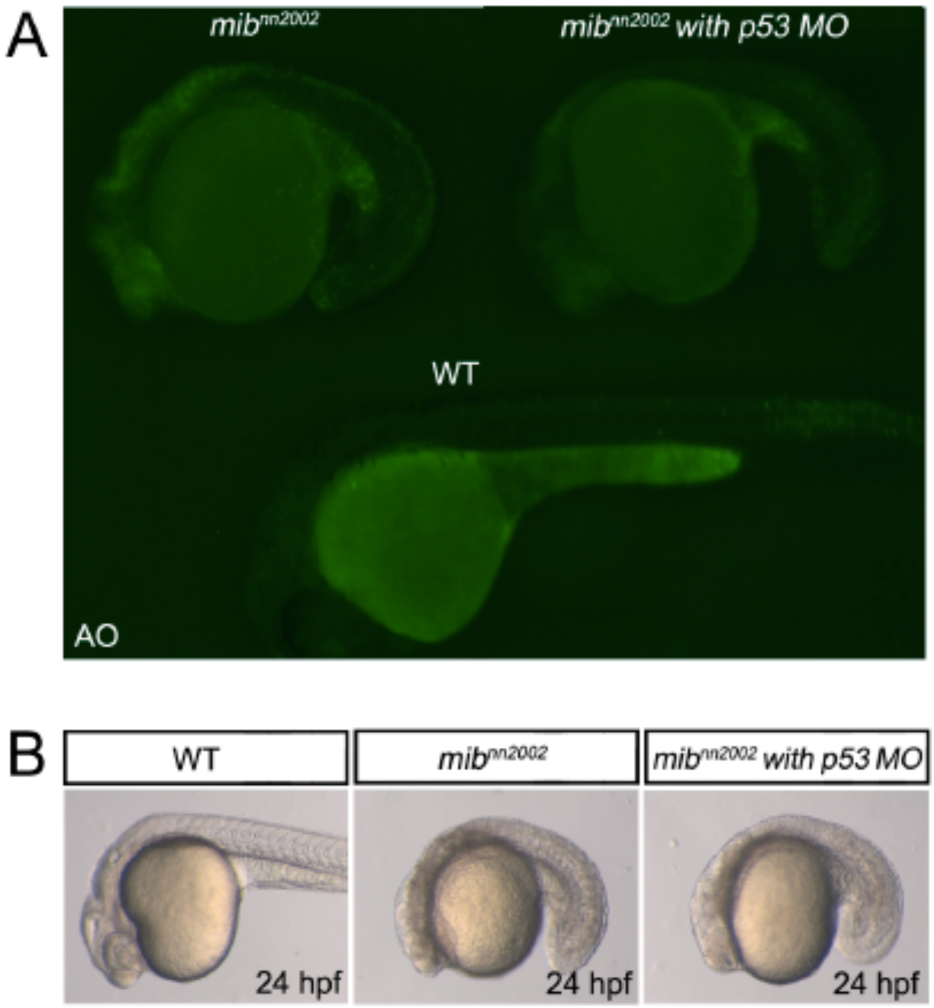


**Figure S15.** Cell death in *mib*$^{nn2002}$ mutants is *p53*-dependent.
(A) Acridine orange (AO) staining in *mib*$^{nn2002}$, *mib*$^{nn2002}$ injected with *p53* morpholino and WT. AO staining was partially reduced in *mib*$^{nn2002}$ mutants with *p53* morpholino injection. (B) Nomarski images of WT, *mib*$^{nn2002}$ and *mib*$^{nn2002}$ injected with *p53* morpholino. The deposition of dark granules in *mib*$^{nn2002}$ mutants was suppressed by *p53* down-regulation. This result suggests that the cell death in *mib*$^{nn2002}$ mutants is *p53*-dependent.

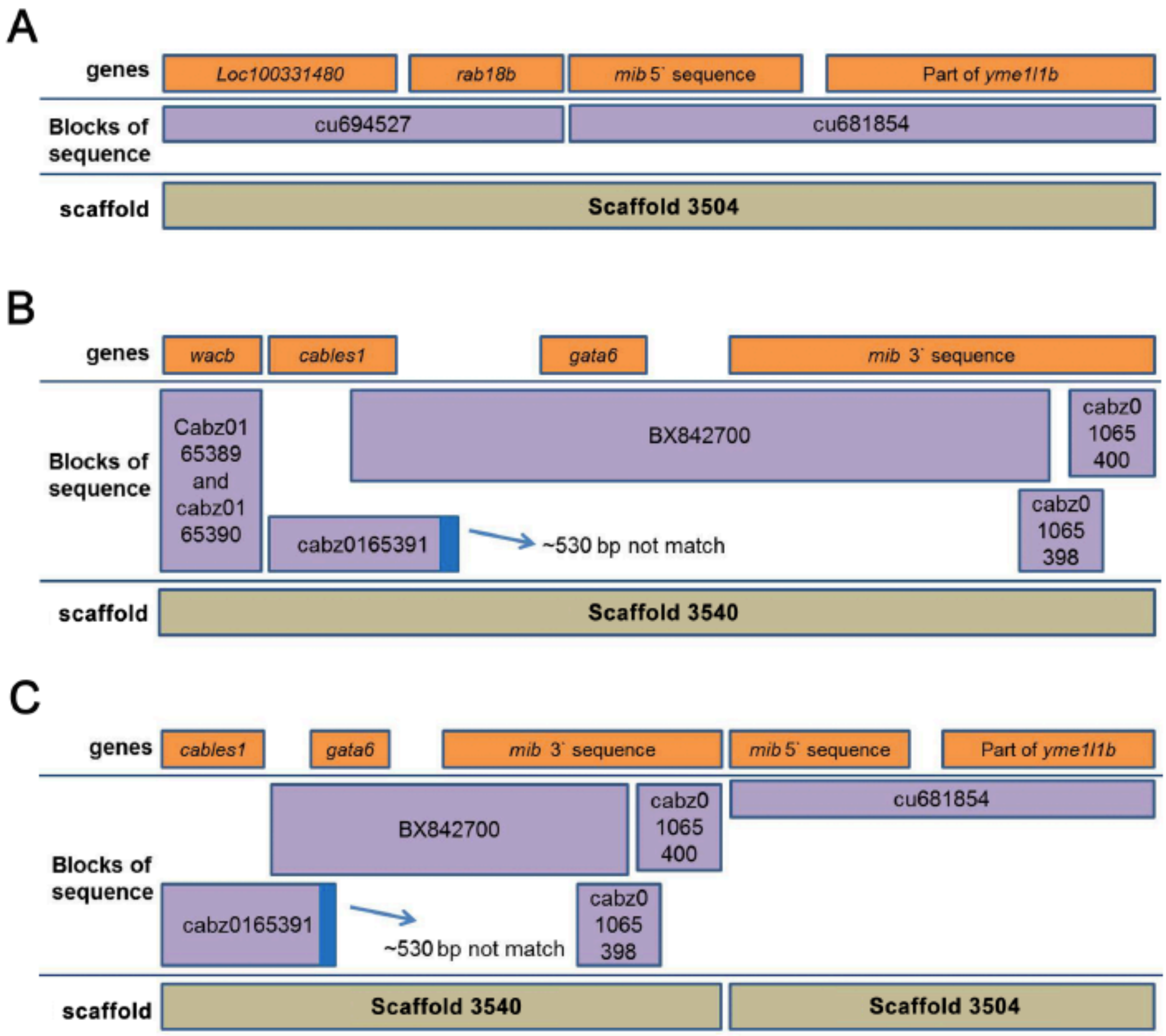


**Figure S16.** The assembly of *mib* genomic sequence.
(A) Scaffold 3504 of assembling 5' *mib* genomic sequence is illustrated. Scaffold 3504 contains cu694527 and cu681854 sequences. There is no overlap between these two sequences. (B) Scaffold 3540 of assembling of 3' *mib* genomic sequence is illustrated. Scaffold 3540 contains cabz0165389, cabz0165390, cabz0165391, BX842700, cabz01065398 and cabz01065400 sequences. There is no overlap between sequences coding for *wacb* and *cables1*. There is a 530 bp mismatch between cabz0165391 and BX842700. (C) Hypothetical assembly of *mib* genomic sequence is illustrated. In the hypothetical map, the *mib* is located between genes *gata6* and *yme1l1b*.

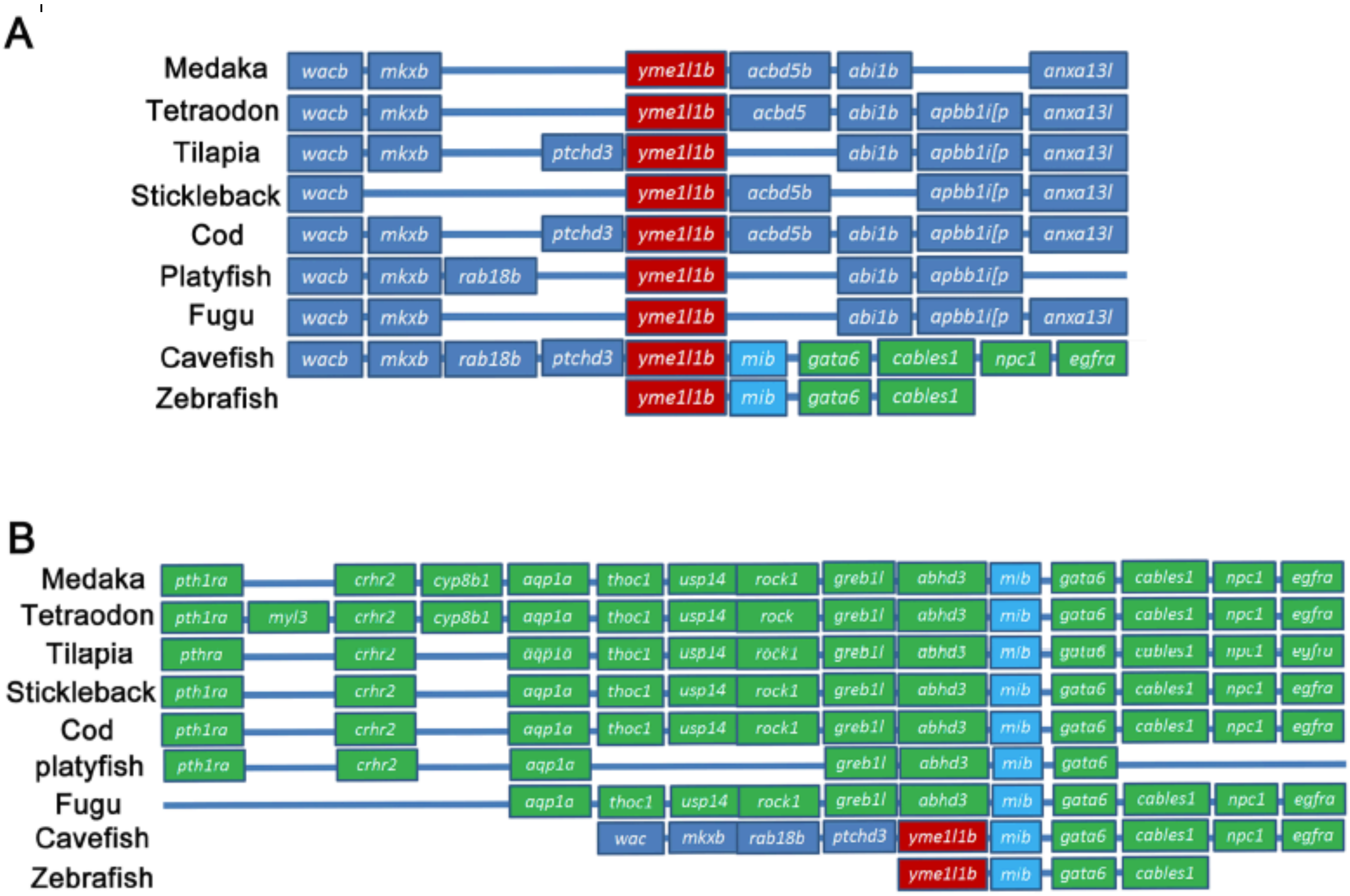


**Figure S17.** The synteny of genes near *mib* in different fish species.
The synteny in (A) is arranged by aligning the gene *yme1l1b*. The synteny in (B) is arranged by aligning the gene *mib*. The synteny of genes nearby *mib* in zebrafish is much more similar to that of cavefish.

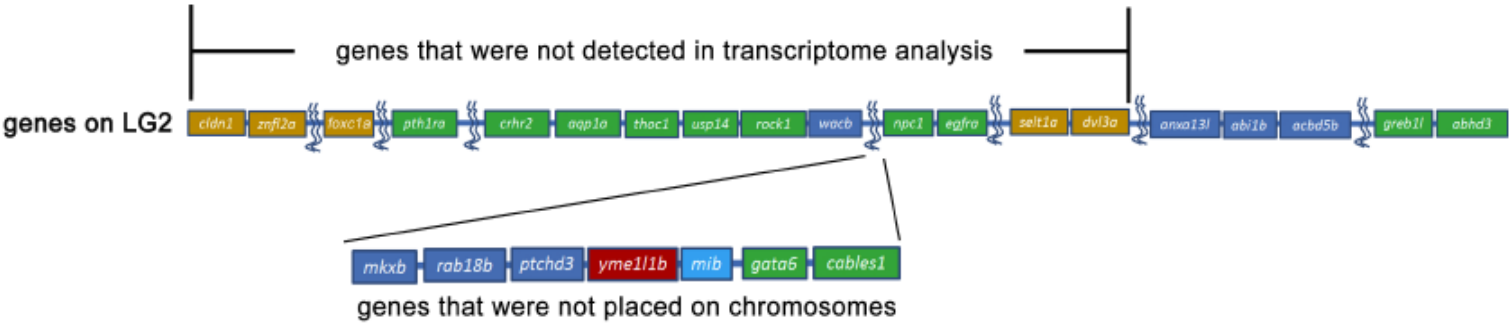


**Figure S18.** The hypothetical genomic sequence of zebrafish LG2 around *mib* locus.
The location of genes, *cldn1*, *znfl2a* and *foxc1a*, near the chromosome terminal are based on Zv9 zebrafish Ensembl sequence. The gene positions, such as *pth1a*, *crhr2*, *aqp1a*, *thoc1*, *usp14*, *rock1* and *wacb*, are based on the LG2 Ensembl sequence (supplementary Table 4), which match the gene syntenies left to *greb1l* in Medaka, Tetarodon, Tilapia, Stickleback, Cod and Platyfish (supplementary Fig. 17B). This also suggests there is a recombination happened between *wacb* and *rock1* in zebrafish while it was compared with fishes like Medaka. Based on the synteny of cavefish in supplementary Fig. 17, genes, such as *mkxb*, *rab18b*, *ptchd3*, *yme1l1b*, *mib*, *gata6* and *cables1* were placed on the right hand side of *wacb* (these genes are not assembled in any LGs and their genomic DNA is not PCR-amplifiable in $mib^{nn2002}$ mutants). *npc1* and *egfra* were placed on the right hand side of the *mib* scaffold based on the synteny of Cavefish, although *egfra* is much closer to chromosome terminal than *npc1* in Zv9 Ensembl sequence. The location of *selt1a*, *dvl3a*, *anxa13l*, *abi1b*, *acbd5b*, *greb1l* and *abhd3* were based on the Zv9 Ensembl sequence. *anxa13l*, *abi1b* and *acbd5b* were still located near each other as expected (synteny from Medaka to *Fugu*, though *apbb1ip* is located in chromosome 24 in Zv9 Ensembl sequence). *greb1l* and *abhd3* are also located nearby position as expected in synteny. The genes much closer to chromosome terminal than *dvl3a* in the reconsitituted hypothetical map are not detected by RNA-seq and genomic PCR.

## References

1. Clingenpeel, S., Schwientek, P., Hugenholtz, P. & Woyke, T. Effects of sample treatments on genome recovery via single-cell genomics. *ISME J.* **8,** 2546–2549 (2014).
